# Design and Synthesis of Visible-Light-Responsive Azobenzene Building Blocks for Chemical Biology

**DOI:** 10.1021/acs.joc.2c01777

**Published:** 2022-10-26

**Authors:** Jana Volarić, Jeffrey Buter, Albert M. Schulte, Keimpe-Oeds van den Berg, Eduardo Santamaría-Aranda, Wiktor Szymanski, Ben L. Feringa

**Affiliations:** † Stratingh Institute for Chemistry, University of Groningen, 9747 AG Groningen, The Netherlands; ‡ Departamento de Química, Universidad de la Rioja, Centro de investigación en Síntesis Química, Madre de Dios 53, 26006 Logroño, Spain; § Department of Radiology, Medical Imaging, Center, University of Groningen, University Medical Center Groningen, 9713 GZ Groningen, The Netherlands

## Abstract

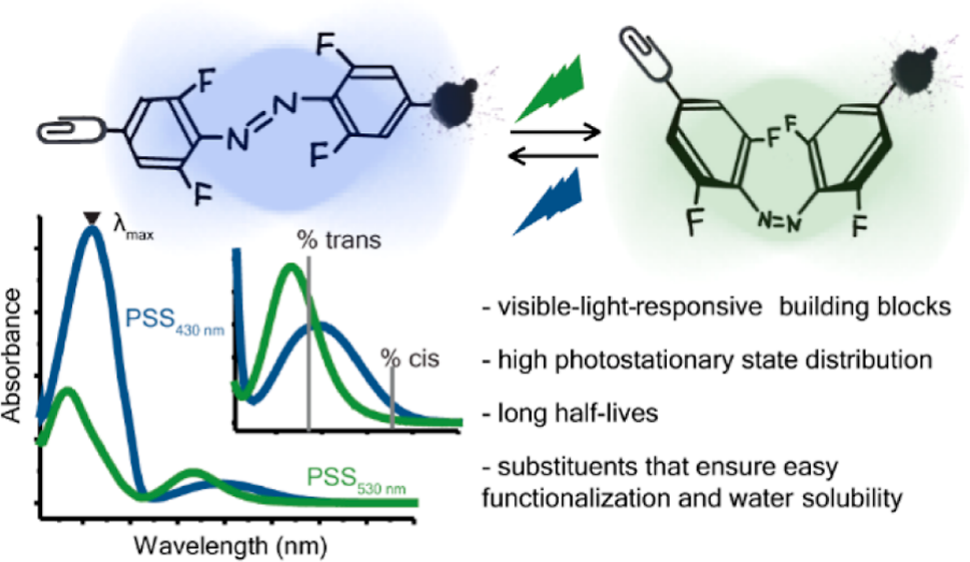

Tetra-*ortho*-fluoro-azobenzenes are a class of photoswitches useful for the construction of visible-light-controlled molecular systems. They can be used to achieve spatio-temporal control over the properties of a chosen bioactive molecule. However, the introduction of different substituents to the tetra-fluoro-azobenzene core can significantly affect the photochemical properties of the switch and compromise biocompatibility. Herein, we explored the effect of useful substituents, such as functionalization points, attachment handles, and water-solubilizing groups, on the photochemical properties of this photochromic system. In general, all the tested fluorinated azobenzenes exhibited favorable photochemical properties, such as high photostationary state distribution and long half-lives, both in organic solvents and in water. One of the azobenzene building blocks was functionalized with a trehalose group to enable the uptake of the photoswitch into mycobacteria. Following metabolic uptake and incorporation of the trehalose-based azobenzene in the mycobacterial cell wall, we demonstrated photoswitching of the azobenzene in the isolated total lipid extract.

## Introduction

Molecular photoswitches are powerful tools to both manipulate and study biological systems.^1−5^ Among those, azobenzenes are the most widely used photochromic molecules, in particular due to the large change in geometry and polarity they undergo upon photoswitching.^5−9^ Their synthetic accessibility enables the adaptation of the system for the desired application while mainly retaining excellent photochemical properties.

However, a disadvantage of the classical azobenzene photochromes is the need for using UV light for photoswitching of the stable *trans* isomer to the metastable *cis* isomer. UV light is not optimal for biological applications since it causes damage to living cells and has a low penetration depth.^10−13^ While the azobenzene core does absorb in the visible-light region, the n−π* transition bands of both isomers overlap, preventing selective addressing of the isomers with visible light. Nevertheless, many adaptations have been made to enable the operation of the azobenzene switch with visible light by separating the n−π* absorption bands of the two isomers.^14−18^ For example, the presence of a restricting bridge in diazocines distorts the planarity of the molecule, thus shifting the n−π* bands of the respective isomers.^19−22^ Furthermore, the introduction of substituents in all *ortho*-positions to the azo-bond, such as methoxy, chloro, or fluoro groups, results in n−π* band separation.^14,17,23,24^ Both for the tetra-*ortho*-methoxy^14,17^ and the tetra-*ortho*-chloro system,^15,25,26^ the band shift is caused by geometry distortion (nonplanar conformation) of the *trans* isomer. Conversely, for the *ortho*-fluorinated azobenzene, the electron-withdrawing fluorine atoms stabilize the nonbonding electron pairs around the azo-bond, thus lowering the energy of the n-orbital.^27,28^

In the recent years, the development of visible-light-responsive azobenzenes has driven their application in a biological context,^2,5^ generating the need for functionalized visible-light-responsive azobenzene building blocks which can dissolve and operate in aqueous environments. Due to the favorable photochemical properties of tetra-*ortho*-fluoro azobenzenes, it comes as no surprise that this system has been most widely applied for various biological targets, both as freely diffusing effectors,^29−33^ as well as through incorporation into proteins.^34−36^ Bioactive molecules containing the fluorinated azobenzene were used to reversibly modulate the circadian rhythm,^37^ regulate the activity of the carbonic anhydrase enzyme *in vivo*,^32^ control the activity of muscarinic acetylcholine receptors,^31^ photoregulate transmembrane transport,^30^ as well as to intercalate DNA.^29^ Furthermore, fluorinated azobenzene-amino acids were incorporated into proteins via genetic code expansion^34,35^ or into the peptide backbone^36^ and peptide nucleic acid chain^38^ via solid-phase peptide synthesis.

It was reported by the Hecht group that the introduction of an electron-donating group to the *ortho*-fluorinated azobenzene in the *para* positions negatively influenced the photochemical properties as the n−π* band separation was smaller when compared to the unsubstituted parent azobenzene.^27,28^ However, *ortho*-fluorination lowers the energy of the n-orbital only for the *cis* isomer, which causes the separation of the two n−π* transitions. Larger overlap of the n−π* bands of each isomer results in a lower photostationary state distribution (PSD) upon irradiation. Electron-withdrawing groups (EWGs) on the other hand have the opposite effect as they help to lower the energy of the *trans* isomer, thus resulting in a larger band separation.^27,28^ Often, fluorinated azobenzenes feature impressively long half-lives, likely due to the stabilization effect of the EWG fluoro substituents in the *cis* isomer.^27,28^

Usually, tetra-*ortho*-fluoro azobenzenes do not, or hardly, absorb in the red-light region of the spectrum (>600 nm). In an attempt to further red-shift the tetra-*ortho*-fluoro azobenzenes, Priimagi and co-authors report an extensive library of combined tetra-*ortho*-substituted fluorinated and aminated azobenzenes.^39^ The addition of tertiary amines in the *ortho*-positions of classical azobenzenes increases the molar absorptivity in the visible region and ensures resistance toward glutathione (GSH) reduction, yet results in diminishing the half-lives into the second range.^17,40^ Toward applying wavelengths for photoswitching within the “therapeutic window” (600–900 nm) where light has the maximum tissue-penetration depth,^41^ Pianowski and colleagues reported that introducing sp^2^-hybridized substituents that extend the conjugated π-electron system resulted in further red-shifting of the n−π* absorption bands.^42^ Notably, while the functionalized switch could successfully switch in an aqueous environment, in the presence of GSH at 25 °C, half of the compound degraded within 10 h.

The various applications of the tetra-*ortho*-fluoro azobenzene indicate the importance of this switch, thus suggesting the necessity for further development of easily modifiable building blocks featuring this useful chromophore. Here, we focus on the tetra-*ortho*-fluoro-azobenzene,^27−29,38^ exploring the development and applications of this system by taking advantage of its electron-poor nature, which allows for direct and hitherto underexplored functionalization via a nucleophilic aromatic substitution (S_N_Ar) reaction. While there are initial reports on utilizing the S_N_Ar reactivity of fluorinated azobenzenes for derivatization in the *ortho* positions^39,43^ or demonstrating the reactivity in the *para* position,^35 ,44^ this feature was never utilized for useful functionalization in the *para* positions, especially in the chemical biology context.

## Results and Discussion

### Design

**Figure 1:**
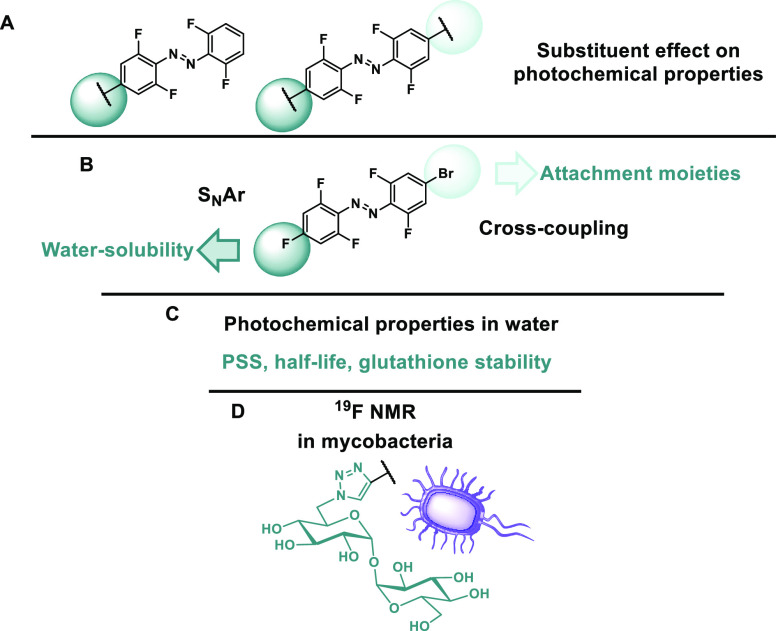
Outline of the goals of this study: (A) investigating the photochemical properties of mono-substituted and bis-substituted tetra-*ortho*-fluoro azobenzene building blocks. (B) Functionalization of the bis-substituted azobenzene for water solubility and further functionalization. (C) Studying the photochemical properties of water-soluble building blocks and their stability in the presence of GSH. (D) Application of the designed building block for functionalization with a bioactive molecule, trehalose, and studying the photochemical properties after incorporation into a mycobacterial cell wall.

We designed and synthesized tetra-*ortho*-fluoro-substituted azobenzene building blocks, with functional groups useful for biological applications, and studied their photochemical properties. The tetra-*ortho*-fluoro groups enable the operation of these photoswitches by using exclusively visible light, namely, green and blue, which is nonharmful to biological systems. Besides visible-light control, water solubility is a crucial, yet often overlooked, the parameter for biological applications.^5^ It is not trivial to dissolve inherently lipophilic aromatic photoswitches in water while also preserving their photochemical properties. Therefore, we aimed to investigate the photochemical properties of several fluorinated azobenzene molecules featuring a water-solubilizing group in an aqueous medium (Figure 1C). Furthermore, since in living organisms, the cellular environment is reducing due to, among others, high amounts of GSH,^45−47^ we evaluated the stability of the designed molecules in the presence of 10 mM GSH.

The electron-poor nature of highly fluorinated azobenzenes, besides lending them unique photochemical properties, also potentially enables their new route through the S_N_Ar reaction. S_N_Ar is particularly interesting as it can be applied orthogonally to classically used cross-coupling reactions for the synthesis of bifunctionally modified azobenzene building blocks (Figure 1). With this in mind, we aimed to use the S_N_Ar approach to investigate the effect of a single substituent in the *para* position on the photochemical properties, followed by bis-*para*-substituted molecules (Figure 1A). In both cases, we chose the substituent moieties that are useful handles for modification. The azobenzenes carrying two substituents in the *para* positions can further be functionalized to act as a symmetric crosslinker or used for orthogonal functionalization on both sides (Figure 1B), as well as decorated with water-solubilizing groups (Figure 1C).

Finally, we designed and synthesized a tetra-*ortho*-fluoro azobenzene molecule carrying the α,α-trehalose moiety (Figure 1D). It has been previously demonstrated that chemically modified trehalose can be metabolically incorporated in the outer cell wall of *Mycobacterium tuberculosis*, the causative agent of the tuberculosis disease, by esterification of the trehalose with mycolic acids to form trehalose monomycolates.^48^ Remarkably, large structural modifications on trehalose are tolerated, and trehalose probes have been used to label mycobacteria with photosensitizers,^49^ fluorescent probes,^50−52^ and even nanoparticles.^53−55^ Therefore, we anticipated that our azobenzene structure carrying a trehalose molecule can be metabolically incorporated into the mycobacterium, allowing us to not only confirm its metabolic stability but also to observe photoswitching of the molecule within the complex mycobacterial cell wall via ^19^F NMR spectroscopy.

### Effect of Functional Substituents on Photochemical Properties

**Figure 2:**
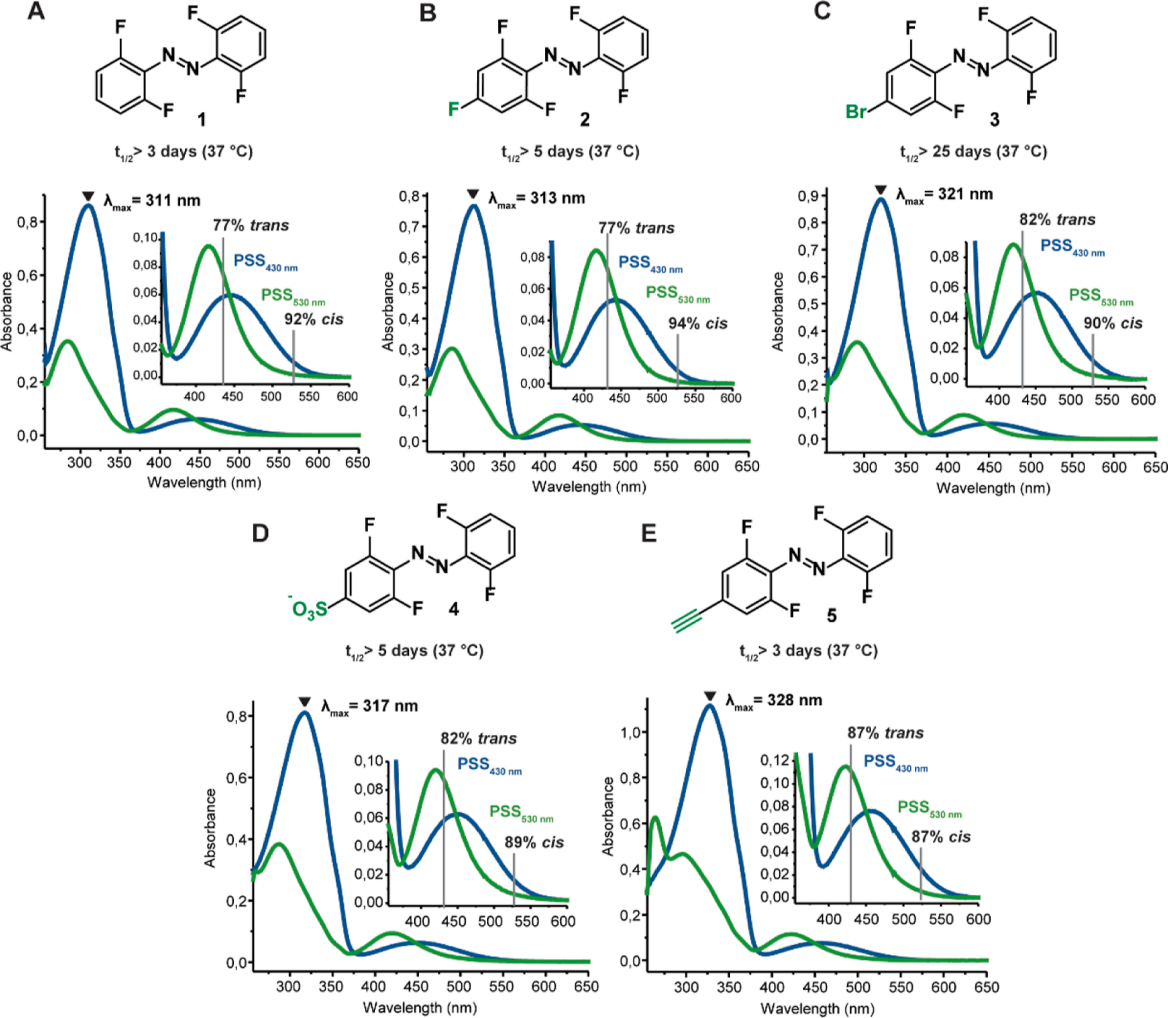
Effect of a single *para*-substituent on the photochemical properties of the tetra-*ortho*-fluoro azobenzene in DMSO (A–E). All UV–vis spectra are measured at 50 μM concentration in DMSO at 25 °C. Half-lives are determined in DMSO at 37 °C. PSDs were determined via ^19^F NMR spectroscopy in DMSO-*d*_6_.

First, we investigated the effect of a single substituent in the *para* position on the photochemical properties of the tetra-*ortho*-fluoro azobenzene core. For this, we chose moieties applicable for further modification via S_N_Ar (−F, **2**) and cross-coupling reactions (−Br, **3**), as well as for solubilization in aqueous environments (−SO_3_^–^, **4**) and a click reaction handle (alkyne, **5**) (Figure 2). The detailed synthetic procedures for the preparation of compounds **1**–**5** and their precursors can be found in the Methods section.

Compounds **1–5** were dissolved in DMSO to study their photochemical properties (Figure 2). Compared to the unsubstituted tetra-*ortho*-fluoro-azobenzene structure (compound **1**), all substituted compounds exhibited a slight (from 311 nm for **1** to maximal 328 nm for **5**) bathochromic shift of the absorbance maximum at the photostationary state (PSS_430nm_), where the switch is predominantly in the *trans*-form. Upon closer inspection of the visible-light region of the spectrum, where the n−π* transition is located, the characteristic band separation of the S_0_–S_1_ absorption bands of two isomers is observed for all compounds (Figure 2). For all substituted azobenzenes, a slight bathochromic shift of the respective S_0_–S_1_ absorption maxima is observed, compared to the unsubstituted compound (Figure S99). Upon irradiation with 530 and 430 nm light, the observed PSDs remained very high for all introduced substituents, with values of 87–94% *cis* at PSS_530nm_ and 77–87% *trans* at PSS_430nm_. Finally, all compounds exhibited very long half-lives of the metastable *cis* states in DMSO, even at the physiological temperature (37 °C), namely, over 2–100 days (see Supporting Information sections 6.1 and 6.2). In general, the photochemical properties of compounds **1–5** were similar to previously reported tetra-*ortho*-fluoro azobenzenes with a single substituent in the *para* position. Namely, several known compounds feature relatively long half-lives in organic solvents (e.g., 11^42^ or 30 h in ACN at 60 °C^28^), while in aqueous solutions, the metastable *cis* isomer was stable for 1–4 days at 37 °C.^36 ,38^ The previously published compounds also reached high PSD values in both organic and aqueous solvents when irradiated with 520–530 nm (82–95% *cis*) and 390–405 nm (90–95% *trans*).

**Figure 3:**
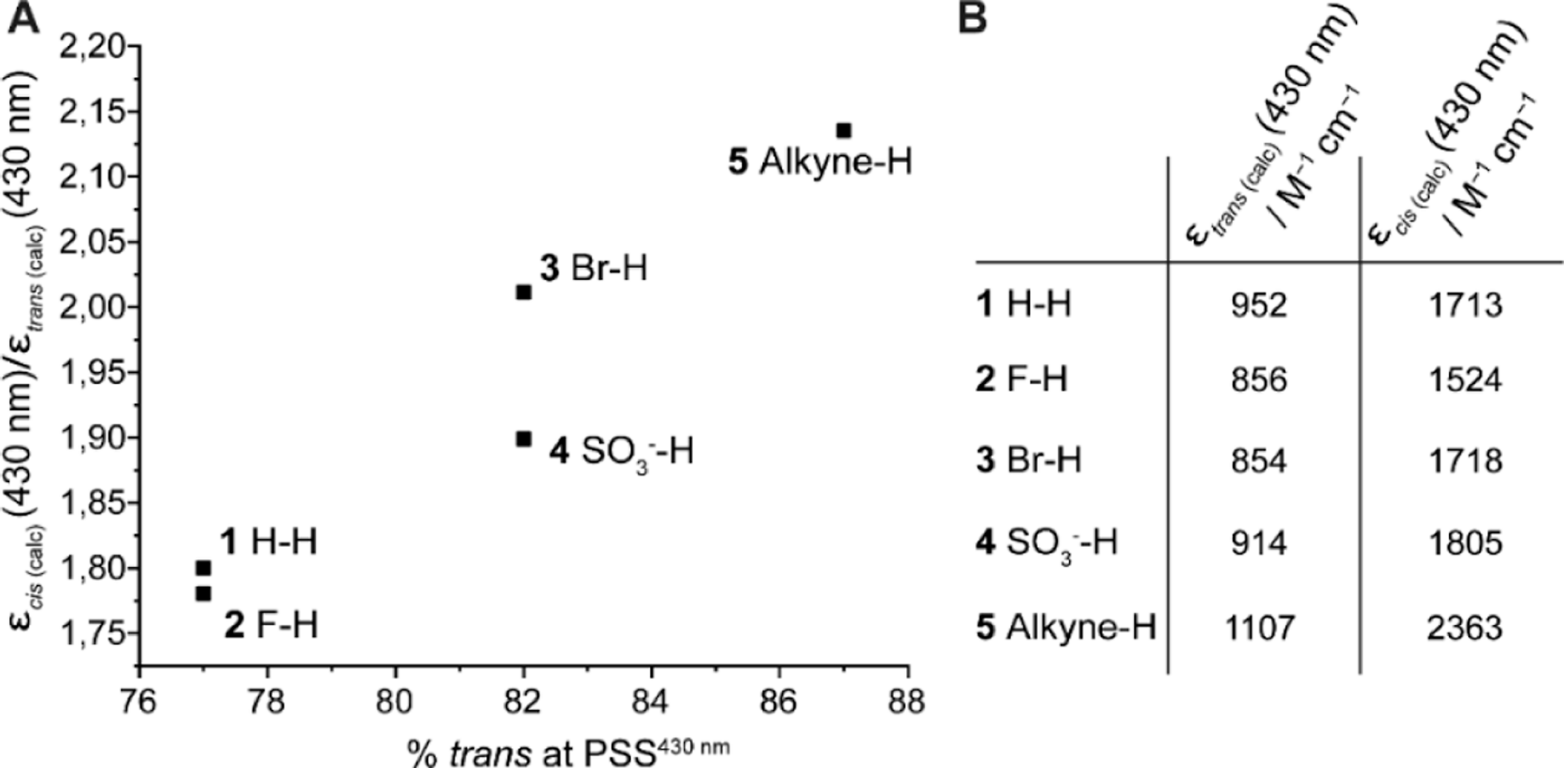
Effect of a single *para*-substituent on the molar extinction coefficient (ε) of the tetra-*ortho*-fluoro azobenzene in DMSO at a 50 μM concentration at 20 °C. (A) Graph depicting the correlation of the *cis*/*trans* ε ratio *vs* the percentage of *trans* isomer formed at PSS_430nm_. (B) Calculated values of ε at 430 nm for the pure trans and pure cis isomers.

Encouraged by the observed high PSS ratios, we wanted to verify if they indeed correlate to the n−π* band separation due to the presence of the tetra-*ortho*-fluoro groups. To this end, we studied the relation between the ratio of the molar extinction coefficients (ε) of both isomers (*cis*/*trans*) at 430 nm, where the irradiation is carried out, with the respective PSS ratio. The ε values of pure *trans* and pure *cis* were calculated from the PSDs determined by ^19^F NMR and the respective PSS UV–vis spectra (irradiation with 430 and 530 nm) for compounds **1–5**. The data (Figure 3) show that the better the band separation is, as represented by the higher the ε ratio of *cis*/*trans*, and indicative of the cis isomer absorbing more strongly compared to the *trans* isomer at the irradiation wavelength, the higher the percentage of *trans* will be formed upon irradiation at the same wavelength (i.e., the higher the PSS_430nm_). Thus, the observed higher PSS values at the investigated wavelength are a result of both band separation and a red shift of the whole spectrum for some compounds, such as azobenzene **5** (see S_0_–S_1_ absorption band maxima in Figure S99). Furthermore, the influence of the quantum yields of switching in both directions is not dramatic in this relation. Based on the observed photochemical properties, we concluded that the applied functionalizations (compounds **2–5**) in the *para* position did not compromise the favorable photochemical properties of the tetra-*ortho*-fluoro system.

### Building Blocks with Two Functional Moieties

**Figure 4:**
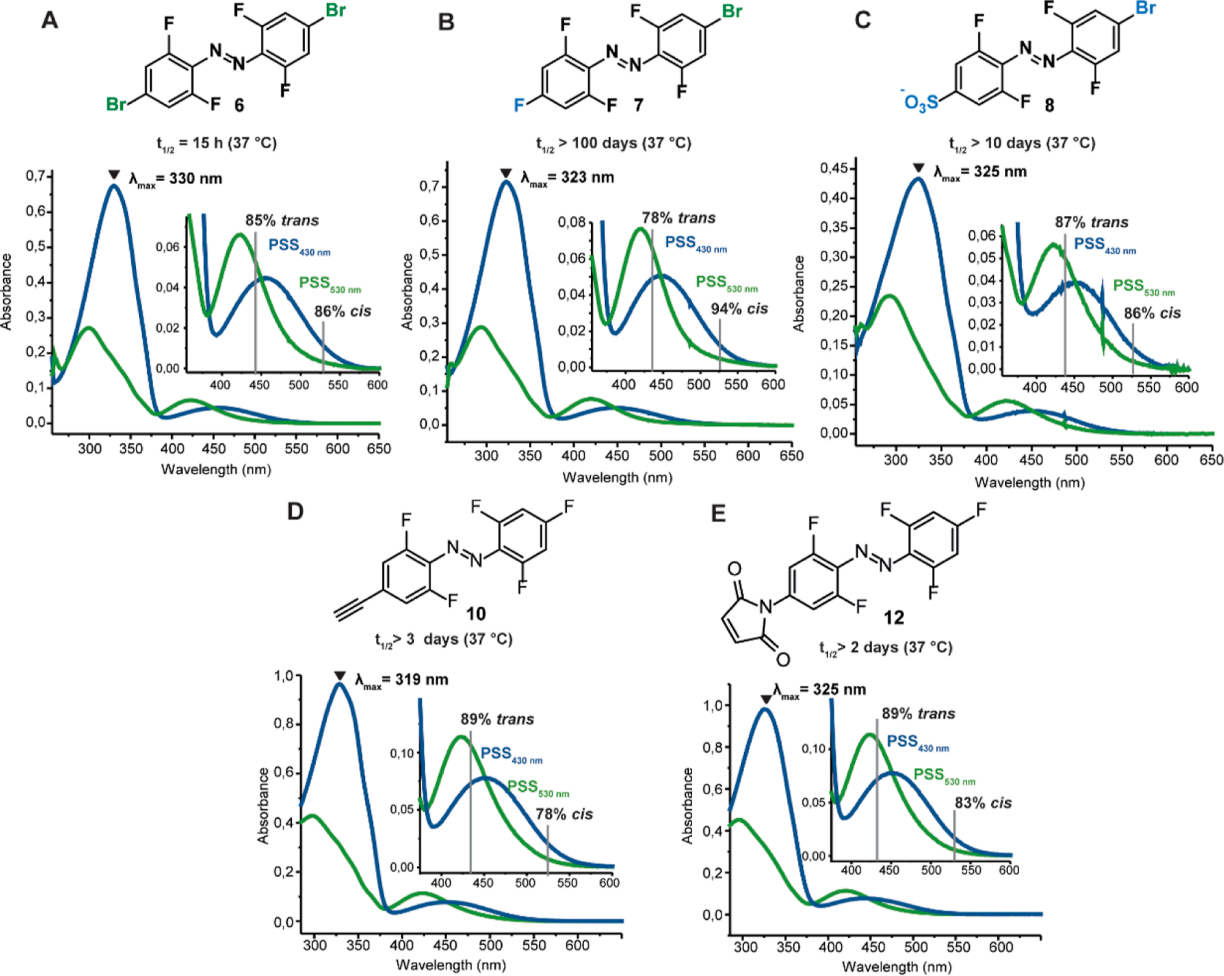
Effect of *p*,*p*-substitution of tetra-*ortho*-fluoro azobenzene with functional groups on the photochemical properties of the tetra-*ortho*-fluoro azobenzene in DMSO (A–E). All UV–vis spectra are measured at 50 μM concentration in DMSO at 25 °C. Half-lives are determined in DMSO at 37 °C. PSDs were determined via ^19^F NMR spectroscopy in DMSO-*d*_6._

The visible-light-responsive azobenzene core can be used as a building block for functional modification on both sides of the switch. Therefore, we investigated the effect of two substituents in the *para* positions of three model compounds posing as interesting targets for modification (Figure 4). Compound 6, containing two bromide groups, can serve as a building block for the synthesis of crosslinkers via cross-coupling reactions, while compound **7** with a fluoride and bromide atom can be orthogonally modified via cross-coupling and S_N_Ar to install two groups of interest (Figure 4). Azobenzene **8**, besides being furnished with a reactive handle (bromide atom), also features a sulfonate group, rendering it water-soluble. Furthermore, azobenzenes **10** and **12**, featuring an acetylene and a maleimide group, are examples of functionalizing building block **7** to carry groups used in chemical biology for covalent protein modification.

As in the previous section, the photochemical properties of compounds **6**, **7**, **8**, **10**, and **12** were studied in DMSO. All compounds featured similar absorption maxima (319–330 nm) at PSS_430nm_. While all the PSDs were above 78% for both wavelengths, compound **7** reached the highest PSS_530nm_ with 94% *cis*, and azobenzenes **10** and **12** showed 89% *trans* at PSS_430nm_. Interestingly, the half-life of the symmetrically substituted azobenzene **6** at 37 °C was significantly shorter (15 h) when compared to the mono-substituted *para*-bromo azobenzene **3** (25 d). The same trend was observed before, where in two reported examples. the symmetric bis-*para*-substituted azobenzene had a significantly shorter half-life compared to its mono-substituted counterpart.^39,42^ Particularly surprising was that compound **7**, which contains a fluoride instead of a bromide in one *para* position, exhibited the longest half-life with over 100 d under the same conditions (Figure 4). An analogous azobenzene in the literature with two fluorines in the *para* positions was reported to have a somewhat shorter half-life (95 h in ACN at 60 °C),^28^ which follows the observed phenomenon of shortened half-lives of symmetric fluorinated azobenzenes.

For the functionalized azobenzenes **10** and **12**, both introduction of an alkyne (**10**) and the nitrogen atom (**12**) in the *para* position instead of the bromine (**7**) resulted in a shorter half-life and reduction of PSD_530nm_, while PSD_430nm_ was slightly increased. Nevertheless, half-lives of **10** and **12** are still in the range of days at physiological temperature, making them highly applicable for chemical biology applications. The PSDs for previously reported fluorinated azobenzenes with two substituents in the *para* positions are within the observed range for the compounds reported here (73–95% cis and 83–97% trans),^28,29,31,36^ with the exception of a switch featuring an extended conjugated system with a 17 min half-life in an aqueous solvent with 55% cis formation.^42^

### Examples of Building Block Incorporation

**Scheme 1:**
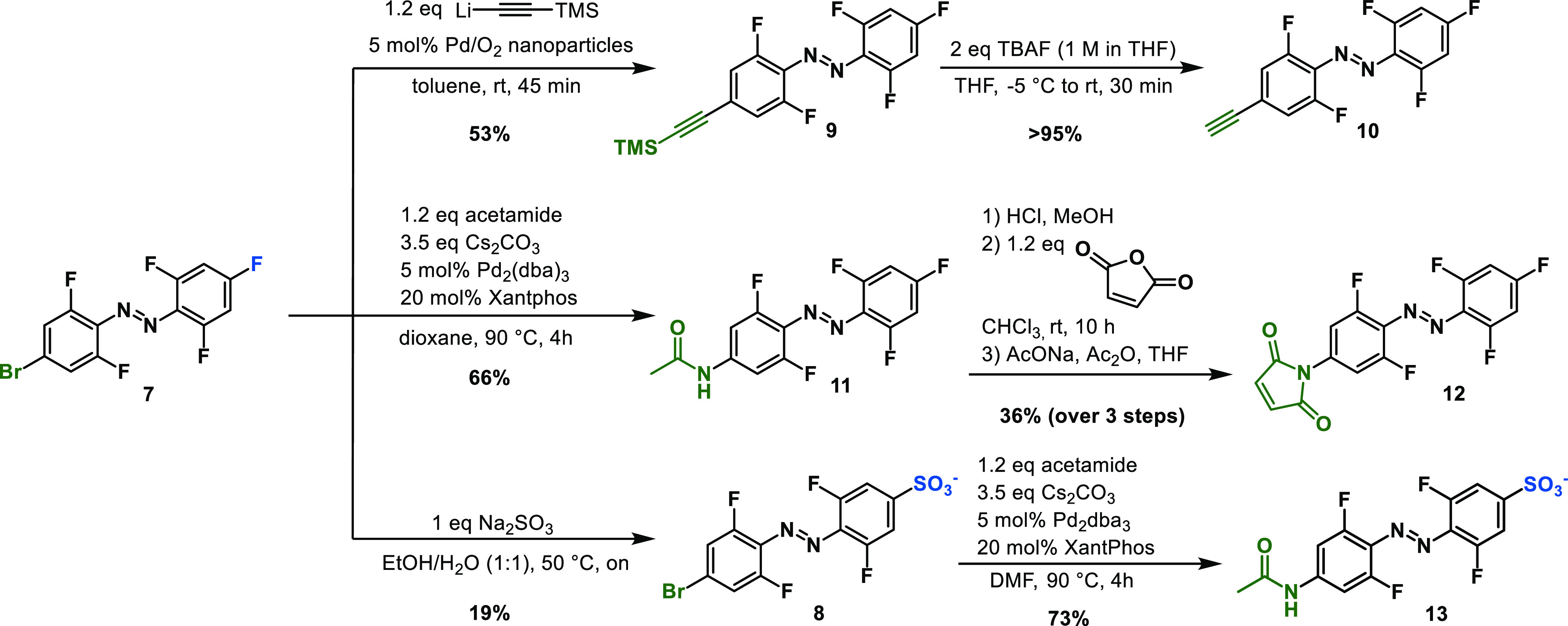
Synthesis of Four Functionalized Building Blocks (**10**, **12**, **8**, and **13**) from *p*-Fluoro-bromo Azobenzene **7**

We have evaluated the synthetic utility of the functionalized visible-light-responsive azobenzenes **3** and **7** by performing several syntheses aimed at their transformation into model products. Specifically, building block **7**, carrying a S_N_Ar-reactive fluoride atom and a bromo group for functionalization via cross-coupling reactions, was utilized to synthesize three bifunctional azobenzene compounds (Scheme 1). First, we attempted the synthesis of azobenzene **10**, which features an acetylene handle for the copper-catalyzed azide–alkyne cycloaddition (CuAAC), which is the benchmark click reaction used to incorporate moieties into, for example, biomolecules.^56^ Several standard Sonogashira reaction conditions were tested to install the acetylene group, yet the desired alkyne **10** was obtained in low yields and proved difficult to isolate from the obtained complex product mixture. In order to circumvent these problems, we used the palladium-catalyzed cross-coupling of lithium acetylides, recently developed in our group.^57^ This method provided the desired product **10** in good yield in a much cleaner fashion (Scheme 1). Interestingly, whereas in the original publication, *p*-bromofluorobenzene was not tolerated as a substrate,^57^ the heavily fluorinated azobenzene core posed no problem in the transformation. Second, we attempted the synthesis of compound **12**, which contains the maleimide moiety, that is, a useful handle for bioconjugation to proteins by means of a thio-Michael addition with cysteine.^58^ Starting again from azobenzene **7**, an acetamide was introduced via Buchwald–Hartwig amination, followed by hydrolysis and condensation with maleic anhydride, resulting in azobenzene **12** (Scheme 1).

Lastly, we synthesized the bis-*para*-substituted azobenzene molecule **8** carrying a water-solubilizing sulfonate group and a bromide for potential further functionalization via cross-coupling chemistry.^59^ To introduce the sulfonate group, we utilized the susceptibility of the highly fluorinated starting material **7** to undergo the S_N_Ar reaction. Specifically, we used the poorly nucleophilic sodium sulfite in a mixture of water and ethanol to ensure that both starting materials are at least partially dissolved during the reaction. The reaction proceeded with relatively low conversion; however, it was possible to recover the remaining pure starting material simply by extraction of the reaction mixture with DCM. The reaction was stopped at low conversion to minimize over-sulfonation of the azobenzene, mainly due to the difficult purification needed to separate the sulfonated azobenzene species via reverse-phase chromatography. Nevertheless, after obtaining pure sulfonated azobenzene **8**, we further functionalized the system by the introduction of an acetamide group via Buchwald–Hartwig coupling to make compound **9**. This new orthogonal synthetic approach clearly illustrates the advantage of S_N_Ar as an orthogonal functionalization method which allows further modification via cross-coupling to synthesize bifunctional azobenzenes and diversify available tetra-*ortho*-fluoro azobenzene designs.

**Scheme 2:**
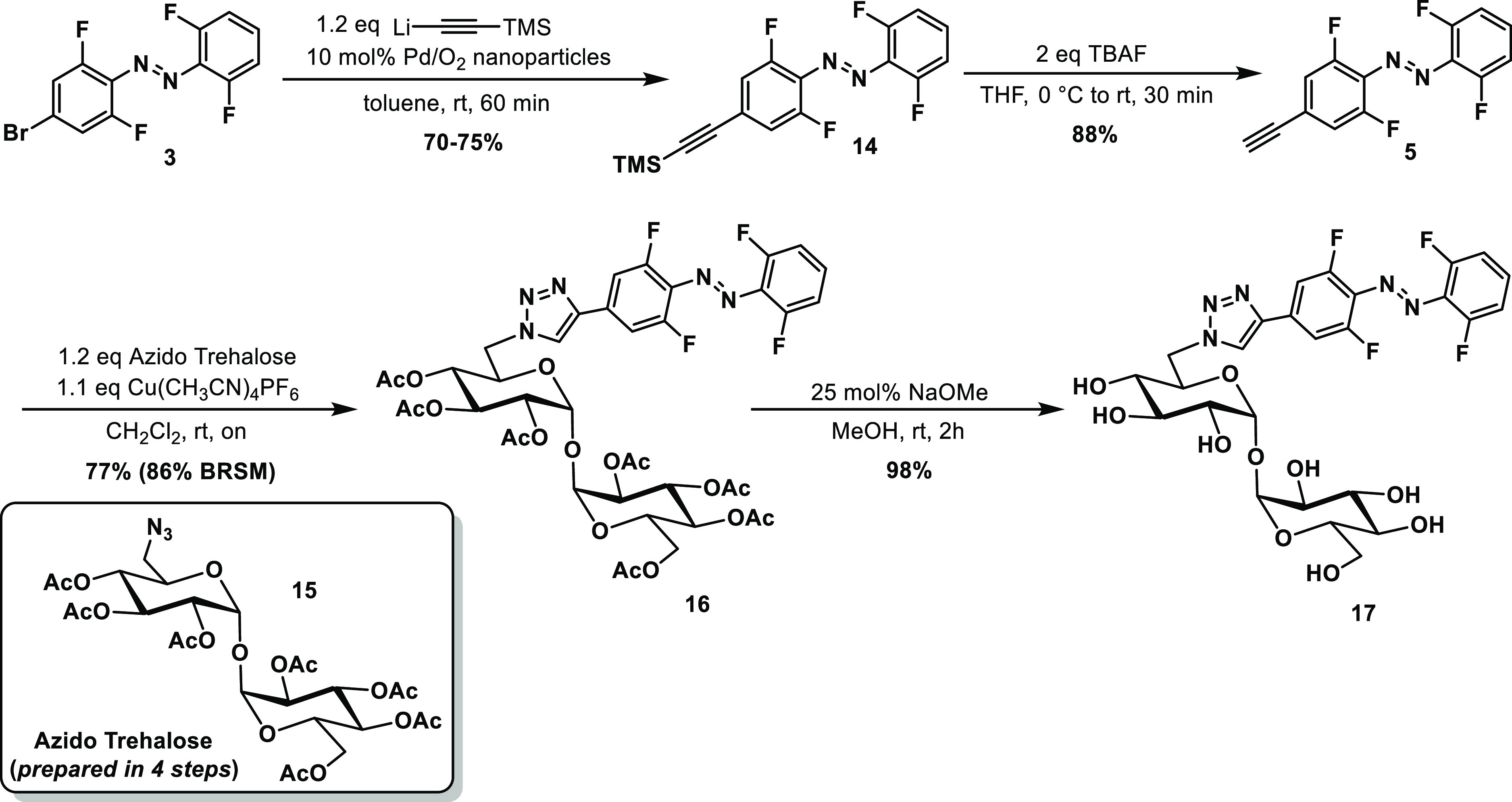
Synthesis of Azobenzene **17** Carrying a Water-Solubilizing Trehalose Moiety

Besides the charged groups, such as sulfonates in compound **8**, water solubility can also be introduced by functionalization with carbohydrates.^5,60−65^ One such molecule is α,α-trehalose, a diglucose present in many organisms such as invertebrates, bacteria, and plants.^66−69^ Having access to acetylated 6-azido-trehalose^70^ (Scheme 2), we decided to ligate it to the fluorinated azobenzene core. In short, azobenzene **3** was subjected to palladium-catalyzed cross-coupling with lithium TMS-acetylide to afford the desired product **14**. After removal of the TMS group, alkyne **5** was reacted with azido-trehalose **15** in a copper-catalyzed click reaction to yield triazole **16**. Finally, the acetyl protecting groups were removed by methanolysis to provide the target molecule **17**. As expected, the trehalose-ligated azobenzene was soluble in water, thus allowing the study of its photochemical properties in an aqueous medium.

### Photochemical Properties in Water

**Figure 5:**
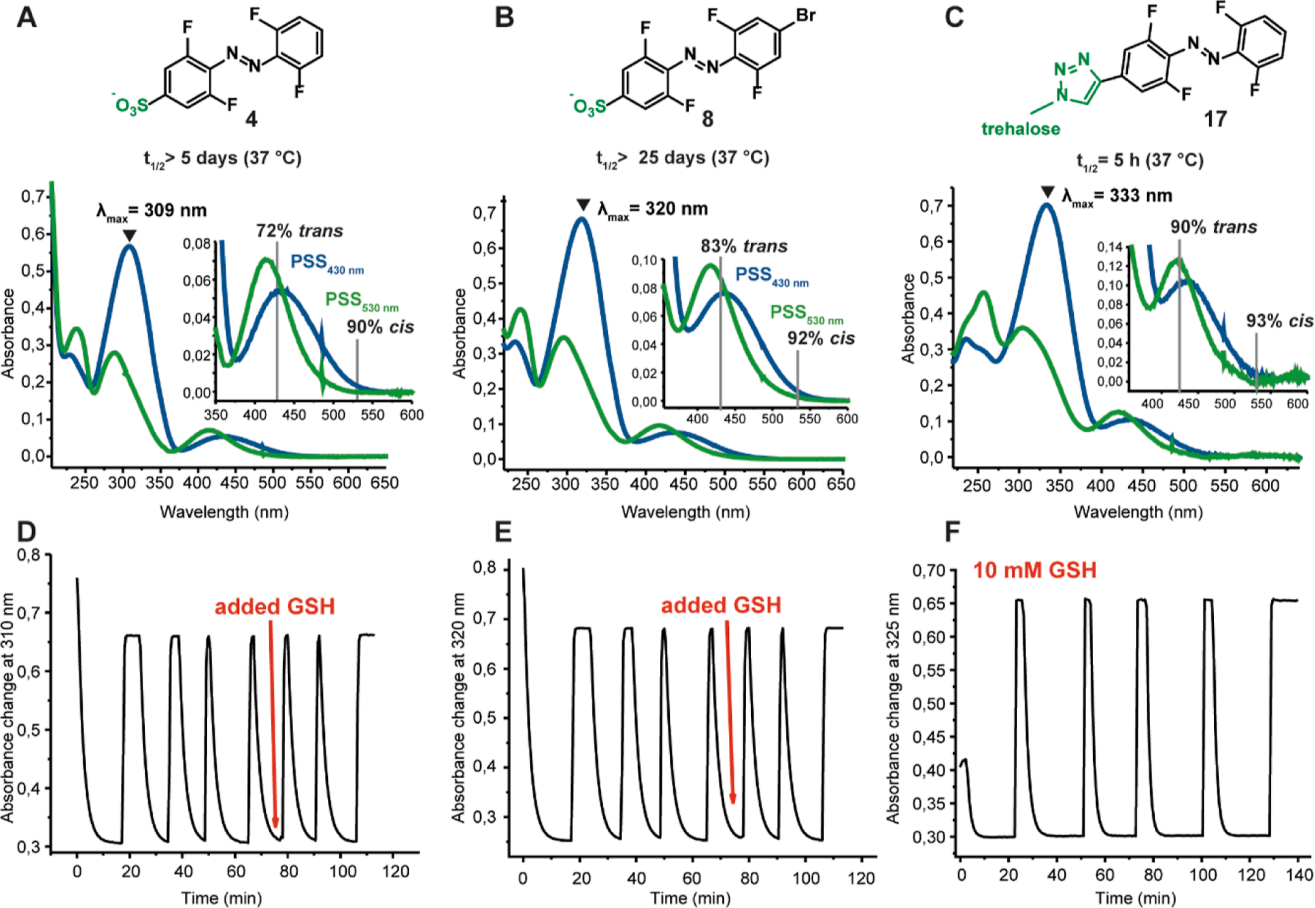
Summary of photochemical properties with respective spectra in PBS buffer of water-soluble compounds. (A–C) Determined at 20 °C at a 50 μM concentration in PBS buffer (pH = 7.5), half-lives are determined at 37 °C at 50 μM concentration in PBS buffer (pH = 7.5), PSS % determined in 2.3% DMSO-*d*_6_ in PBS buffer with 20% D_2_O at 2 mM concentration. (D–F) Fatigue resistance test upon irradiation with 530 and 430 nm light in PBS buffer at 50 μM concentration and 10 mM GSH at 20 °C.

Water solubility is crucial for most biological applications, and the photochemical properties of the photoswitch are often heavily affected by the aqueous environment.^5^ Therefore, we evaluated the photochemical properties of the three water-soluble molecules, namely, the two sulfonated azobenzenes **4** and **8**, and the trehalose containing probe **17**, in phosphate-buffered saline (PBS) buffer (Figure 5). The aforementioned compounds exhibited excellent solubility in PBS buffer; namely, stock solutions of 2–10 mM could be prepared without facing any aggregation issues. The sulfonated azobenzenes **4** and **8** both retained their properties in buffer compared to DMSO, with similar absorption maxima and slight differences in the PSD percentages, such as 90 *versus* 89 (PSS_530nm_) and 82 *versus* 72 (PSS_430nm_) for azobenzene **4** and 92% *versus* 86% (PSS_530nm_) and 83% *versus* 87% (PSS_530nm_) for azobenzene **8** (see Supporting Information sections 6.1 and 6.2).

Furthermore, it was observed that the introduction of sulfonate groups in the *para* positions did not shorten the half-life of the azobenzenes in PBS buffer since both molecules **4** and **8** exhibited very long half-lives (Figure 5). The trehalose probe **17** had higher percentages of the respective isomer formed upon irradiation at both wavelengths; however, the half-life was drastically shortened from 10 days in DMSO to 5 h in PBS buffer (Figure 5 and Figure S98). At this moment, there is no exact explanation for this trend based on the electronic properties of the three described azobenzenes. All three water-soluble compounds (**4**, **8**, and **17**) were subjected to several irradiation cycles (530 and 430 nm light) in PBS buffer and in the presence of 10 mM GSH to mimic the reducing environment within a living cell (Figure 5D–F).^45−47^ In all three examples, no degradation in the reducing GSH environment was observed, indicating the stability of the described azobenzenes for potential application *in vivo*.

### Incorporation of Azobenzene-Modified Trehalose in Mycobacteria

Probe **17** was designed to contain a trehalose group which can be metabolically incorporated into the cell wall of mycobacteria via covalent attachment to mycolic acids.^48^ Conveniently, the fluorinated core of the azobenzene allows selective detection of the switch with ^19^F NMR spectroscopy since, to the best of our knowledge, no fluorine-containing compounds are naturally present in the mycobacteria.

To assess if the trehalose-modified visible-light switch **17** can be metabolically incorporated into the mycobacterial cell wall remaining intact, we cultured *M. smegmatis* MC^2^-155 (*M. smegmatis*) until the midlog phase (optical density at 600 nm, OD_600nm_ = 0.6) and subjected the culture to 100 μM concentration of compound **17** overnight. The following day, the culture was washed to remove the unbound azobenzene probe, and the lipids were extracted using a 1:1 methanol–chloroform mixture.

**Figure 6:**
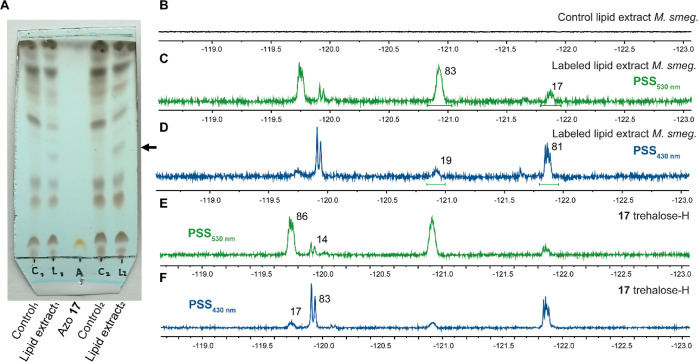
(A) TLC analysis of lipid extracts from *M. smegmatis* (MC^2^-155), control samples (C1 and C2), after labeling with azobenzene **17** (L1 and L2), and the free molecule **17** in chloroform/methanol/water = 20/4/0.5 on normal-phase silica gel TLC stained with CuSO_4_ stain (10% CuSO_4_ in an 8% H_3_PO_4_ aqueous solution). Irradiation study of the labeled lipid extract of *M. smegmatis* in DMSO-*d*_6_ (C and D) and a comparison with the free probe (E and F). (B) ^19^F NMR spectrum of the unlabeled lipid extract of *M. smegmatis.*^19^F NMR spectrum of the lipid extract of *M. smegmatis* labeled with probe **17** upon irradiation with 530 (C) and 430 nm (D) light. ^19^F NMR spectrum of the free trehalose probe **17** upon irradiation with 530 (E) and 430 nm (F) light in DMSO-*d*_6_.

First, we investigated if any new compounds were present in the mycobacterial lipid extract after labeling. Upon thin-layer chromatography (TLC) analysis, we observed a clear spot in the labeled lipid extract, which is not present in the control sample and was not the parent azobenzene **17**, possibly indicating incorporation of the photoswitch in the mycobacterial cell wall (Figure 6A). Subsequently, the isolated mycobacterial lipid extract was analyzed using ^19^F NMR spectroscopy (Figure 6). As expected, there were no signals in the ^19^F NMR spectrum of the lipid fraction of unlabeled *M. smegmatis* (control; Figure 6B), whereas the labeled bacterial lipid extract did show signals at chemical shifts matching that of the parent azobenzene **17** (Figure 6C–F). Next, the labeled lipid extract was irradiated with λ = 530 nm light until PSS was reached. The ^19^F NMR spectrum showed four signals belonging to the *cis* and *trans* isomers of the probe with the same chemical shifts as the free probe (Figure 6C,E). The sample was further irradiated with a 430 nm light to switch back to predominantly the *trans* isomer (Figure 6d,F). The PSD values of the labeled lipid extract were near identical to the free trehalose probe **17**, indicating photoswitching of the azobenzene within the lipid extract while retaining its photophysical properties. However, upon irradiation, the formation of a new set of two upfield signals was observed (Figure S103), and as the irradiation cycles continued, the intensity of these signals was increasing. The emergence of the new signals can possibly be caused by the degradation of the azobenzene due to reduction or oxidation, S_N_Ar of the *ortho* fluorides, or the formation of a new interaction with the lipid environment. This side-product formation is a limitation for the application of molecule **17**, and further investigation is necessary to provide a more stable design.

These results suggest that azobenzene **17** was indeed metabolically processed by the mycobacterium and incorporated into its cell wall. Gratifyingly, the observed fluorine signals in the ^19^F NMR spectra confirm that the trehalose–azobenzene molecule remained stable during culturing, the metabolic process, and the isolation of the lipid extract. Furthermore, the photoswitching studies on the labeled lipid extract show that switch **17** remained functional after the uptake experiment. The observation of a new species on the TLC sample indicates covalent attachment to mycolic acids to form an azobenzene-modified trehalose monomycolate.

## Conclusions

**Figure 7:**
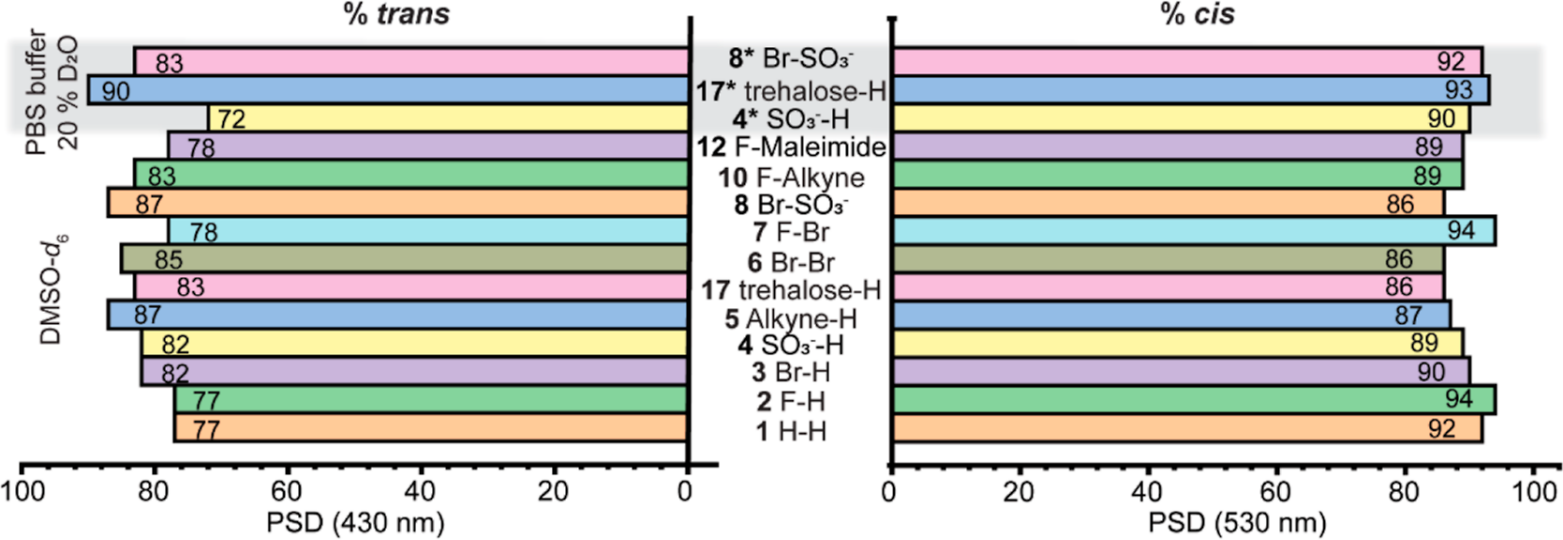
Bar graph depicting the measured PSD % for all compounds in DMSO-*d*_6_ and 20% D_2_O in PBS buffer (gray area). On the left, the percentage of the formed *trans* isomer upon irradiation with 430 nm. On the right, the percentage of *cis* isomer formed upon irradiation with 530 nm light. All percentages were determined based on the integration of relative signals in ^19^F NMR spectra.

Here, we present the photochemical properties of nine functional tetra-*ortho*-fluoro azobenzenes, of which four were highly water-soluble and therefore were studied in PBS buffer. All azobenzenes were fully operational in the tested media and exhibited high PSD upon both green (530 nm) and blue (430 nm) light irradiations. The azobenzenes with a fluorine in the *para* position, **2** and **7**, had the highest PSD_530nm_ (94%), yet the same compounds had lower PSD_430nm_ (78%) (Figure 7). Interestingly, in the aqueous medium, the sulfonated compounds **4** and **8** had high PSDs for both wavelengths except for PSD_430nm_ (**4**) with the lowest measured value of 72%. The highest PSD for both wavelengths was observed for the trehalose probe **17** in buffer (PSD_430nm_ 90% and PSD_530nm_ = 93%, Figure 7). It is important to note that the PSDs can further be increased as they most likely do not represent the highest possible values since the irradiation wavelengths can be optimized for each individual azobenzene. While most azobenzene building blocks exhibited very long half-lives, in the range of days, both in DMSO and in PBS buffer even at 37 °C, two exceptions were observed. Compound **6** with two bromides in the *para* positions noted a shortened half-life of 15 h in DMSO. Furthermore, while the trehalose probe **17** had a long half-life (>10 d) in DMSO, this value was significantly shortened in an aqueous medium to 5 h at 37 °C. Nevertheless, this phenomenon is often observed upon solubilizing photoswitches in water.^5,71^

We have subjected model building blocks **3** and **7** to several useful functionalization reactions, such as a palladium-catalyzed organolithium cross-coupling, Buchwald–Hartwig amination, copper-catalyzed click reaction, and S_N_Ar reaction to introduce a water-solubilizing group. Thus, we demonstrated the compatibility of the tetra-*ortho*-fluoro azobenzene scaffold with functionalization strategies.

Finally, the trehalose-modified azobenzene **17** was metabolically processed by *M. smegmatis* and we observed photoswitching within the mycobacterial lipid extract, exhibiting very similar PSD values to the free molecule **17** in DMSO, yet with the formation of new signals in the ^19^F NMR upon several irradiation cycles of the lipid extract.

In conclusion, we presented a selection of visible-light-controlled azobenzene building blocks with the tetra-*ortho*-fluorinated core carrying useful groups for biological applications. After confirming that the photochemical properties of water-soluble building blocks remain excellent even in the aqueous environment, we also demonstrated that a trehalose-decorated tetra-fluoro-azobenzene was still photoswitchable upon metabolic processing by a mycobacterium. Having demonstrated the trehalose-decorated azobenzene photoswitching within the mycobacterial lipid extract, the next steps would involve incorporating a more stable system into the mycobacterial cell wall and irradiation of living cells. Photoswitching within the membrane could significantly impact its turbidity and integrity, which can be of great use in delivering antibiotics to tuberculosis mycobacteria to more efficiently combat this difficult-to-treat disease. All in all, the favorable features of the tetra-*ortho*-fluoro azobenzene system bring the described switches closer to applications within living systems.

## Methods

### General Information

All chemicals for synthesis were obtained from commercial sources and used as received unless stated otherwise. Technical-grade solvents were used for extraction and chromatography. TLC was performed using commercial Kieselgel 60 F254 silica gel plates with fluorescence-indicator UV254 (Merck, TLC silica gel 60 F254). For the detection of components, UV light at λ = 254 nm or λ = 365 nm was used. Alternatively, oxidative staining was performed using a basic solution of potassium permanganate in water or aqueous cerium phosphomolybdic acid solution (Seebach’s stain). Merck silica gel 60 (230–400 mesh, ASTM) was used in normal-phase flash chromatography. A Büchi Reveleris X2 automatic column was used with Büchi EcoFlex silica columns (4–40 g, 40–63 μM, 60 Å).

### Analysis

Spectroscopic measurements were made in Uvasol-grade solvents using a quartz cuvette (path length 10.0 mm). UV–vis measurements were performed on an Agilent 8453 UV–visible absorption spectrophotometer. UV–vis irradiation experiments were carried out using a custom-built (Prizmatix/Mountain Photonics) multiwavelength fiber-coupled light-emitting diode (LED) system (FC6-LED-WL) with LED lights (425A and 530B) and 530 and 430 nm LED light sources (3 × 530 nm, 3 × 420 nm, LED Nichia NCSB219B-V1, Sahlmann Photochemical Solutions). The temperature was controlled with a Quantum Northwest TC1 temperature controller. The data was processed using Agilent UV–vis ChemStation B.02.01 SP1, Spectragryph 1.2, OriginPro 2016, and all images were assembled in Adobe Illustrator. NMR spectra were obtained using Agilent Technologies 400 MR (400/54 Premium Shielded) (^1^H: 400 MHz, ^13^C: 101 MHz, ^19^F: 61 MHz) and Bruker Innova (^1^H: 600 MHz, ^13^C: 151 MHz) spectrometers at room temperature (rt) (22–24 °C). Chemical shift values (δ) are reported in parts per million (ppm) with the solvent resonance as the internal standard (CDCl_3_: δ 7.26 for ^1^H, δ 77.16 for ^13^C; DMSO: δ 2.50 for ^1^H, δ 39.52 for ^13^C; CD_3_OD: δ 3.31 for ^1^H, δ 49.0 for ^13^C; CD_3_CN: δ 1.95 for ^1^H, δ 1.32 and δ 118.26 for ^13^C; D_2_O: δ 4.79 for ^1^H). The following abbreviations are used to indicate signal multiplicity: s (singlet), d (doublet), t (triplet), q (quartet), m (multiplet), brs (broad signal), or dd (double doublet). Structural assignments were made with additional information from gCOSY, gHSQC, and gHMBC experiments. Exact mass spectra were recorded on an LTQ Orbitrap XL (ESI^+^, ESI^–^, and APCI). All reactions requiring an inert atmosphere were carried out under a nitrogen atmosphere using oven-dried glassware and standard Schlenk techniques. Dichloromethane and toluene were used from a solvent purification system using an MBraun SPS-800 column. Melting points were determined using a Stuart analogue capillary melting point SMP11 apparatus. All errors are given as standard deviations.

#### 1,3-Difluoro-2-nitrosobenzene (**19**)

Compound **19** was synthesized according to the literature procedures.^72^ Compound **19** was isolated as a light-green solid (7.7 g, yield >95%).^1^H NMR (400 MHz, CDCl_3_): δ 7.68–7.58 (m, 1H), 7.12 (t, *J* = 8.6 Hz, 2H). ^19^F NMR (376 MHz, CDCl_3_): δ −130.24. FTIR (ATR): 1607 s (C=C), 1474 s (N=O), 1243 s (C–N), 1014 s (C–N), 416 m (C–F). mp 109–111 °C.

#### Trans-1,2-bis(2,6-difluorophenyl)diazene (**1**)

Compound **1** was synthesized according to the literature procedures.^24,73^ Compound **1** was isolated as deep-red needle crystals (10.8 g, yield = 79%). ^1^H NMR (400 MHz, CDCl_3_): δ 7.43–7.33 (m, 1H), 7.07 (m, 2H). ^19^F NMR (376 MHz, CDCl_3_): δ −121.25 (dd, *J* = 9.0, 5.8 Hz). ^1^H NMR (400 MHz, DMSO-*d*_6_): δ 7.69–7.60 (m, 1H), 7.37 (t, *J* = 8.4 Hz, 2H). ^19^F NMR (376 MHz, DMSO-*d*_6_): δ −122.02. HRMS (ESI^+^) *m*/*z*: [M + H]^+^ calcd for C_12_H_6_F_4_N_2_ + H, 255.0539; found, 255.0539. mp 75–79 °C.

#### (Trans)-1-(2,6-difluorophenyl)-2-(2,4,6-trifluorophenyl)diazenediazene (**2**)

Nitroso compound **19** (4.8 g, 34 mmol) was dissolved in 53 mL of the solvent mixture (toluene/AcOH/TFA = 35/35/5) and 2,4,6-trifluoroaniline **20** (1 equiv, 4.9 g, 34 mmol) was added to the reaction mixture, which was stirred at rt for 2 d. The solvent was removed by rotary evaporation from the reddish-brown solution with a base (aq. NaHCO_3_ solution) in the rotary evaporator collection flask. The crude product was purified via column chromatography on silica with an eluent mixture (pentane/DCM = 7/3). The pure product was isolated by recrystallization from hot methanol and obtained as red needle crystals (5.6 g, yield = 61%). ^1^H NMR (400 MHz, DMSO-*d*_6_): δ 7.58 (tt, *J* = 8.5, 6.1 Hz, 1H), 7.41 (t, *J* = 9.6 Hz, 2H), 7.30 (t, *J* = 9.0 Hz, 2H). ^19^F NMR (376 MHz, DMSO-*d*_6_): δ −102.22 (p, *J* = 8.9 Hz, 1F), −117.33 (t, *J* = 9.3 Hz, 2F), −121.81 (dd, *J* = 10.4, 6.1 Hz, 2F). ^13^C{^1^H} NMR (101 MHz, DMSO-*d*_6_): δ 162.9 (dt, *J* = 253.3, 15.8 Hz), 157.0 (dd, *J* = 16.1, 6.6 Hz), 154.7 (dd, *J* = 259.4, 4.1 Hz), 133.0 (t, *J* = 10.7 Hz), 130.6 (t, *J* = 9.9 Hz), 128.1 (d, *J* = 5.2 Hz), 113.1 (dd, *J* = 20.0, 3.5 Hz), 102.2 (ddd, *J* = 26.9, 24.7, 3.9 Hz). HRMS (APCI^+^) *m*/*z*: [M + H]^+^ calcd for C_12_H_6_F_5_N_2_ + H, 273.0446; found, 273.0447. mp. 77–78 °C.

#### Trans-1-(4-bromo-2,6-difluorophenyl)-2-(2,6-difluorophenyl)diazene (**3**)

Compound **3** was synthesized according to the literature procedures.^74,75^ Compound **3** was isolated as a deep-red crystalline solid (4.1 g, yield = 76%). ^1^H NMR (400 MHz, CDCl_3_): δ 7.43–7.34 (m, 1H), 7.26 (d, *J* = 8.0 Hz, 2H), 7.06 (t, *J* = 8.7 Hz, 2H). ^19^F NMR (376 MHz, CDCl_3_): δ −119.04 (d, *J* = 9.1 Hz), −120.72 (q, *J* = 6.3, 5.7, 2.8 Hz). ^1^H NMR (400 MHz, DMSO-*d*_6_): δ 7.77 (d, *J* = 9.4 Hz, 2H), 7.64 (ddd, *J* = 14.5, 8.6, 6.3 Hz, 1H), 7.35 (t, *J* = 9.6 Hz, 2H). ^19^F NMR (376 MHz, DMSO-*d*_6_): δ −119.85 (d, *J* = 12.2 Hz), −121.50 (dd, *J* = 10.2, 5.9 Hz). HRMS (APCI^+^) *m*/*z*: [M + H]^+^ calcd for C_12_H_6_BrF_4_N_2_ + H, 332.9645; found, 332.9650. mp 60–63 °C.

#### (Trans)-4-((2,6-difluorophenyl)diazenyl)-3,5-difluorobenzenesulfonate (**4**)

Compound **2** (2 g, 7.3 mmol) and sodium sulfite (1 equiv, 930 mg, 7.3 mmol) were weighed into a round-bottom flask. The solids were dissolved in a water: ethanol mixture (1:1, 80 mL), and the solution was purged from oxygen by bubbling nitrogen gas through the solution for 5 min. The reaction mixture was left to stir at 50 °C (metal heating mantle) overnight. The remaining starting material was washed away with DCM (3 × 50 mL), while the aqueous layer, containing the sulfonated product, was collected and freeze-dried. The crude orange solid was subsequently purified by GRACE automatic chromatography or a reversed-phase C18 silica gel with a gradient of 1 mM NH_4_HCO_3_ buffer and acetonitrile as eluents. The pure fractions were verified by LC–MS (0.01% ammonia in water and acetonitrile as eluents, negative mode) and freeze-dried to obtain the relatively pure product as a light-orange powder (490 mg, 19% yield). ^1^H NMR (400 MHz, CD_3_CN, drop D_2_O): δ 7.54–7.49 (m, 3H), 7.17 (ddd, *J* = 10.1, 8.5, 1.6 Hz, 2H). ^19^F NMR (376 MHz, CD_3_CN, drop D_2_O): δ −120.92 (d, *J* = 9.1 Hz), −122.53 (dd, *J* = 10.5, 6.1 Hz). ^13^C{^1^H} NMR (101 MHz, CD_3_CN, drop D_2_O): δ 157.3 (dd, *J* = 65.4, 4.0 Hz), 154.7 (dd, *J* = 67.4, 3.9 Hz), 149.8 (t, *J* = 8.4 Hz), 134.2 (t, *J* = 10.8 Hz), 133.8 (t, *J* = 10.6 Hz), 132.4 (t, *J* = 10.0 Hz), 114.0 (dd, *J* = 20.5, 3.6 Hz), 111.6 (dd, *J* = 23.0, 3.2 Hz). HRMS (ESI−) *m*/*z*: [M]^−^ calcd for C_12_H_5_O_3_F_4_N_2_S^–^, 332.9963; found, 332.9965. mp >250 °C. *The issue of aggregate formation at higher concentrations limited the amount of compound used for NMR spectroscopy. Due to C–F coupling, the presence of trans/cis mixture, and the low concentration making the 1D spectra complex for analysis, 2D NMR spectra were added for characterization.

#### (Trans)-1-(2,6-difluoro-4-((trimethylsilyl)ethynyl)phenyl)-2-(2,6-difluorophenyl)diazene (**14**)

The reported synthetic procedure was adapted from the literature.^57^ Pd[P(*t*Bu)_3_]_2_/O_2_ was prepared by purging a solution of Pd[P(*t*Bu)_3_]_2_ in toluene (10 mg/mL) with oxygen (3 × 20 mL for 200 mL of the catalyst solution), resulting in a rapid color transition from orange to dark-reddish brown. The reaction mixture was left to stir for 16 h. In a dry Schlenk flask under an inert atmosphere, the freshly prepared Pd[P(*t*Bu)_3_]_2_/O_2_ solution (5.2 mL, 10 mg/mL, 10 mol %) and the aryl bromide **3** (330 mg, 1.0 mmol) were dissolved in dry toluene (5.4 mL) and left to stir at rt for 5 min. Meanwhile, a solution of TMS-acetylene (240 mg, 340 μL, 2.4 mmol, 2.4 equiv) in dry THF (3.3 mL) was stirred at 0 °C (iced water bath), and *n*BuLi (1.5 mL, 1.6 M in hexanes; 2.4 mmol, 2.4 equiv) was slowly added, resulting in a 0.5 M solution, after which the mixture was allowed to warm to rt. The freshly prepared lithium acetylide (124 mg, 2.4 mL, 0.5 M in THF, 1.2 mmol, 1.2 equiv) was subsequently added to the reaction mixture over 60 min using a syringe pump and quenched by the addition of *i*PrOH (2 mL). The reaction mixture was washed with H_2_O (2 × 20 mL), and the organic phase was dried over Na_2_SO_4_, filtered, and concentrated under reduced pressure to afford a brown oil. The residue was purified by GRACE automated flash column chromatography on a silica gel with a gradient of DCM and pentane as eluents. A dark-orange oil was isolated (260 mg, 75% yield) as a mixture of *E*/*Z* isomers with a ratio of 29:71 based on the integration of the CH_3_ signal from the TMS moiety in ^1^H NMR.

^1^H NMR (400 MHz, CD_3_CN, trans): δ 7.54 (tt, *J* = 8.5, 6.0 Hz, 1H), 7.25 (d, *J* = 9.4 Hz, 2H), 7.19 (dd, *J* = 9.5, 8.5 Hz, 2H), 0.27 (s, 9H). ^1^H NMR (400 MHz, CD_3_CN, cis): δ 7.37 (tt, *J* = 8.6, 6.3 Hz, 1H), 7.10–6.96 (m, 4H), 0.22 (s, 9H). ^19^F NMR (376 MHz, CD_3_CN): δ −121.33 (dt, *J* = 8.7, 5.6 Hz, cis), −121.85––121.98 (m, cis), −122.45 (d, *J* = 9.9 Hz, trans), −122.72 (dd, *J* = 9.9, 6.0 Hz, trans). ^13^C{^1^H} NMR (101 MHz, CD_3_CN, trans): δ 156.3 (ddd, *J* = 260.1, 28.8, 4.7 Hz), 152.4 (ddd, *J* = 251.9, 21.1, 5.8 Hz), 133.8 (t*, J* = 10.7 Hz), 132.1 (t, *J* = 9.8 Hz), 127.6 (t, *J* = 12.3 Hz), 126.1 (t, *J* = 11.4 Hz), 117.1 (d, *J* = 25.9 Hz), 113.9 (d, *J* = 24.1 Hz), 102.7, 100.7, −0.3. HRMS (APCI^+^) *m*/*z*: [M + H]^+^ calcd for C_17_H_14_F_4_N_2_Si + H^–^, 351.0935; found, 351.0938.

#### (Trans)-1-(2,6-difluorophenyl)-2-(4-ethynyl-2,6-difluorophenyl)diazene (**5**)

A solution of compound **14** (240 mg, 0.68 mmol) in dry THF (20 mL) was cooled to below 0 °C with an ice salt bath followed by the addition of tetra-n-butylammonium fluoride (TBAF, 2 equiv, 1.4 mL, 1 M in THF, 1.4 mmol). The orange solution turned dark upon the addition of the TBAF solution. The ice bath was removed, and the reaction mixture was allowed to warm up to rt and was stirred for an additional 30 min. The reaction mixture was diluted with diethyl ether (100 mL) and extracted with brine (3 × 50 mL). The combined organic phases were dried using Na_2_SO_4_, filtered, and concentrated under reduced pressure to afford a brown solid which was further purified via GRACE automatic chromatography on a silica gel with a gradient of pentane and DCM as solvents. The pure product was isolated as a dark-orange solid (167 mg, 88% yield) ^1^H NMR (400 MHz, CD_3_CN): δ 7.54 (tt, *J* = 8.6, 6.0 Hz, 1H), 7.35–7.29 (m, 2H), 7.24–7.16 (m, 2H), 3.72 (s, 1H). ^19^F NMR (376 MHz, CD_3_CN): δ −122.57 (dd, *J* = 9.6, 3.3 Hz), −122.60––122.68 (m). ^13^C{^1^H} NMR (101 MHz, CD_3_CN): δ 157.5 (d, *J* = 4.0 Hz), 157.2 (d, *J* = 5.4 Hz), 155.0 (d, *J* = 4.1 Hz), 154.6 (d, *J* = 5.3 Hz), 133.7 (t, *J* = 10.8 Hz), 132.4 (t, *J* = 10.0 Hz), 131.9 (t, *J* = 9.9, 8.6 Hz), 126.5 (t, *J* = 12.5 Hz), 117.4–117.1 (m), 113.7 (dd, *J* = 20.4, 3.6 Hz), 83.2, 81.3 (t, *J* = 3.5 Hz). HRMS (APCI^+^) *m*/*z*: [M + H]^+^ calcd for C_14_H_6_F_4_N_2_ + H, 279.0540; found, 279.0540. mp 73–74 °C.

#### 5-Bromo-1,3-difluoro-2-nitrosobenzene (**22**)

Compound **22** was synthesized according to the literature procedure.^76^ Compound **22** was isolated as a pale-green solid (6.8 g, yield = 91%). ^1^H NMR (400 MHz, CDCl_3_): δ 7.38–7.30 (m, 2H). ^19^F NMR (376 MHz, CDCl_3_): δ −128.66 (d, *J* = 8.2 Hz). FTIR (ATR): 1601s (C=C), 1431s (N–O), 1275s (C–N), 1060s (C–N), 542 m (C–F). mp 87–90 °C.

#### Trans-1,2-bis(4-bromo-2,6-difluorophenyl)diazene (**6**)

Compound **6** was synthesized according to the literature procedures.^77,78^^1^H NMR (400 MHz, CHCl_3_): δ 7.27 (d, *J* = 8.1 Hz, 1H). ^19^F NMR (376 MHz, CHCl_3_): δ −118.63 (d, *J* = 8.5 Hz). ^1^H NMR (400 MHz, DMSO-*d*_6_): δ 7.85–7.67 (m, 1H).·^19^F NMR (376 MHz, DMSO-*d*_6_): δ −119.37 (d, *J* = 9.5 Hz). HRMS (ESI^+^) *m*/*z*: [M + H]^+^ calcd for C_12_H_4_Br_2_F_4_N_2_ + H, 410.8750; found, 410.8742. mp. 164–168 °C.

#### (Trans)-1-(4-bromo-2,6-difluorophenyl)-2-(2,4,6-trifluorophenyl)diazene (**7**)

Nitroso compound **22** (6.0 g, 27 mmol) was dissolved in 100 mL of the solvent mixture (toluene/AcOH/TFA, 40:40:6, v/v) and 2,4,6-trifluoroaniline **20** (1.0 equiv, 3.9 g, 27 mmol) was added. The reaction mixture was stirred at rt for 2 d. The solvent was removed by rotary evaporation from the reddish-brown solution with a base (aqueous NaHCO_3_ solution) in the rotary evaporator collection flask. The crude product was purified via column chromatography on silica with pentane as the eluent. The product was isolated as a red solid (7.9 g, yield = 83%). ^1^H NMR (400 MHz, CDCl_3_): δ 7.28–7.23 (m, 2H), 6.88–6.80 (t, *J* = 8.8 Hz, 2H); ^19^F NMR (376 MHz, CDCl_3_): δ −102.04 (p, *J* = 8.5 Hz), −115.95 (t, *J* = 8.8 Hz), −118.63 (d, *J* = 8.4 Hz), −119.03 (d, *J* = 8.4 Hz); ^13^C{^1^H} NMR (101 MHz, CDCl_3_): δ 165.1 (t, *J* = 15.1 Hz), 162.6 (td, *J* = 15.2, 14.9, 13.7 Hz), 158.5–158.1 (m), 157.2 (d, *J* = 5.1 Hz), 155.7 (dd, *J* = 15.2, 6.2 Hz), 154.6 (d, *J* = 5.9 Hz), 131.2, 124.5 (t, *J* = 11.9 Hz), 117.0 (d, *J* = 27.4 Hz), 102.4–101.7 (m). ^1^H NMR (400 MHz, DMSO-*d*_6_): δ 7.77 (d, *J* = 9.6 Hz, 1H), 7.51 (t, *J* = 9.7 Hz, 1H); ^19^F NMR (376 MHz, DMSO-*d*_6_): δ −101.40, −116.88, −119.34, −119.74; ^13^C{^1^H} NMR (101 MHz, DMSO-*d*_6_): δ 164.5 (t, *J* = 15.7 Hz), 161.9 (t, *J* = 15.9 Hz), 157.1 (dd, *J* = 16.0, 6.5 Hz), 156.0 (0 5.1 Hz), 154.5 (dd, *J* = 16.1, 6.5 Hz), 153.4 (d, *J* = 5.0 Hz), 129.8 (t, *J* = 9.7 Hz), 124.4 (t, *J* = 12.4 Hz), 117.1 (d, *J* = 23.7 Hz), 102.7–102.0 (m). HRMS (ESI^+^) *m*/*z*: [M + H]^+^ calcd for C_12_H_4_BrF_5_N_2_ + H, 350.9551; found, 350.9554. mp: 82–95 °C.

#### (Trans)-4-((4-bromo-2,6-difluorophenyl)diazenyl)-3,5-difluorobenzenesulfonate (**8**)^59^

Azobenzene **7** (2.0 g, 5.7 mmol) and sodium sulfite (1.0 equiv, 0.72 g, 5.7 mmol) were weighed in a round-bottom flask and were subjected to three vacuum-dry nitrogen cycles. The solids were dissolved in 150 mL of the solvent mixture (water/EtOH, 1:1, v/v), and the solvent was degassed by bubbling dry N_2_ for 10 min. The reaction mixture was heated to 50 °C using a metal heating mantle and stirred vigorously overnight. The solvent was partially removed *in vacuo*, and the resulting mixture was extracted with DCM (3 × 100 mL) to remove the remaining starting material, which is not soluble in water. The aqueous layer was loaded on Celite and freeze-dried to remove water. The crude product was purified by automatic chromatography on a reversed-phase silica gel (C18) with a gradient of the solvent mixture (0–100% 10 mM NH_4_HCO_3_ (aq) in ACN). The obtained fractions were freeze-dried, and pure product **8** was isolated as a yellow solid (440 mg, 19% yield).

^1^H NMR (600 MHz, CD_3_CN, drop D_2_O): δ 7.57–7.49 (m, 1H), 7.45 (d, *J* = 8.7 Hz, 1H); ^19^F NMR (565 MHz, CD_3_CN): δ −120.46 (dd, *J* = 29.7, 10.2 Hz); ^13^C{^1^H} NMR (151 MHz, CD_3_CN): δ 156.2, 155.7, 154.5, 154.0, 149.4, 131.9, 130.3, 124.7, 117.1, 117.0, 110.8; ^13^C{^1^H} NMR (151 MHz, CD_3_CN, drop D_2_O): δ 156.2, 155.7, 154.5, 154.0, 149.34, 131.9, 130.4, 124.7, 117.1, 117.0, 110.8. ^1^H NMR (400 MHz, DMSO-*d*_6_): δ 8.41 (s, 1H), 7.79 (d, *J* = 9.3 Hz, 2H), 7.42 (d, *J* = 10.1 Hz, 2H); ^19^F NMR (376 MHz, DMSO-*d*_6_): δ −119.45, −119.96. HRMS (ESI^–^) *m*/*z*: [M]^−^ calcd for C_12_H_4_BrF_4_N_2_O_3_S^–^, 410.9068; found, 410.9060. mp > 250 °C.

#### (Trans)-1-(2,6-difluoro-4-((trimethylsilyl)ethynyl)phenyl)-2-(2,4,6-trifluorophenyl)diazene (**9**)

The reported synthetic procedure was adapted from the literature.^57^ Pd[P(*t*Bu)_3_]_2_/O_2_ was prepared by purging a solution of Pd[P(*t*Bu)_3_]_2_ in toluene (10 mg/mL) with oxygen (3 × 20 mL for 200 mL of catalyst solution), resulting in a rapid color transition from orange to dark-reddish brown. The reaction mixture was left to stir for 16 h. In a dry Schlenk flask under an inert atmosphere, the freshly prepared Pd[P(*t*Bu)_3_]_2_/O_2_ solution (0.728 mL, 10 mg/mL, 10 mol %) and azobenzene **7** (100 mg, 0.285 mmol) were dissolved in dry toluene (1.54 mL) and left to stir at rt for 5 min. Meanwhile, a solution of TMS-acetylene (129 μL, 0.910 mmol) in dry THF (1.25 mL) was stirred at 0 °C (iced water bath), and *n*BuLi (0.571 mL, 1.6 M in hexanes; 0.913 mmol) was slowly added, resulting in a 0.5 M solution, after which the mixture was allowed to warm to rt. The freshly prepared lithium acetylide (1.20 equiv, 0.570 mL, 0.342 mmol) was subsequently added to the reaction mixture over 60 min using a syringe pump and quenched by the addition of *i*PrOH. The reaction mixture was washed with H_2_O (2 × 20 mL), and the organic phase dried over Na_2_SO_4_, filtered, and concentrated under reduced pressure to afford a brown oil. The residue was purified by GRACE automated flash column chromatography on a silica gel with a gradient of DCM and pentane as eluents. An orange–red powder was isolated (63.3 mg, 52% yield). ^1^H NMR (400 MHz, CDCl_3_): δ 7.16–7.11 (m, 2H), 6.87–6.80 (m, 2H), 0.28 (s, 9H); ^19^F NMR (376 MHz, CDCl_3_): δ −102.27 (p, *J* = 8.6 Hz), −115.97 (t, *J* = 8.7 Hz), −120.77 (d, *J* = 9.7 Hz); ^13^C{^1^H} NMR (101 MHz, CDCl_3_): δ 163.4 (dt, *J* = 255.9, 15.0 Hz), 156.7 (ddd, *J* = 264.2, 15.2, 6.4 Hz), 155.4 (dd, *J* = 261.7, 5.1 Hz), 131.9–131.5 (m), 129.1 (d, *J* = 4.7 Hz), 126.9 (t, *J* = 12.1 Hz), 116.4–116.0 (m), 102.1, 101.7 (ddd, *J* = 26.1, 24.4, 4.0 Hz), 100.0, −0.2. HRMS (ESI^+^) *m*/*z*: [M + H]^+^ calcd for C_17_H_13_F_5_N_2_Si + H, 369.0841; found, 369.0846. mp <50 °C.

#### (Trans)-1-(4-ethynyl-2,6-difluorophenyl)-2-(2,4,6-trifluorophenyl)diazene (**10**)

Azobenzene **9** (0.23 g, 0.32 mmol) was dissolved in 10 mL of THF and cooled to −5 °C using an ice/salt bath while stirring. TBAF (1 M in THF, 2 equiv 0.65 mL, 650 mmol) was slowly added to the reaction mixture and left to stir for 30 min at rt. The reaction mixture was diluted with 50 mL of diethyl ether, washed with brine (3 × 50 mL), and dried over MgSO_4_. Subsequently, the solvent was removed by rotary evaporation. Normal-phase flash chromatography on a silica gel was used to purify the crude product using (petroleum ether/acetone, 9:1) as the eluent. The purified product was obtained as an orange–red powder (0.124 g, >95% yield). ^1^H NMR (400 MHz, CD_3_CN): δ 7.33–7.28 (m, 2H), 7.09–7.02 (m, 2H), 3.72 (s, 1H); ^19^F NMR (376 MHz, CD_3_CN): δ −103.55 (p, *J* = 8.9 Hz), −117.95 (t, *J* = 9.3 Hz), −122.44 (d, *J* = 9.8 Hz); ^13^C{^1^H} NMR (101 MHz, CD_3_CN): δ 165.8 (t, *J* = 15.7 Hz), 163.3, 157.4 (ddd, *J* = 261.9, 15.5, 6.5 Hz), 156.1 (dd, *J* = 259.5, 5.4 Hz), 126.6 (t, *J* = 12.6 Hz), 117.7, 102.9 (dd, *J* = 4.1, 1.9 Hz), 83.4, 82.8, 81.4 (t, *J* = 3.5 Hz). HRMS (ESI^+^) *m*/*z*: [M + H]^+^ calcd for C_14_H_5_F_5_N_2_ + H, 297.0446; found, 297.0447. mp 111–115 °C.

#### (Trans)-*N*-(3,5-difluoro-4-((2,4,6-trifluorophenyl)diazenyl)-phenyl)acetamide (**11**)

Azobenzene **7** (0.20 g, 0.57 mmol), Pd_2_(dba)_3_ (0.05 equiv 0.026 g, 0.028 mmol, 5 mol %), Cs_2_CO_3_ (3.5 equiv 0.65 g, 1.995 mmol), XantPhos (0.066 g, 0.114 mmol, 20 mol %), and acetamide (1.2 equiv 0.040 g, 0.684 mmol) were added into a sealable vial. The reaction vial was purged three times with vacuum/N_2_ cycles. The reagents were dissolved in 5.7 mL of dioxane, and the reaction mixture was degassed by five cycles of freeze–pump–thaw. The reaction mixture was left to stir at 90 °C (metal heating mantle) for 4 h. After completion, the reaction mixture was diluted with 40 mL of diethyl ether and filtered through Celite. The organic layer was washed with brine (3 × 20 mL) and dried over MgSO_4_. The solvents were removed by rotary evaporation, resulting in the crude. The crude product was purified by normal-phase flash chromatography on a silica gel (using petroleum ether/ethyl acetate, 8:2 to 7:3, as the eluent). The purified product was obtained as an orange–red powder (0.123 g, 66% yield). ^1^H NMR (400 MHz, CD_3_CN): δ 8.84 (s, 1H), 7.44–7.36 (m, 2H), 7.05–6.97 (m, 2H), 2.21 (s, 3H); ^19^F NMR (376 MHz, CD_3_CN): δ −105.76 (p, *J* = 8.6 Hz), −119.06 (d, *J* = 8.2 Hz), −119.69 (d, *J* = 12.4 Hz); ^13^C{^1^H} NMR (101 MHz, CD_3_CN): δ 170.5, 165.0 (t, *J* = 15.4 Hz), 162.5 (d, *J* = 15.5 Hz), 157.4 (dd, *J* = 258.2, 6.6 Hz), 157.1 (ddd, *J* = 260.1, 15.5, 6.9 Hz), 144.2 (t, *J* = 14.4 Hz), 127.5 (t, *J* = 9.4 Hz), 103.5 (dd, *J* = 25.7, 3.0 Hz), 102.6 (ddd, *J* = 26.7, 25.1, 3.9 Hz), 24.53. HRMS (ESI^+^) *m*/*z*: [M + H]^+^ calcd for C_14_H_8_F_5_N_3_O + H, 330.0660; found, 330.0661. mp 175–178 °C.

#### (Trans)-1-(3,5-difluoro-4-((2,4,6-trifluorophenyl)diazenyl)-phenyl)-1H-pyrrole-2,5-dione (**12**)

Azobenzene **11** (70 mg, 0.21 mmol) was dissolved in MeOH (2.3 mL), and aq HCl was added (6 M, 2.3 mL). The solution was left to stir at 70 °C for 90 min. The precipitated dark-orange solid was filtered off, suspended in water, and saponified with a NaOH solution (2.5 M) until neutral, and the product was extracted with DCM (3 × 5 mL). The crude product (23 mg) was dissolved in chloroform (0.8 mL) and was added dropwise to an ice-cooled solution of maleic anhydride (1.2 equiv, 9.4 mg, 0.1 mmol) in chloroform (0.2 mL). The reaction mixture was left to warm up to rt while stirring for 10 h. The crude reaction mixture was diluted with DCM and THF, and the solvent was removed with the rotary evaporator. The residue (16 mg), AcONa (1.5 equiv, 5.2 mg, 0.064 mmol), and acetic anhydride (10 equiv, 40 μL, 0.42 mmol) were dissolved in THF (40 μL) and left to stir at 80 C for 3 h under a nitrogen atmosphere in a sealed vial. The resulting mixture was diluted with cold water (20 mL), resulting in the formation of a precipitate, which was removed by filtration. The obtained solid was purified via GRACE automatic column chromatography on a silica gel with a gradient of DCM and MeOH as eluents. The final compound was isolated as an orange solid (28 mg, yield over three steps 36%). ^1^H NMR (400 MHz, CDCl_3_): δ 7.36 (d, *J* = 9.8 Hz, 1H), 6.92 (s, 1H), 6.85 (t, *J* = 8.7 Hz, 1H); ^19^F NMR (376 MHz, CDCl_3_): δ −102.38 (p, *J* = 8.5 Hz), −116.08 (t, *J* = 8.7 Hz), −118.82 (d, *J* = 10.0 Hz). ^13^C{^1^H} NMR (101 MHz,, CDCl_3_): δ 168.4, 164.7 (t, *J* = 14.9 Hz), 162.1 (t, *J* = 15.0 Hz), 158.0 (dd, *J* = 15.1, 6.4 Hz), 157.0 (d, *J* = 5.8 Hz), 155.4 (dd, *J* = 15.1, 6.5 Hz), 154.4 (d, *J* = 5.8 Hz), 134.7, 134.1 (t, *J* = 13.5 Hz), 130.3 (t, *J* = 9.9 Hz), 129.1, 109.2 (dd, *J* = 25.0, 3.6 Hz), 101.7 (ddd, *J* = 26.0, 24.4, 4.0 Hz). HRMS (APCI^+^) *m*/*z*: [M + H]^+^ calcd for C_16_H_6_F_5_N_3_O_2_ + H, 368.0453; found, 368.0457. mp 119–120 °C.

#### Trans-4-((4-acetamido-2,6-difluorophenyl)diazenyl)-3,5-difluorobenzenesulfonate (**13**)

Compound **8** (300 mg, 0.726 mmol), acetamide (1.20 equiv, 51.5 mg, 0.871 mmol), Cs_2_CO_3_ (3.5 equiv, 828 mg, 2.54 mmol), Pd_2_dba_3_ (5 mol %, 33.2 mg, 0.0360 mmol), and XantPhos (20 mol %, 84.0 mg, 0.145 mmol) were weighed into a dry sealable vial equipped with a magnetic stirrer. The solids were subjected to three vacuum-dry nitrogen cycles. Peptide-grade DMF (8 mL) was added via a syringe, and the mixture was degassed via five freeze–thaw–pump cycles. (*The mixture was frozen by submerging the vial into liquid nitrogen. The frozen solid was put under vacuum, and the valve was closed, leaving a residual vacuum. The reaction mixture was thawed by submerging it into lukewarm water while observing some bubbling. The mixture was again frozen, and the process was repeated.) The reaction mixture was put under nitrogen and left to stir at 90 °C for 4 h. The crude product mixture was diluted with water/ACN, and most of the solvent was removed by rotary evaporation. The remaining solution was transferred with additional water, loaded on Celite, and freeze-dried. The crude product of Celite was dissolved in water and freeze-dried another two times to remove all the remaining traces of DMF. The crude product was purified by automatic chromatography on a reversed-phase silica gel (C18) with a gradient of the solvent mixture (0–100% 10 mM NH_4_HCO_3_ (aq) in ACN). The product elutes at 30% ACN. The obtained fractions were freeze-dried, and the pure product was isolated as an orange solid (208 mg, yield = 73%). ^1^H NMR (600 MHz, CD_3_CN, drop D_2_O): δ 8.94 (s, 1H), 7.46 (d, *J* = 9.7 Hz, 2H), 7.43 (d, *J* = 12.3 Hz, 2H), 2.12 (s, 3H); ^19^F NMR (565 MHz, CD_3_CN, drop D_2_O): δ −119.68 (d, *J* = 12.5 Hz), −122.25 (d, *J* = 9.8 Hz); ^13^C{^1^H} NMR (151 MHz, CD_3_CN, drop D_2_O): δ 170.6, 156.6 (dd, *J* = 7.8, 4.2 Hz), 154.9 (d, *J* = 6.3 Hz), 152.8 (t, *J* = 7.7 Hz), 144.3 (t, *J* = 14.4 Hz), 132.3, 127.7, 111.3 (d, *J* = 3.8 Hz), 111.2 (d, *J* = 3.8 Hz), 103.7 (d, *J* = 3.1 Hz), 103.5 (d, *J* = 3.0 Hz), 24.6. HRMS (ESI^–^) *m*/*z*: [M]^−^ calcd for C_14_H_8_F_4_N_3_O_4_S^–^, 390.0166; found, 390.0179. mp. >250 °C.

#### (2*R*,3*R*,4*S*,5*R*,6*R*)-2-(Acetoxymethyl)-6-(((2*R*,3*R*,4*S*,5*R*,6*R*)-3,4,5-triacetoxy-6-((4-(4-((trans)-(2,6-difluorophenyl)diaze-nyl)-3,5-difluorophenyl)-1*H*-1,2,3-triazol-1-yl)methyl)tetrahydro-2*H*-pyran-2-yl)oxy)tetrahydro-2*H*-pyran-3,4,5-triyl Triacetate (**16**)

To a solution of compound **5** (100 mg, 0.36 mmol) in dry DCM (20 mL, degassed via two freeze–pump–thaw cycles) was added (OAc)_7_-azidotrehalose **15** (synthesized according to ref (70)) (260 mg, 40 mmol, 1.1 equiv), followed by the addition of Cu(MeCN)_4_PF_6_ (134 mg, 0.36 mmol, 1 equiv). The reaction mixture was stirred overnight at rt. TLC (CH_2_Cl_2_/EtOAc, 9:1, v/v) indicated the appearance of one new product; however, no complete conversion of the starting material was observed. The crude reaction mixture was purified by GRACE automated chromatography on a silica gel with EtOAc and DCM as eluents to obtain the product as a dark-orange solid (278 mg). ^18^F NMR indicated the presence of the PF_6_^–^ ion, as evident from two signals around −72.5 and −74.5 ppm. The product was dissolved in CH_2_Cl_2_ (10 mL) and extracted with water (4 × 7.5 mL). The organic phase was dried using Na_2_SO_4_, filtered, and concentrated under reduced pressure to afford the title compound as a dark-orange solid (260 mg, 77% yield).

PSS_430nm_(80% trans): ^1^H NMR (400 MHz, CD_3_CN): δ 8.30 (s, 1H), 7.68 (d, *J* = 11.2 Hz, 2H), 7.53 (m, 1H), 7.20 (t, *J* = 9.4 Hz, 2H), 5.50–5.31 (m, 3H), 5.10 (dd, *J* = 10.4, 3.7 Hz, 1H), 5.05–4.94 (m, 4H), 4.71 (dd, *J* = 14.7, 2.6 Hz, 1H), 4.64–4.52 (m, 1H), 4.28 (ddd, *J* = 10.3, 7.8, 2.5 Hz, 1H), 4.17 (dd, *J* = 12.5, 6.4 Hz, 1H), 4.03 (d, *J* = 10.8 Hz, 2H), 2.07 (s, 3H), 2.03–1.97 (m, 18H, overlapping with solvent and water signals). ^19^F NMR (376 MHz, CD_3_CN): δ −121.44 (d, *J* = 11.4 Hz), −122.99 (dd, *J* = 10.2, 5.9 Hz). ^13^C{^1^H} NMR (101 MHz CD_3_CN): δ 171.4, 171.2, 171.2, 171.1, 170.8, 170.7, 170.6, 158.0 (dd, *J* = 59.5, 4.6 Hz), 155.4 (dd, *J* = 59.5, 4.5 Hz), 145.6, 136.5 (t, *J* = 11.3 Hz), 133.5 (t, *J* = 10.6 Hz), 132.0 (t, *J* = 10.0, 8.6 Hz), 125.1, 114.1, 113.8, 110.7, 110.5 (d, *J* = 3.0 Hz), 92.8, 92.5, 70.5, 70.4, 70.3, 70.1, 70.1, 70.0, 69.6, 69.2, 62.8, 51.4, 21.0, 21.0, 20.9, 20.9, 20.9, 20.9, 20.8.

Spectroscopic data for the cis isomer (in a 64% cis isomer PSS mixture): ^1^H NMR (400 MHz, CD_3_CN): δ 8.13 (s, 1H), 7.47 (d, *J* = 9.3 Hz, 2H), 7.40–7.27 (m, 1H), 7.01 (t, *J* = 8.5 Hz, 2H), 5.52–5.24 (m, 5H), 5.11–4.85 (m, 9H), 4.75–4.43 (m, 4H), 4.33–4.19 (m, 1H), 4.14 (dd, *J* = 12.2, 6.1 Hz, 2H), 4.05–3.94 (m, 3H), 2.03 (s, 3H), 2.00–1.91 (m, 21H, overlapping with the solvent peak). HRMS (ESI^+^) *m*/*z*: [M + H]^+^ calcd for C_40_H_41_F_4_N_5_O_17_ + H, 940.2506; found, 940.2494. mp 99–102 °C.

#### (2*R*,3*S*,4*S*,5*R*,6*R*)-2-((4-(4-((trans)-(2,6-Difluorophenyl)diaz-enyl)-3,5-difluorophenyl)-1*H*-1,2,3-triazol-1-yl)methyl)-6-(((2*R*,3*R*,4*S*,5*S*,6*R*)-3,4,5-trihydroxy-6-(hydroxymethyl)tetrahydro-2*H*-pyran-2-yl)oxy)tetrahydro-2*H*-pyran-3,4,5-triol (**17**)

To a solution of **16** (90 mg, 96 μmol) in dry methanol (2 mL) was added a stock solution of sodium methoxide in methanol (1.3 mg, 23.9 μmol, 25 mol %), and the reaction mixture was allowed to stir for 2 h. TLC (CH_2_Cl_2_/EtOAc, 85:15, v/v) indicated complete conversion of the starting material. The reaction mixture was neutralized using Amberlyst 15 hydrogen form (15 min) and filtered, and the filtrate was concentrated under reduced pressure. The residue was dissolved in dH_2_O and freeze-dried, affording an orange solid (61 mg, 98% yield). PSS_430nm_(81% trans): ^1^H NMR (400 MHz, CD_3_CN, drop of D_2_O): δ 8.42 (d, *J* = 1.2 Hz, 1H), 7.72–7.60 (m, 2H), 7.56–7.43 (m, 1H), 7.18 (ddd, *J* = 9.7, 8.5, 1.3 Hz, 2H), 5.03 (d, *J* = 3.7 Hz, 1H), 4.80–4.72 (m, 1H), 4.70 (d, *J* = 3.9 Hz, 1H), 4.67–4.56 (m, 1H), 4.16 (t, *J* = 8.4 Hz, 1H), 3.83–3.74 (m, 2H), 3.73–3.64 (m, 4H), 3.58–3.50 (m, 1H), 3.26–3.10 (m, 3H). Part of the signals overlaps with the water signal. ^19^F NMR (376 MHz, CD_3_CN, drop of D_2_O): δ −121.27 (d, *J* = 11.1 Hz), −123.03 (dd, *J* = 10.3, 6.0 Hz). ^13^C{^1^H} NMR (101 MHz, CD_3_CN, drop of D_2_O): δ 158.1 (d, *J* = 4.9 Hz), 157.5 (d, *J* = 4.1 Hz), 155.5 (d, *J* = 5.0 Hz), 154.9 (d, *J* = 4.1 Hz), 145.3, 135.8 (t, *J* = 11.4 Hz), 133.6 (t, *J* = 10.7 Hz), 132.4–131.9 (m), 125.4, 113.8 (dd, *J* = 20.5, 3.4 Hz), 110.5 (d, *J* = 23.9 Hz), 94.3, 94.2, 73.7, 73.5, 73.5, 72.1 (d, *J* = 1.8 Hz), 72.0, 71.8 (d, *J* = 2.0 Hz), 70.9, 70.8, 61.9, 52.0.

Spectroscopic data for the cis isomer (in an 86% cis isomer PSS mixture): ^1^H NMR (400 MHz, CD_3_CN, drop of D_2_O): δ 8.30 (d, *J* = 1.4 Hz, 1H), 7.51 (d, *J* = 9.5 Hz, 2H), 7.45–7.34 (m, 1H), 7.04 (t, *J* = 8.7 Hz, 2H), 5.02 (d, *J* = 3.8 Hz, 1H), 4.81–4.66 (m, 2H), 4.56 (ddd, *J* = 14.5, 7.6, 1.7 Hz, 1H), 4.16 (ddd, *J* = 10.1, 7.5, 2.6 Hz, 1H), 3.86–3.64 (m, 5H), 3.54 (dd, *J* = 12.5, 6.4 Hz, 1H), 3.41 (ddd, *J* = 9.7, 3.8, 1.7 Hz, 1H), 3.34–3.25 (m, 1H), 3.24–3.09 (m, 2H). HRMS (ESI^+^) *m*/*z*: [M + H]^+^ calcd for C_26_H_27_F_4_N_5_O_10_ + H, 646.1767; found, 646.1754. mp decomposition at 174–180 °C.

### Mycobacterial Culturing and Labeling

To 100 mL of a freshly prepared culture of *M. smegmatis* MC^2^-155, from a single colony, at OD_600_ = 0.6 (log phase), the trehalose–azobenzene conjugate **17** (6.5 mg) was added to afford a final concentration of 100 μM. The bacteria were incubated for 24 h, at 30 °C, while shaking at 200 rpm. A final OD_600_ = 0.76 was obtained for the untreated bacteria (control) and OD_600_ = 0.66 for the labeled bacteria.

The bacterial culture was centrifuged at 4000 RPM, and the supernatant was removed. The bacteria were resuspended in PBS buffer (20 mL) and again centrifuged. This process was performed three times in total. The bacterial pellet was resuspended in MeOH (3 mL) and transferred to a 15 mL conical glass centrifuge tube. To the suspension of bacteria in MeOH (3 mL) was added CHCl_3_ (6 mL), and the lipids were extracted for 1 h on a rocking plate at rt. This suspension was centrifuged at 2000*g*, resulting in a two-phase system from which the lower, organic layer was collected. The organic phase was then filtered over a 2 μm hydrophilic filter to remove solid cellular debris. The slightly yellow-colored solution was concentrated under reduced pressure on a rotary evaporator (water bath at 40 °C), providing the mycobacterial lipids (∼60 mg of the control and ∼42 mg of the labeled bacteria).

## Supporting Information Available

List of all synthesized compounds; synthetic schemes; ^1^H NMR, ^19^F NMR, and ^13^C NMR spectra; UV–vis spectra; FT-IR spectra; and NMR studies of lipid extracts (PDF)
