# Supplementary material for: Design and Synthesis of Visible-Light-Responsive Azobenzene Building Blocks for Chemical Biology
Source: J Org Chem. 2022 Oct 26;87(21):14319–33. doi: 10.1021/acs.joc.2c01777 (PMC9639001; doi:10.1021/acs.joc.2c01777)
Supplement: Supplementary file 1 — jo2c01777_si_001.pdf [file jo2c01777_si_001.pdf]

Design and Synthesis of Visible Light-responsive Azobenzene Building Blocks for Chemical Biology

Jana Volarić,[a] Jeffrey Buter,[a] Albert M. Schulte,[a] Keimpe-Oeds van den Berg,[a,†] Eduardo Santamaría-Aranda,[a,b,‡] Wiktor Szymanski,[a,c]* Ben L. Feringa[a]*

[a] Stratingh Institute for Chemistry, University of Groningen, 9747 AG Groningen, The Netherlands

[b] Departamento de Química, Universidad de la Rioja, Centro de investigación en Síntesis Química, Madre de Dios 53, 26006 Logroño, Spain

[c] Department of Radiology, Medical Imaging, Center, University of Groningen, University Medical Center Groningen, 9713 GZ Groningen, The Netherlands

† Symeres BV, 9747 AT Groningen, The Netherlands

‡ Department of Sustainability and Advanced Materials, Footwear Technology Center of La Rioja (CTCR), 26580 Arnedo, La Rioja, Spain

* to whom correspondence should be addressed: w.szymanski@umcg.nl; b.l.feringa@rug.nl

Table of contents

1. Chemical Synthesis……………………………………………………………..………..S3

2. Analytical data of all synthesized compounds……………………………..……….S5

3. ^{1}H, ^{19}F and ^{13}C NMR spectra……………………………………………..…...…S9

4. FT-IR spectra………………………………...……………………………….…….S45

5. HRMS data…………………...………………………….……………………….….….S46

6. Photochemical properties……………….……………..…………...………………..….S58

    6.1 Photochemical properties in DMSO………….……………………….…S59

    6.2 Photochemical properties in aqueous medium……………………......S71

    6.3 19F NMR spectroscopy of the unlabeled and labeled lipid extract……S74

7. References…………………………………………………………………………S74

1. Chemical Synthesis

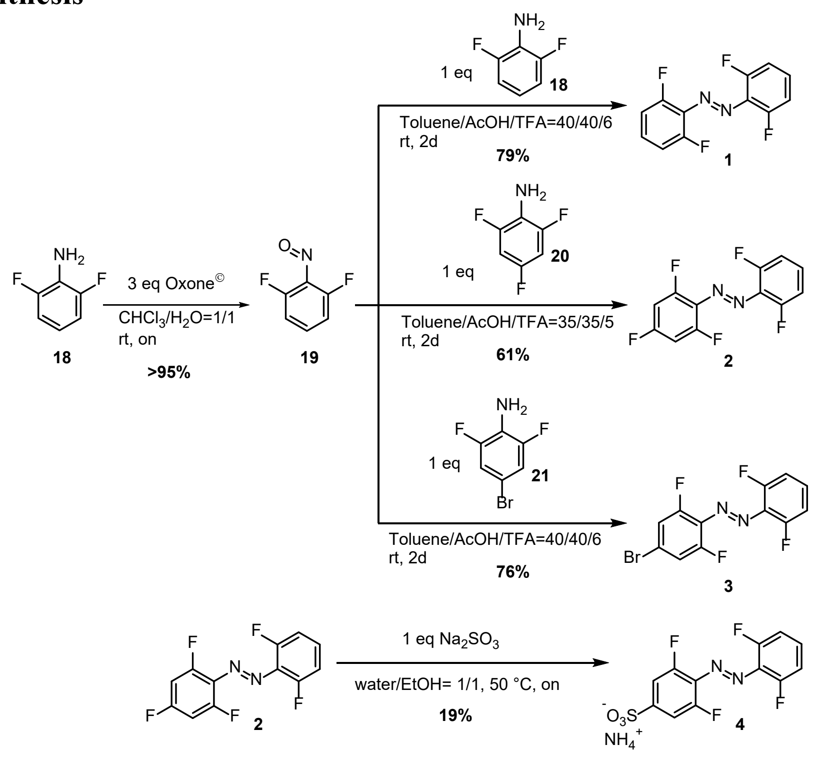

Scheme S1. Synthesis of mono-*para*-substituted tetra-*ortho*-fluoro azobenzenes **1**,**2**,**3** and **4**.

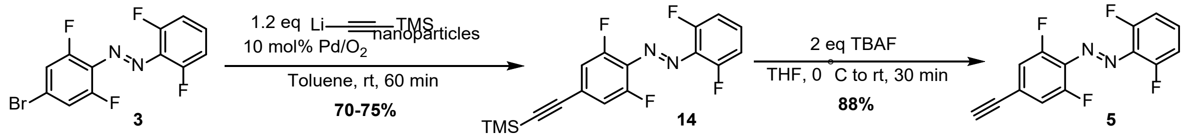

Scheme S2. Synthesis of the mono-*para*-substituted tetra-*ortho*-fluoro azobenzene **5** furnished with an alkyne moiety via the palladium-catalyzed cross-coupling procedure.[1]

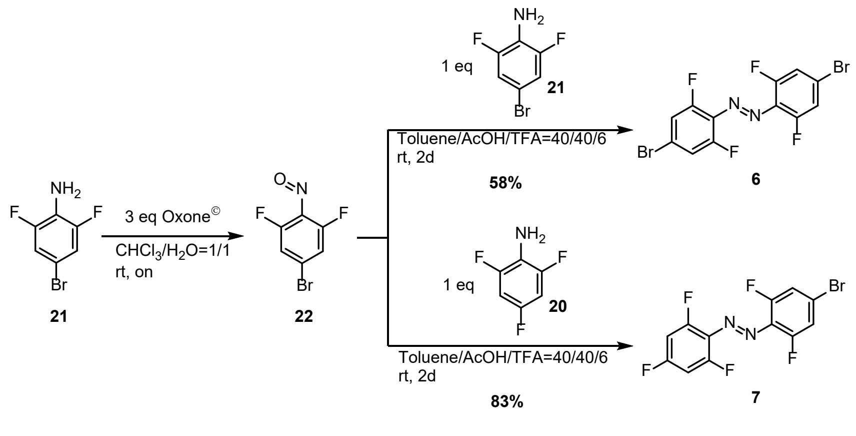

Scheme S3. Synthesis of the bis-*para*-substituted tetra-*ortho*-fluoro azobenzenes **6** and **7**.

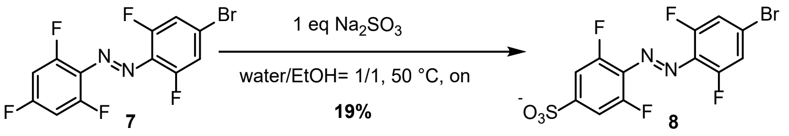

Scheme S4. Synthesis of the sulfonated bis-*para*-substituted tetra-*ortho*-fluoro azobenzenes **8** via SNAr.

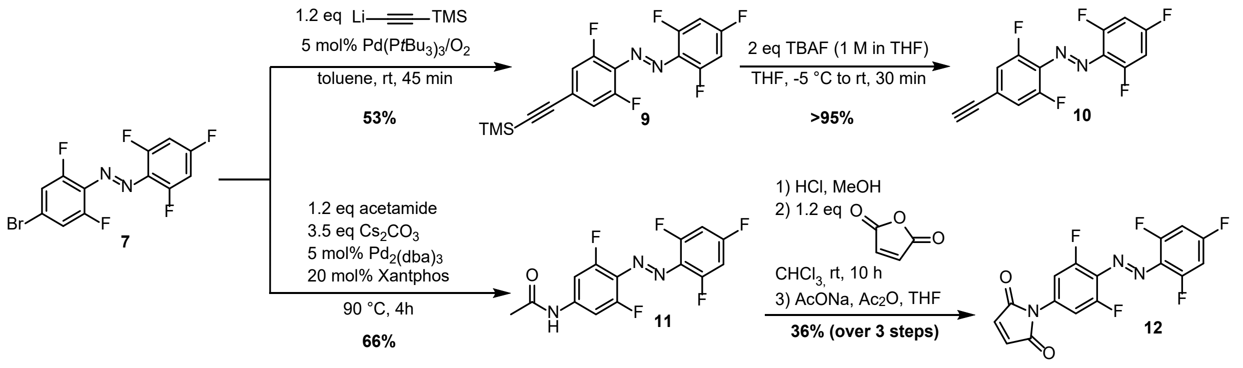

Scheme S5. Synthesis of the bis-*para*-substituted tetra-*ortho*-fluoro azobenzenes functionalized with an alkyne (**10**) and a maleimide group (**12**).

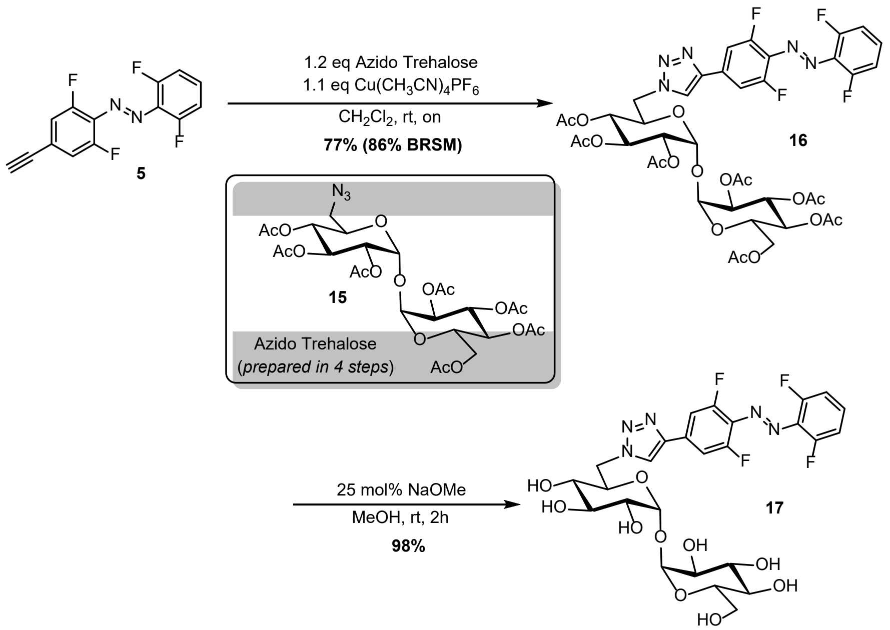

Scheme S6. Synthesis of the tetra-*ortho*-fluoro azobenzene **17** functionalized with a trehalose group

2. Analytical data of all synthesized compounds

1,3-difluoro-2-nitrosobenzene (19)[2]

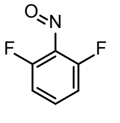

19

^{1}H NMR (400 MHz, $CDCl_3$) δ 7.68 – 7.58 (m, 1H), 7.12 (t, *J* = 8.6 Hz, 2H).
^{19}F NMR (376 MHz, $CDCl_3$) δ -130.24.
FTIR (ATR): 1607s (C=C), 1474s (N=O), 1243s (C-N), 1014s (C-N), 416m (C-F).
Mp. 109-111 °C.
The spectroscopic data is in accordance with the literature.[2]

***trans*-1,2-bis(2,6-difluorophenyl)diazene (1)**[3,4]

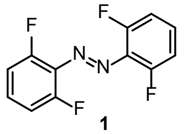

1

^{1}H NMR (400 MHz, $CDCl_3$) δ 7.43 – 7.33 (m, 1H), 7.07 (m, 2H).
^{19}F NMR (376 MHz, $CDCl_3$) δ -121.25 (dd, J = 9.0, 5.8 Hz).
^{1}H NMR (400 MHz, DMSO-d_6) δ 7.69 – 7.60 (m, 1H), 7.37 (t, *J* = 8.4 Hz, 2H).
^{19}F NMR (376 MHz, DMSO-d_6) δ -122.02.
HRMS (ESI+) m/z: $[M + H]^+$ Calcd for $C_{12}H_6F_4N_2$+H 255.0539; Found 255.0539.
Mp. 75-79 °C.
The spectroscopic data is in accordance with the literature.[3,4]

(*trans*)-1-(2,6-difluorophenyl)-2-(2,4,6-trifluorophenyl)diazenediazene (2)

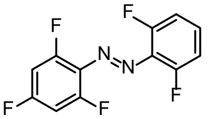

2

^{1}H NMR (400 MHz, DMSO-d_6) δ 7.58 (tt, *J* = 8.5, 6.1 Hz, 1H), 7.41 (t, *J* = 9.6 Hz, 2H), 7.30 (t, *J* = 9.0 Hz, 2H).
^{19}NMR (376 MHz, DMSO-d_6) δ -102.22 (p, *J* = 8.9 Hz, 1F), -117.33 (t, *J* = 9.3 Hz, 2F), -121.81 (dd, *J* = 10.4, 6.1 Hz, 2F).
^{13}C{^{1}H} NMR (101 MHz, DMSO-d_6) δ 163.0 (dt, *J* = 253.3, 15.8 Hz), 157.0 (dd, *J* = 16.1, 6.6 Hz), 154.7 (dd, *J* = 259.4, 4.1 Hz), 133.0 (t, *J* = 10.7 Hz), 130.6 (t, *J* = 9.9 Hz), 128.1 (d, *J* = 5.2 Hz), 113.1 (dd, *J* = 20.0, 3.5 Hz), 102.22 (ddd, *J* = 26.9, 24.7, 3.9 Hz).
HRMS (APCI+) m/z: $[M + H]^+$ Calcd for $C_{12}H_6F_5N_2$+H 273.0446; Found: 273.0447
Mp. 77-78 °C.

***trans*-1-(4-bromo-2,6-difluorophenyl)-2-(2,6-difluorophenyl)diazene (3)**[5,6]

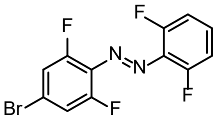

3

^{1}H NMR (400 MHz, $CDCl_3$) δ 7.43 – 7.34 (m, 1H), 7.26 (d, J = 8.0 Hz, 2H), 7.06 (t, J = 8.7 Hz, 2H).
^{19}F NMR (376 MHz, $CDCl_3$) δ -119.04 (d, *J* = 9.1 Hz), -120.72 (q, *J* = 6.3, 5.7, 2.8 Hz).
^{1}H NMR (400 MHz, DMSO-d_6) δ 7.77 (d, *J* = 9.4 Hz, 2H), 7.64 (ddd, *J* = 14.5, 8.6, 6.3 Hz, 1H), 7.35 (t, *J* = 9.6 Hz, 2H).
^{19}F NMR (376 MHz, DMSO-d_6) δ -119.85 (d, *J* = 12.2 Hz), -121.50 (dd, *J* = 10.2, 5.9 Hz).
HRMS (APCI+) m/z: [M + H]+ Calcd for $C_{12}H_6BrF_4N_2$+H 332.9645; Found: 332.9650.
Mp. 60-63 °C.
The spectroscopic data is in accordance with the literature.[5,6]

(*trans*)-4-((2,6-difluorophenyl)diazenyl)-3,5-difluorobenzenesulfonate (4)

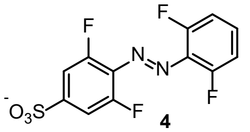

^{1}H NMR (400 MHz, CD_3CN, drop D_2O) δ 7.54 – 7.49 (m, 3H), 7.17 (ddd, *J* = 10.1, 8.5, 1.6 Hz, 2H).
^{19}F NMR (376 MHz, CD_3CN, drop D_2O) δ -120.92 (d, *J* = 9.1 Hz), -122.53 (dd, *J* = 10.5, 6.1 Hz).
^{13}C{^{1}H} NMR (101 MHz, CD3CN, drop D2O) δ 157.3 (dd, J = 65.4, 4.0 Hz), 154.7 (dd, J = 67.4, 3.9 Hz), 149.8 (t, *J* = 8.4 Hz), 134.2 (t, *J* = 10.8 Hz), 133.8 (t, *J* = 10.6 Hz), 132.4 (t, *J* = 10.0 Hz), 114.0 (dd, *J* = 20.5, 3.6 Hz), 111.6 (dd, *J* = 23.0, 3.2 Hz).
HRMS (ESI-) m/z: $[M]^-$ Calcd for $C_{12}H_5O_3F_4N_2S^-$ 332.9963; Found: 332.9965.
Mp. >250 °C.
*The issue of aggregate formation at higher concentrations limited the amount of compound used for NMR spectroscopy. Due to C-F coupling, presence of *trans*/*cis* mixture and the low concentration making the 1D spectra complex for analysis, 2D NMR spectra were added for characterization.

(*trans*)-1-(2,6-difluoro-4-((trimethylsilyl)ethynyl)phenyl)-2-(2,6-difluorophenyl)diazene (14)

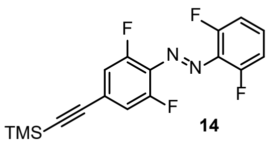

14

^{1}H NMR (400 MHz, CD_3CN, *trans*) δ 7.54 (tt, *J* = 8.5, 6.0 Hz, 1H), 7.25 (d, *J* = 9.4 Hz, 2H), 7.19 (dd, *J* = 9.5, 8.5 Hz, 2H), 0.27 (s, 9H).
^{1}H NMR (400 MHz, CD_3CN, cis) δ 7.37 (tt, *J* = 8.6, 6.3 Hz, 1H), 7.10 – 6.96 (m, 4H), 0.22 (s, 9H).
^{19}F NMR (376 MHz, CD_3CN) δ -121.33 (dt, *J* = 8.7, 5.6 Hz, cis), -121.85 – -121.98 (m, *cis*), -122.45 (d, *J* = 9.9 Hz, *trans*), -122.72 (dd, *J* = 9.9, 6.0 Hz, *trans*).
^{13}C{^{1}H} NMR (101 MHz, CD3CN, trans) δ 156.3 (ddd, *J* = 260.1, 28.8, 4.7 Hz), 152.4 (ddd, *J* = 251.9, 21.1, 5.8 Hz), 133.8 (t, *J* = 10.7 Hz), 132.1 (t, J = 9.8 Hz), 127.6 (t, *J* = 12.3 Hz), 126.1 (t, *J* = 11.4 Hz), 117.1 (d, *J* = 25.9 Hz), 113.9 (d, *J* = 24.1 Hz), 102.7, 100.7, -0.3.
HRMS (APCI+) m/z: $[M + H]^+$ Calcd for $C_{17}H_{14}F_4N_2Si$+H^- 351.0935; Found: 351.0938.

(*trans*)-1-(2,6-difluorophenyl)-2-(4-ethynyl-2,6-difluorophenyl)diazene (5)

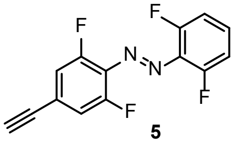

5

^{1}H NMR (400 MHz, CD_3CN) δ 7.54 (tt, *J* = 8.6, 6.0 Hz, 1H), 7.35 – 7.29 (m, 2H), 7.24 – 7.16 (m, 2H), 3.72 (s, 1H).
^{19}F NMR (376 MHz, CD_3CN) δ -122.57 (dd, *J* = 9.6, 3.3 Hz), -122.60 – -122.68 (m).
^{13}C{^{1}H} NMR (101 MHz, CD_3CN) δ 157.5 (d, *J* = 4.0 Hz), 157.2 (d, *J* = 5.4 Hz), 155.0 (d, *J* = 4.1 Hz), 154.6 (d, *J* = 5.3 Hz), 133.7 (t, *J* = 10.8 Hz), 132.4 (t, *J* = 10.0 Hz), 131.9 (t, *J* = 9.9, 8.6 Hz), 126.5 (t, *J* = 12.5 Hz), 117.4 – 117.1 (m), 113.7 (dd, *J* = 20.4, 3.6 Hz), 83.2, 81.3 (t, *J* = 3.5 Hz).
HRMS (APCI+) m/z: [M + H]+ Calcd for $C_{14}H_6F_4N_2$ +H 279.0540; Found: 279.0540.
Mp. 73-74 °C.

5-bromo-1,3-difluoro-2-nitrosobenzene (22)[7]

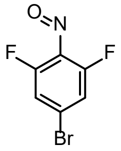

22

FTIR (ATR): 1601s (C=C), 1431s (N-O), 1275s (C-N), 1060s (C-N), 542m (C-F).
^{1}H NMR (400 MHz, $CDCl_3$) δ 7.38 – 7.30 (m, 2H).
^{19}F NMR (376 MHz, $CDCl_3$) δ -128.66 (d, *J* = 8.2 Hz).
Mp. 87–90 °C.
The spectroscopic data is in accordance with the literature.[7]

***trans*-1,2-bis(4-bromo-2,6-difluorophenyl)diazene (6)**[8,9]

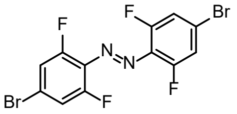

6

^{1}H NMR (400 MHz, $CHCl_3$) δ 7.27 (d, *J* = 8.1 Hz, 1H).
^{19}F NMR (376 MHz, $CHCl_3$) δ -118.63 (d, J = 8.5 Hz).
^{1}H NMR (400 MHz, DMSO-d_6) δ 7.85 – 7.67 (m, 1H).
^{19}F NMR (376 MHz, DMSO-d_6) δ -119.37 (d, *J* = 9.5 Hz).
HRMS (ESI+) m/z: [M + H]+ Calcd for $C_{12}H_4Br_2F_4N_2$+H 410.8750; Found: 410.8742.
Mp. 164-168°C.
The spectroscopic data is in accordance with the literature.[8,9]

(*trans*)-1-(4-bromo-2,6-difluorophenyl)-2-(2,4,6-trifluorophenyl)diazene (7)

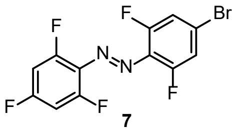

7

^{1}H NMR (400 MHz, $CDCl_3$) δ 7.28-7.23 (m, 2H), 6.88-6.80 (t, *J* = 8.8 Hz, 2H),
^{19}F NMR (376 MHz, $CDCl_3$) δ -102.04 (p, *J* = 8.5 Hz), -115.95 (t, *J* = 8.8 Hz), -118.63 (d, *J* = 8.4 Hz), -119.03 (d, *J* = 8.4 Hz).
^{13}C{^{1}H} NMR (101 MHz, $CDCl_3$) δ 165.1 (t, *J* = 15.1 Hz), 162.6 (td, *J* = 15.2, 14.9, 13.7 Hz), 158.5 – 158.1 (m), 157.2 (d, J = 5.1 Hz), 155.7 (dd, *J* = 15.2, 6.2 Hz), 154.6 (d, *J* = 5.9 Hz), 131.2, 124.5 (t, *J* = 11.9 Hz), 117.0 (d, *J* = 27.4 Hz), 102.4 – 101.7 (m).
^{1}H NMR (400 MHz, DMSO-d_6) δ 7.77 (d, *J* = 9.6 Hz, 1H), 7.51 (t, *J* = 9.7 Hz, 1H).
^{19}F NMR (376 MHz, DMSO-d_6) δ -101.40, -116.88, -119.34, -119.74.
^{13}C{^{1}H} NMR (101 MHz, DMSO-d_6) δ 164.5 (t, *J* = 15.7 Hz), 161.9 (t, *J* = 15.9 Hz), 157.1 (dd, *J* = 16.0, 6.5 Hz), 156.0 (d, *J* = 5.1 Hz), 154.5 (dd, *J* = 16.1, 6.5 Hz), 153.4 (d, *J* = 5.0 Hz), 129.8 (t, *J* = 9.7 Hz), 124.4 (t, *J* = 12.4 Hz), 117.1 (d, *J* = 23.7 Hz), 102.7 – 102.0 (m).
HRMS (ESI+) m/z: [M + H]+ Calcd for $C_{12}H_4BrF_5N_2$+H 350.9551; Found: 350.9554.
Mp: 82-95°C.

(*trans*)-4-((4-bromo-2,6-difluorophenyl)diazenyl)-3,5-difluorobenzenesulfonate (8)

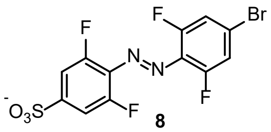

8

^{1}H NMR (600 MHz, CD_3CN, drop D_2O) δ 7.57 – 7.49 (m, 1H), 7.45 (d, *J* = 8.7 Hz, 1H).
^{19}F NMR (565 MHz, CD_3CN) δ -120.46 (dd, *J* = 29.7, 10.2 Hz).
^{13}C{^{1}H} NMR (151 MHz, CD_3CN) δ 156.2, 155.7, 154.5, 154.0, 149.4, 131.9, 130.3, 124.7, 117.1, 117.0, 110.8.
^{13}C{^{1}H} NMR (151 MHz, CD_3CN, drop D_2O) δ 156.2, 155.7, 154.5, 154.0, 149.34, 131.9, 130.4, 124.7, 117.1, 117.0, 110.8.
^{1}H NMR (400 MHz, DMSO-d_6) δ 8.41 (s, 1H), 7.79 (d, *J* = 9.3 Hz, 2H), 7.42 (d, *J* = 10.1 Hz, 2H).
^{19}F NMR (376 MHz, DMSO-d_6) δ -119.45, -119.96.
HRMS (ESI-) m/z: $[M]^-$ Calcd for $C_{12}H_4BrF_4N_2O_3S^-$ 410.9068; Found: 410.9060.
Mp. > 250 C.

(*trans*)-1-(2,6-difluoro-4-((trimethylsilyl)ethynyl)phenyl)-2-(2,4,6-trifluorophenyl)diazene (9)

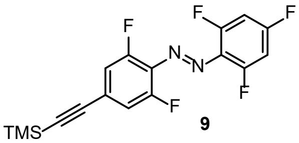

9

^{1}H NMR (400 MHz, $CDCl_3$) δ 7.16 – 7.11 (m, 2H), 6.87 – 6.80 (m, 2H), 0.28 (s, 9H).
^{19}F NMR (376 MHz, $CDCl_3$) δ -102.27 (p, *J* = 8.6 Hz), -115.97 (t, *J* = 8.7 Hz), -120.77 (d, *J* = 9.7 Hz).
^{13}C{^{1}H} NMR (101 MHz, $CDCl_3$) δ 163.4 (dt, *J* = 255.9, 15.0 Hz), 156.7 (ddd, *J* = 264.2, 15.2, 6.4 Hz), 155.4 (dd, *J* = 261.7, 5.1 Hz), 131.9 – 131.5 (m), 129.1 (d, *J* = 4.7 Hz), 126.9 (t, *J* = 12.1 Hz), 116.4 – 116.0 (m), 102.1, 101.7 (ddd, J = 26.1, 24.4, 4.0 Hz), 100.0, -0.2.
HRMS (ESI+) m/z: [M + H]+ Calcd for $C_{17}H_{13}F_5N_2Si$+H 369.0841; Found: 369.0846.
Mp. <50°C.

(*trans*)-1-(4-ethynyl-2,6-difluorophenyl)-2-(2,4,6-trifluorophenyl)diazene (10)

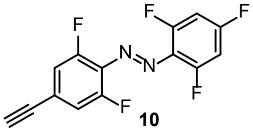

10

^{1}H NMR (400 MHz, CD_3CN) δ 7.33 – 7.28 (m, 2H), 7.09 – 7.02 (m, 2H), 3.72 (s, 1H).
^{19}F NMR (376 MHz, CD_3CN) δ -103.55 (p, *J* = 8.9 Hz), -117.95 (t, *J* = 9.3 Hz), -122.44 (d, *J* = 9.8 Hz).
^{13}C{^{1}H} NMR (101 MHz, CD_3CN) δ 165.8 (t, *J* = 15.7 Hz), 163.3, 157.4 (ddd, *J* = 261.9, 15.5, 6.5 Hz), 156.1 (dd, *J* = 259.5, 5.4 Hz), 126.6 (t, *J* = 12.6 Hz), 117.7, 102.9 (dd, *J* = 4.1, 1.9 Hz), 83.4, 82.8, 81.4 (t, *J* = 3.5 Hz).
HRMS (ESI+) m/z: [M + H]+ Calcd for $C_{14}H_5F_5N_2$+H 297.0446; Found: 297.0447.
Mp. 111-115°C.

(*trans*)-*N*-(3,5-difluoro-4-((2,4,6-trifluorophenyl)diazenyl)phenyl)acetamide (11)

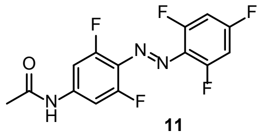

11

^{1}H NMR (400 MHz, CD_3CN) δ 8.84 (s, 1H), 7.44 – 7.36 (m, 2H), 7.05 – 6.97 (m, 2H), 2.21 (s, 3H).
19FNMR (376 MHz, CD_3CN) δ -105.76 (p, *J* = 8.6 Hz), -119.06 (d, *J* = 8.2 Hz), -119.69 (d, *J* = 12.4 Hz).
^{13}C{^{1}H} NMR (101 MHz, CD_3CN) δ 170.5, 165.0 (t, *J* = 15.4 Hz), 162.5 (d, *J* = 15.5 Hz), 157.4 (dd, *J* = 258.2, 6.6 Hz), 157.1 (ddd, *J* = 260.1, 15.5, 6.9 Hz), 144.2 (t, *J* = 14.4 Hz), 127.5 (t, *J* = 9.4 Hz), 103.5 (dd, *J* = 25.7, 3.0 Hz), 102.6 (ddd, *J* = 26.7, 25.1, 3.9 Hz), 24.53.
HRMS (ESI+) m/z: [M + H]+ Calcd for $C_{14}H_8F_5N_3O$+H 330.0660; Found: 330.0661.
Mp. 175-178 °C.

(*trans*)-1-(3,5-difluoro-4-((2,4,6-trifluorophenyl)diazenyl)phenyl)-1H-pyrrole-2,5-dione (12)

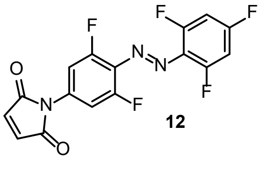

12

^{1}H NMR (400 MHz, $CDCl_3$) δ 7.36 (d, *J* = 9.8 Hz, 1H), 6.92 (s, 1H), 6.85 (t, *J* = 8.7 Hz, 1H).
^{19}F NMR (376 MHz, $CDCl_3$) δ -102.38 (p, *J* = 8.5 Hz), -116.08 (t, *J* = 8.7 Hz), -118.82 (d, *J* = 10.0 Hz).
^{13}C{^{1}H} NMR (101 MHz, , $CDCl_3$) δ 168.4, 164.7 (t, *J* = 14.9 Hz), 162.1 (t, *J* = 15.0 Hz), 158.0 (dd, *J* = 15.1, 6.4 Hz), 157.0 (d, *J* = 5.8 Hz), 155.4 (dd, *J* = 15.1, 6.5 Hz), 154.4 (d, *J* = 5.8 Hz), 134.7, 134.1 (t, *J* = 13.5 Hz), 130.3 (t, *J* = 9.9 Hz), 129.1, 109.2 (dd, *J* = 25.0, 3.6 Hz), 101.7 (ddd, *J* = 26.0, 24.4, 4.0 Hz).
HRMS (APCI+) m/z: [M + H]+ Calcd for $C_{16}H_6F_5N_3O_2$ +H 368.0453; Found: 368.0457.
Mp. 119-120 °C.

***trans*-4-((4-acetamido-2,6-difluorophenyl)diazenyl)-3,5-difluorobenzenesulfonate (13)**
^{1}H NMR (400 MHz, CD_3CN, drop D_2O) δ 8.94 (s, 1H), 7.46 (d, *J* = 9.7 Hz, 2H), 7.43 (d, *J* = 12.3 Hz, 2H), 2.12 (s, 3H).
^{19}F NMR (376 MHz, CD_3CN, drop D_2O) δ -119.68 (d, *J* = 12.5 Hz), -122.25 (d, *J* = 9.8 Hz).

$^{13}C\{^1H\}$ NMR (101 MHz, CD_3CN, drop D_2O) δ 170.6, 156.6 (dd, *J* = 7.8, 4.2 Hz), 154.9 (d, *J* = 6.3 Hz), 152.8 (t, *J* = 7.7 Hz), 144.3 (t, *J* = 14.4 Hz), 132.3, 127.7, 111.3 (d, *J* = 3.8 Hz), 111.2 (d, *J* = 3.8 Hz), 103.7 (d, *J* = 3.1 Hz), 103.5 (d, *J* = 3.0 Hz), 24.6.
HRMS (ESI-) m/z: [M]- Calcd for $C_{14}H_8F_4N_3O_4S^-$ 390.0166 Found: 390.0179.
Mp.>250 °C.

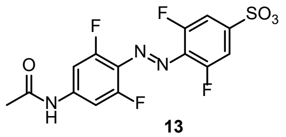

13

(2*R*,3*R*,4*S*,5*R*,6*R*)-2-(acetoxymethyl)-6-(((2*R*,3*R*,4*S*,5*R*,6*R*)-3,4,5-triacetoxy-6-((4-(4-((*trans*)-(2,6-difluorophenyl)diazenyl)-3,5-difluorophenyl)-1*H*-1,2,3-triazol-1-yl)methyl)tetrahydro-2*H*-pyran-2-yl)oxy)tetrahydro-2*H*-pyran-3,4,5-triyl triacetate (16)

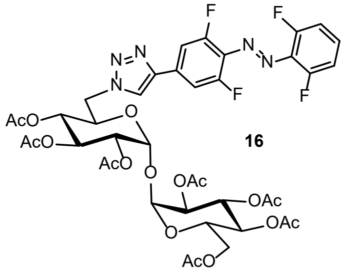

16

PSS_{430nm}(80% *trans*):
^1H NMR (400 MHz, CD_3CN) δ 8.30 (s, 1H), 7.68 (d, *J* = 11.2 Hz, 2H), 7.53 (m, 1H), 7.20 (t, *J* = 9.4 Hz, 2H), 5.50 – 5.31 (m, 3H), 5.10 (dd, *J* = 10.4, 3.7 Hz, 1H), 5.05 – 4.94 (m, 4H), 4.71 (dd, *J* = 14.7, 2.6 Hz, 1H), 4.64 – 4.52 (m, 1H), 4.28 (ddd, *J* = 10.3, 7.8, 2.5 Hz, 1H), 4.17 (dd, *J* = 12.5, 6.4 Hz, 1H), 4.03 (d, *J* = 10.8 Hz, 2H), 2.07 (s, 3H), 2.03 – 1.97 (m, 18H, overlapping with solvent and water signals).
^{19}F NMR (376 MHz, CD_3CN) δ -121.44 (d, *J* = 11.4 Hz), -122.99 (dd, *J* = 10.2, 5.9 Hz).
$^{13}C\{^1H\}$ NMR (101 MHz CD_3CN) δ 171.4, 171.2, 171.2, 171.1, 170.8, 170.7, 170.6, 158.0 (dd, *J* = 59.5, 4.6 Hz), 155.4 (dd, *J* = 59.5, 4.5 Hz), 145.6, 136.5 (t, *J* = 11.3 Hz), 133.5 (t, *J* = 10.6 Hz), 132.0 (t, *J* = 10.0, 8.6 Hz), 125.1, 114.1, 113.8, 110.7, 110.5 (d, *J* = 3.0 Hz), 92.8, 92.5, 70.5, 70.4, 70.3, 70.1, 70.1, 70.0, 69.6, 69.2, 62.8, 51.4, 21.0, 21.0, 20.9, 20.9, 20.9, 20.9, 20.8.

Spectroscopic data for the *cis* isomer (in a 64 % *cis* isomer PSS mixture): ^1HNMR (400 MHz, CD_3CN) δ 8.13 (s, 1H), 7.47 (d, *J* = 9.3 Hz, 2H), 7.40 – 7.27 (m, 1H), 7.01 (t, *J* = 8.5 Hz, 2H), 5.52 – 5.24 (m, 5H), 5.11 – 4.85 (m, 9H), 4.75 – 4.43 (m, 4H), 4.33 – 4.19 (m, 1H), 4.14 (dd, *J* = 12.2, 6.1 Hz, 2H), 4.05 – 3.94 (m, 3H), 2.03 (s, 3H), 2.00 – 1.91 (m, 21H, overlapping with solvent peak).

HRMS (ESI+) m/z: [M + H]+ Calcd for $C_{40}H_{41}F_4N_5O_{17}$+H 940.2506; Found: 940.2494.
Mp. 99-102 °C.

(2*R*,3*S*,4*S*,5*R*,6*R*)-2-((4-(4-((*trans*)-(2,6-difluorophenyl)diazenyl)-3,5-difluorophenyl)-1*H*-1,2,3-triazol-1-yl)methyl)-6-(((2*R*,3*R*,4*S*,5*S*,6*R*)-3,4,5-trihydroxy-6-(hydroxymethyl)tetrahydro-2*H*-pyran-2-yl)oxy)tetrahydro-2*H*-pyran-3,4,5-triol (17)

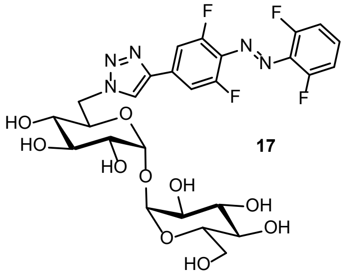

17

PSS_{430nm}(81% *trans*):
^1H NMR (400 MHz, CD_3CN, drop of D_2O) δ 8.42 (d, *J* = 1.2 Hz, 1H), 7.72 – 7.60 (m, 2H), 7.56 – 7.43 (m, 1H), 7.18 (ddd, *J* = 9.7, 8.5, 1.3 Hz, 2H), 5.03 (d, *J* = 3.7 Hz, 1H), 4.80 – 4.72 (m, 1H), 4.70 (d, *J* = 3.9 Hz, 1H), 4.67 – 4.56 (m, 1H), 4.16 (t, *J* = 8.4 Hz, 1H), 3.83 – 3.74 (m, 2H), 3.73 – 3.64 (m, 4H), 3.58 – 3.50 (m, 1H), 3.26 – 3.10 (m, 3H). Part of the signals are overlapping with the water signal.
^{19}F NMR (376 MHz, CD_3CN, drop of D_2O) δ -121.27 (d, *J* = 11.1 Hz), -123.03 (dd, *J* = 10.3, 6.0 Hz).
$^{13}C\{^1H\}$ NMR (101 MHz, CD_3CN, drop of D_2O) δ 158.1 (d, *J* = 4.9 Hz), 157.5 (d, *J* = 4.1 Hz), 155.5 (d, *J* = 5.0 Hz), 154.9 (d, *J* = 4.1 Hz), 145.3, 135.8 (t, *J* = 11.4 Hz), 133.6 (t, *J* = 10.7 Hz), 132.4 – 131.9 (m), 125.4, 113.8 (dd, *J* = 20.5, 3.4 Hz), 110.5 (d, *J* = 23.9 Hz), 94.3, 94.2, 73.7, 73.5, 73.5, 72.1 (d, *J* = 1.8 Hz), 72.0, 71.8 (d, *J* = 2.0 Hz), 70.9, 70.8, 61.9, 52.0.

Spectroscopic data for the *cis* isomer (in a 86 % *cis* isomer PSS mixture):
^1H NMR (400 MHz, CD_3CN, drop of D_2O) δ 8.30 (d, *J* = 1.4 Hz, 1H), 7.51 (d, *J* = 9.5 Hz, 2H), 7.45 – 7.34 (m, 1H), 7.04 (t, *J* = 8.7 Hz, 2H), 5.02 (d, *J* = 3.8 Hz, 1H), 4.81 – 4.66 (m, 2H), 4.56 (ddd, *J* = 14.5, 7.6, 1.7 Hz, 1H), 4.16 (ddd, *J* = 10.1, 7.5, 2.6 Hz, 1H), 3.86 – 3.64 (m, 5H), 3.54 (dd, *J* = 12.5, 6.4 Hz, 1H), 3.41 (ddd, *J* = 9.7, 3.8, 1.7 Hz, 1H), 3.34 – 3.25 (m, 1H), 3.24 – 3.09 (m, 2H).

HRMS (ESI+) m/z: [M + H]+ Calcd for $C_{26}H_{27}F_4N_5O_{10}$+H 646.1767; Found: 646.1754.
Mp. decomposition at 174-180 °C.

3. ^{1}H, ^{19}F and ^{13}C NMR spectra

1,3-difluoro-2-nitrosobenzene (19)[2]

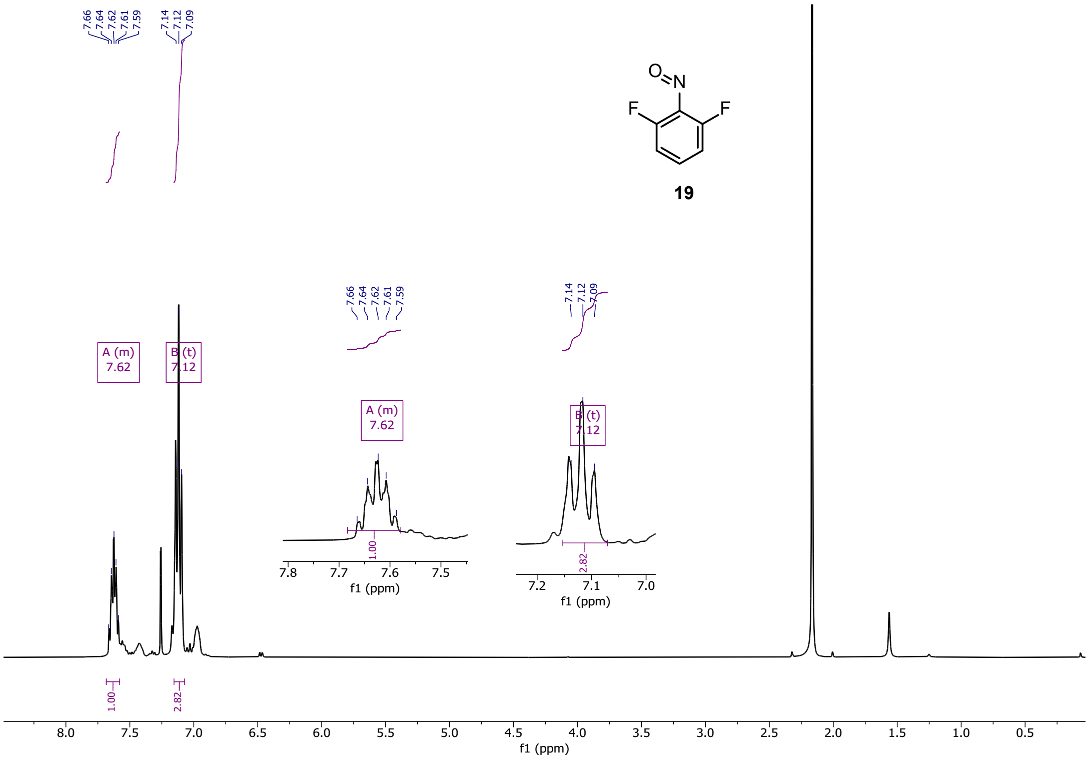

Figure S1. ^{1}H NMR (400 MHz) of compound **19** in $CDCl_3$.

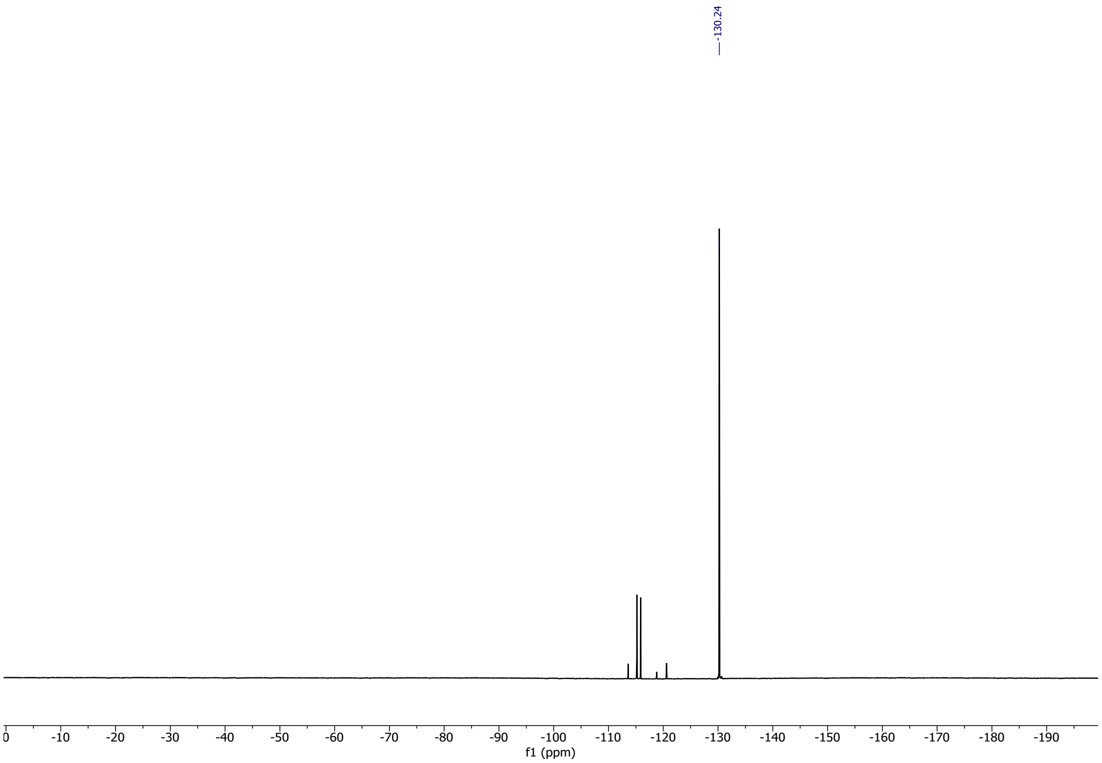

Figure S2. ^{19}F NMR (376 MHz) of compound **19** in $CDCl_3$.

***trans*-1,2-bis(2,6-difluorophenyl)diazene (1)**[3,4]

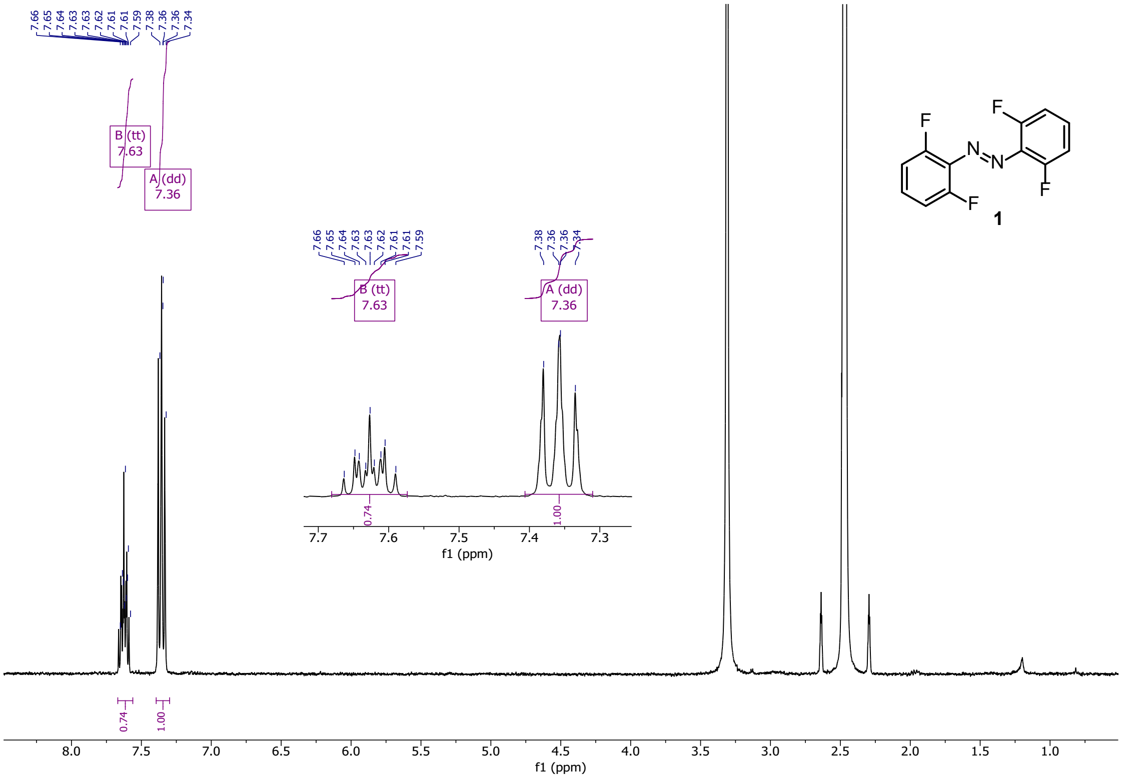

Figure S3. ^{1}H NMR (400 MHz) of compound **1** in DMSO-d_6.

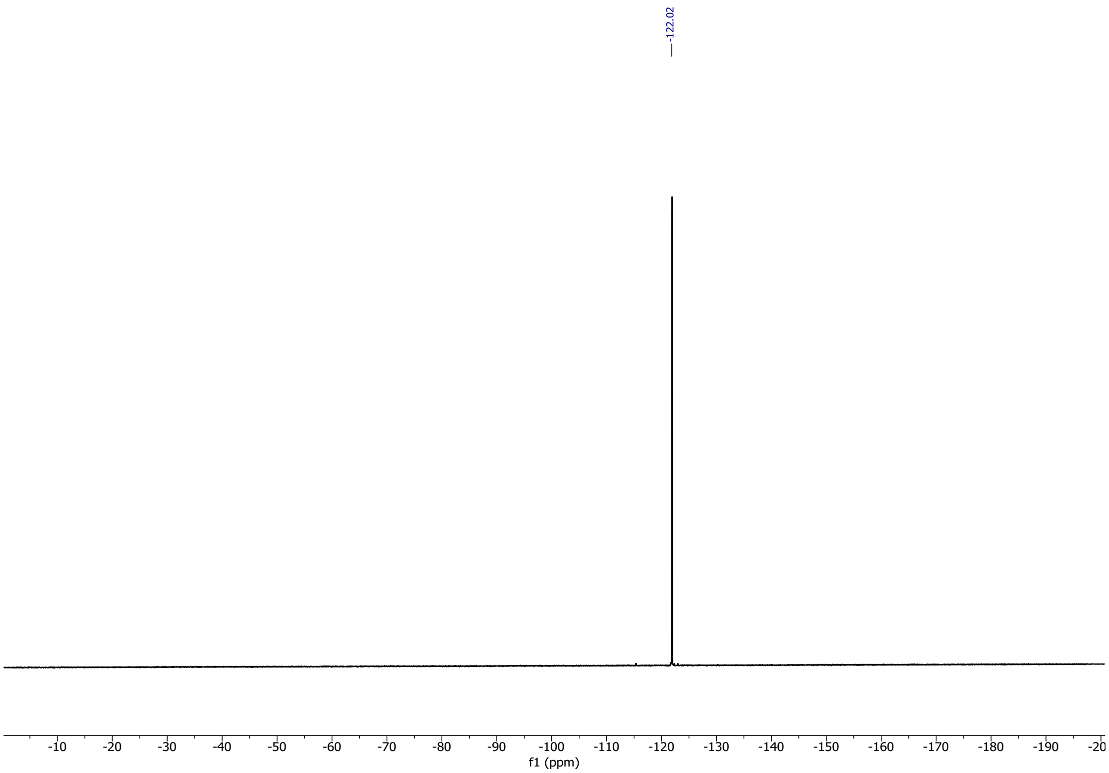

Figure S4. ^{19}F NMR (376 MHz) of compound **1** in DMSO-d_6.

***trans*-1-(4-bromo-2,6-difluorophenyl)-2-(2,6-difluorophenyl)diazene (3)**[5,6]

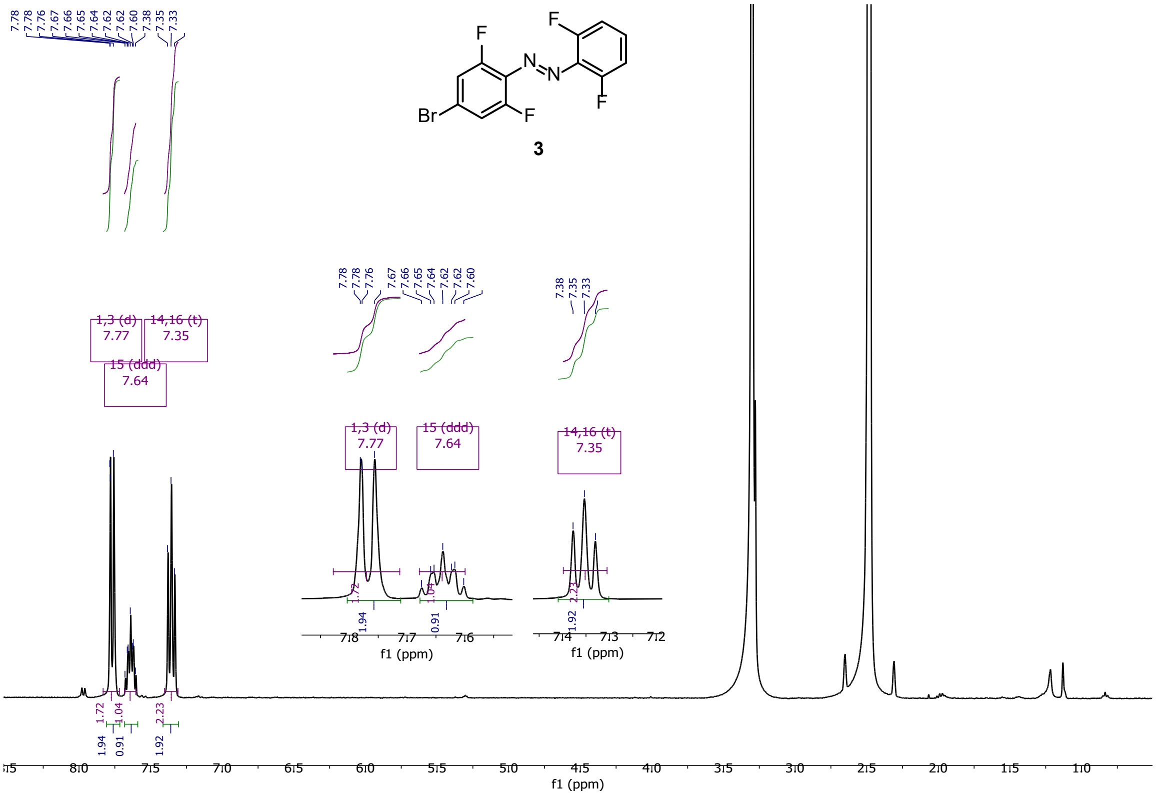

Figure S5. ^{1}H NMR (400 MHz) of compound **3** in DMSO-d_6.

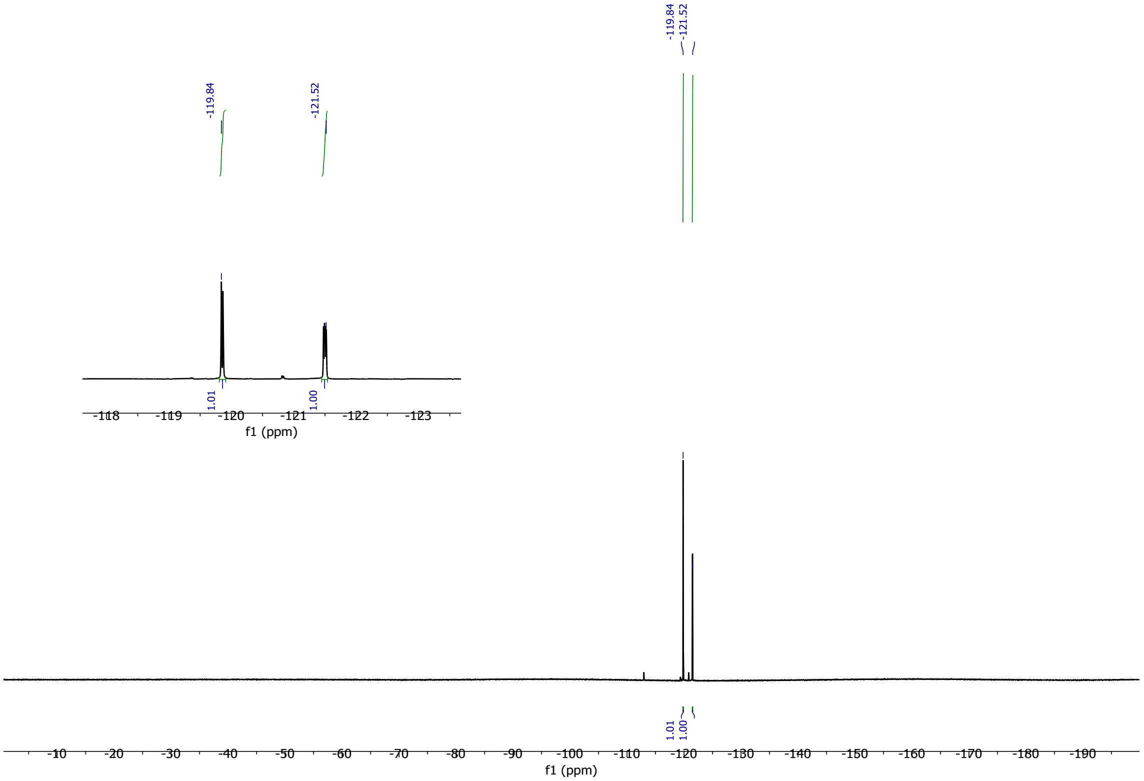

Figure S6. ^{19}F NMR (376 MHz) of compound **3** in DMSO-d_6.

5-bromo-1,3-difluoro-2-nitrosobenzene (22)[7]

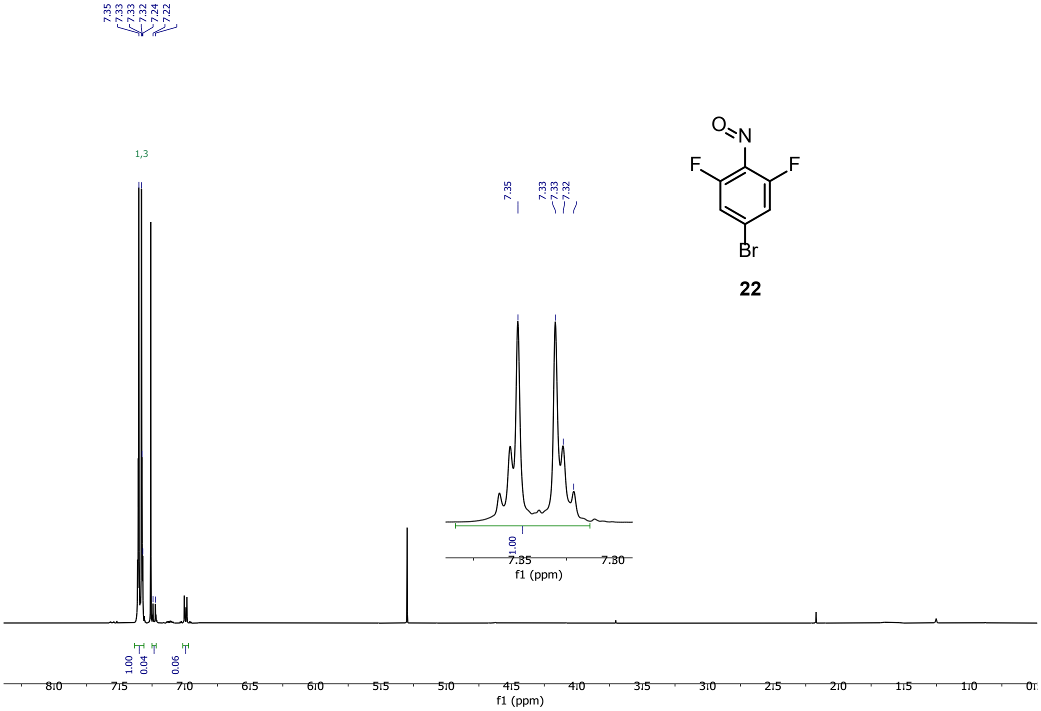

Figure S7. ^{1}H NMR (400 MHz) of compound **22** in $CDCl_3$.

***trans*-1,2-bis(4-bromo-2,6-difluorophenyl)diazene (6)**[8,9]

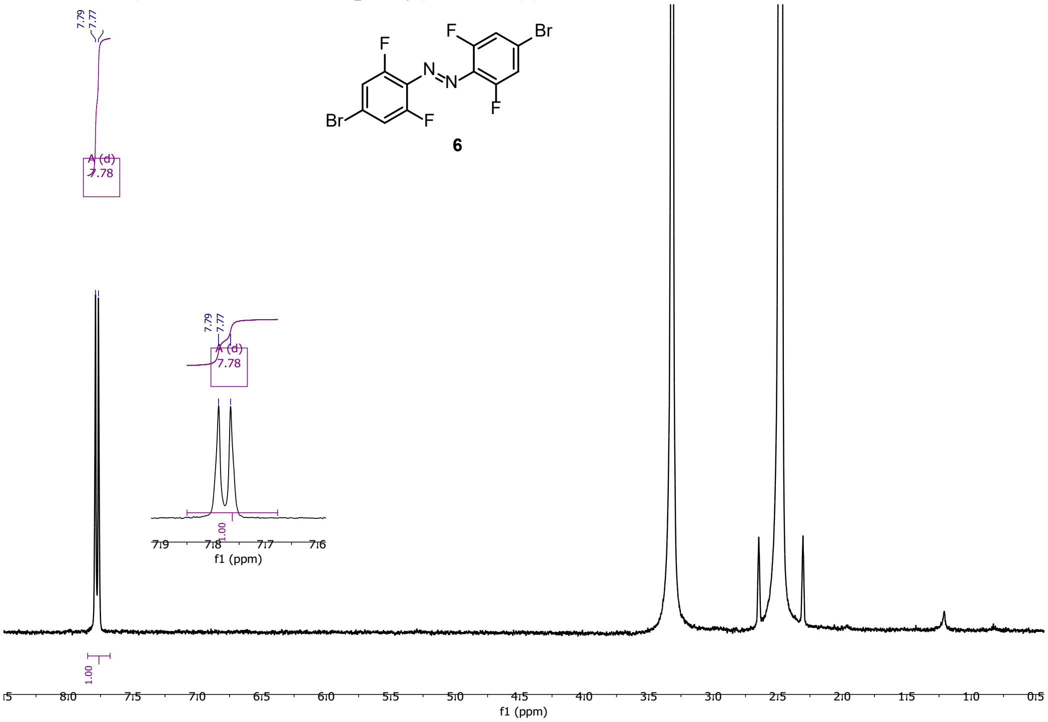

Figure S8. ^{1}H NMR (400 MHz) of compound **3** in DMSO-d_6.

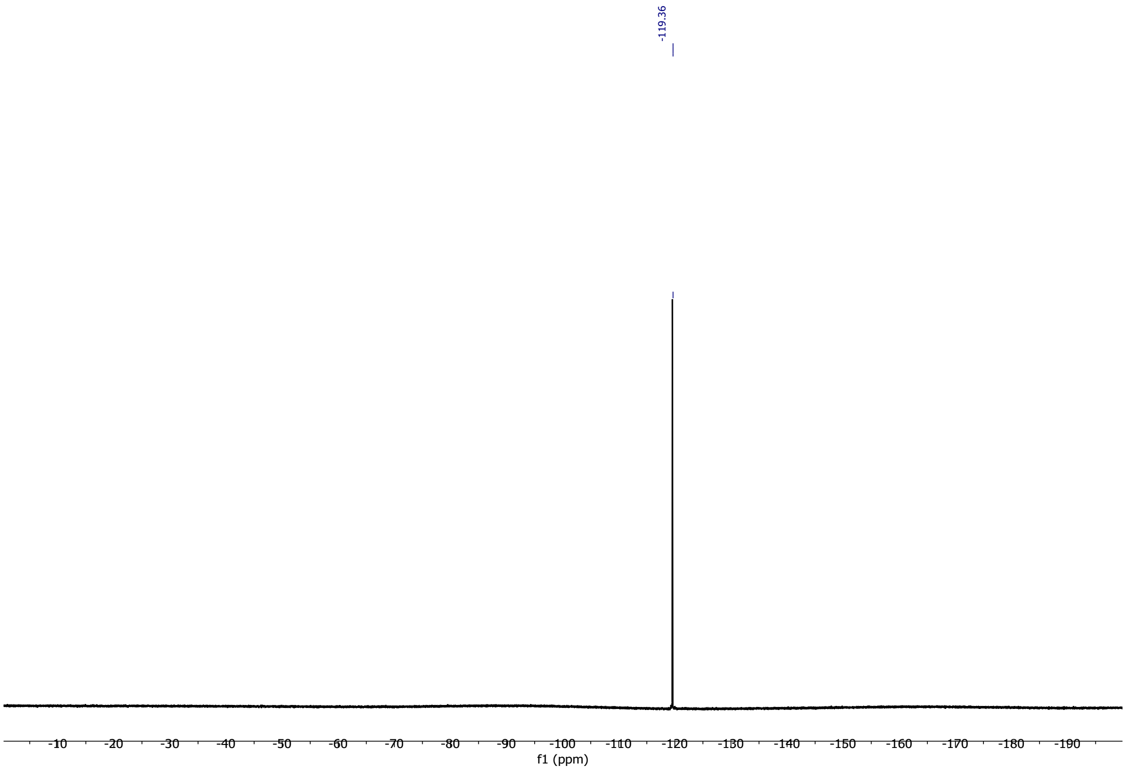

Figure S9. ^{19}F NMR (376 MHz) of compound **3** in DMSO-d_6.

(*trans*)-1-(2,6-difluorophenyl)-2-(2,4,6-trifluorophenyl)diazenediazene (2)

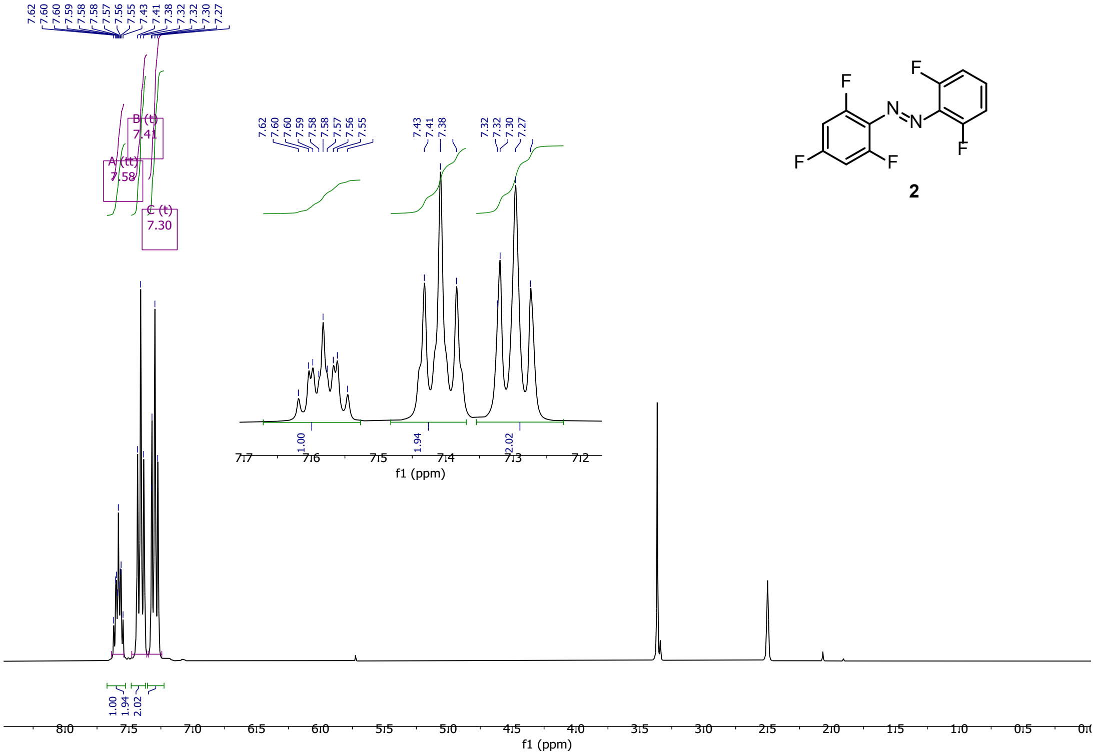

Figure S10. ^{1}H NMR (400 MHz) of compound **2** in DMSO-d_6.

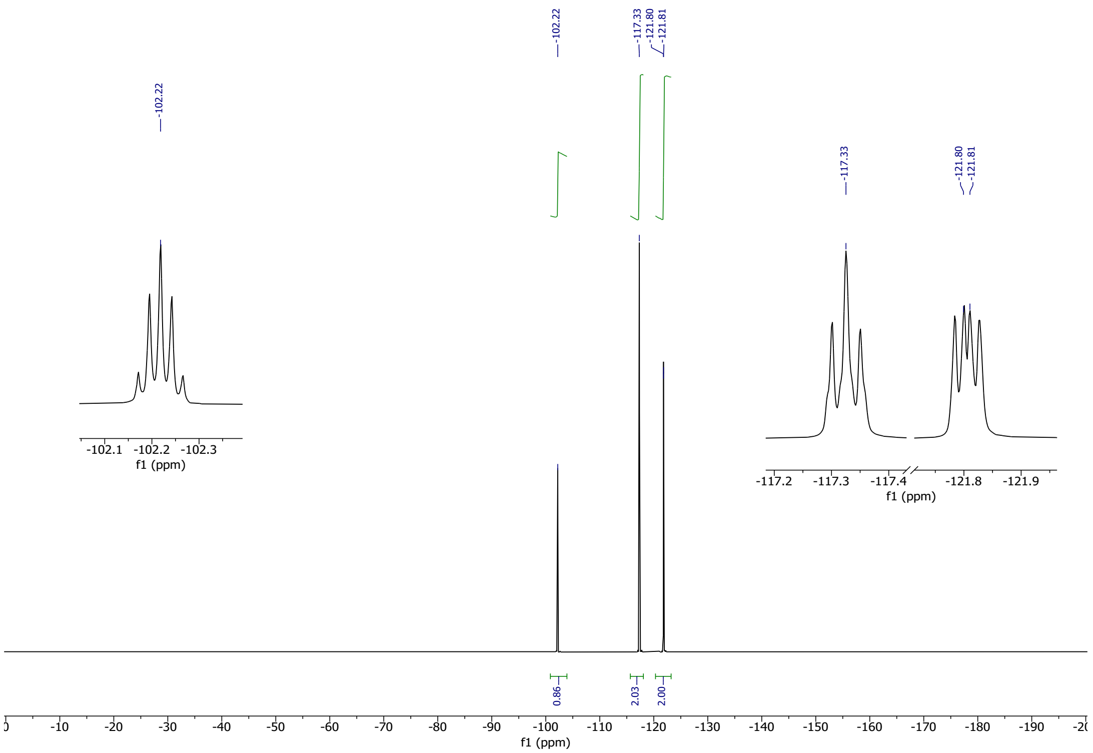

Figure S11. ^{19}F NMR (376 MHz) of compound **2** in DMSO-d_6.

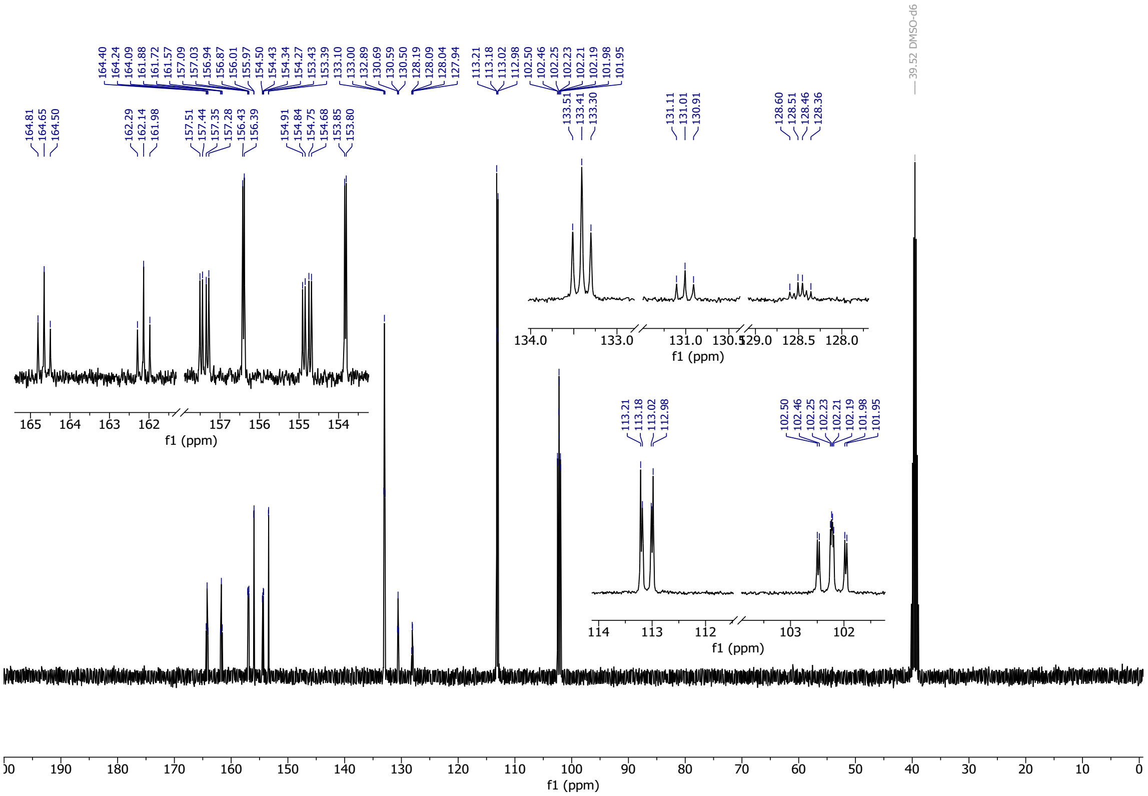

Figure S12. $^{13}C\{^{1}H\}$ NMR (101 MHz) of compound **2** in DMSO-d_6.

(*trans*)-4-((2,6-difluorophenyl)diazenyl)-3,5-difluorobenzenesulfonate (4)

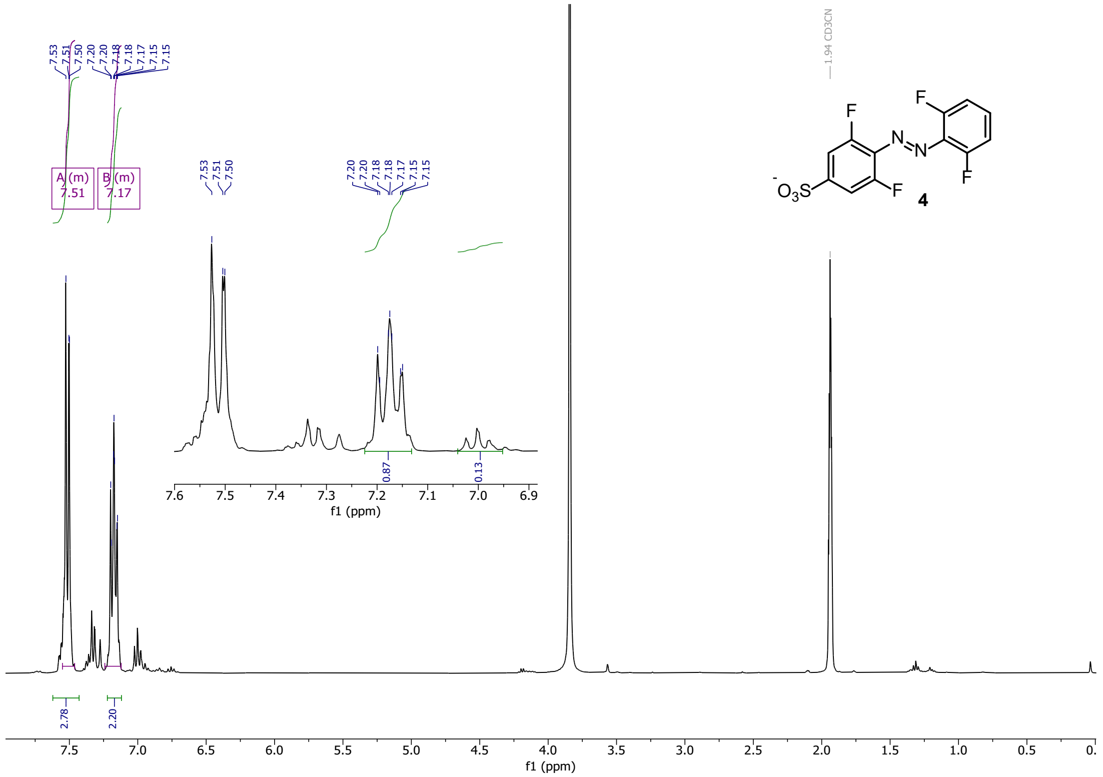

Figure S13. ^{1}H NMR (400 MHz) of compound **4** (87:13=*trans*:*cis*) in CD_3CN with a drop of D_2O. The observed impurities are present despite of challenging at repeated reverse-phase chromatography purification of this highly polar product.

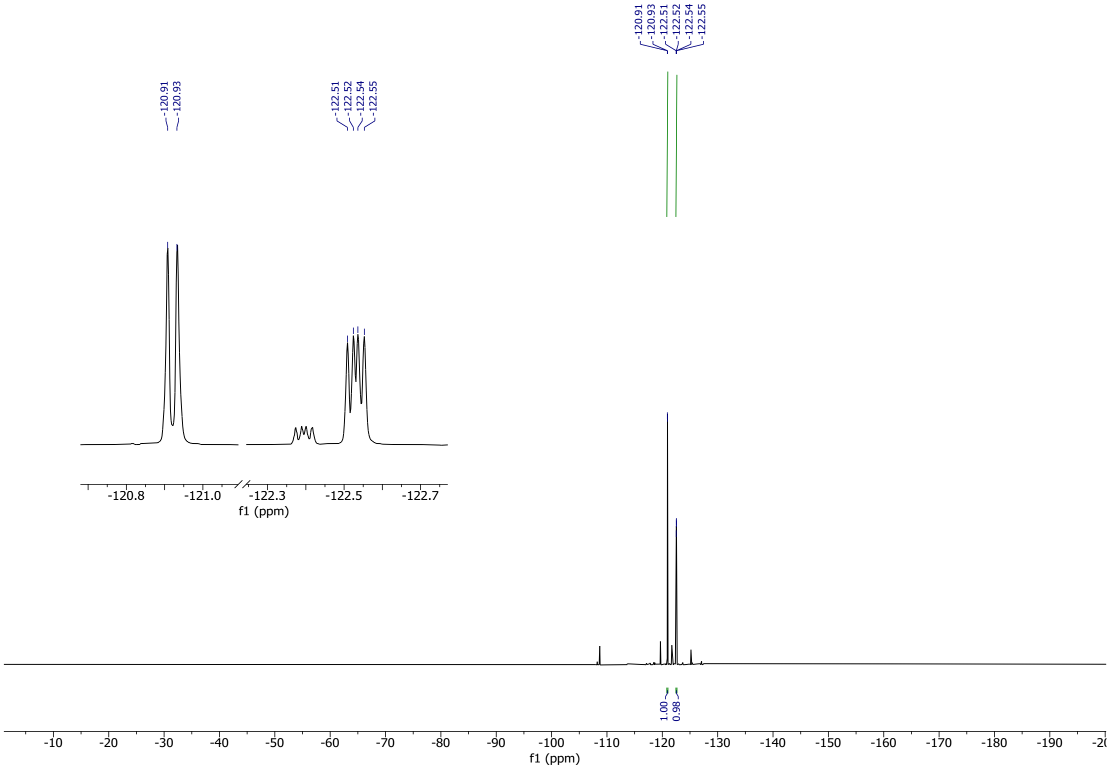

Figure S14. ^{19}F NMR (376 MHz) of compound **4** (87:13=*trans*:*cis*) in CD_3CN with a drop of D_2O. The observed impurities are present despite of challenging at repeated reverse-phase chromatography purification of this highly polar product.

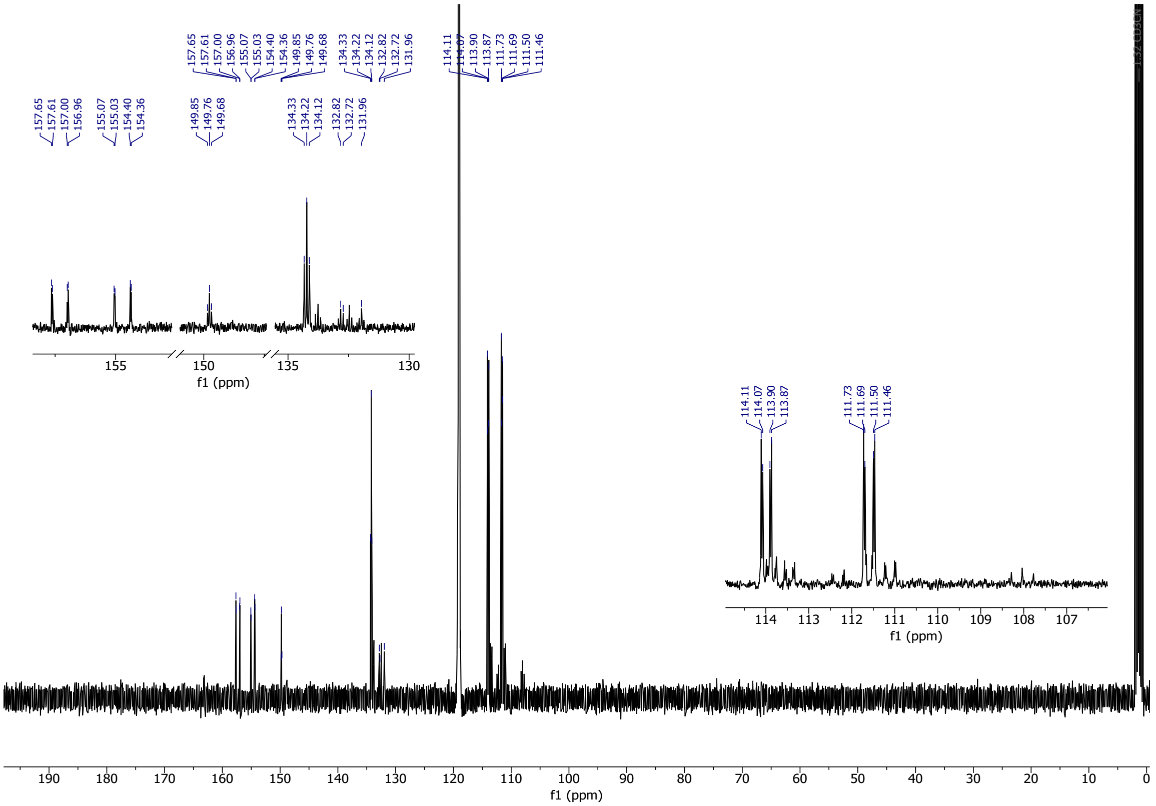

Figure S15. $^{13}C\{^1H\}$ NMR (101 MHz) of compound **4** (87:13=*trans*:*cis*) in CD_3CN with a drop of D_2O. The observed impurities are present despite of challenging at repeated reverse-phase chromatography purification of this highly polar product.

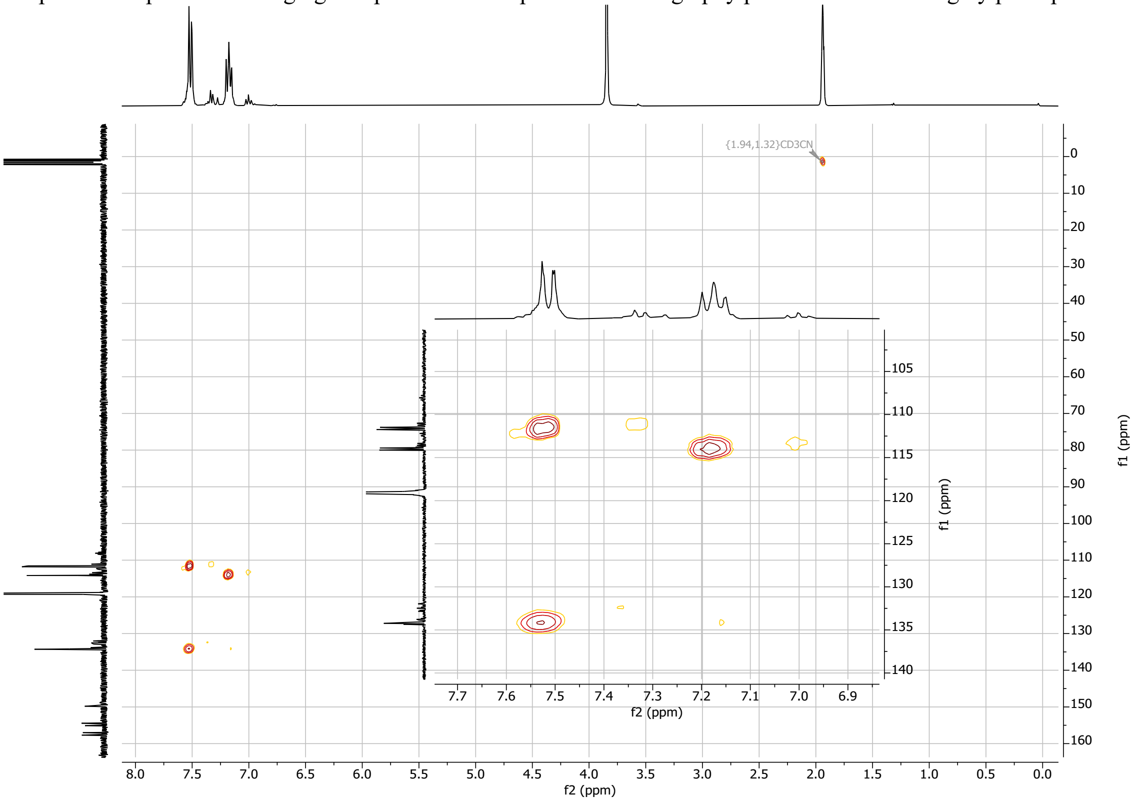

Figure S16. HSQC 2D NMR (400 MHz) of compound **4** (87:13=*trans*:*cis*) in CD_3CN with a drop of D_2O. The observed impurities are present despite of challenging at repeated reverse-phase chromatography purification of this highly polar product.

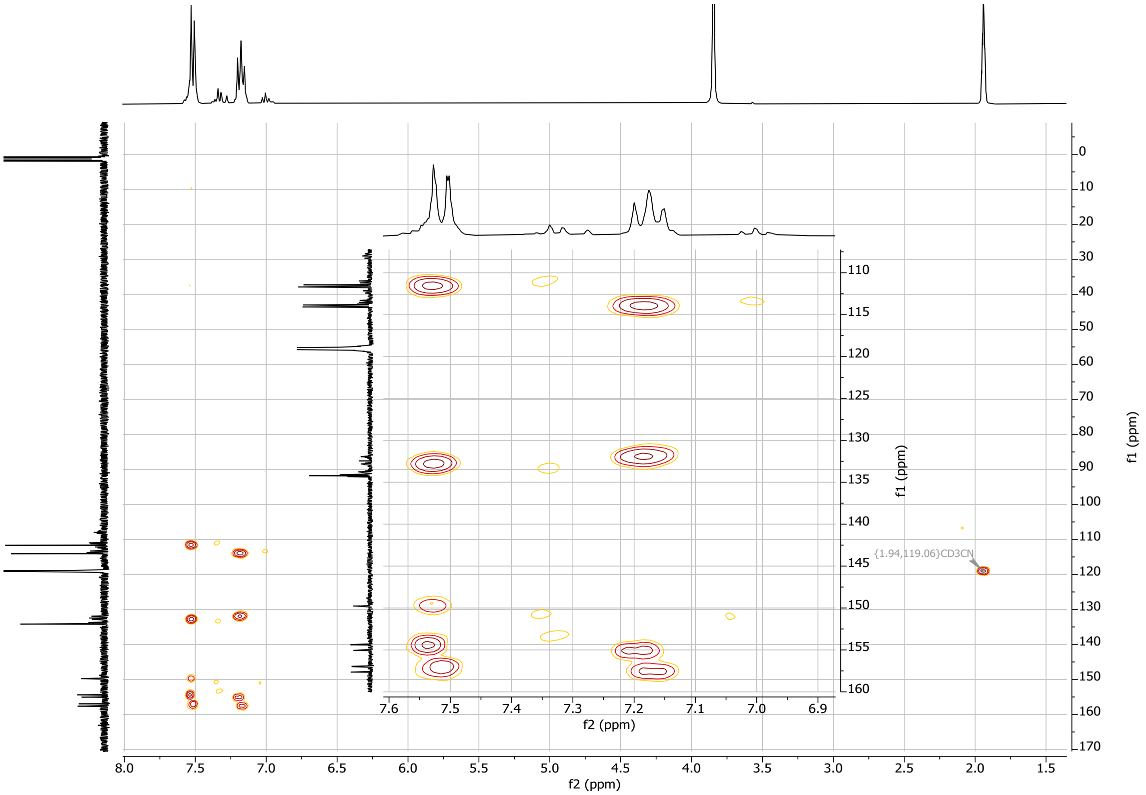

Figure S17. HMBC 2D NMR (400 MHz) of compound **4** (87:13=*trans*:*cis*) in CD_3CN with a drop of D_2O. The observed impurities are present despite of challenging at repeated reverse-phase chromatography purification of this highly polar product.

(*trans*)-1-(2,6-difluoro-4-((trimethylsilyl)ethynyl)phenyl)-2-(2,6-difluorophenyl)diazene (14)

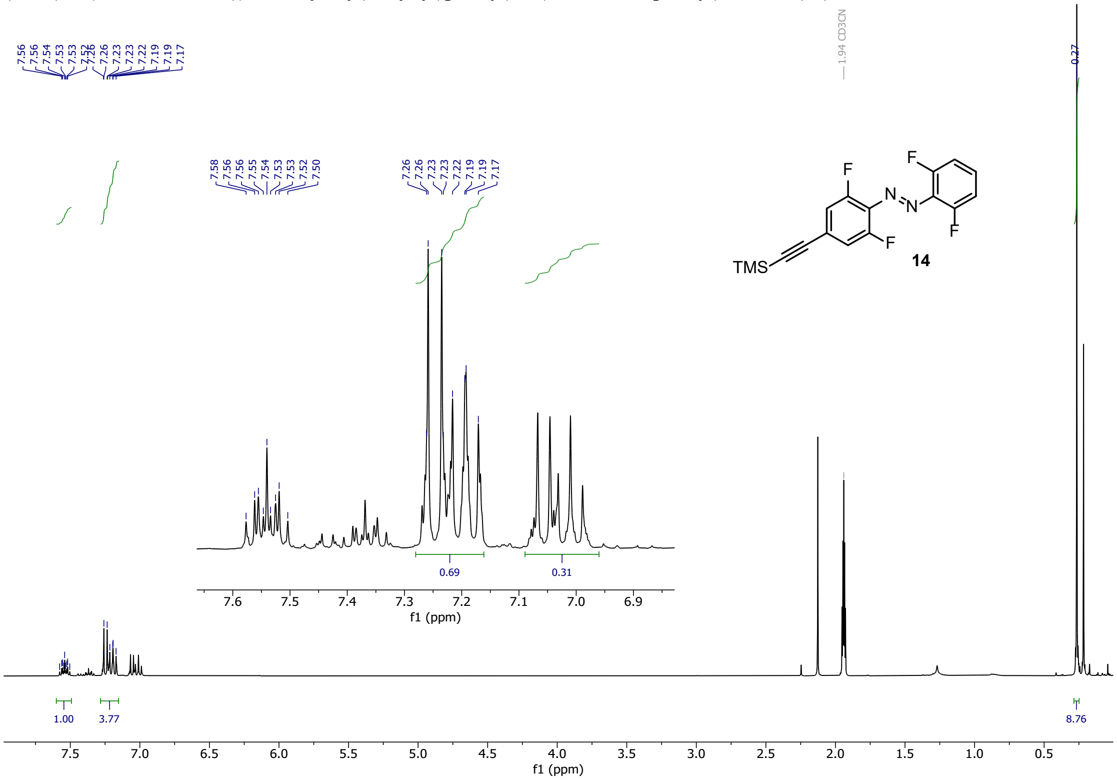

Figure S18. ^{1}H NMR (400 MHz) of compound **14** (69:31=*trans*:*cis*) in CD_3CN.

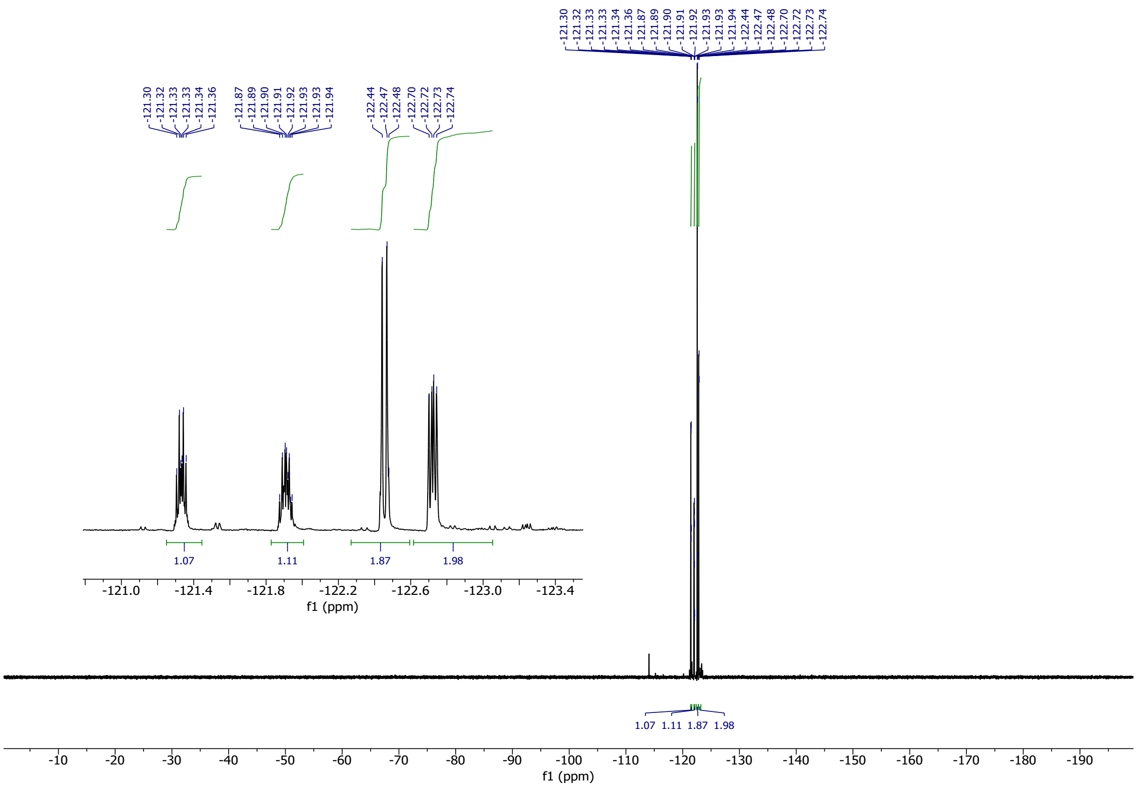

Figure S19. ^{19}F NMR (376 MHz) of compound **14** (87:31=*trans*:*cis*) in CD_3CN.

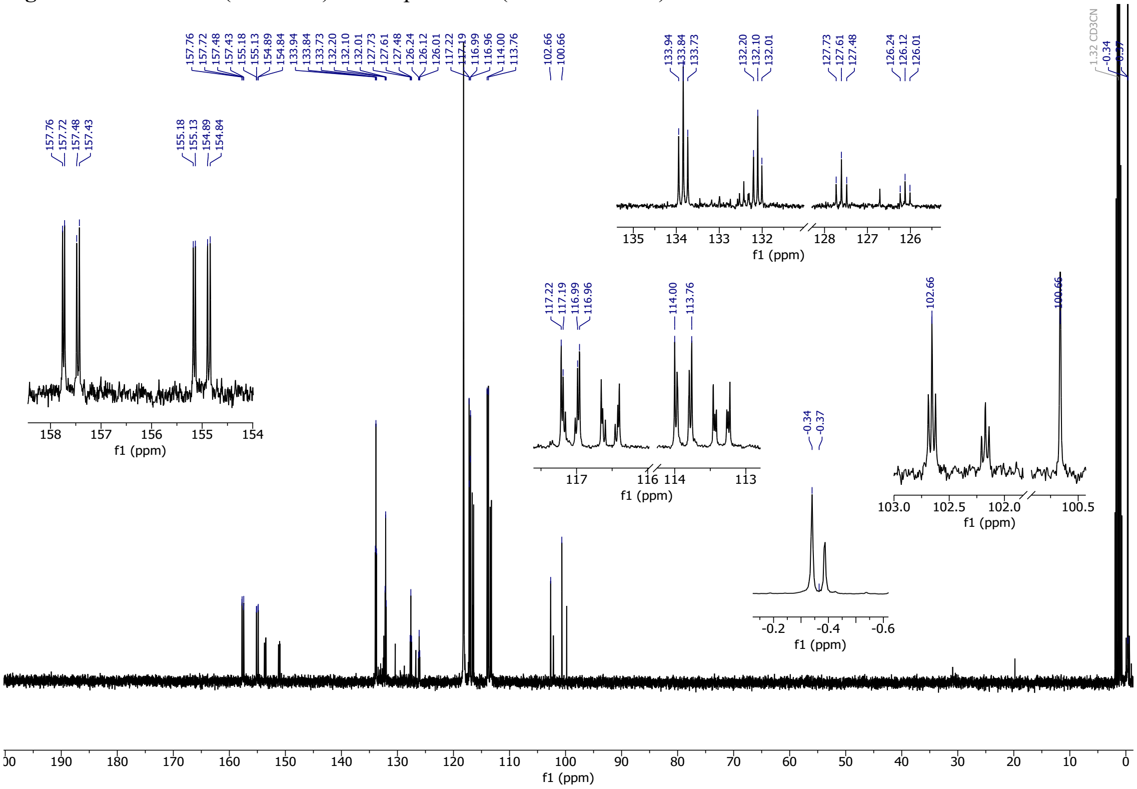

Figure S20. $^{13}C\{^{1}H\}$ NMR (101 MHz) of compound **14** (87:31=*trans*:*cis*) in CD_3CN.

(*trans*)-1-(4-ethynyl-2,6-difluorophenyl)-2-(2,4,6-trifluorophenyl)diazene (10)

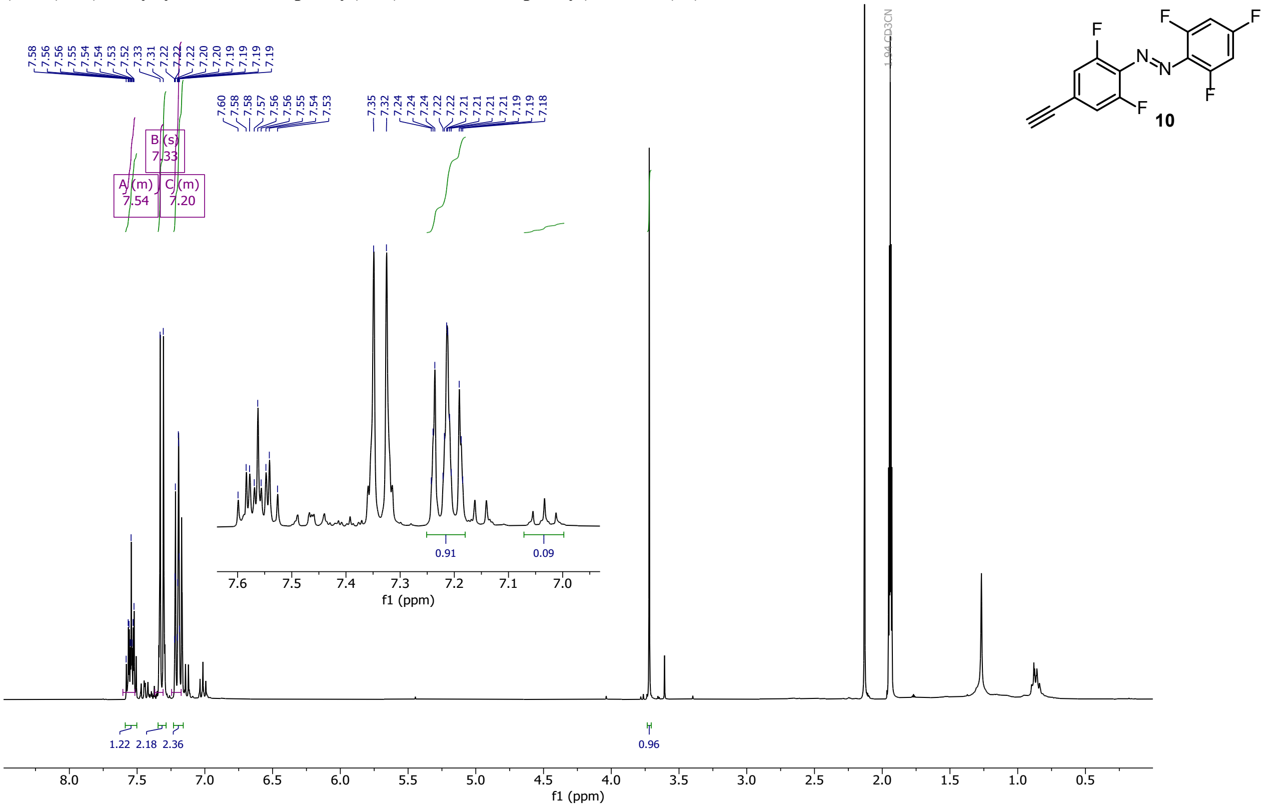

Figure S21. ^{1}H NMR (400 MHz) of compound **10** (91:9=*trans*:*cis*) in CD_3CN.

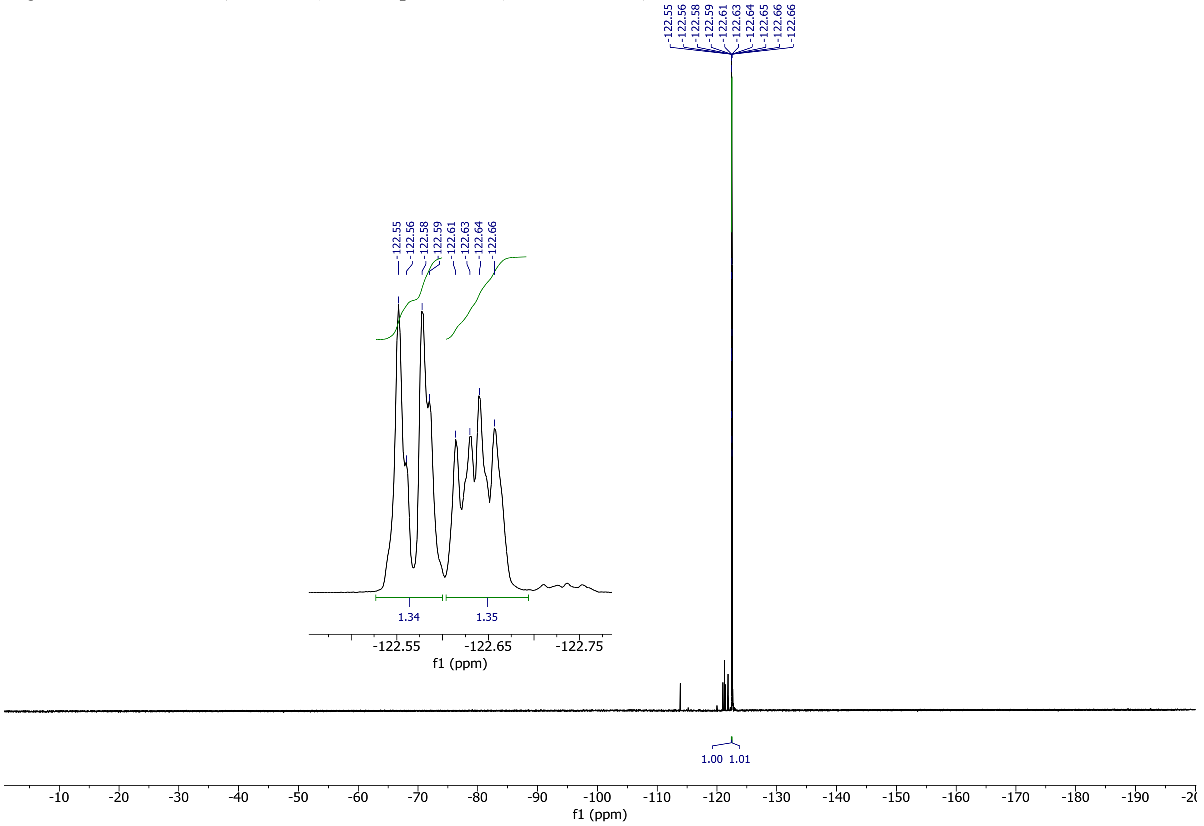

Figure S22. ^{19}F NMR (376 MHz) of compound **10** (91:9=*trans*:*cis*) in CD_3CN.

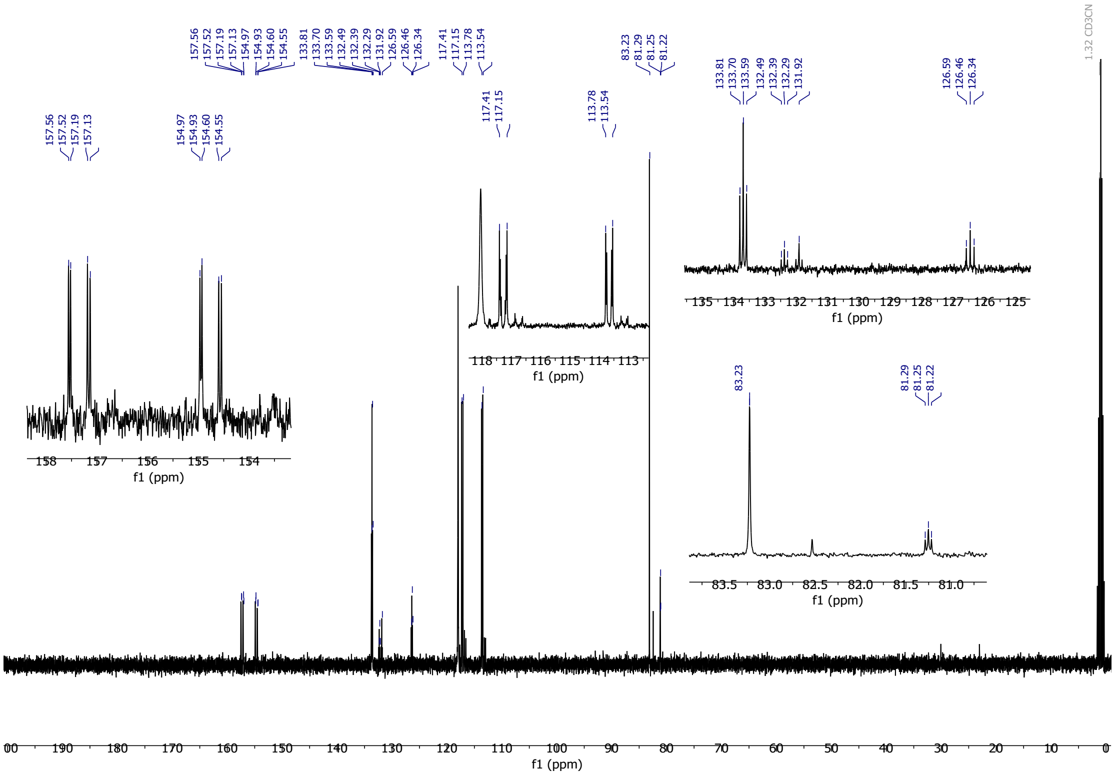

Figure S23. $^{13}C\{^{1}H\}$ NMR (101 MHz) of compound **10** (91:9=*trans*:*cis*) in CD_3CN.

(*trans*)-1-(4-bromo-2,6-difluorophenyl)-2-(2,4,6-trifluorophenyl)diazene (7)

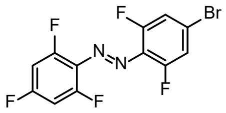

7

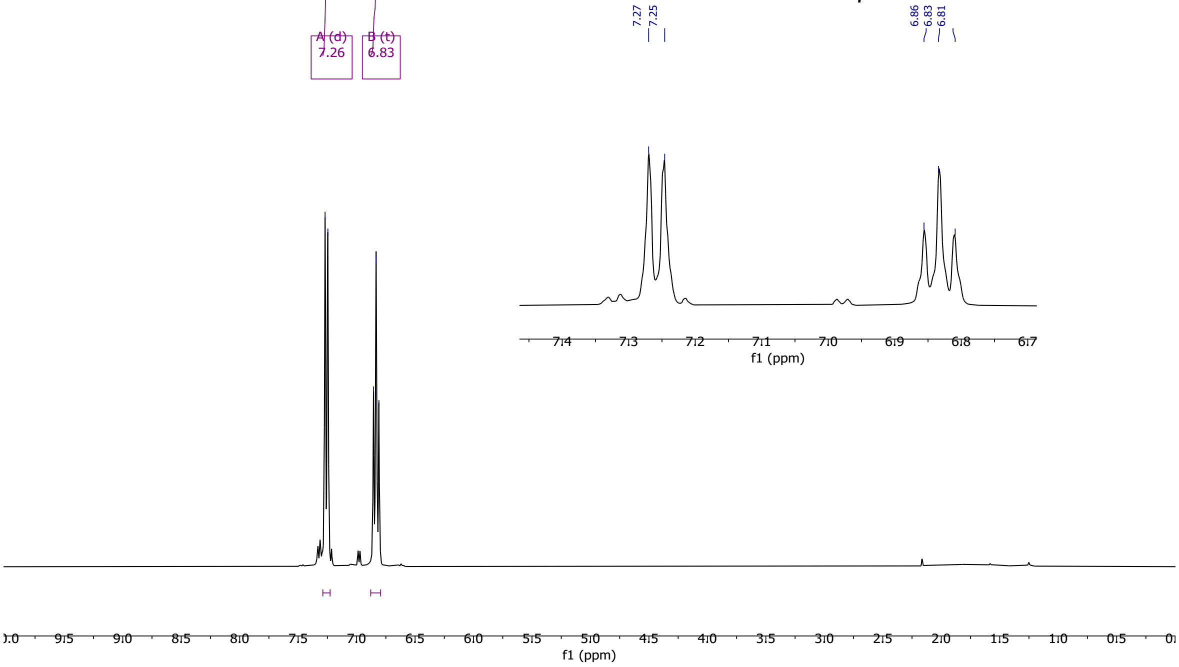

Figure S24. ^{1}H NMR (400 MHz) of compound **7** in $CDCl_3$.

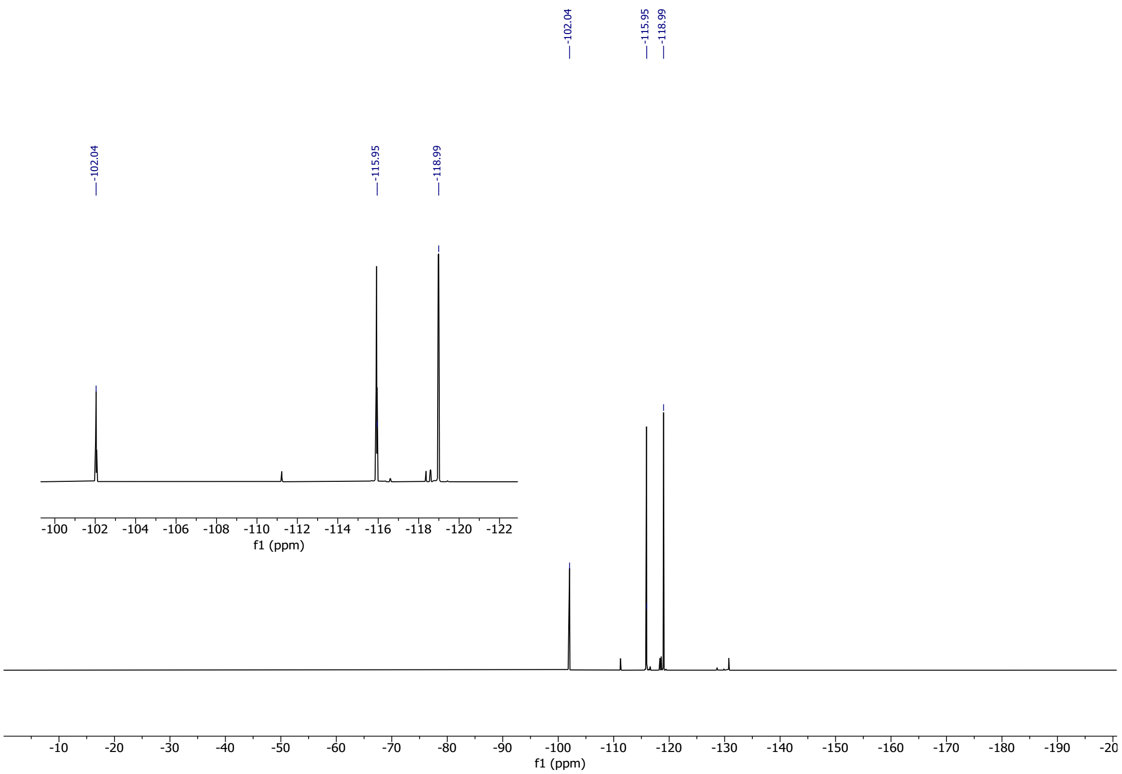

Figure S25. ^{19}F NMR (376 MHz) of compound **7** in $CDCl_3$.

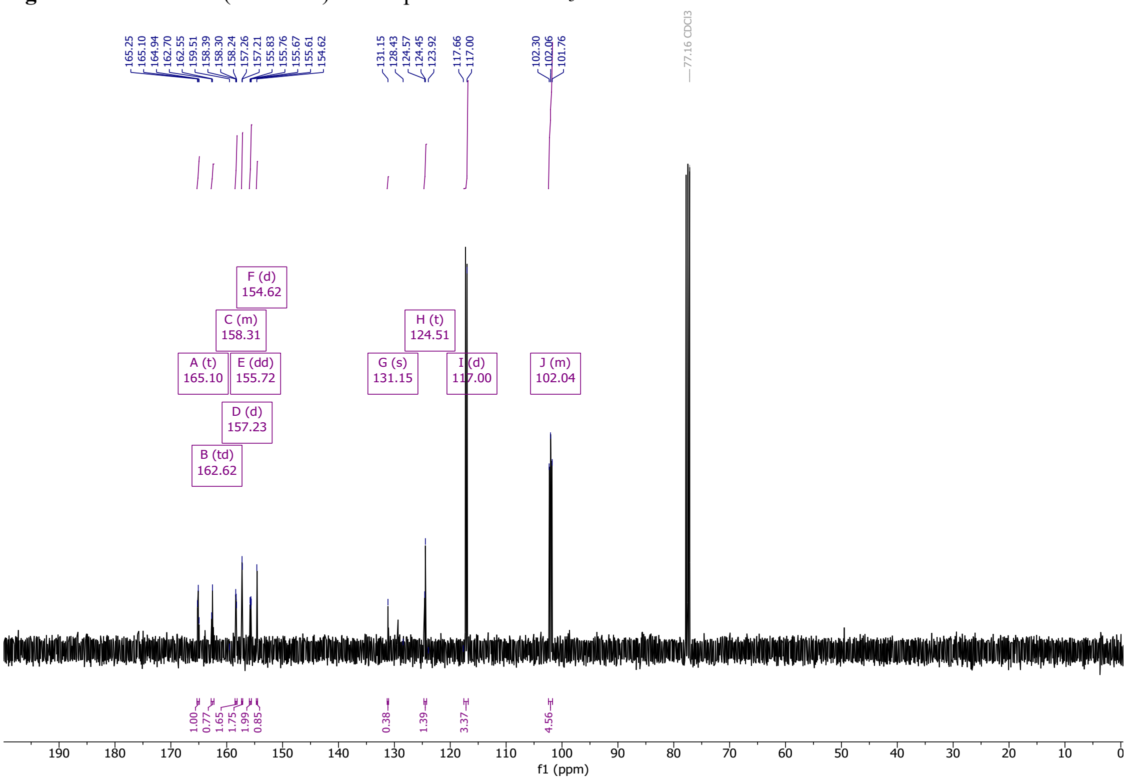

Figure S26. $^{13}C\{^1H\}$ NMR (101 MHz) of compound **7** in $CDCl_3$.

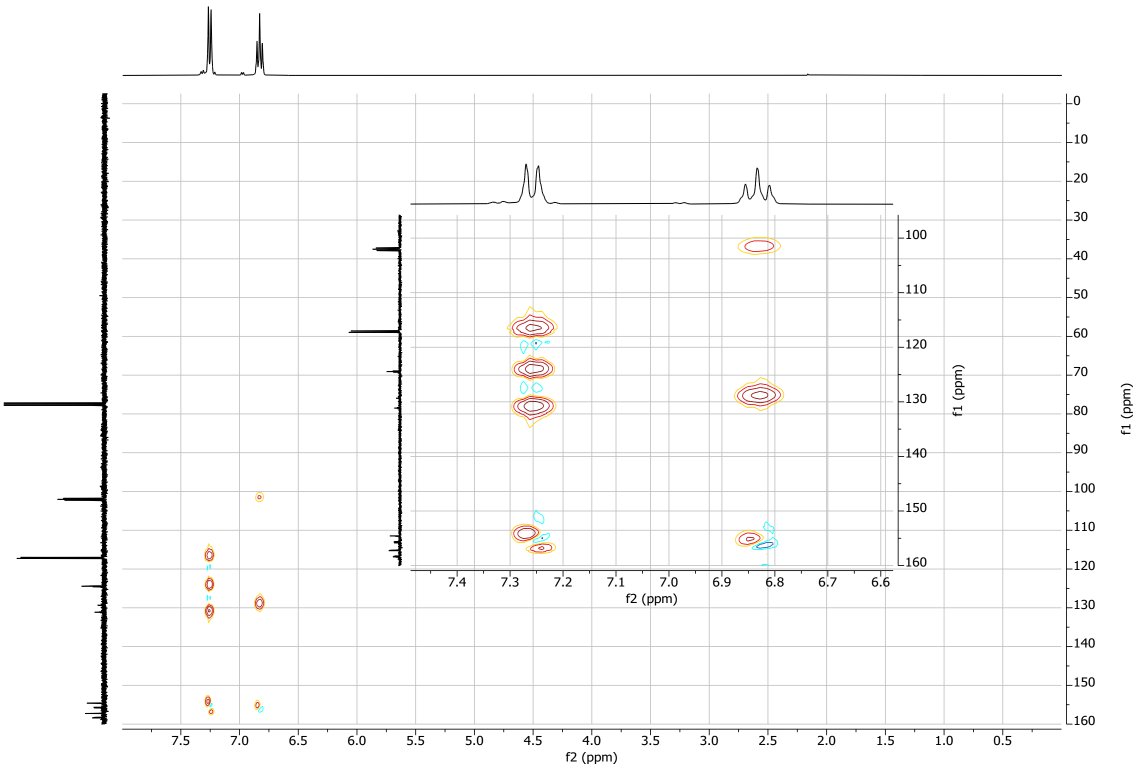

Figure S27. HSQC 2D NMR (400 MHz) of compound **7** in $CDCl_3$.

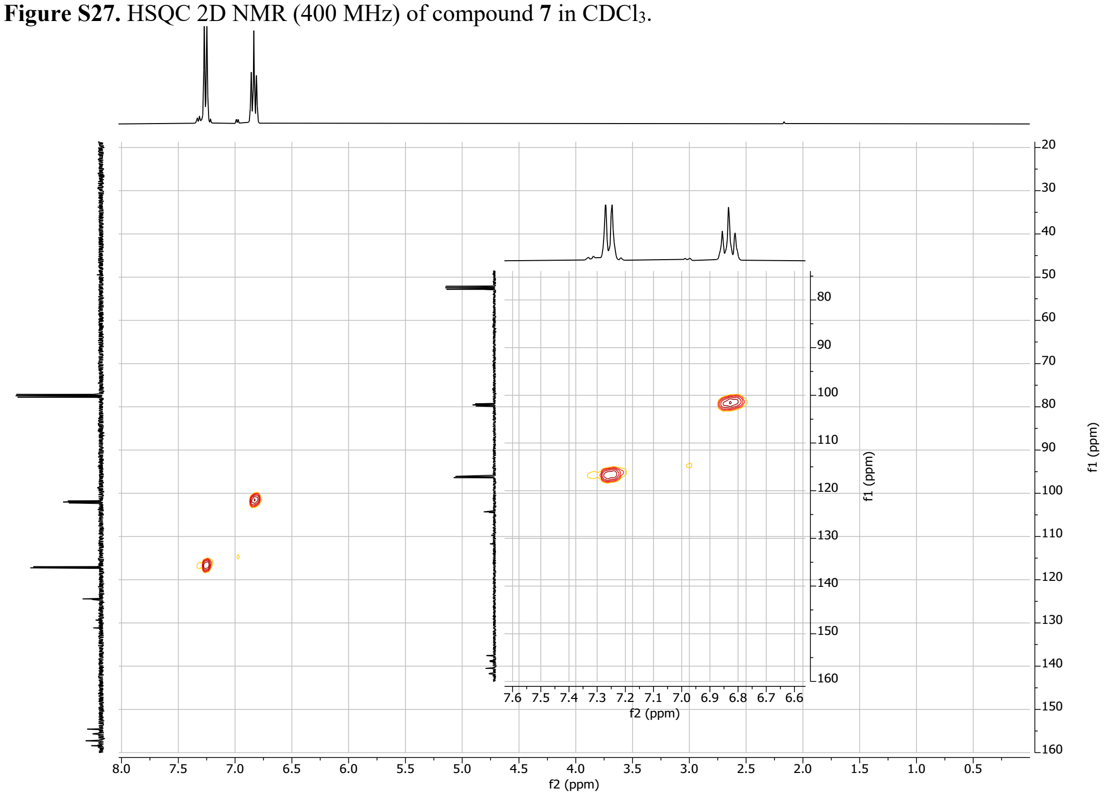

Figure S28. HMBC 2D NMR (400 MHz) of compound **7** in $CDCl_3$.

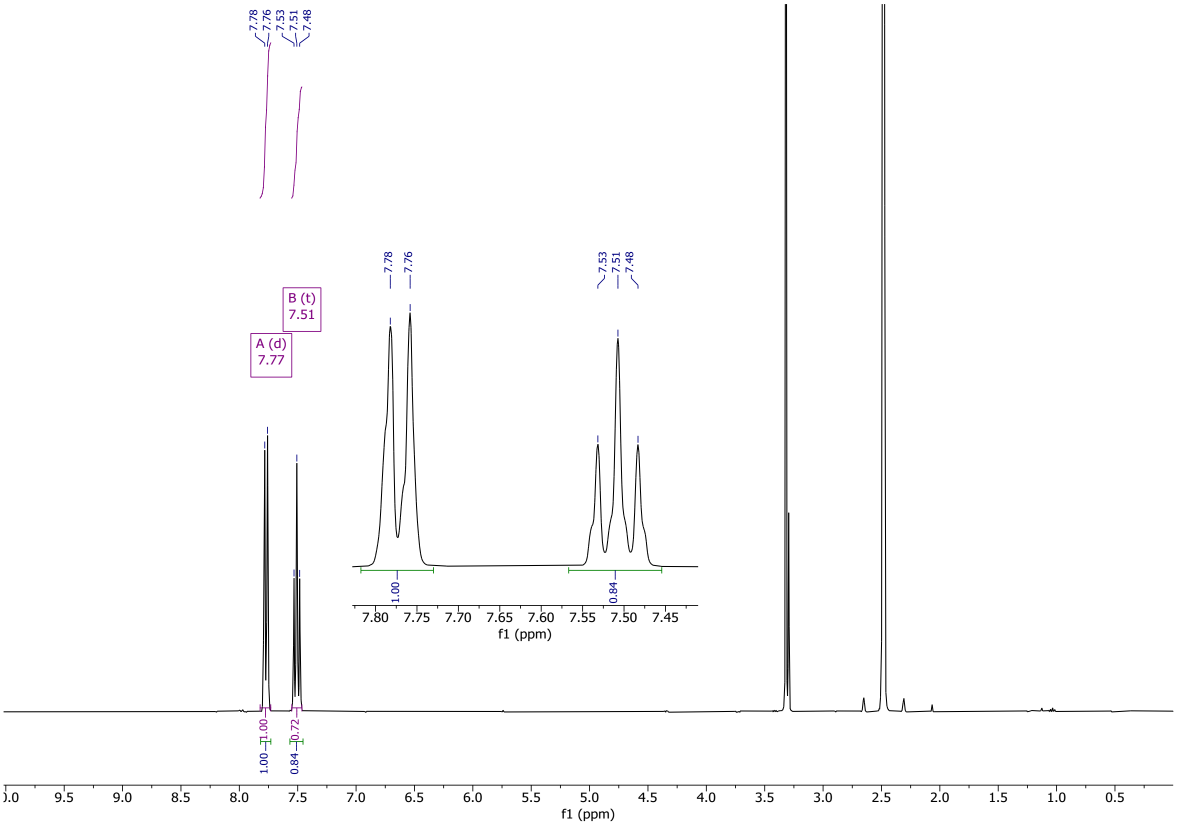

Figure S29. ^{1}H NMR (400 MHz) of compound **7** in DMSO-d_6.

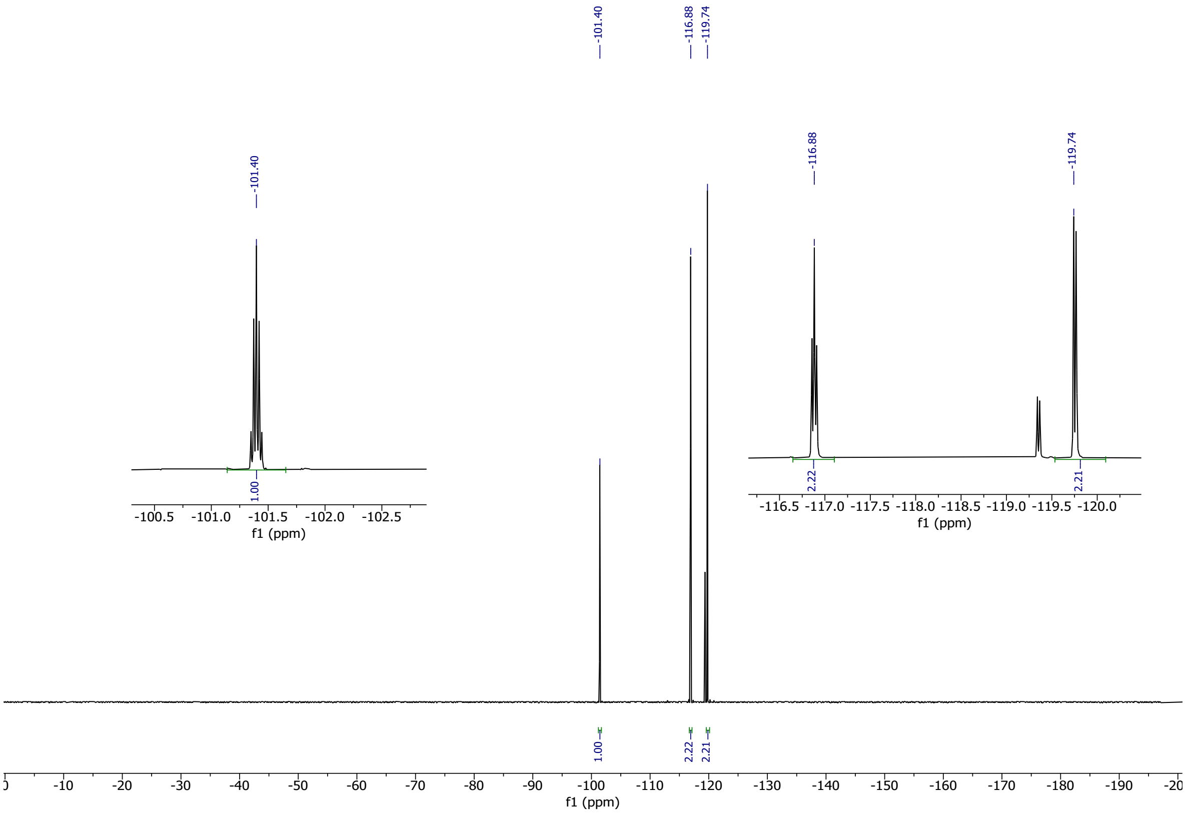

Figure S30. ^{19}F NMR (376 MHz) of compound **7** in DMSO-d_6.

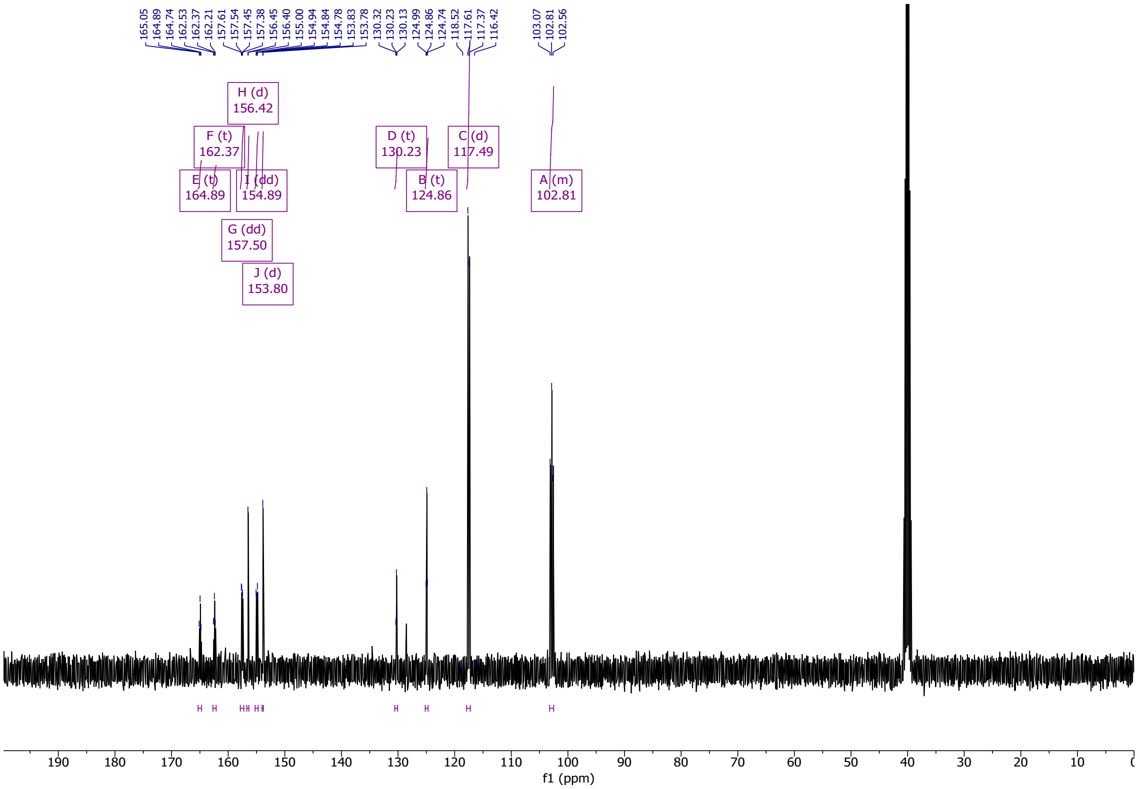

Figure S31. $^{13}C\{^{1}H\}$ NMR (101 MHz) of compound **7** in DMSO-d_6.

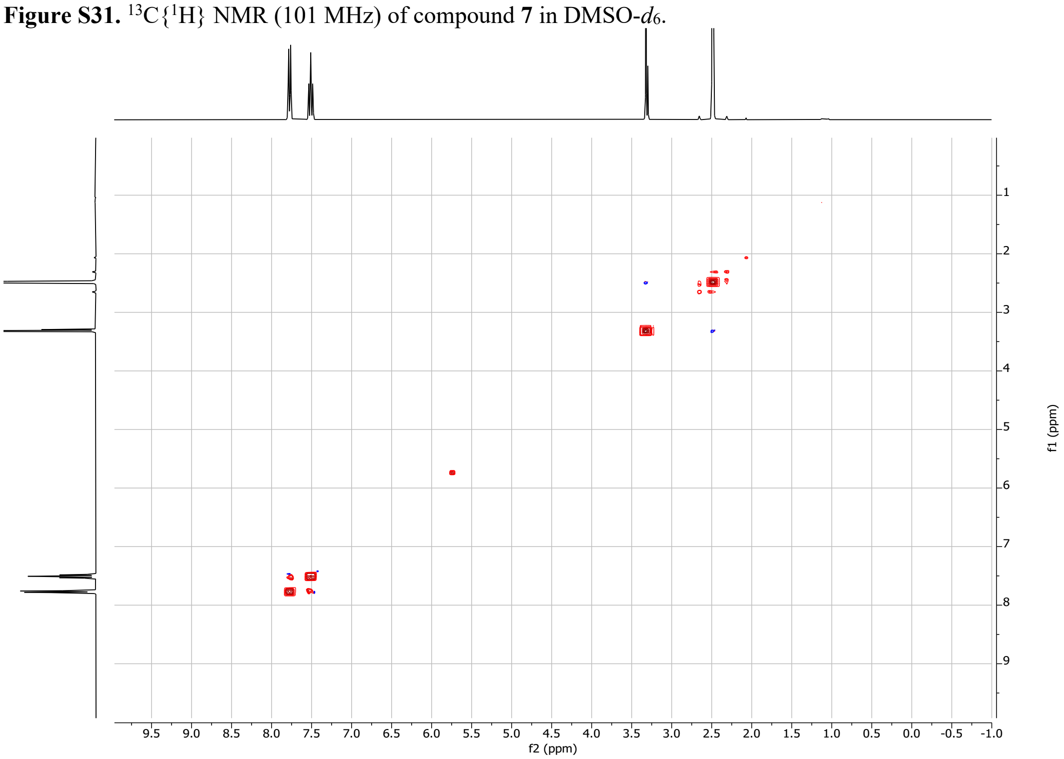

Figure S32. COSY 2D NMR (400 MHz) of compound **7** in DMSO-d_6.

(*trans*)-4-((4-bromo-2,6-difluorophenyl)diazenyl)-3,5-difluorobenzenesulfonate (8)

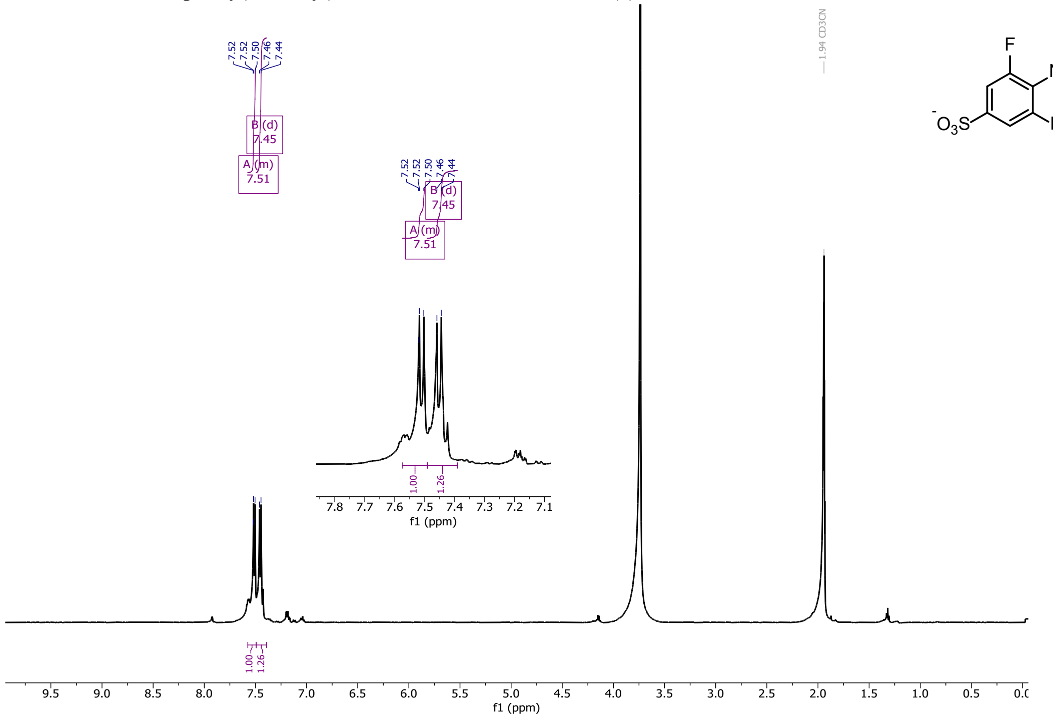

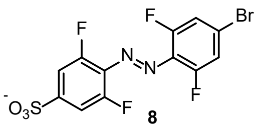

8

Figure S33. ^{1}H NMR (400 MHz) of compound **8** in CD_3CN, drop D_2O. The observed impurities are present despite of challenging at repeated reverse-phase chromatography purification of this highly polar product.

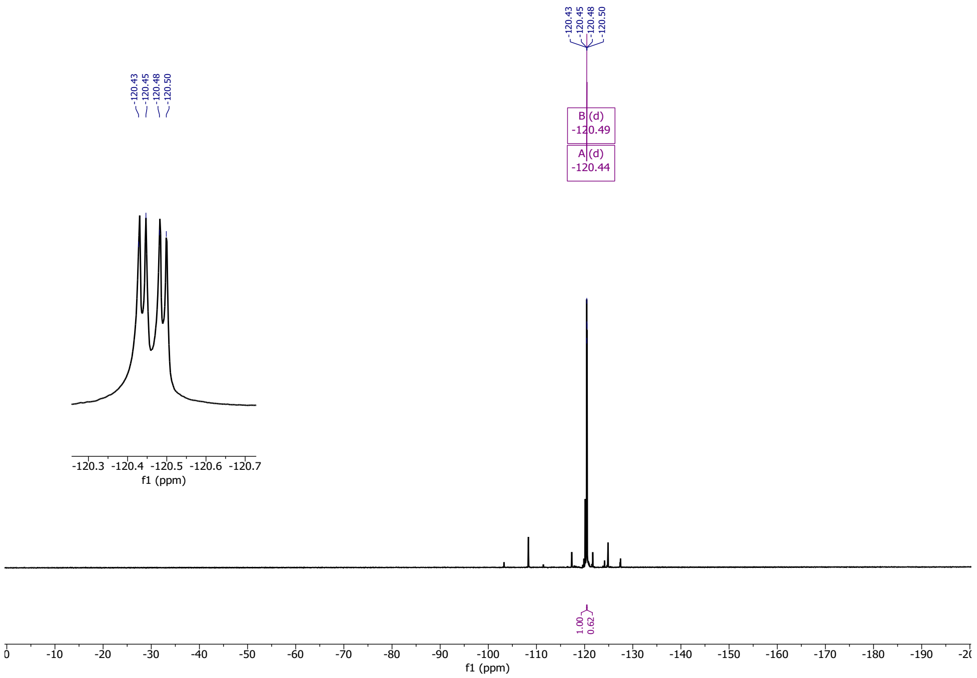

Figure S34. ^{19}F NMR (376 MHz) of compound **8** in CD_3CN, drop D_2O. The observed impurities are present despite of challenging at repeated reverse-phase chromatography purification of this highly polar product.

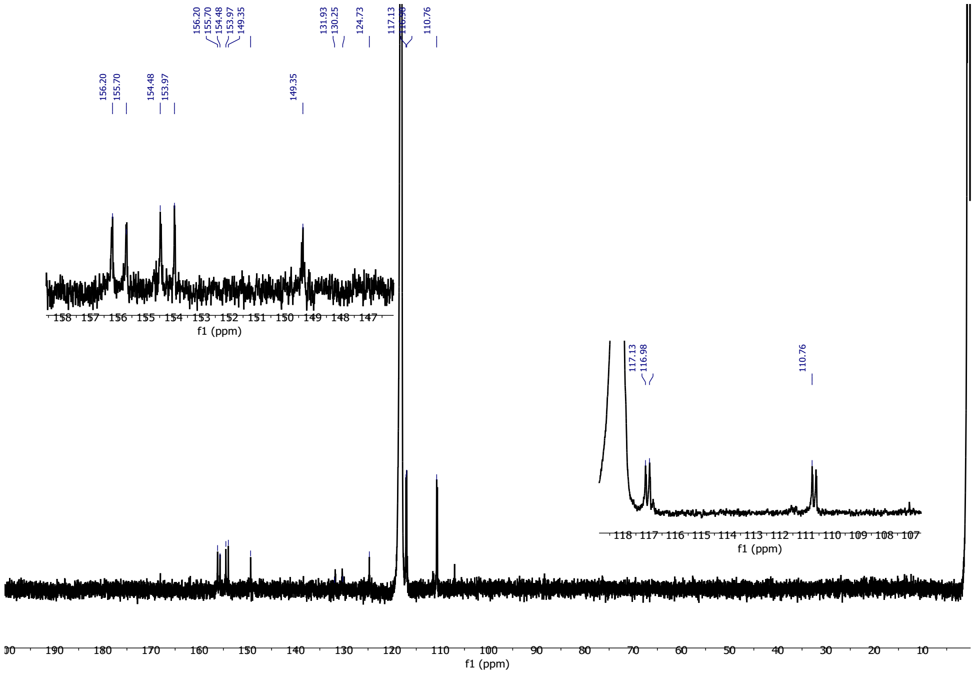

Figure S35. $^{13}C\{^{1}H\}$ NMR (101 MHz) of compound **8** in CD_3CN, drop D_2O. The observed impurities are present despite of challenging at repeated reverse-phase chromatography purification of this highly polar product.

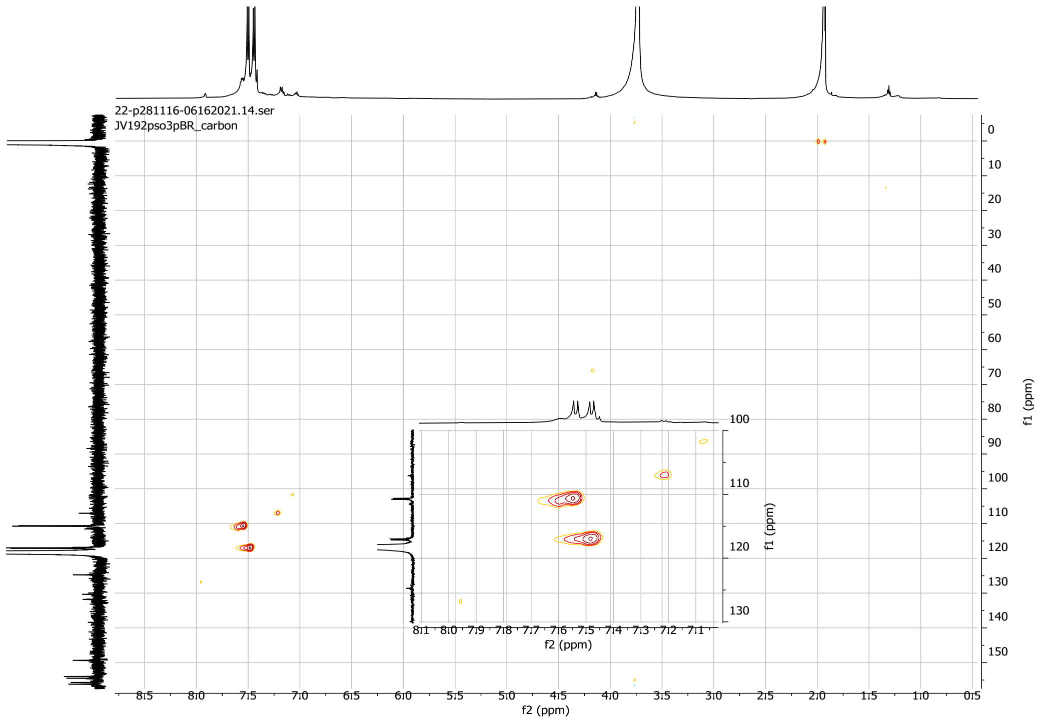

Figure S36. HSQC 2D NMR (400 MHz) of compound **8** in CD_3CN, drop D_2O. The observed impurities are present despite of challenging at repeated reverse-phase chromatography purification of this highly polar product.

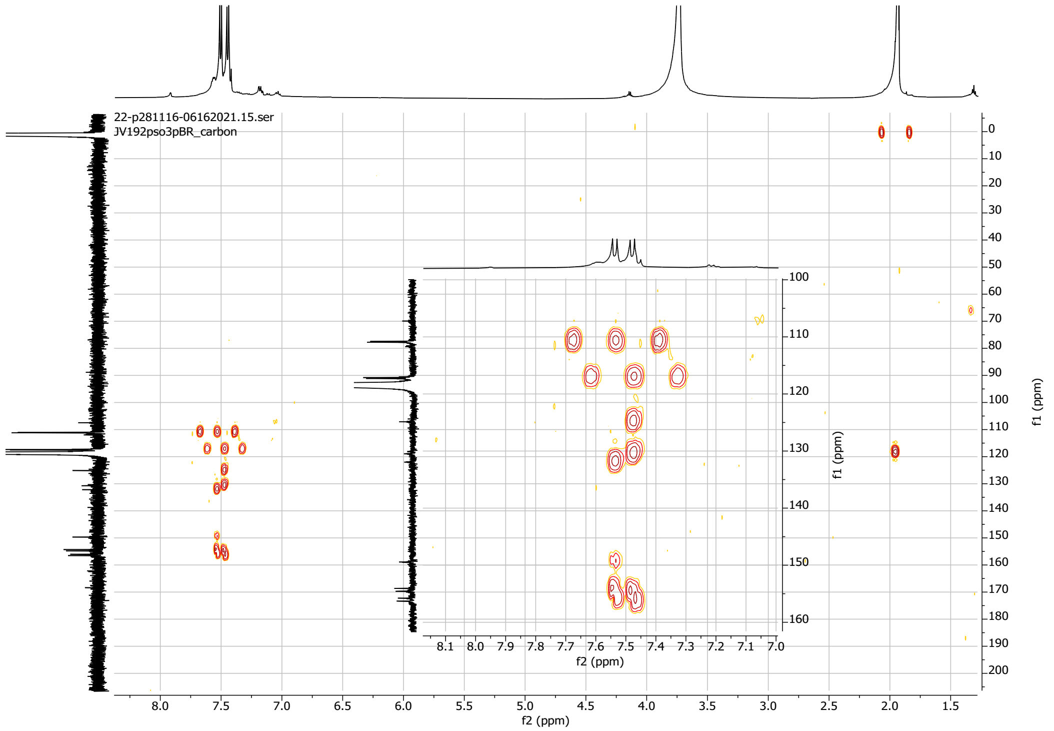

Figure S37. HMBC 2D NMR (400 MHz) of compound **8** in CD_3CN, drop D_2O. The observed impurities are present despite of challenging at repeated reverse-phase chromatography purification of this highly polar product.

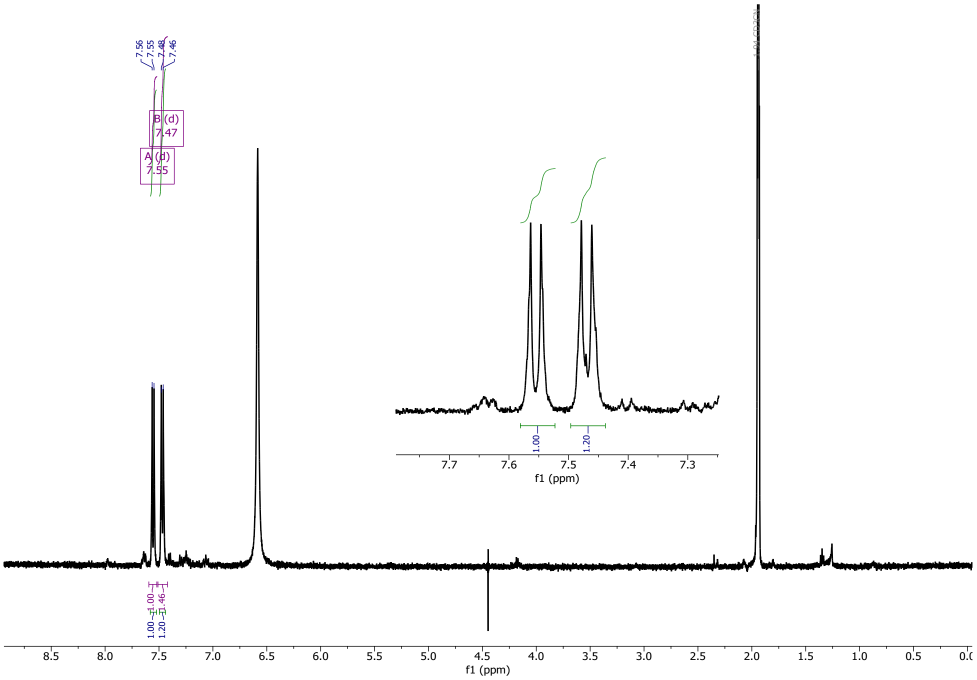

Figure S38. ^{1}H NMR (400 MHz) of compound **8** in CD_3CN with a drop DCl. The observed impurities are present despite of challenging at repeated reverse-phase chromatography purification of this highly polar product.

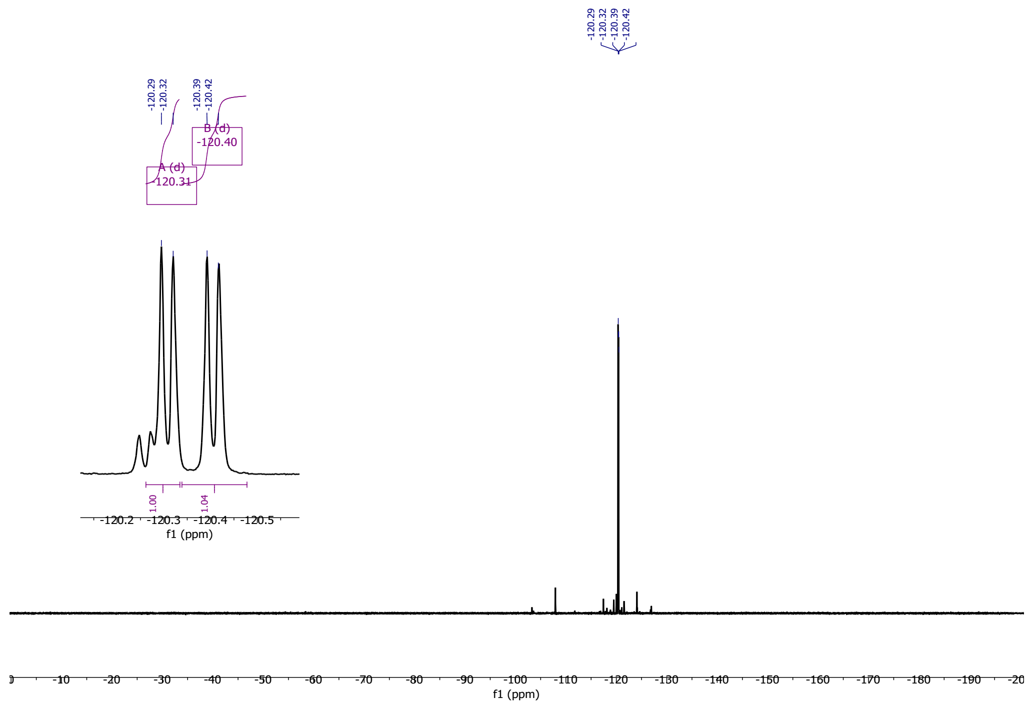

Figure S39. ^{19}F NMR (376 MHz) of compound **8** in CD_3CN with a drop DCl. The observed impurities are present despite of challenging at repeated reverse-phase chromatography purification of this highly polar product.

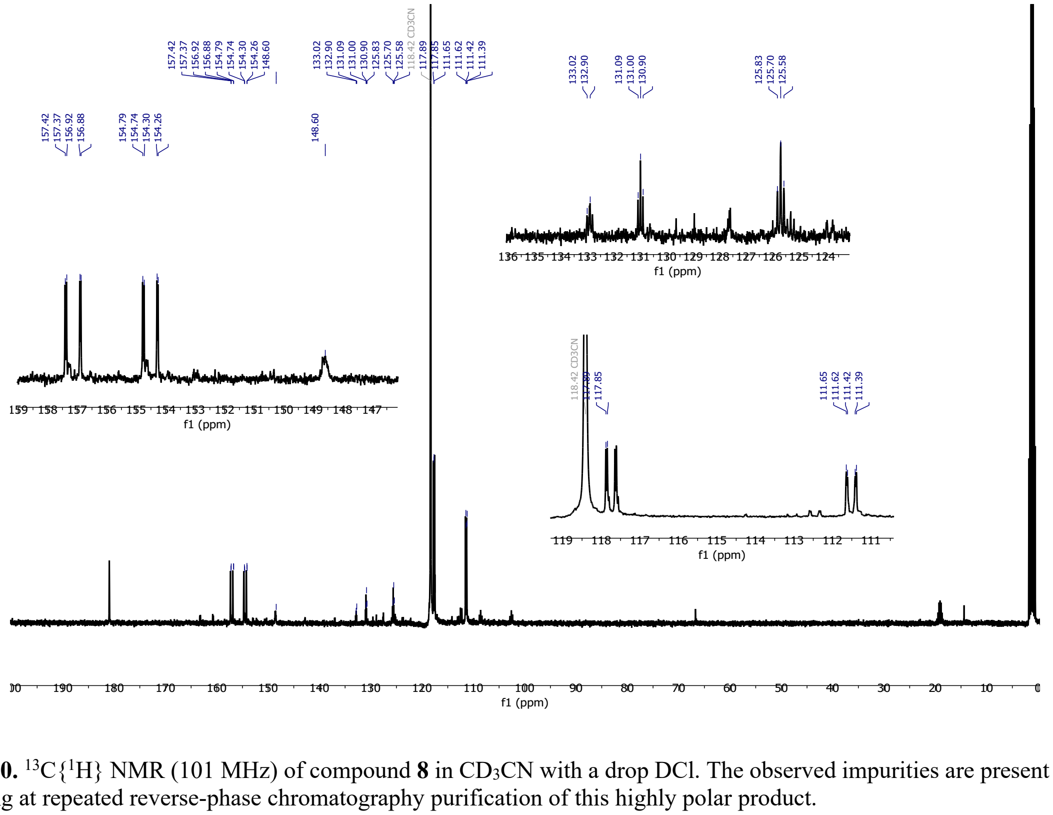

Figure S40. $^{13}C\{^1H\}$ NMR (101 MHz) of compound **8** in CD_3CN with a drop DCl. The observed impurities are present despite of challenging at repeated reverse-phase chromatography purification of this highly polar product.

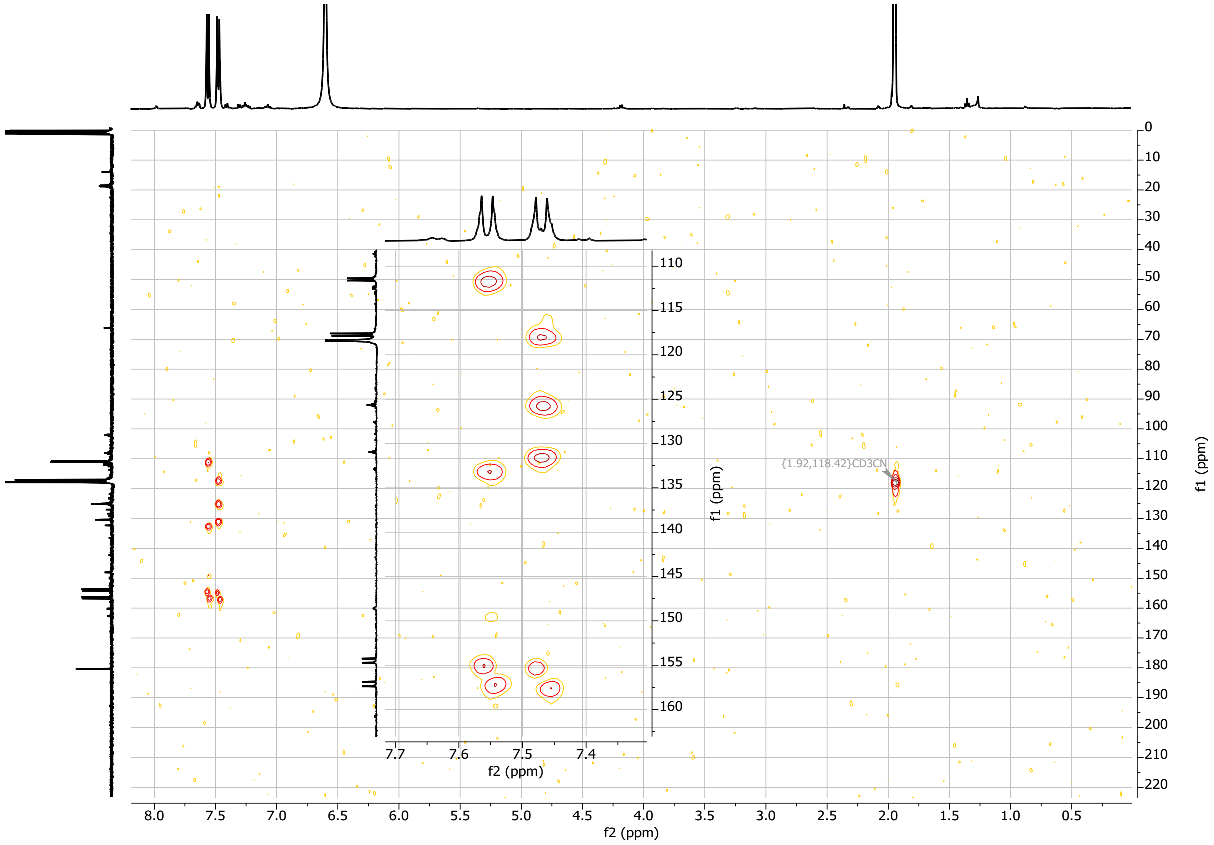

Figure S41. HMBC 2D NMR (400 MHz) of compound **8** in CD_3CN with a drop DCl. The observed impurities are present despite of challenging at repeated reverse-phase chromatography purification of this highly polar product.

(*trans*)-1-(2,6-difluoro-4-((trimethylsilyl)ethynyl)phenyl)-2-(2,4,6-trifluorophenyl)diazene (9)

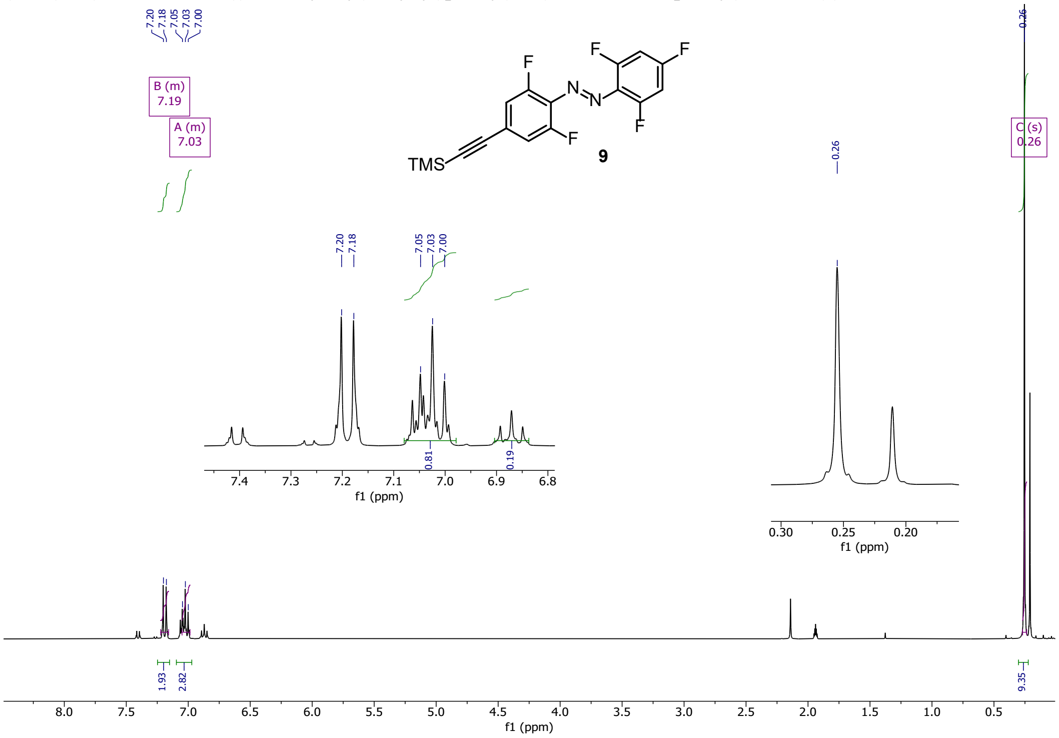

Figure S42. ^{1}H NMR (400 MHz) of compound **9** (81:19=*trans*:*cis*) in $CHCl_3$.

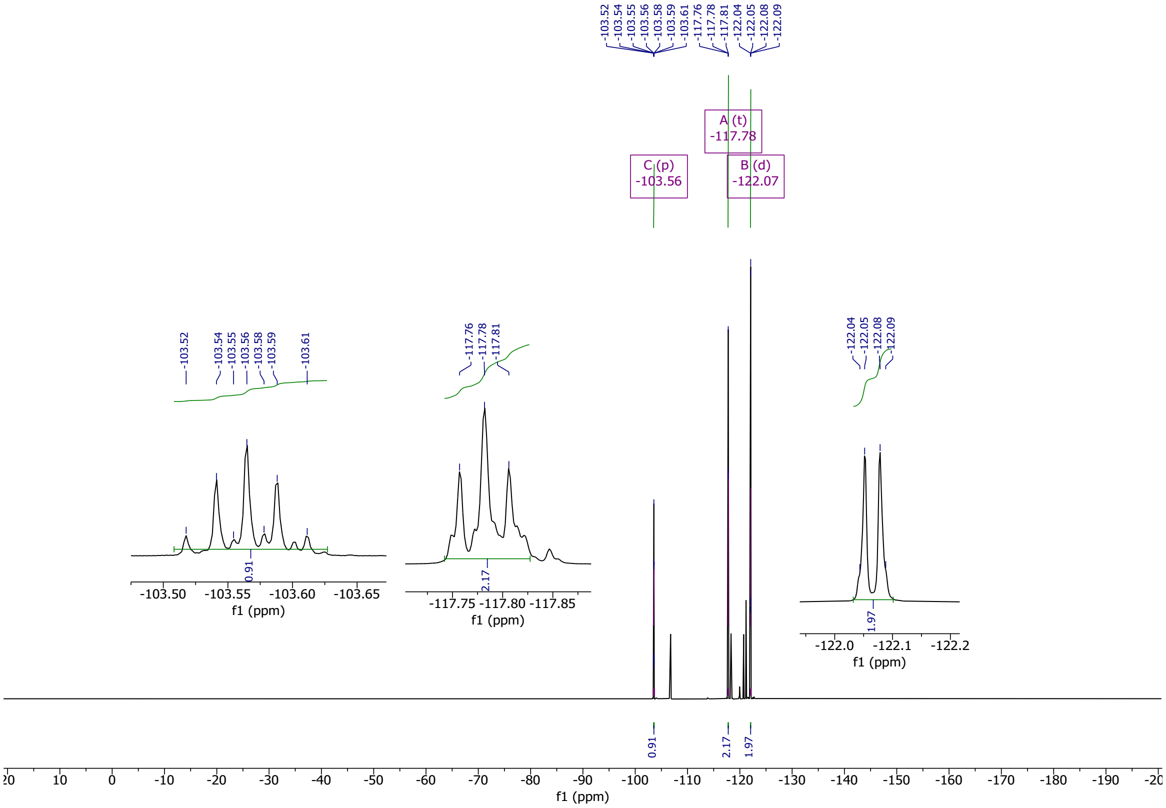

Figure S43. ^{19}F NMR (376 MHz) of compound **9** (81:19=*trans*:*cis*) in $CHCl_3$.

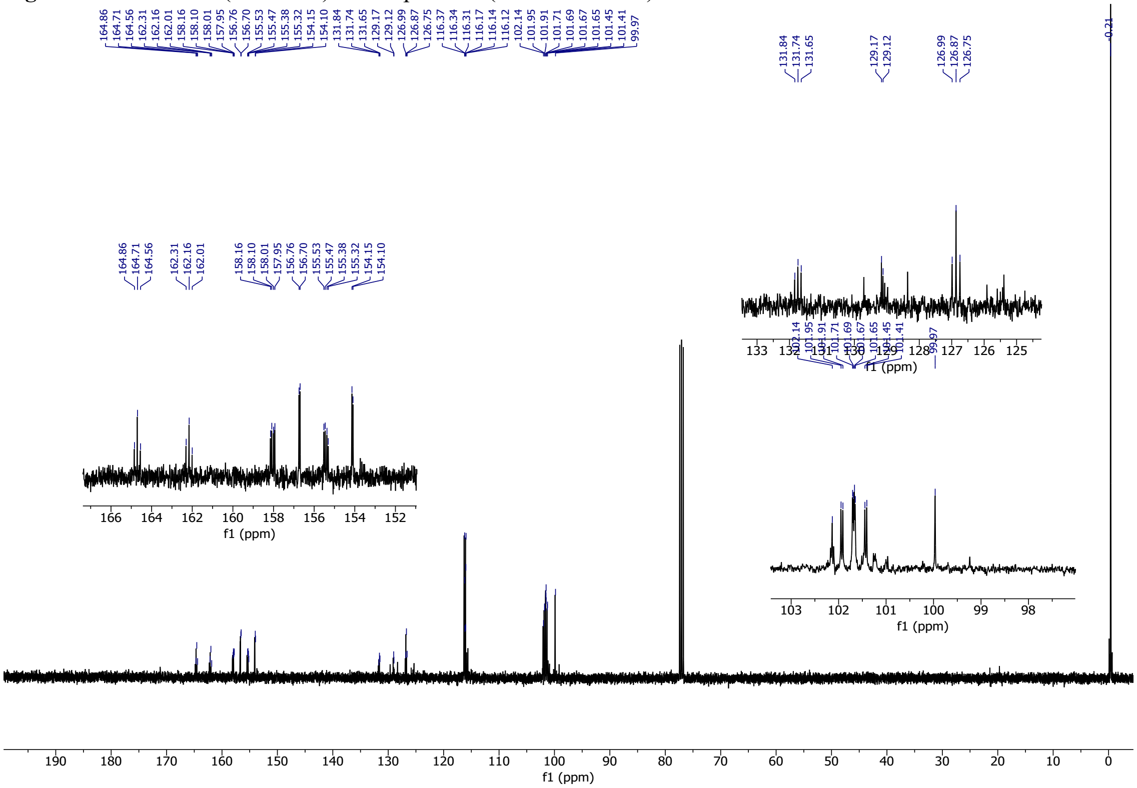

Figure S44. $^{13}C\{^1H\}$ NMR (101 MHz) of compound **9** (81:19=*trans*:*cis*) in $CHCl_3$.

(*trans*)-1-(2,6-difluorophenyl)-2-(4-ethynyl-2,6-difluorophenyl)diazene (5)

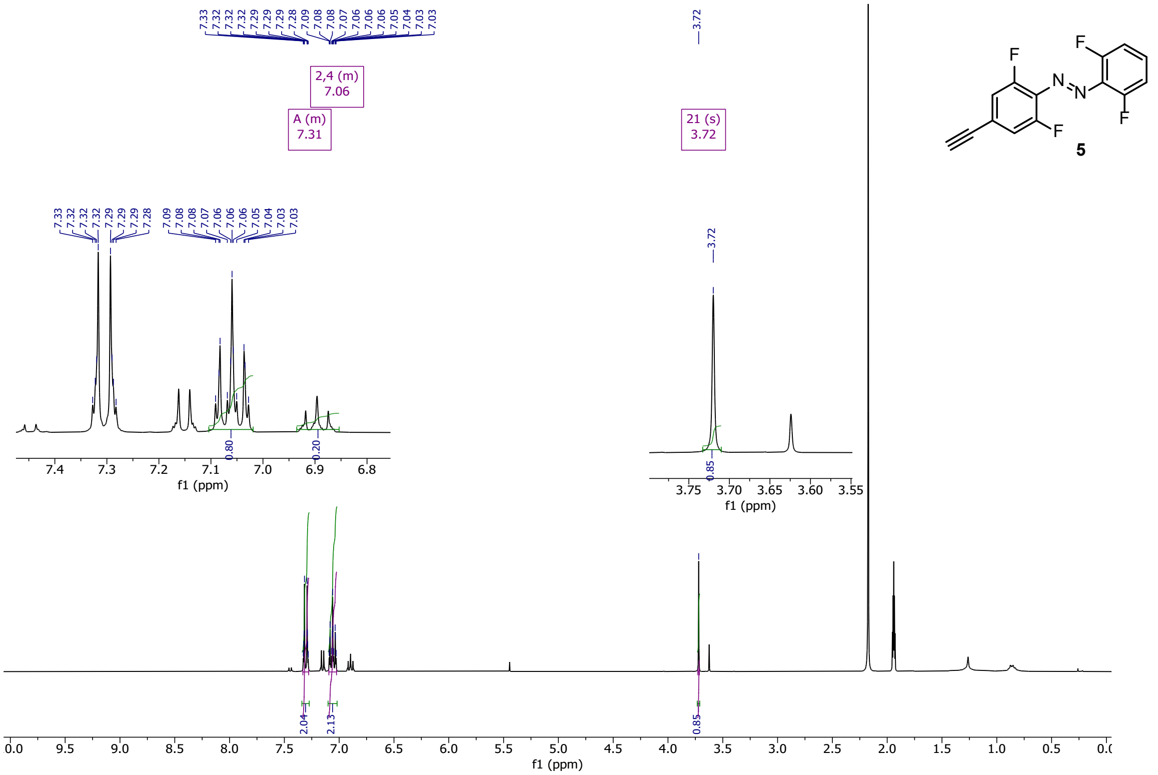

Figure S45. ^1H NMR (400 MHz) of compound **5** (80:20=*trans*:*cis*) in CD_3CN.

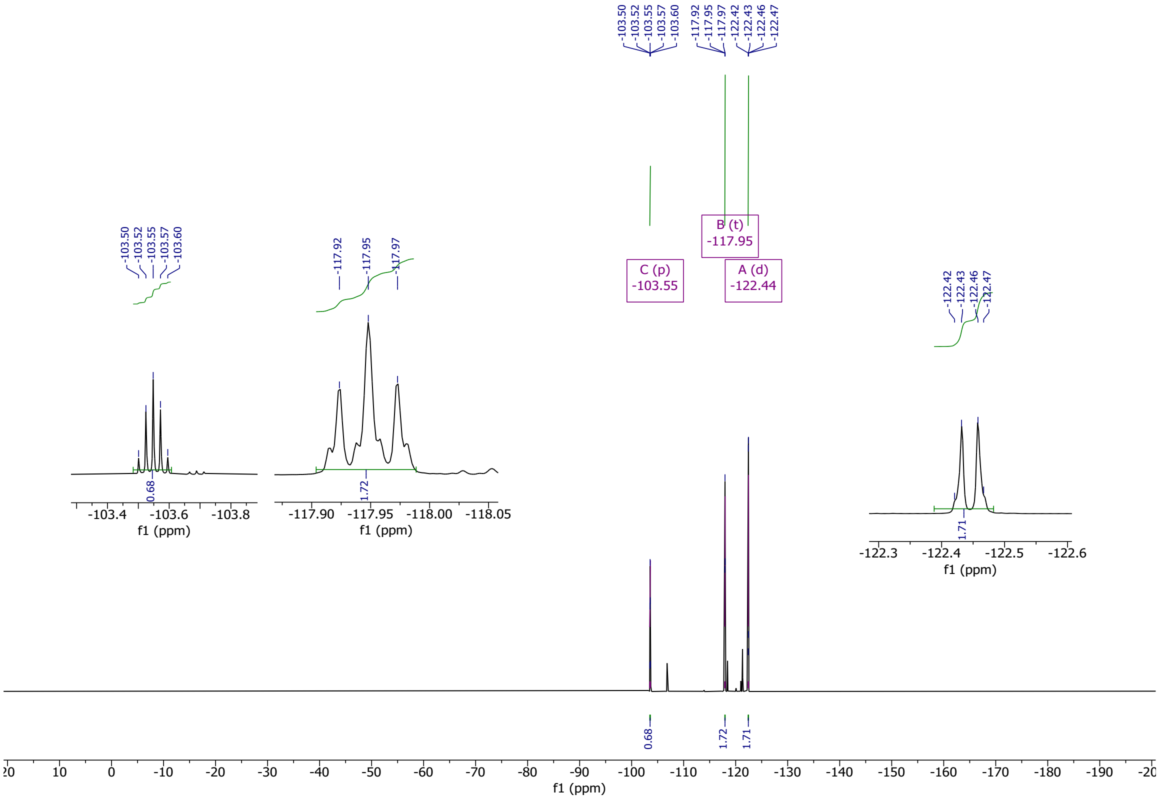

Figure S46. ^{19}F NMR (376 MHz) of compound **5** (80:20=*trans*:*cis*) in CD_3CN.

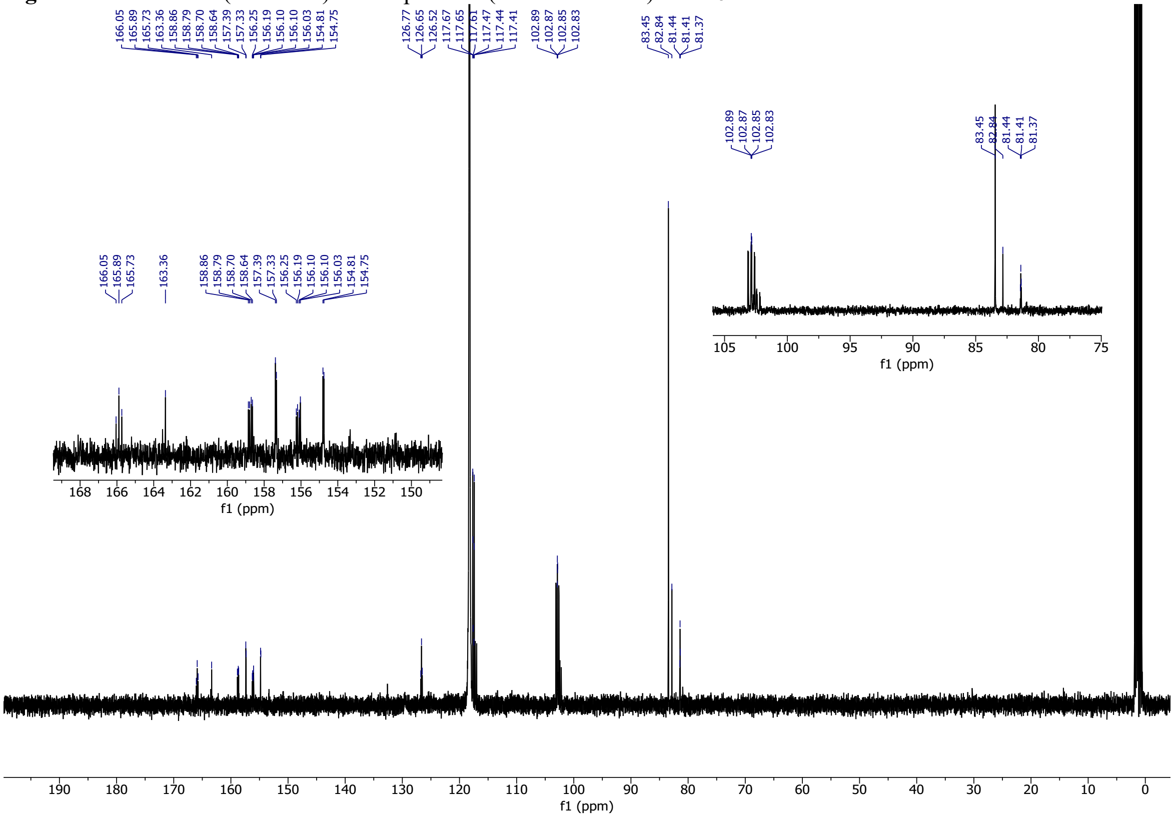

Figure S47. ^{13}C{^{1}H} NMR (101 MHz) of compound **5** (80:20=*trans*:*cis*) in CD_3CN.

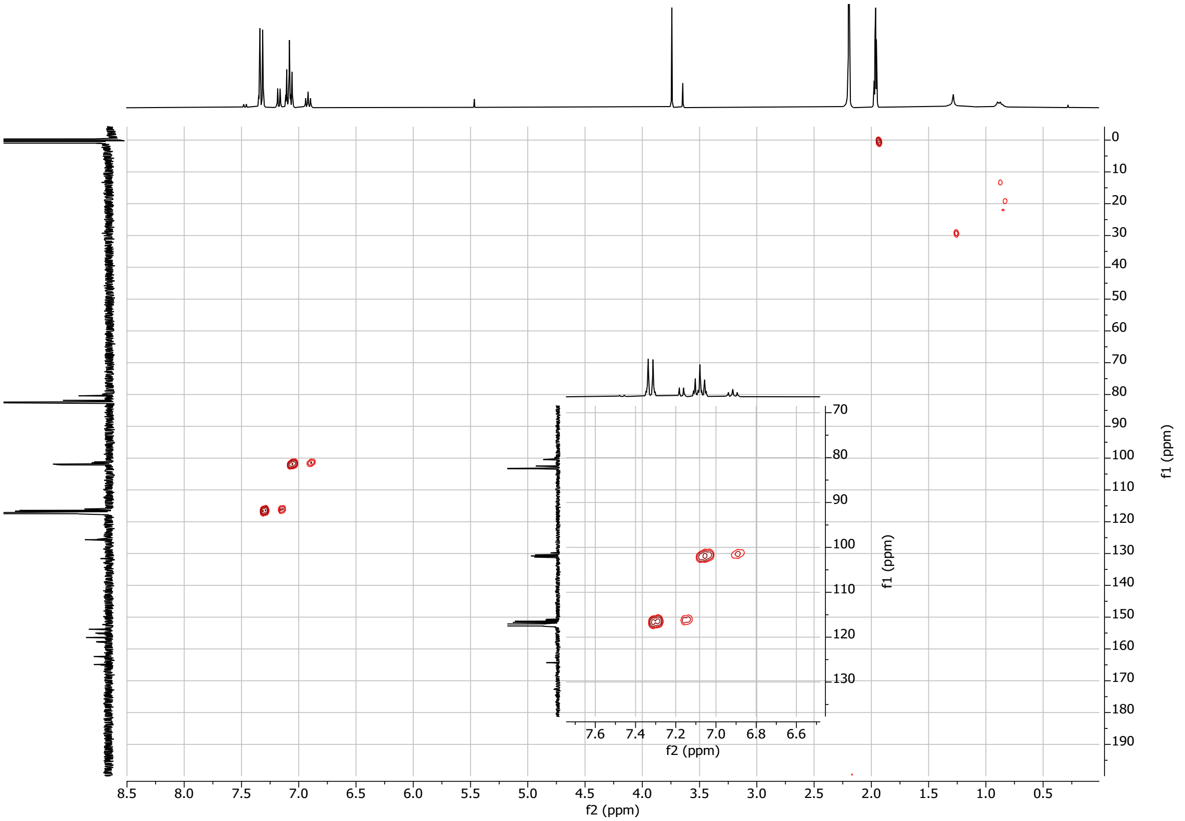

Figure S48. HSQC 2D NMR (400 MHz) of compound **5** (80:20=*trans*:*cis*) in CD_3CN.

***(trans)*-*N*-(3,5-difluoro-4-((2,4,6-trifluorophenyl)diazenyl)phenyl)acetamide (11)**

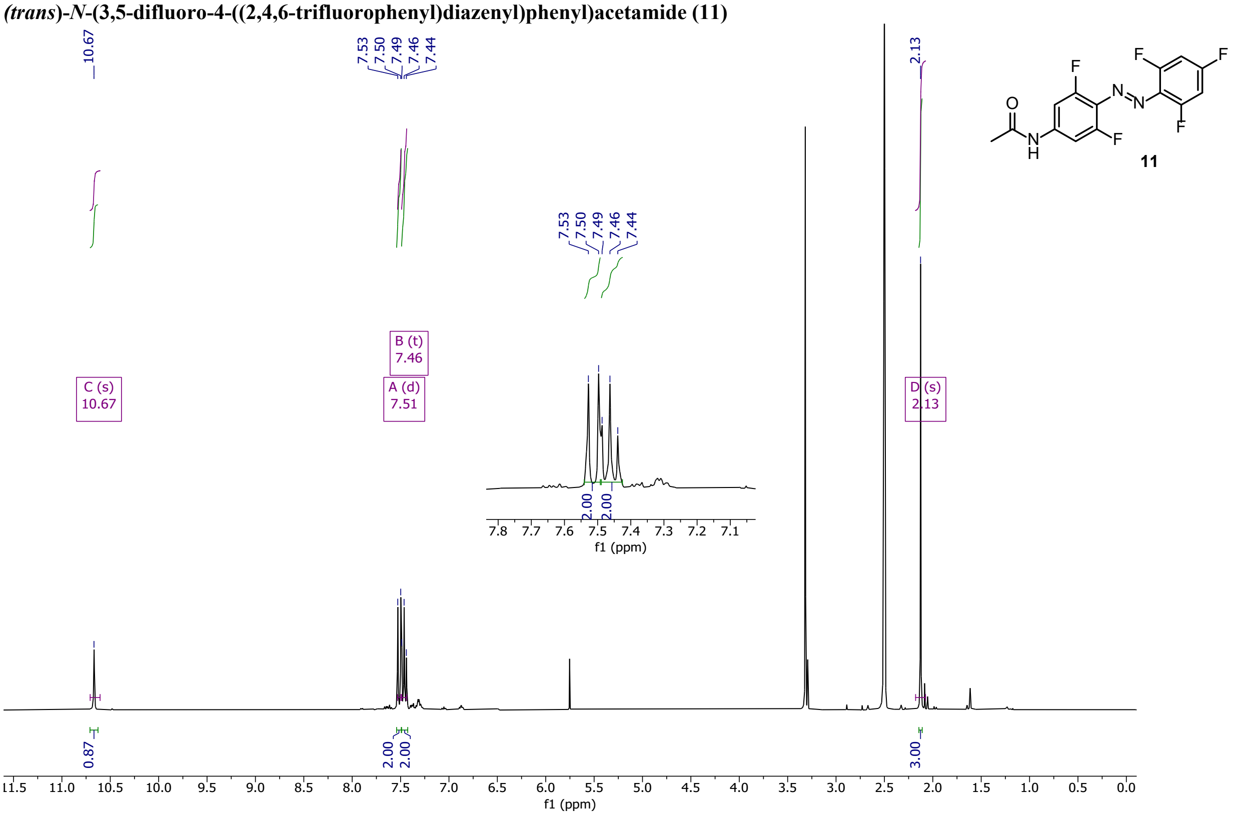

Figure S49. ^{1}H NMR (400 MHz) of compound **11** in DMSO-d_6.

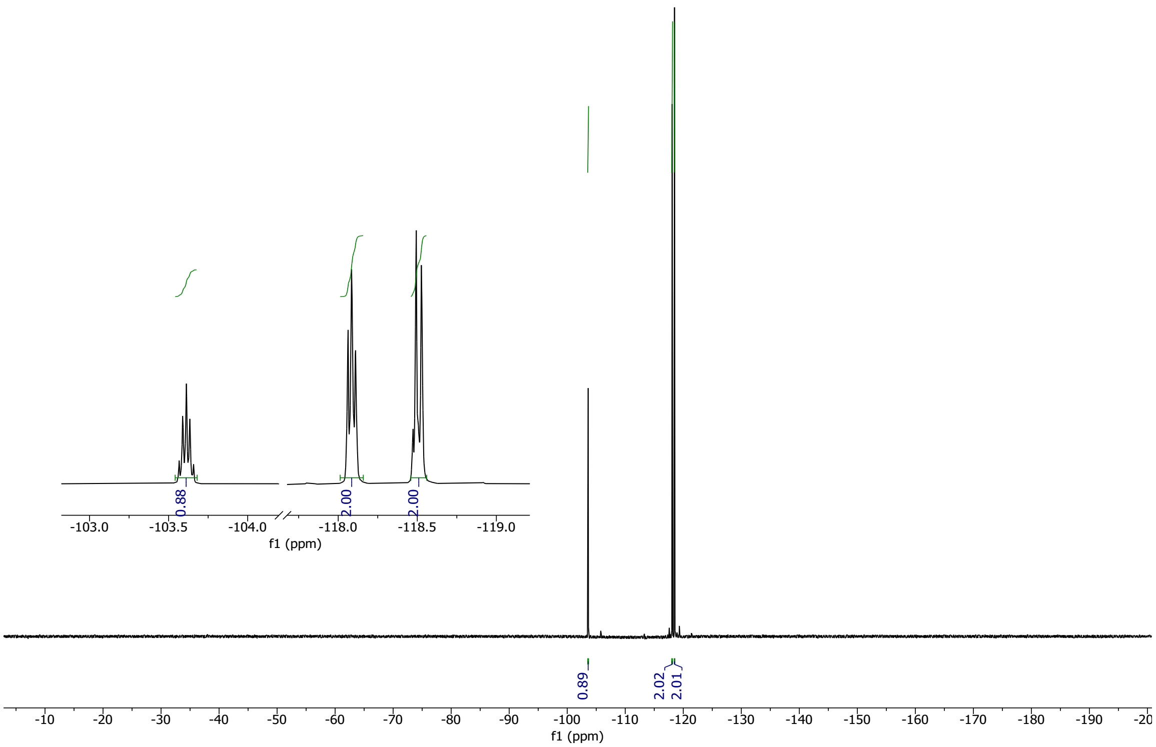

Figure S50. ^{19}F NMR (376 MHz) of compound **11** in DMSO-d_6.

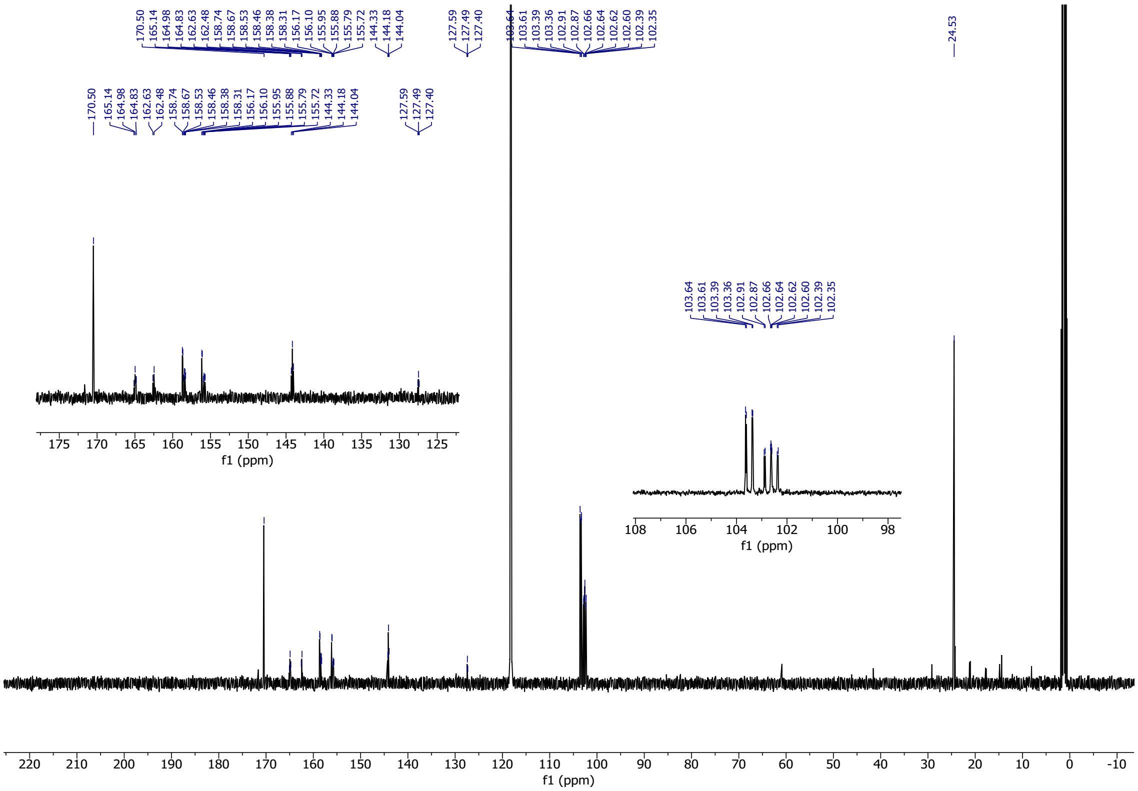

Figure S51. $^{13}C\{^{1}H\}$ NMR (101 MHz) of compound **11** in CD_3CN.

(*trans*)-1-(3,5-difluoro-4-((2,4,6-trifluorophenyl)diazenyl)phenyl)-1H-pyrrole-2,5-dione (12)

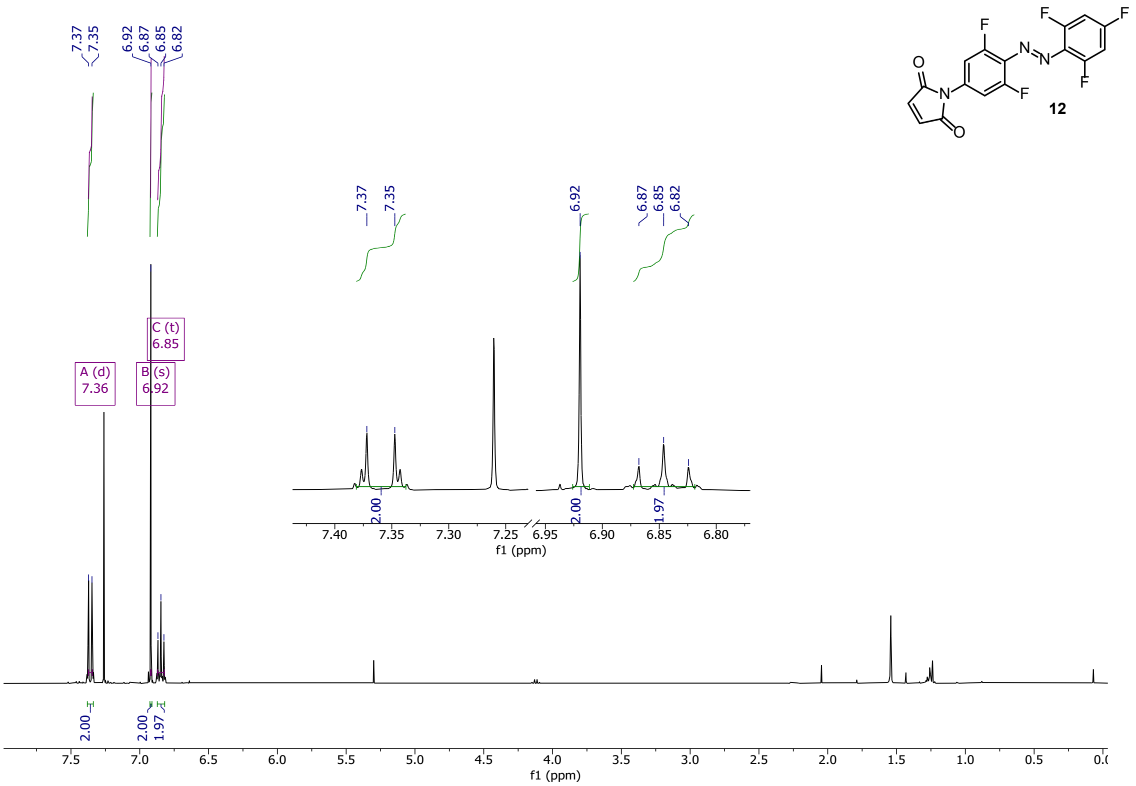

Figure S52. ^{1}H NMR (400 MHz) of compound **12** in $CDCl_3$.

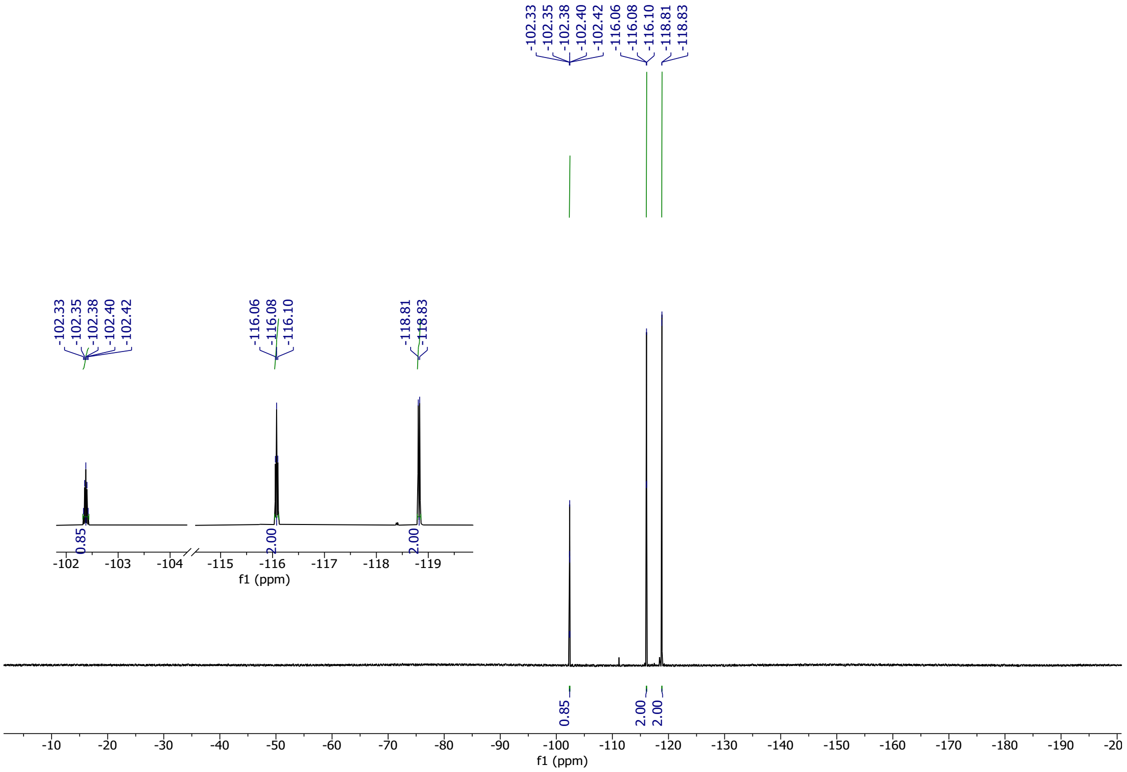

Figure S53. ^{19}F NMR (376 MHz) of compound **12** in $CDCl_3$.

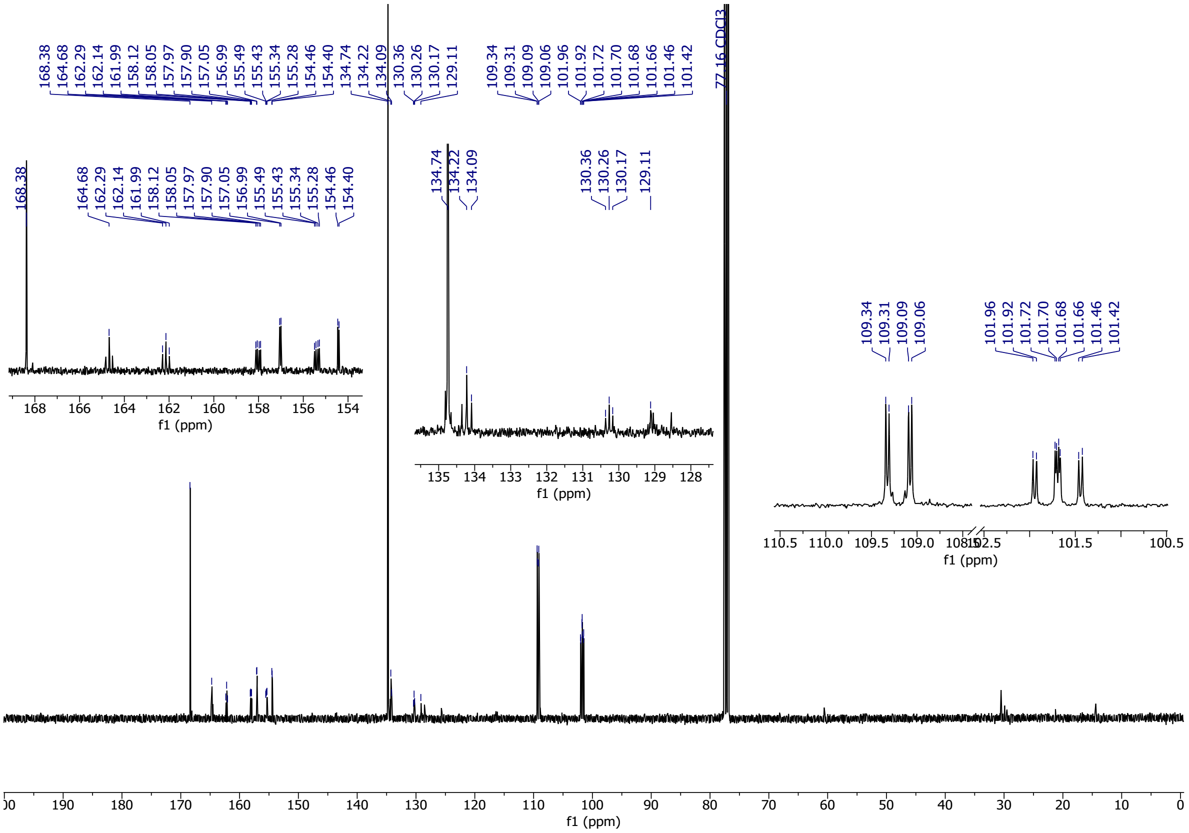

Figure S54. $^{13}C\{^{1}H\}$ NMR (101 MHz) of compound **12** in $CDCl_3$.

trans-4-((4-acetamido-2,6-difluorophenyl)diazenyl)-3,5-difluorobenzenesulfonate (13)

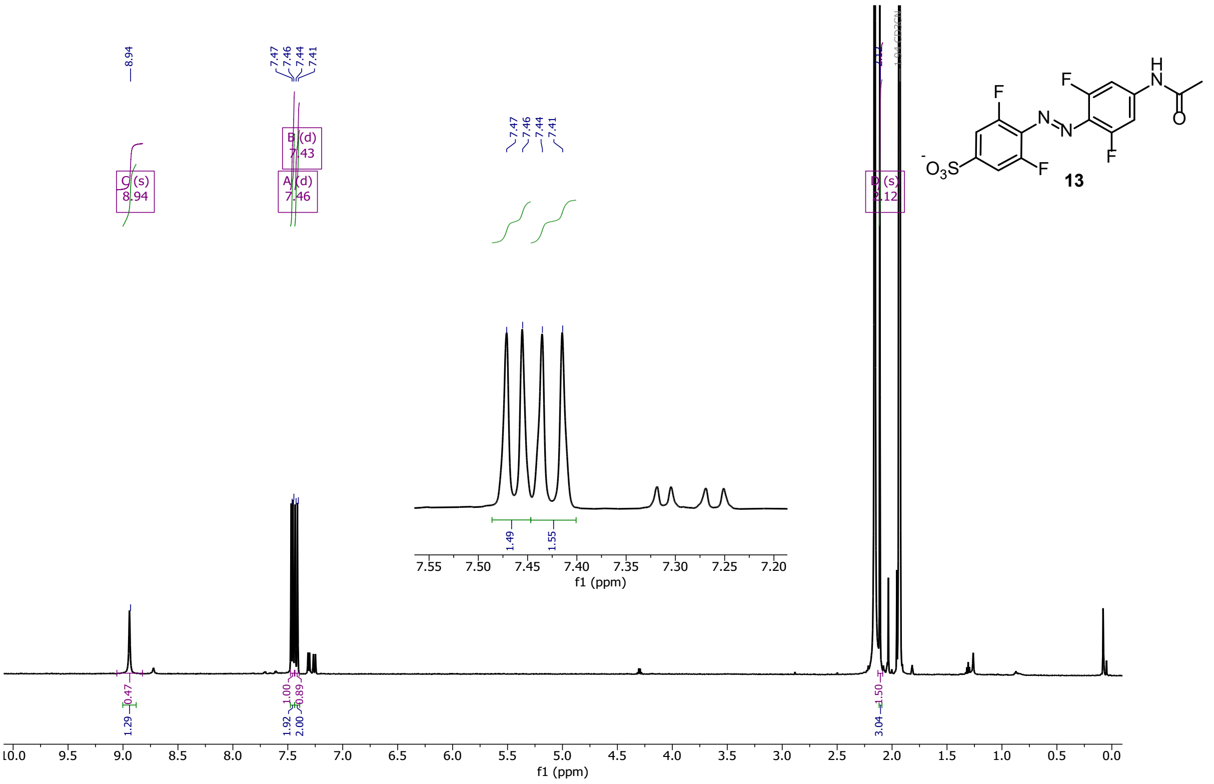

Figure S55. ^{1}H NMR (400 MHz) of compound **13** in CD_3CN with a drop D_2O.

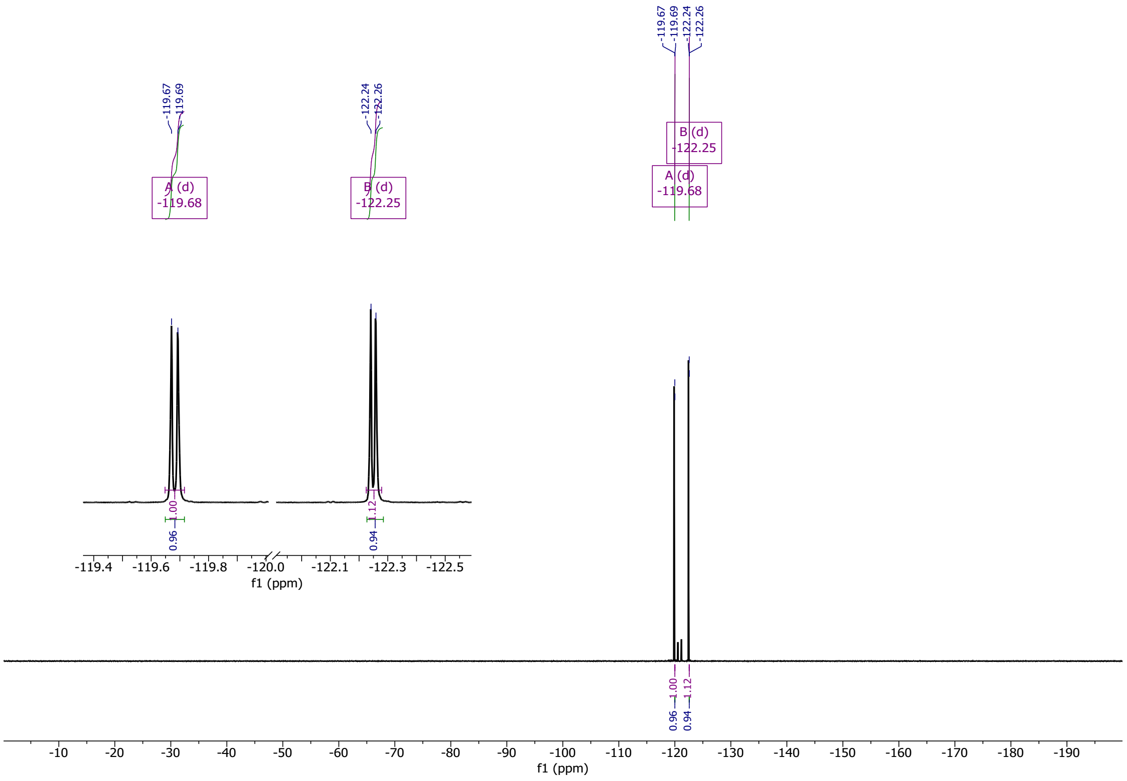

Figure 56. ^{19}F NMR (376 MHz) of compound **13** in CD_3CN with a drop D_2O.

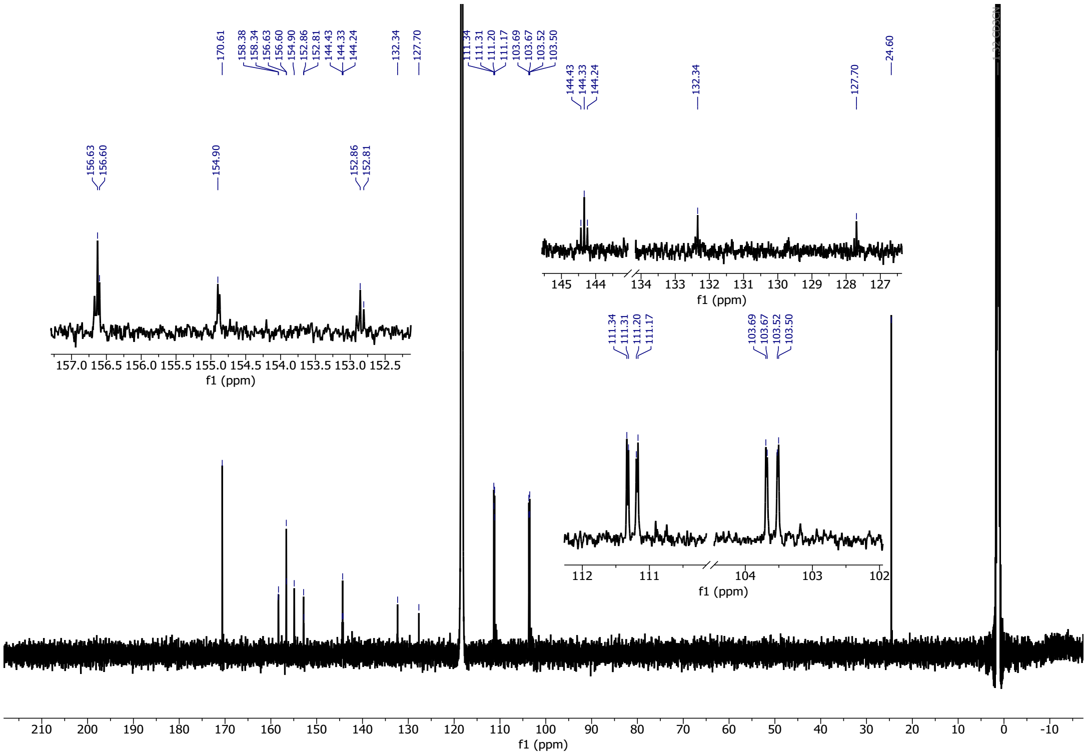

Figure S57 . $^{13}C\{^1H\}$ NMR (101 MHz) of compound **13** in CD_3CN with a drop D_2O.

(2*R*,3*R*,4*S*,5*R*,6*R*)-2-(acetoxymethyl)-6-(((2*R*,3*R*,4*S*,5*R*,6*R*)-3,4,5-triacetoxy-6-((4-(4-((*trans*)-(2,6-difluorophenyl)diazenyl)-3,5-difluorophenyl)-1*H*-1,2,3-triazol-1-yl)methyl)tetrahydro-2*H*-pyran-2-yl)oxy)tetrahydro-2*H*-pyran-3,4,5-triyl triacetate (16)

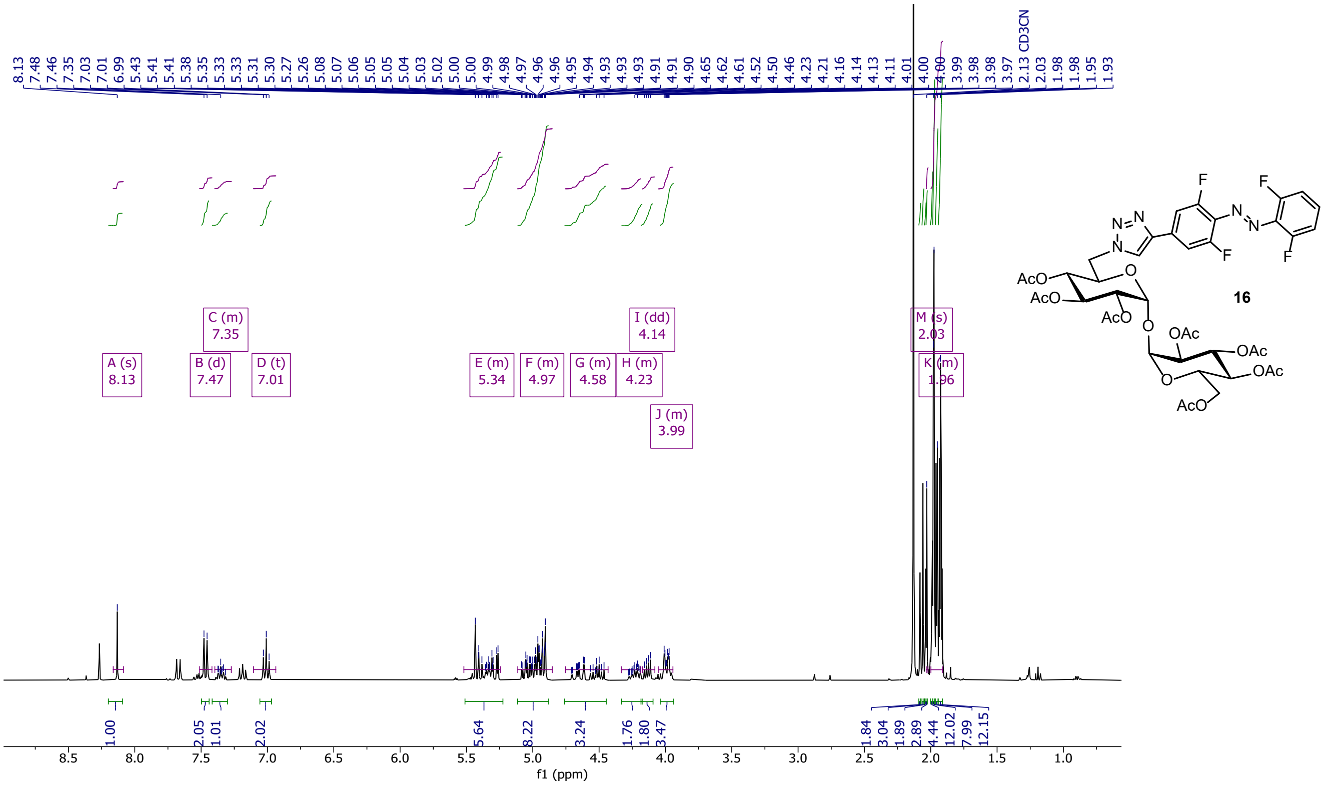

Figure S58. ^1H NMR (400 MHz) of non-irradiated (64% *cis*) **16** in CD_3CN.

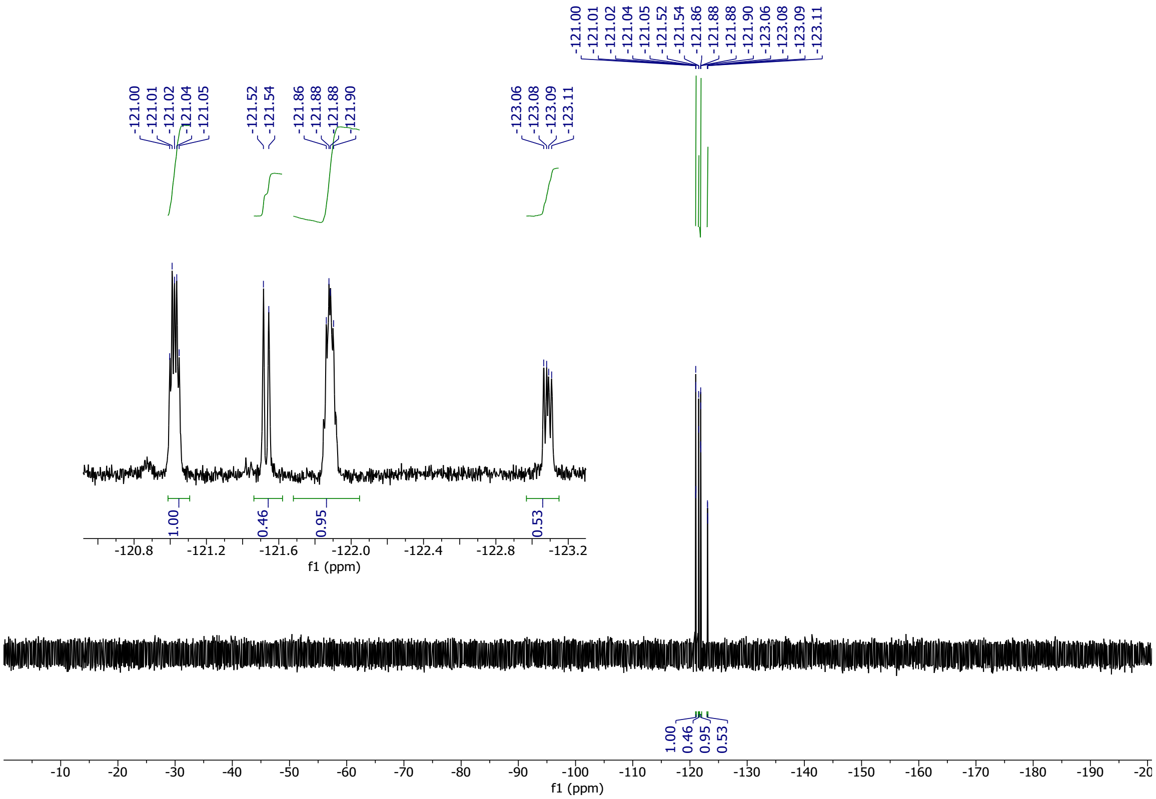

Figure S59. ^{19}F NMR (376 MHz) of non-irradiated (64% cis) **16** in CD_3CN.

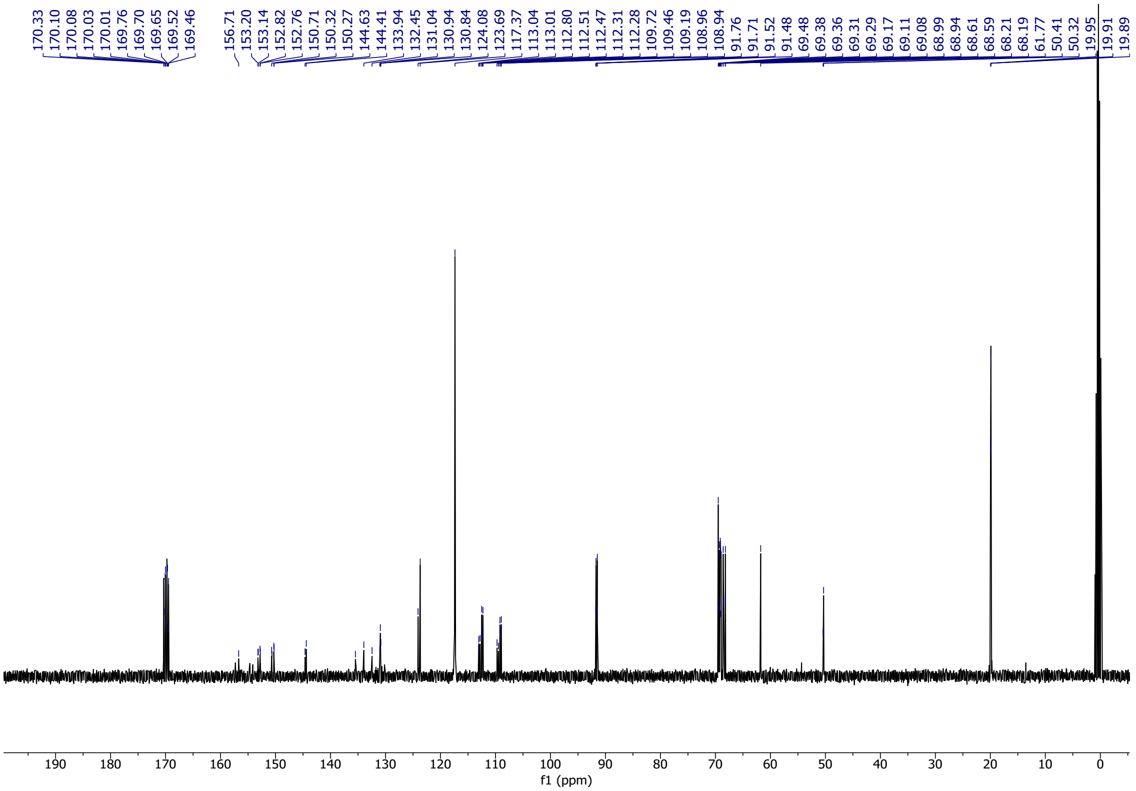

Figure S60. $^{13}C\{^1H\}$ NMR (101 MHz) of non-irradiated (64% *cis*) **16** in CD_3CN.

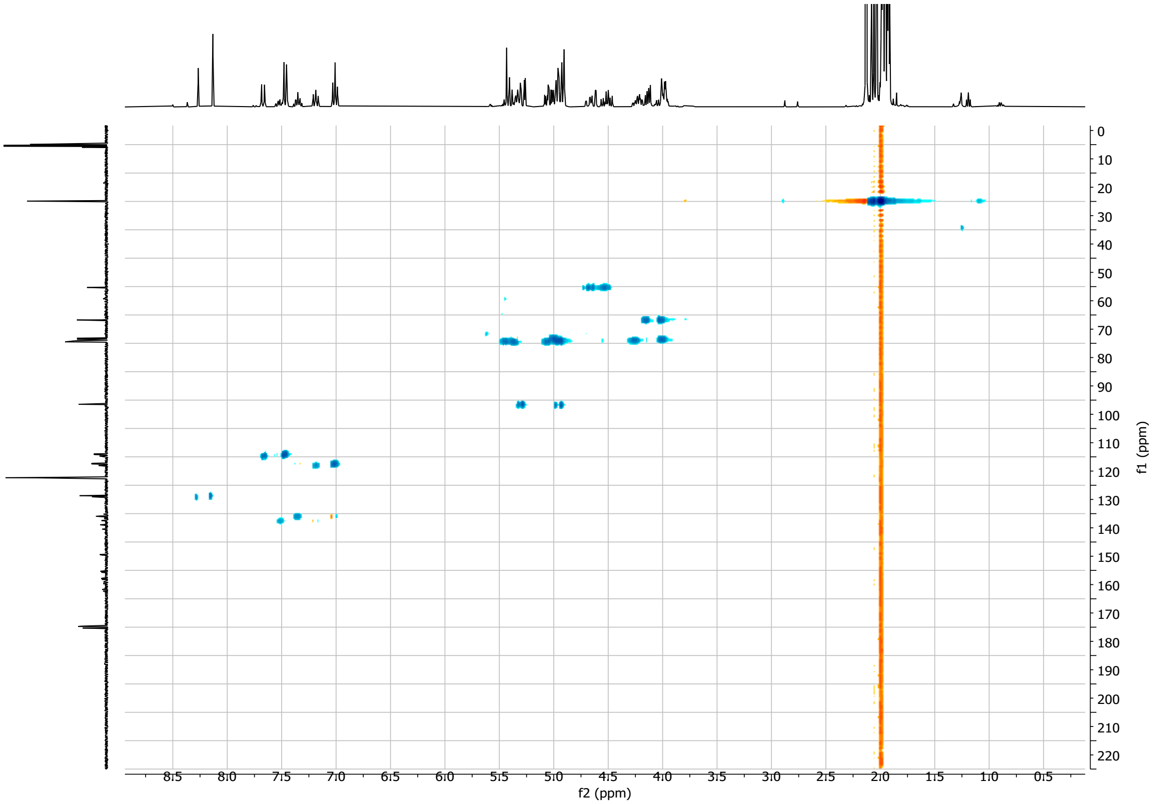

Figure S61. HSQC 2D NMR (400 MHz) of non-irradiated (64% *cis*) **16** in CD_3CN.

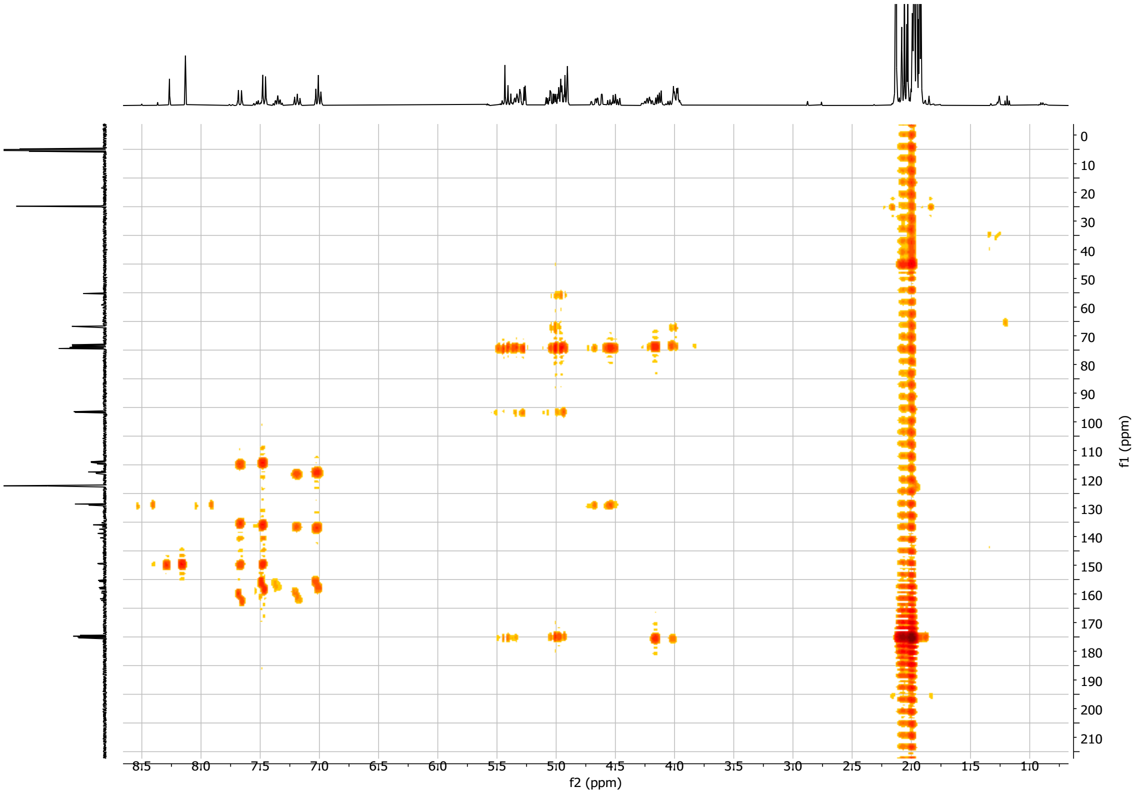

Figure S62. HMBC 2D NMR (400 MHz) of non-irradiated (64% *cis*) **16** in CD_3CN.

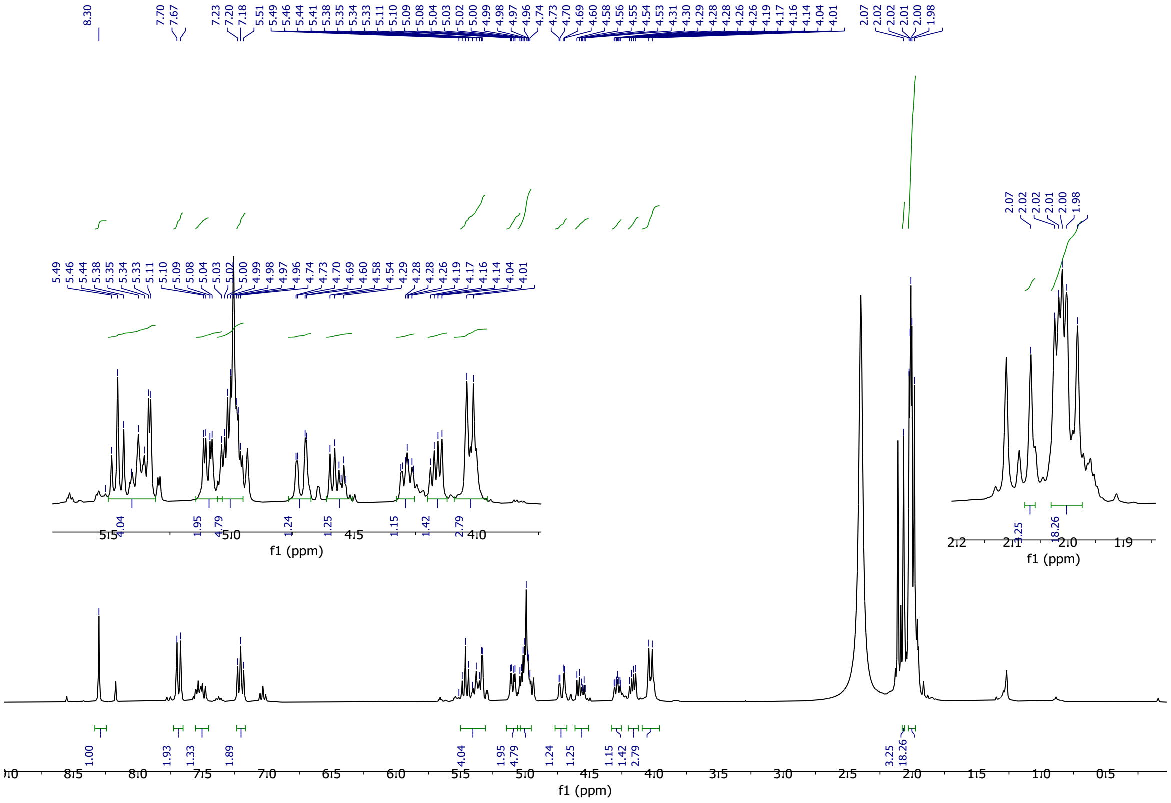

Figure S63. ^{1}H NMR (400 MHz) of **16** at PSS_{430nm} (80% *trans*) in CD_3CN.

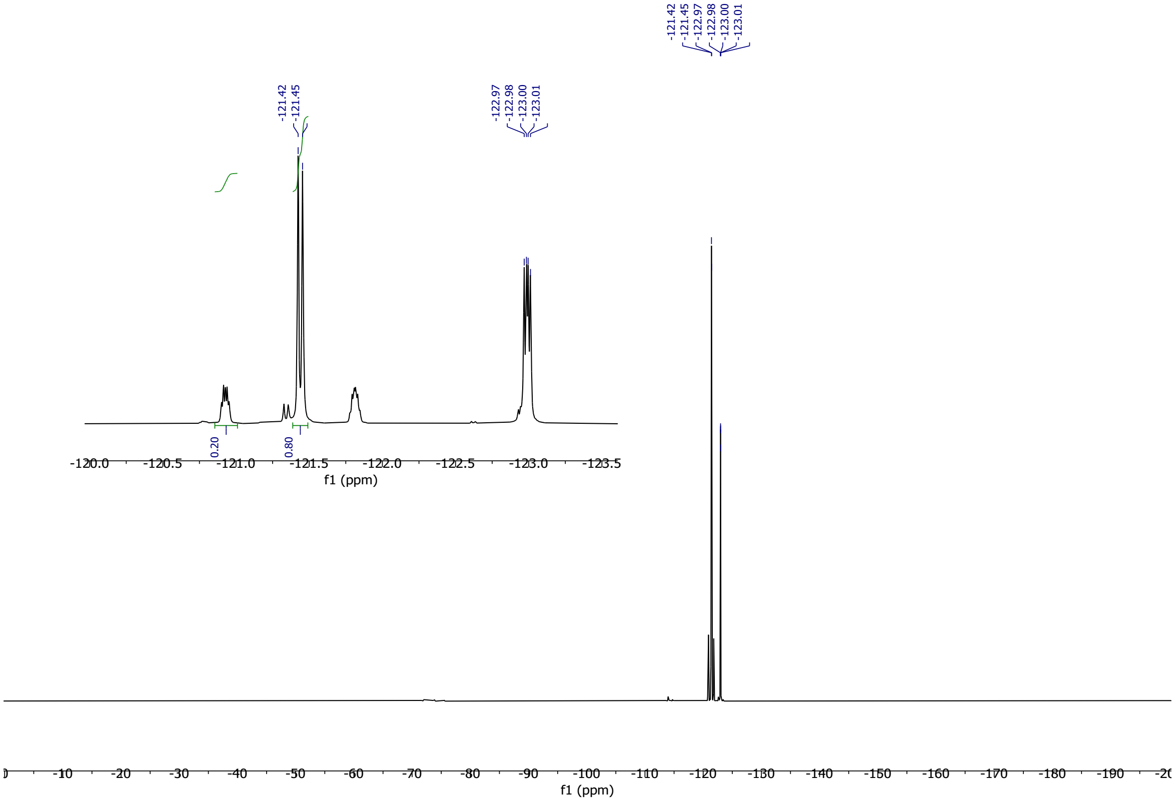

Figure S64. ^{19}F NMR (376 MHz) of **16** at PSS_{430nm} (80% *trans*) in CD_3CN.

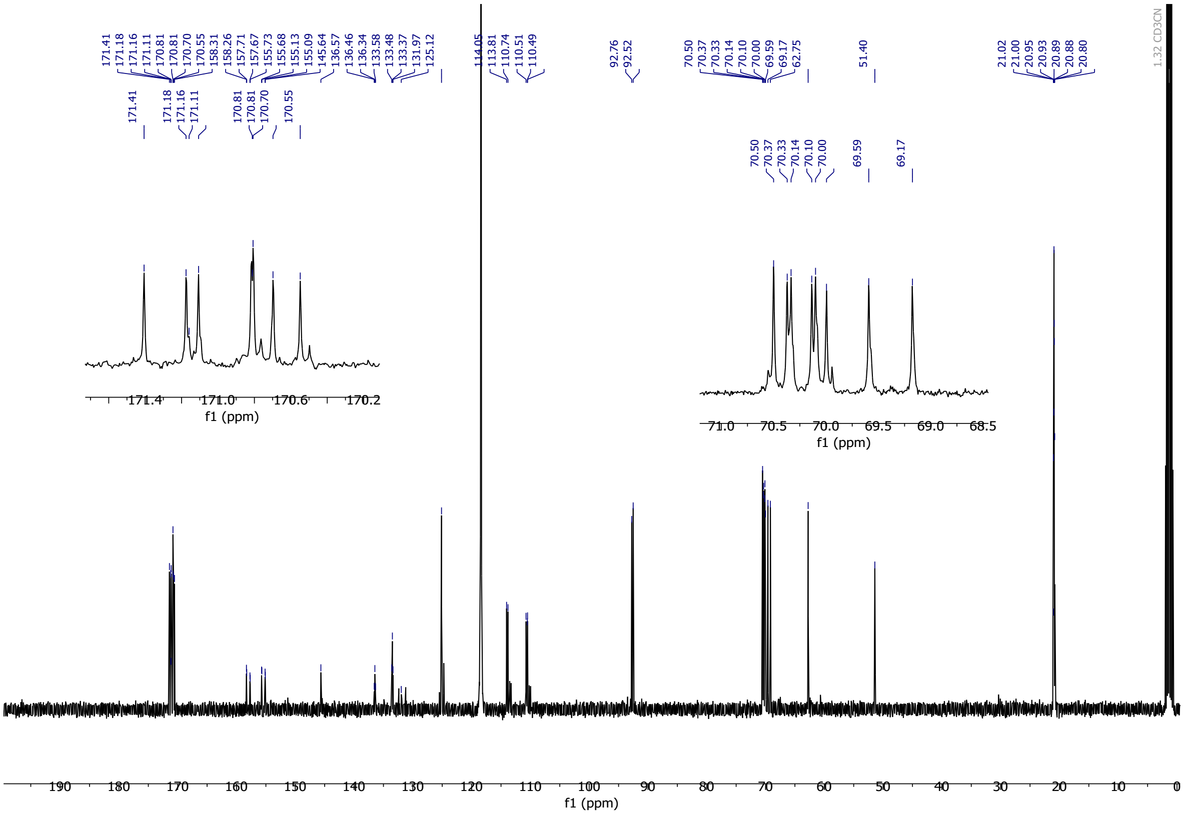

Figure S65. $^{13}C\{^{1}H\}$ NMR (101 MHz) of **16** at PSS_{430nm} (80% *trans*) in CD_3CN.

(2*R*,3*S*,4*S*,5*R*,6*R*)-2-((4-(4-((*trans*)-(2,6-difluorophenyl)diazenyl)-3,5-difluorophenyl)-1*H*-1,2,3-triazol-1-yl)methyl)-6-(((2*R*,3*R*,4*S*,5*S*,6*R*)-3,4,5-trihydroxy-6-(hydroxymethyl)tetrahydro-2*H*-pyran-2-yl)oxy)tetrahydro-2*H*-pyran-3,4,5-triol (17)

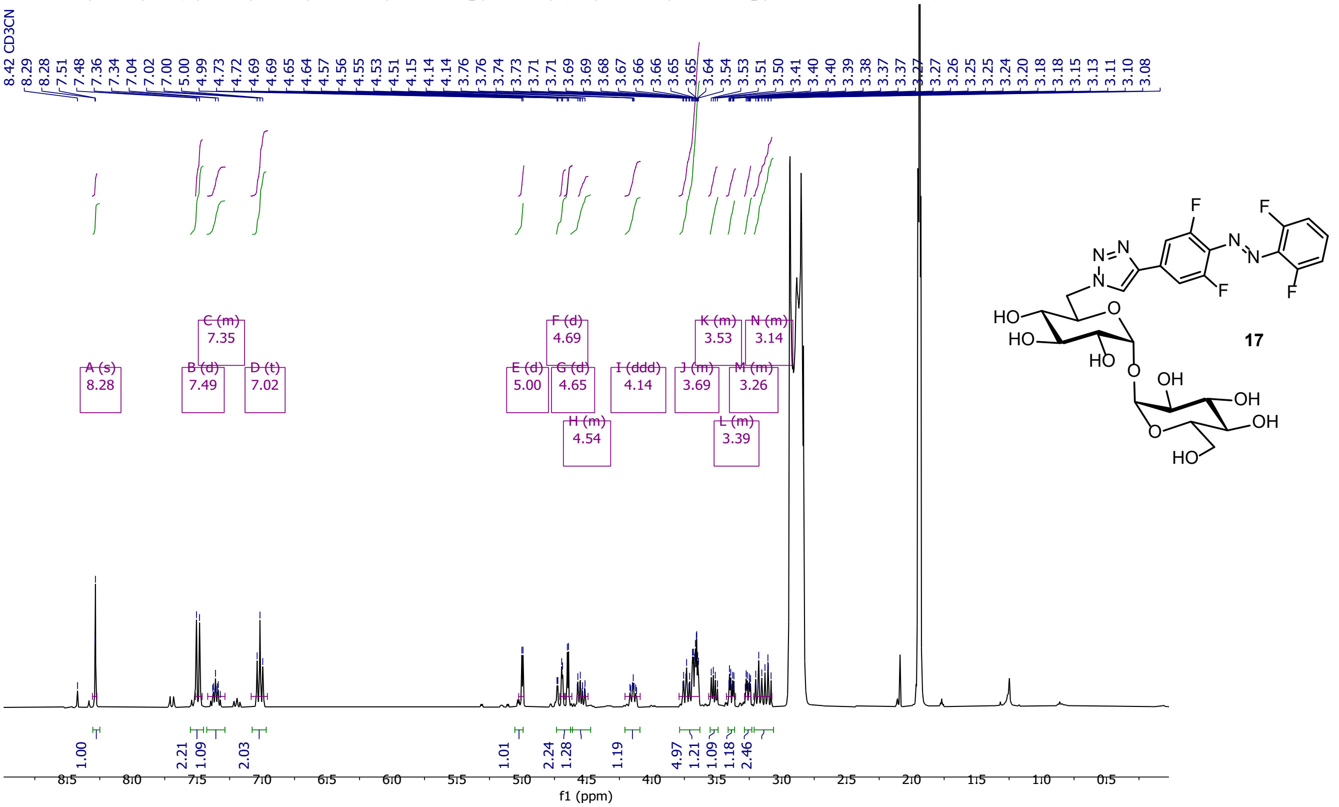

Figure S67. ^{1}H NMR (400 MHz) of non-irradiated **17** (86% *cis*) in CD_3CN with drop of D_2O.

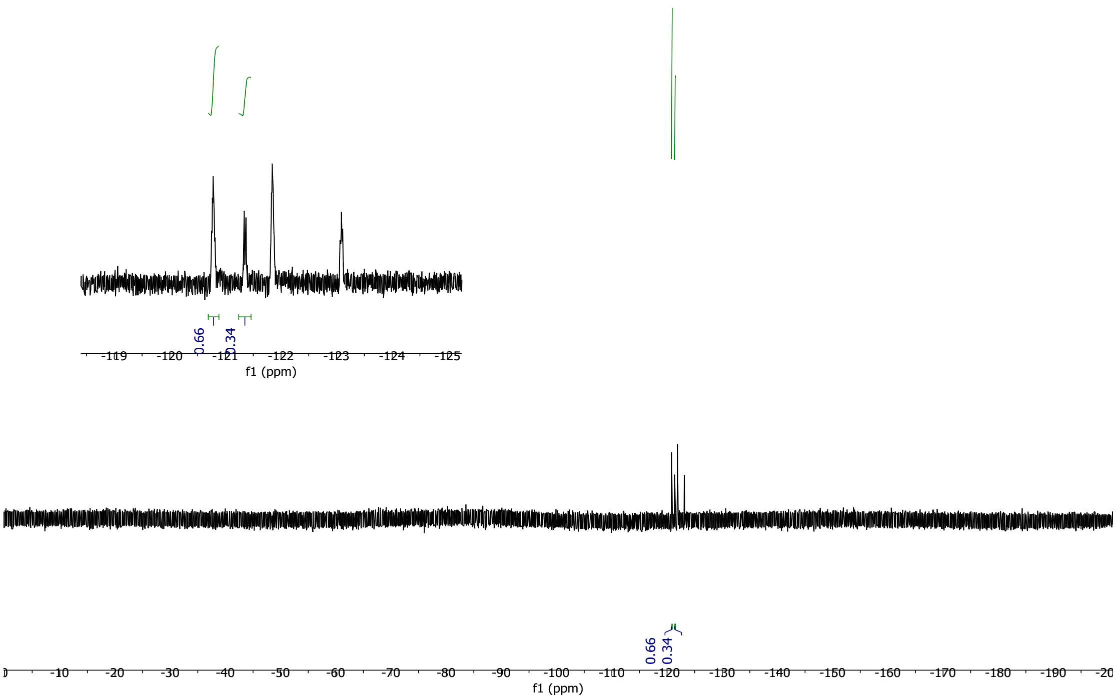

Figure S68. ^{19}F NMR (376 MHz) of non-irradiated **17** (86% *cis*) in CD_3CN with a drop of D_2O.

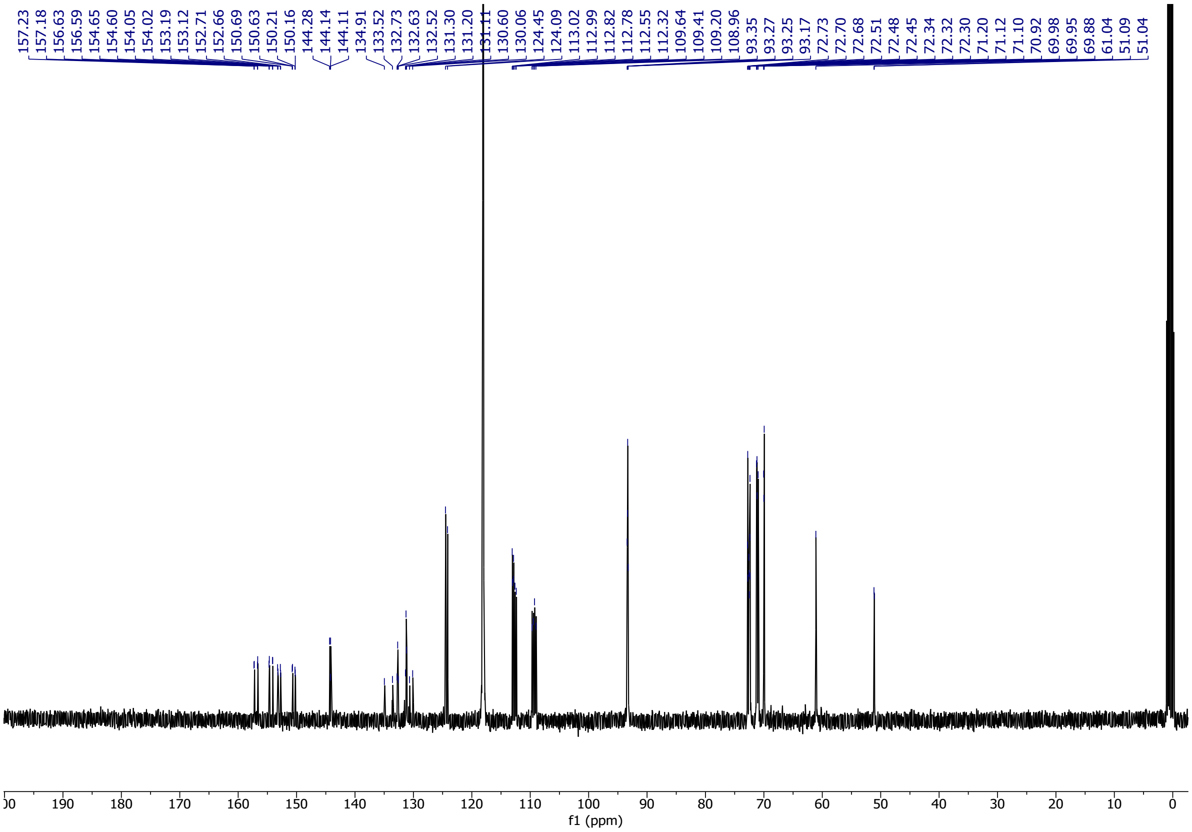

Figure S69. $^{13}C\{^1H\}$ NMR (101 MHz) of non-irradiated **16** (86% *cis*) in CD_3CN with a drop of D_2O.

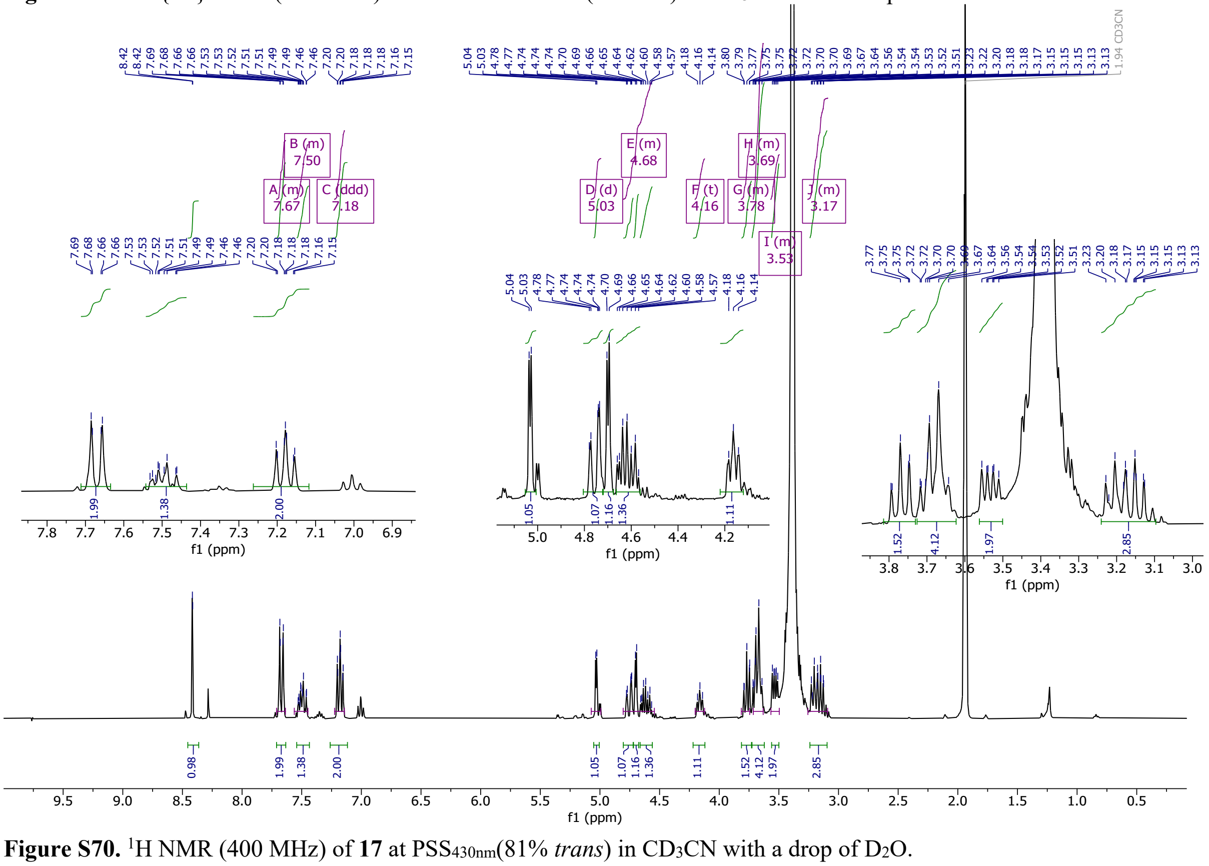

Figure S70. ^1H NMR (400 MHz) of **17** at PSS_{430nm}(81% *trans*) in CD_3CN with a drop of D_2O.

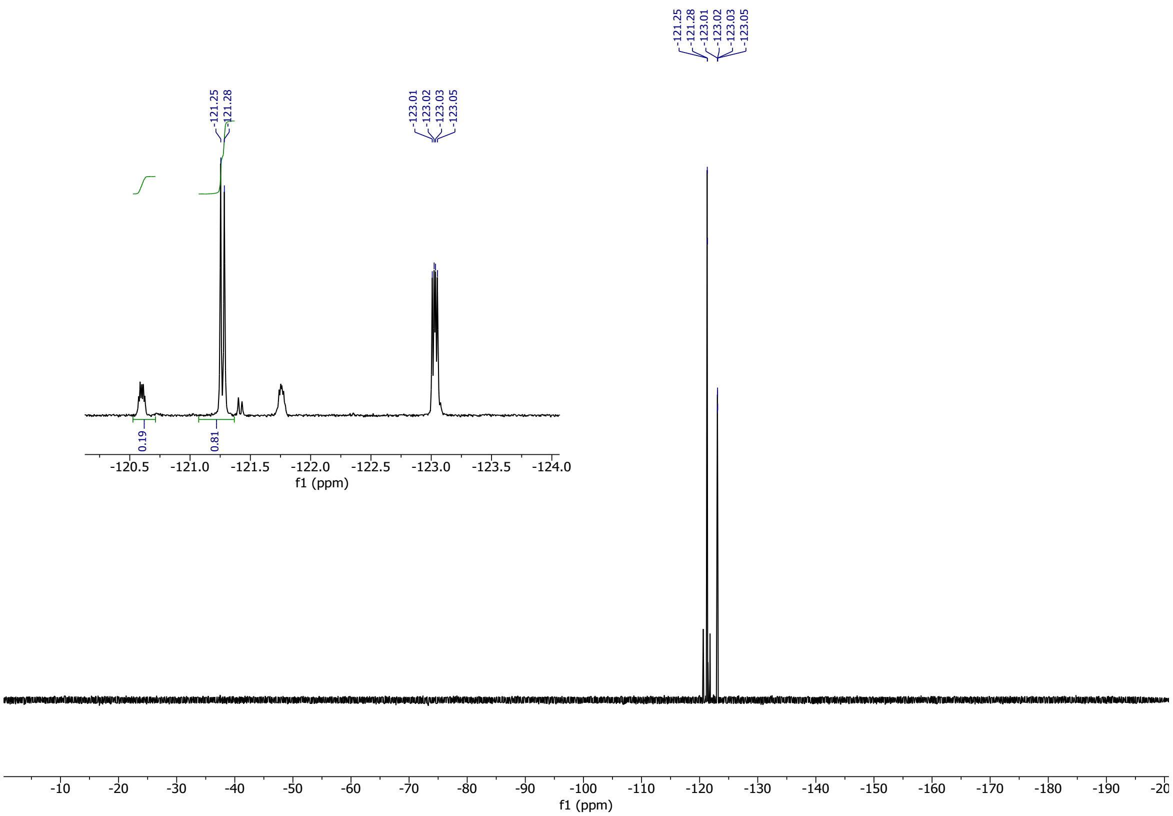

Figure S71. ^{19}F NMR (376 MHz) of **17** at PSS$_{430nm}$(81% *trans*) in CD_3CN with a drop of D_2O.

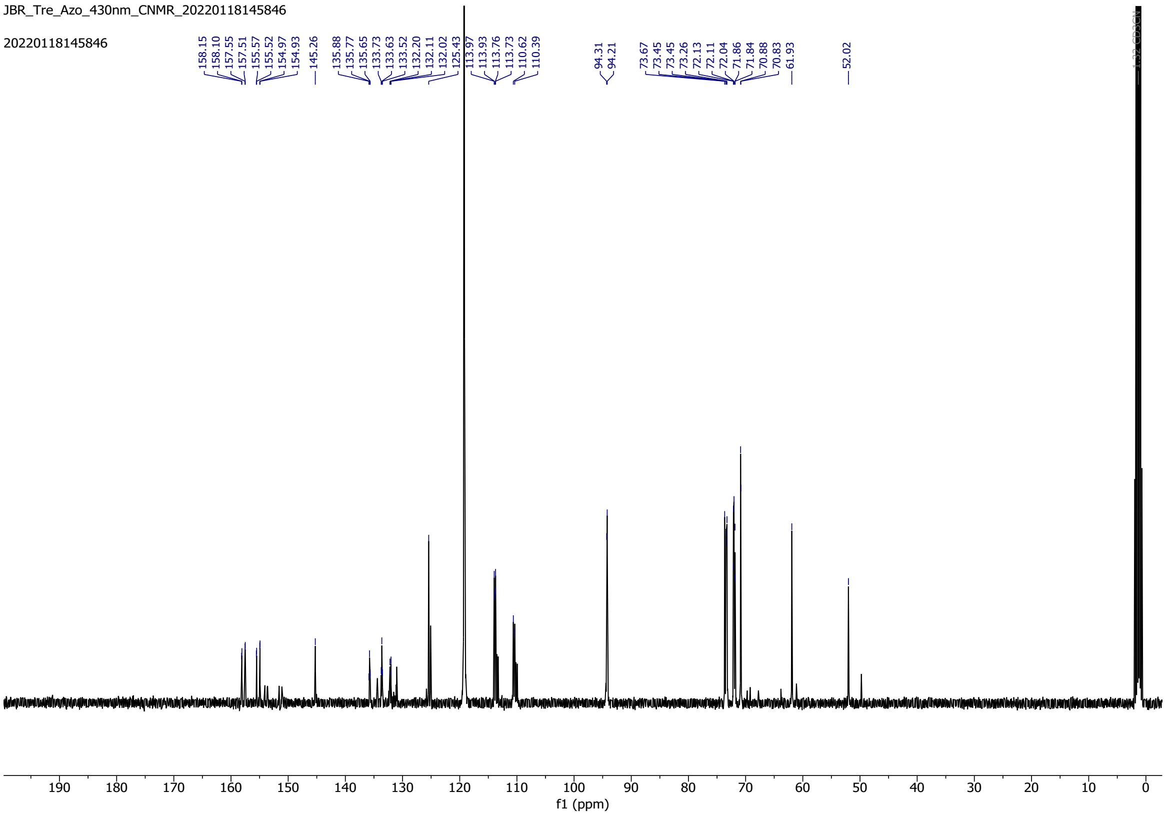

Figure S72. ^{13}C{^{1}H} NMR (101 MHz) of **17** at PSS$_{430nm}$(81% *trans*) in CD_3CN with a drop of D_2O.

4. FT-IR spectra

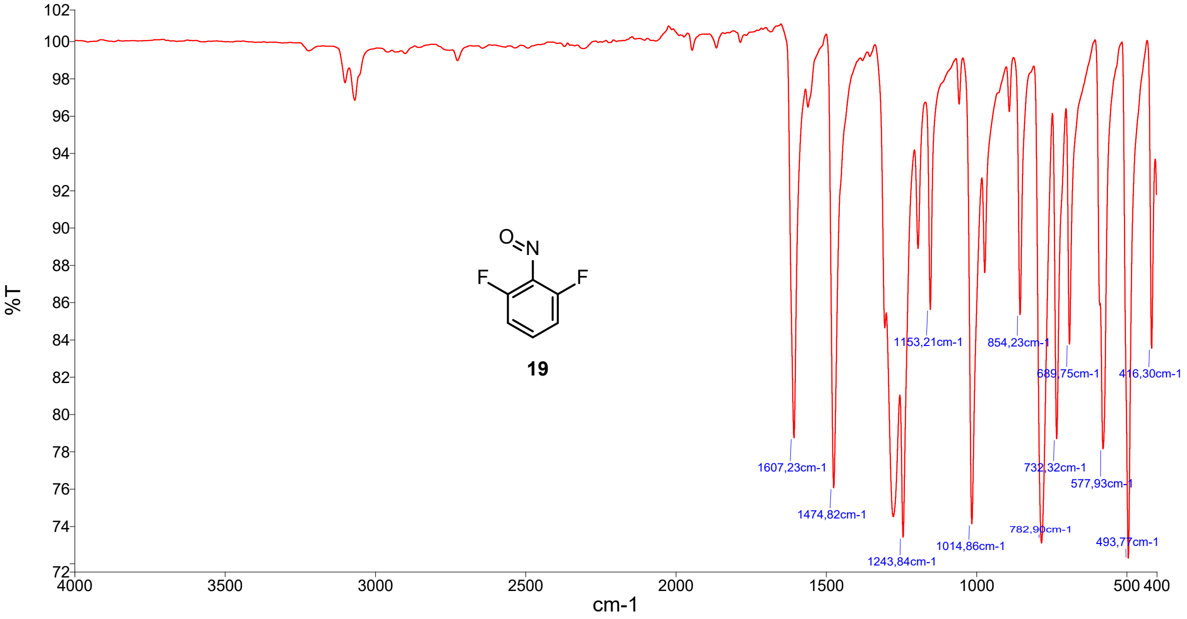

Figure S73. FT-IR spectrum of **19**.

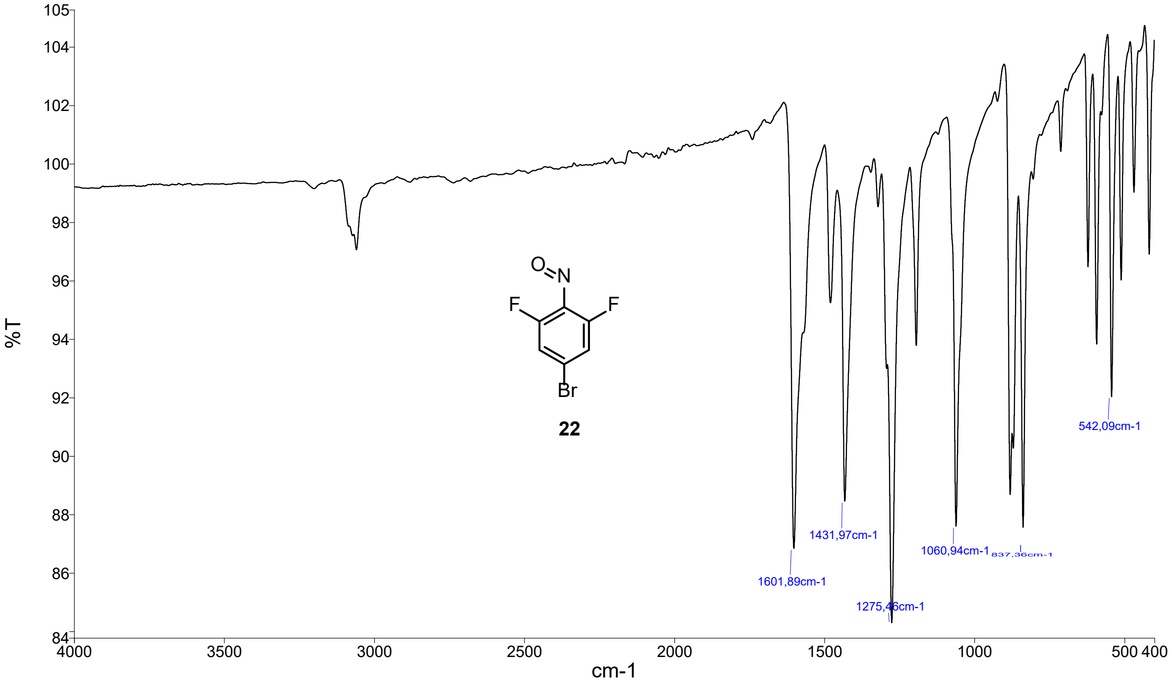

Figure S74. FT-IR spectrum of **22**.

5. HRMS data

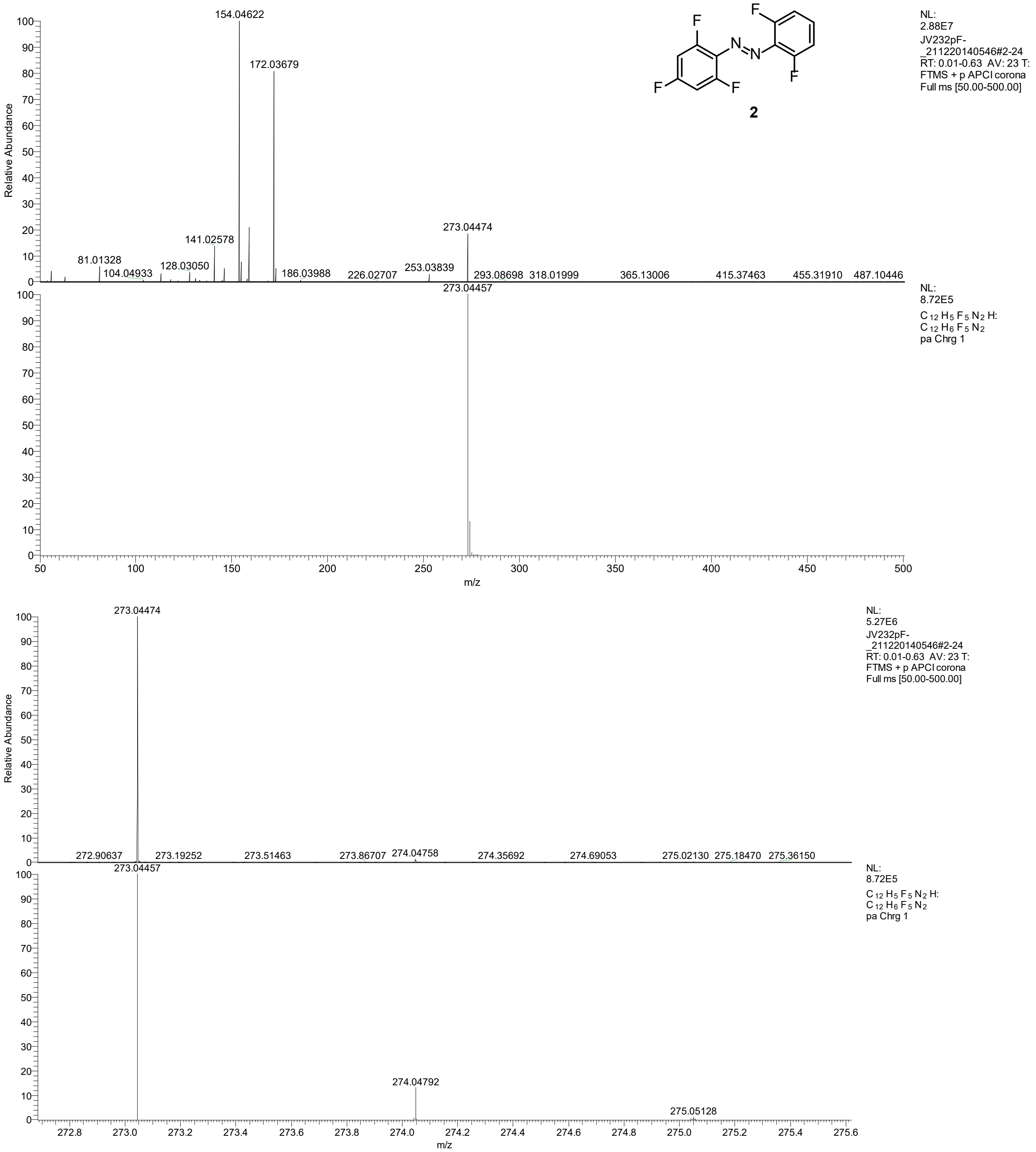

Figure S75. HRMS analysis for compound **2**.

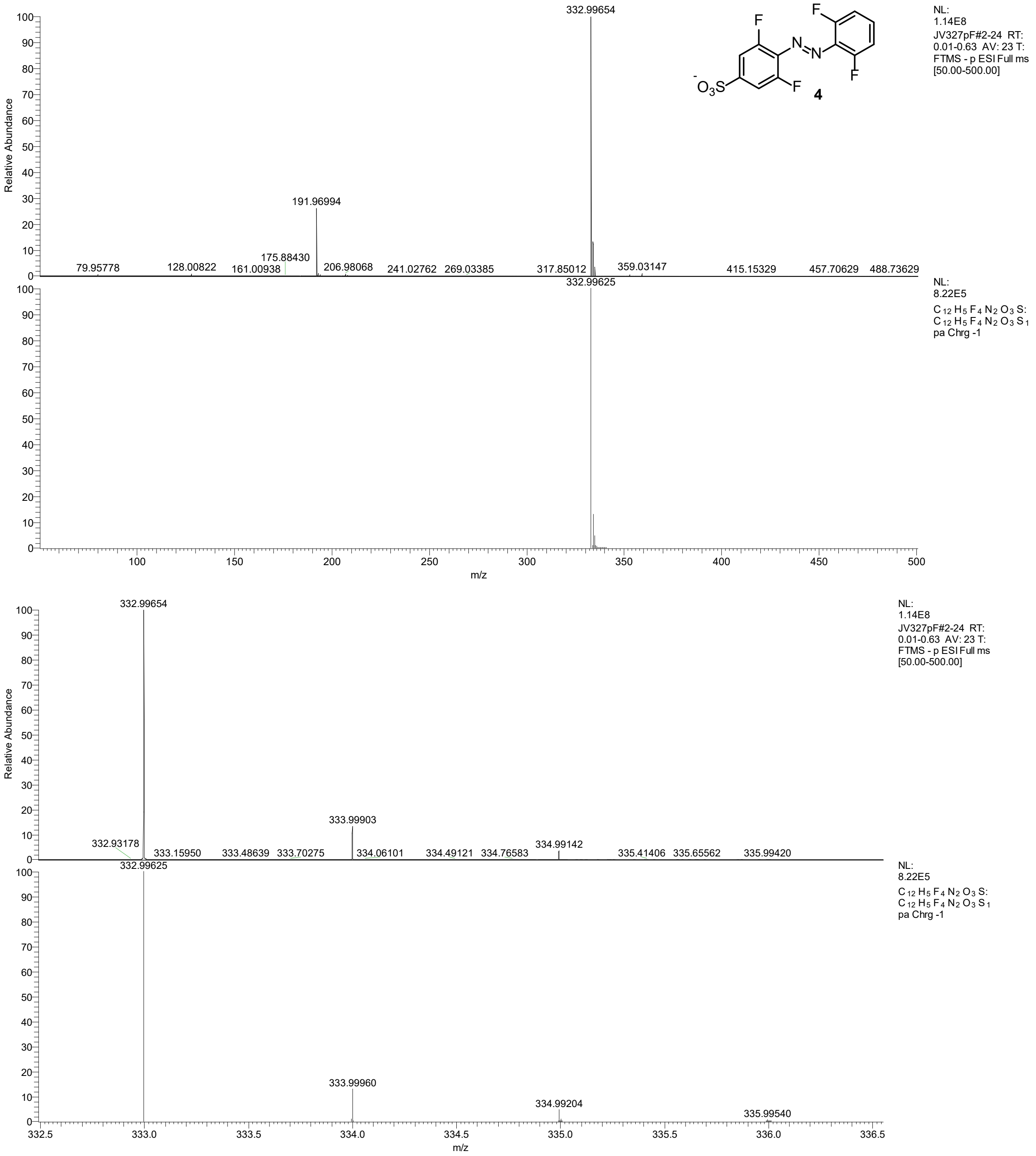

Figure S76. HRMS analysis for compound **4**.

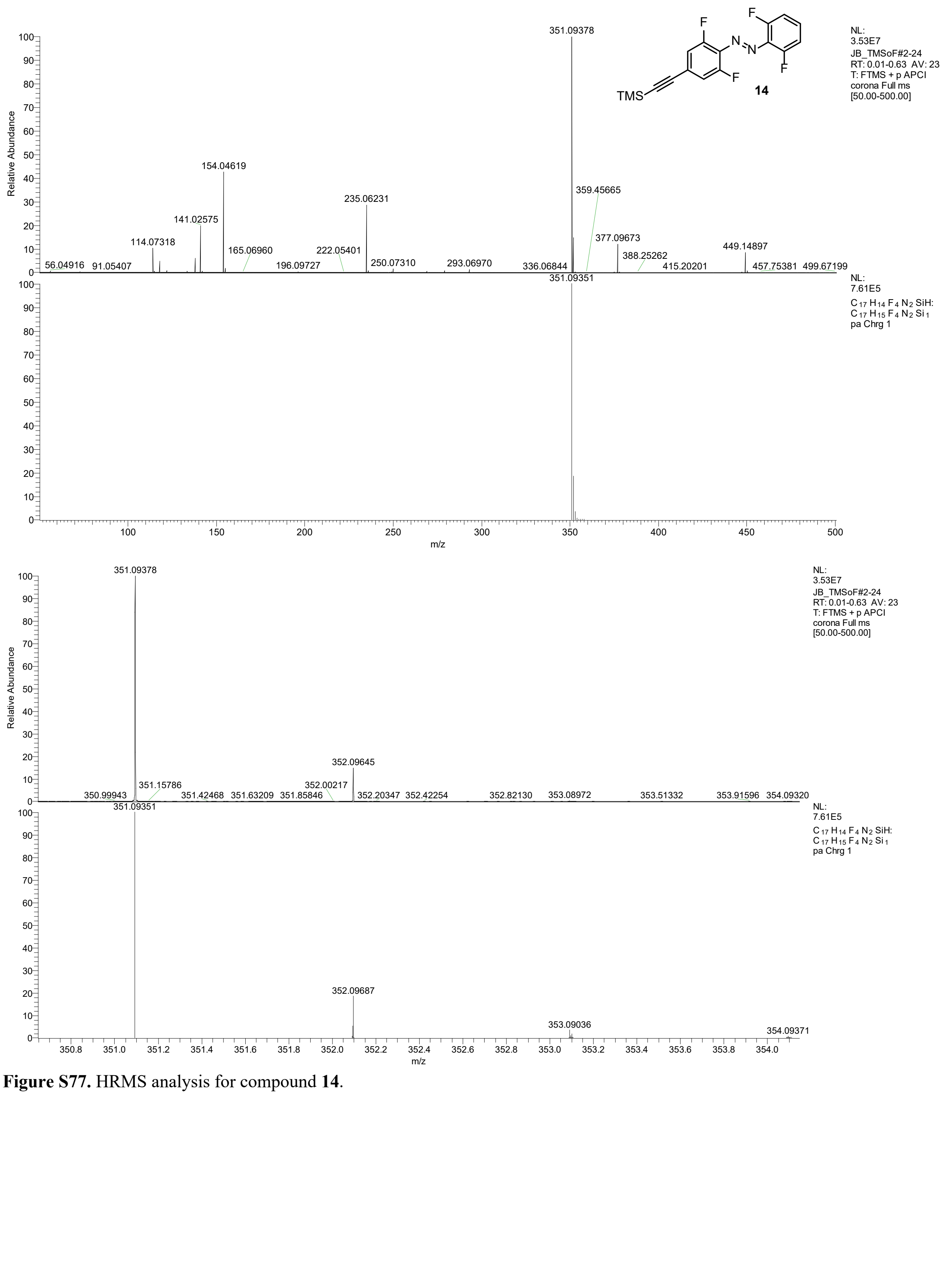

Figure S77. HRMS analysis for compound **14**.

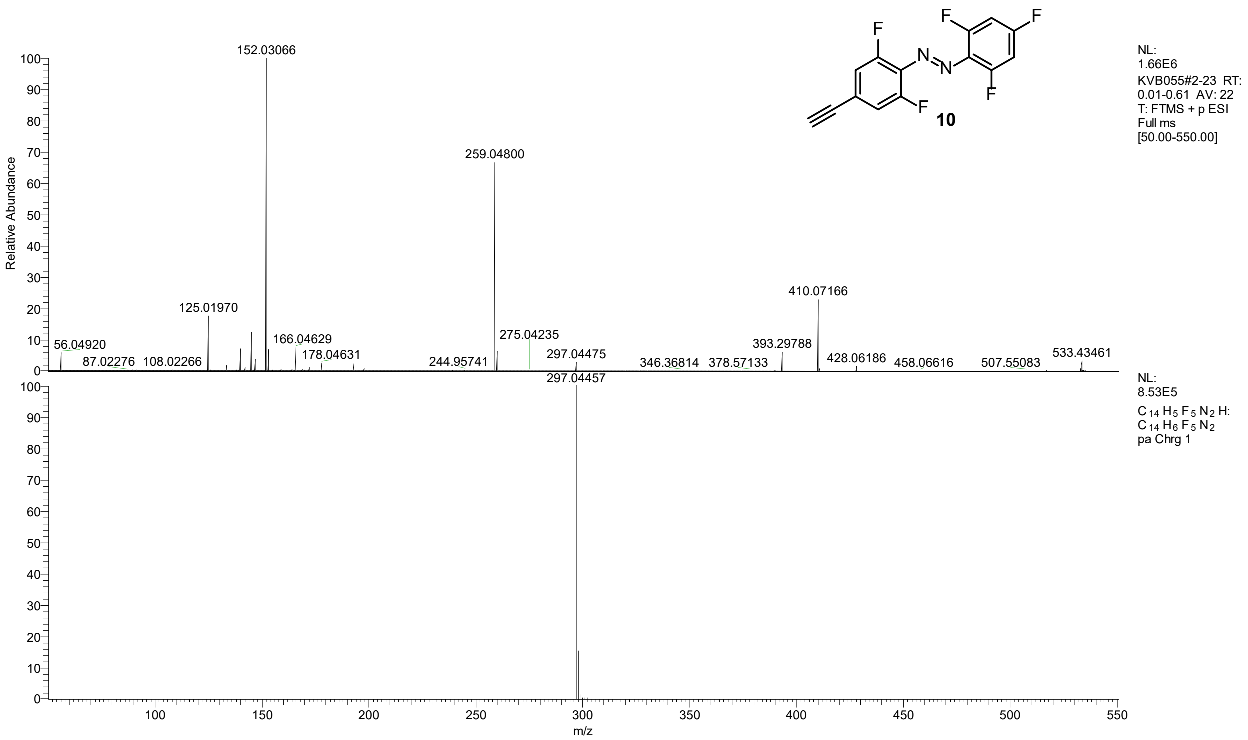

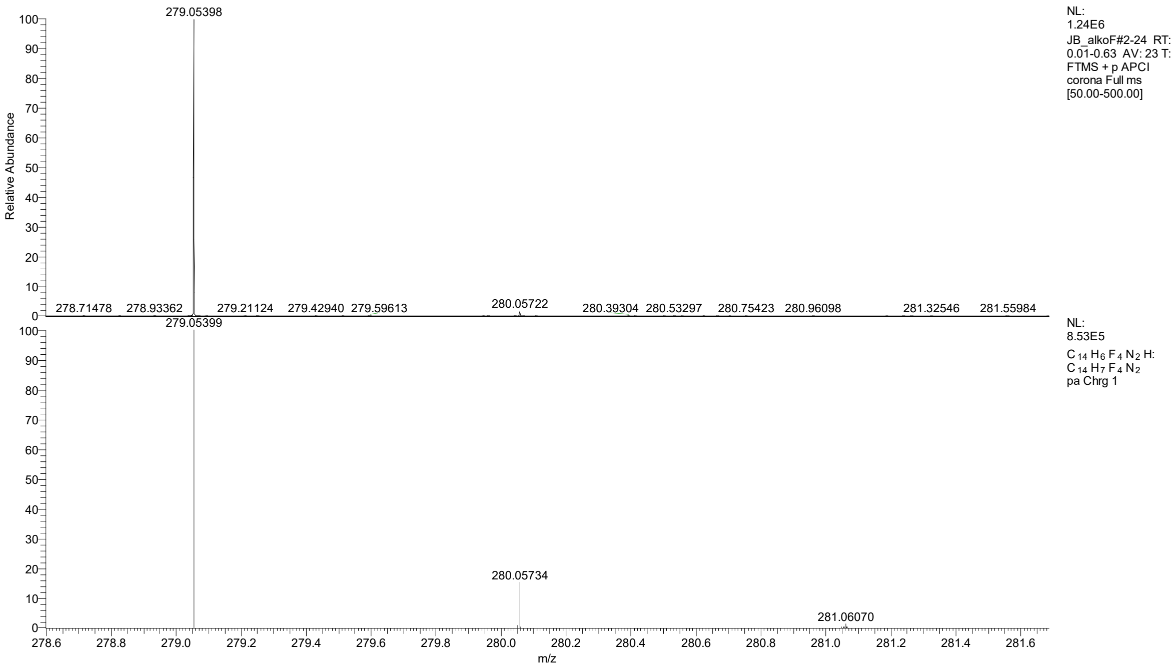

Figure S78. HRMS analysis for compound **10**.

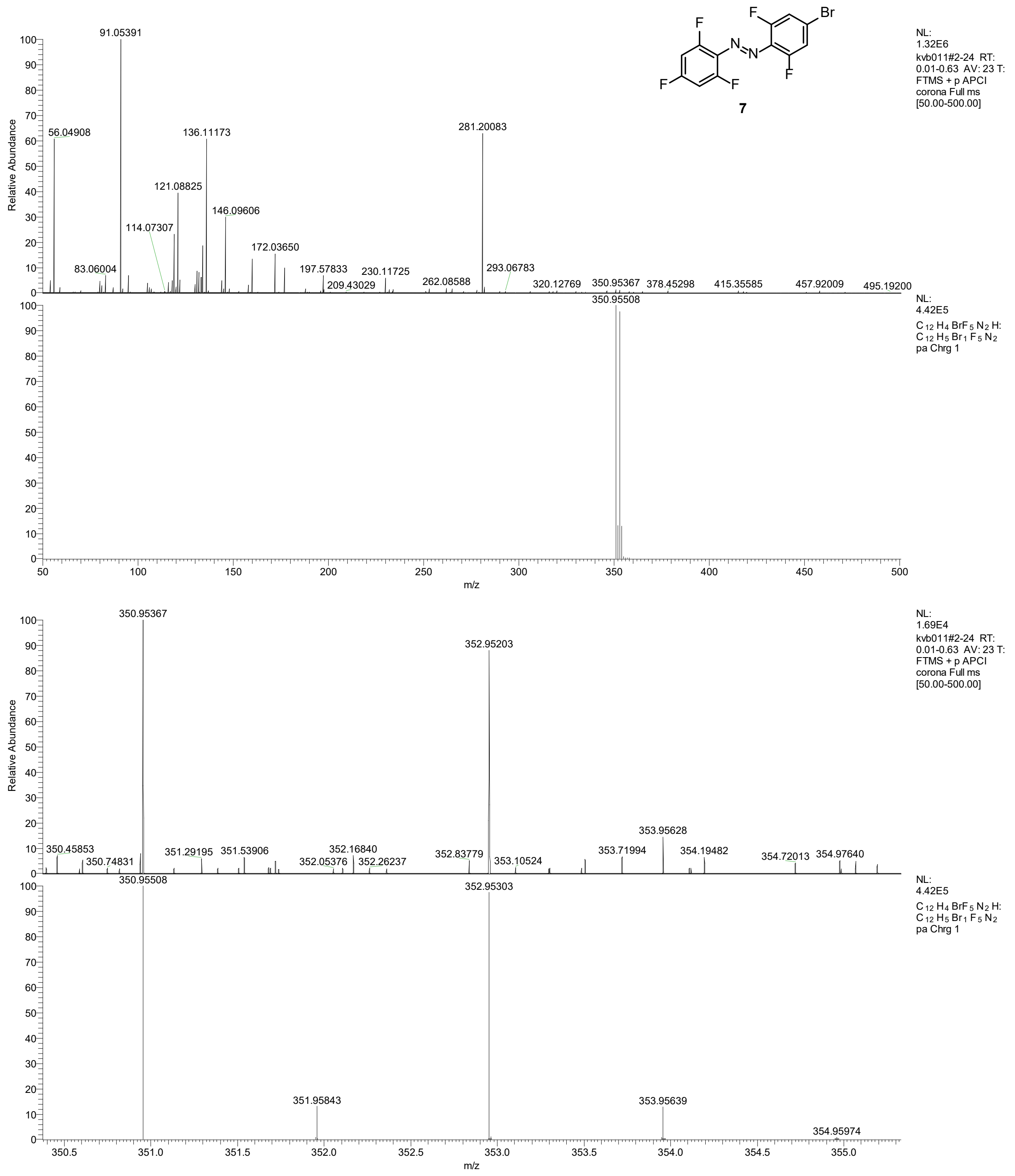

Figure S79. HRMS analysis for compound **7**.

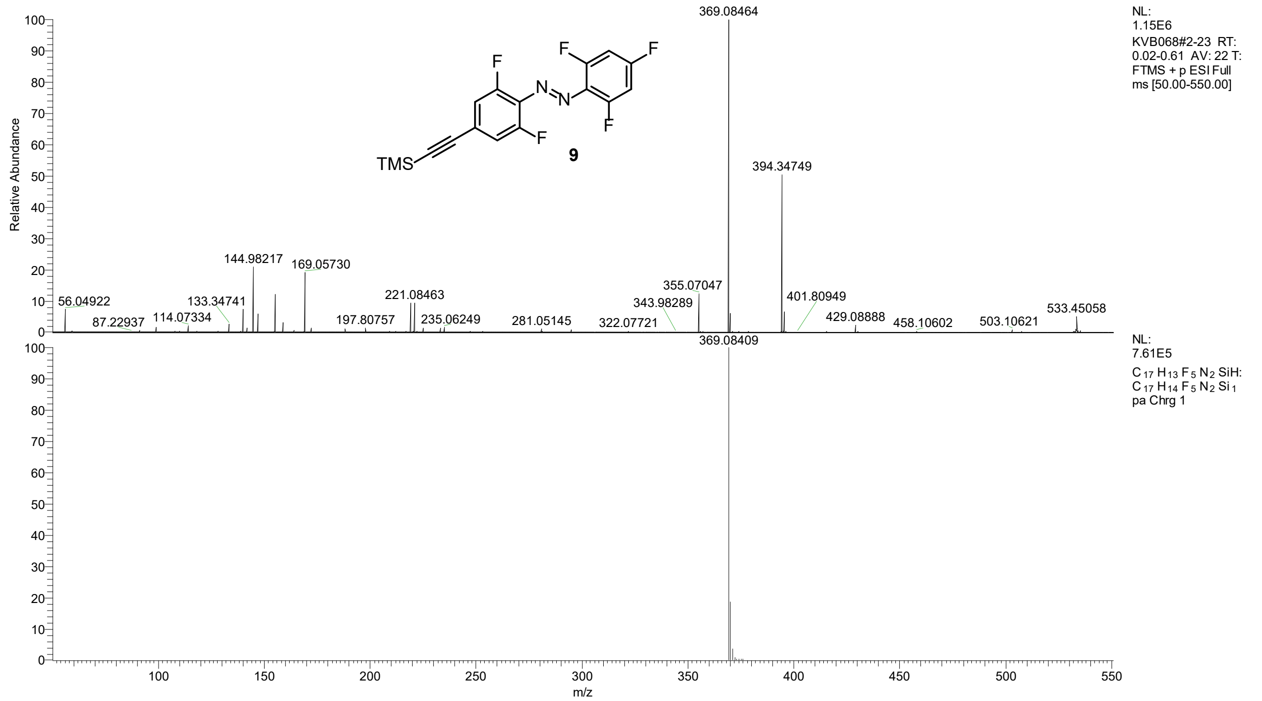

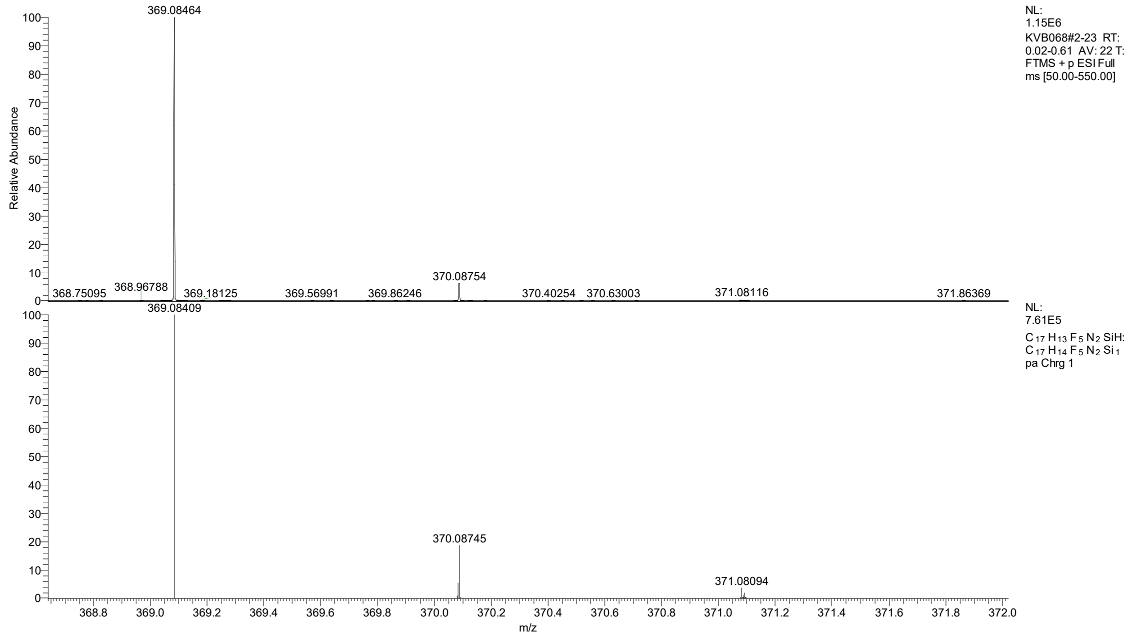

Figure S80. HRMS analysis for compound **9**.

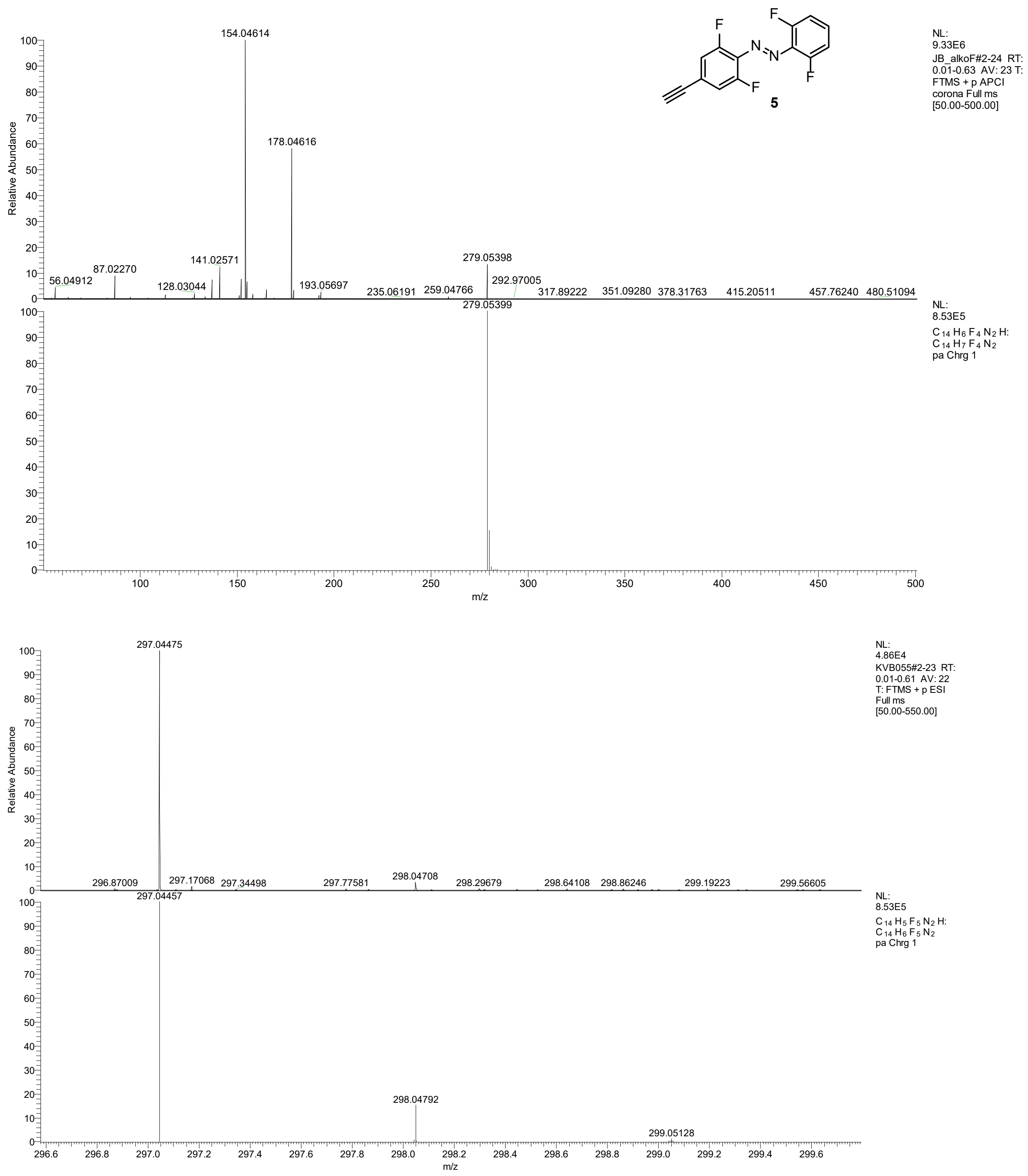

Figure S81. HRMS analysis for compound **5**.

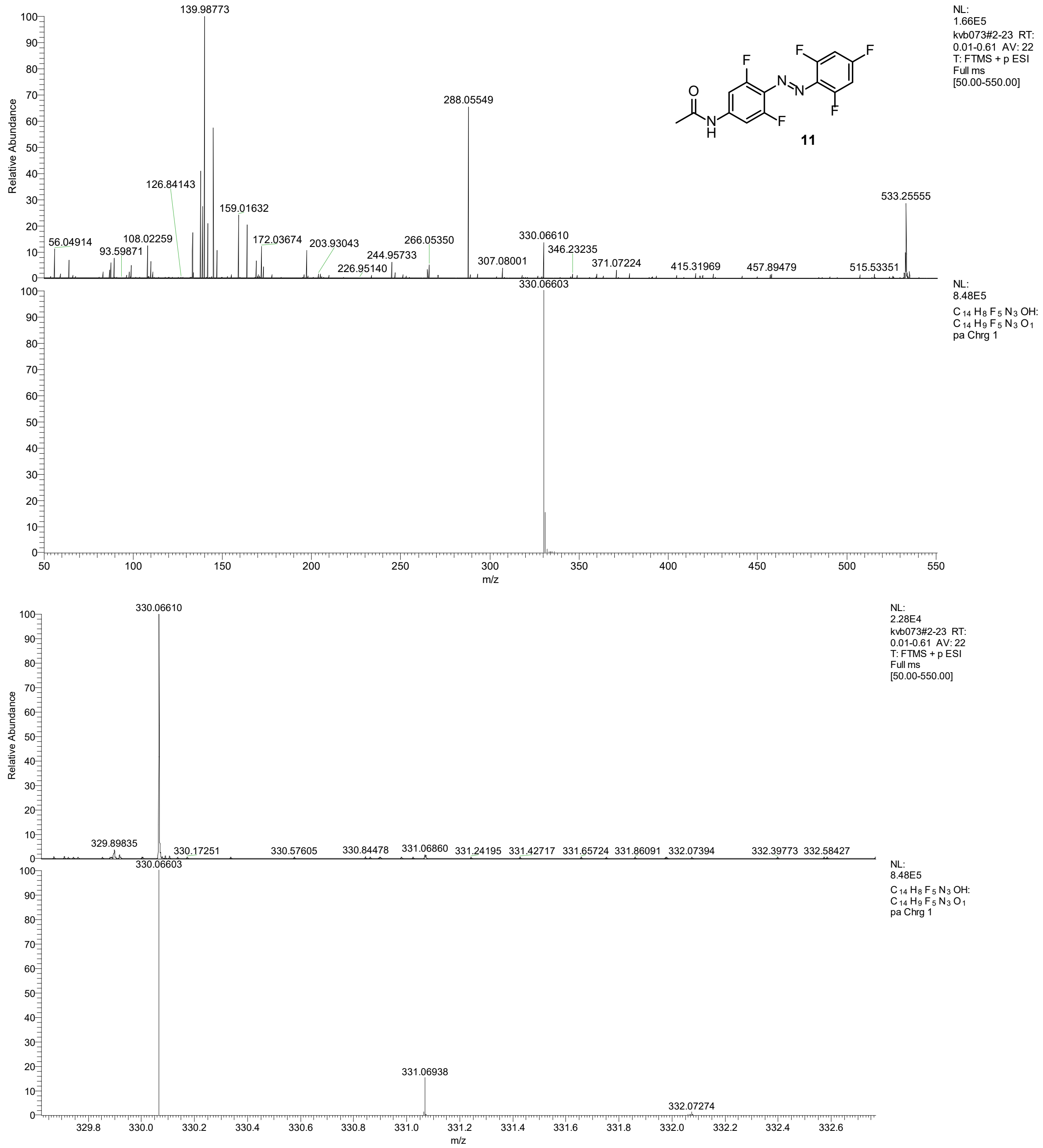

Figure S82. HRMS analysis for compound **11**.

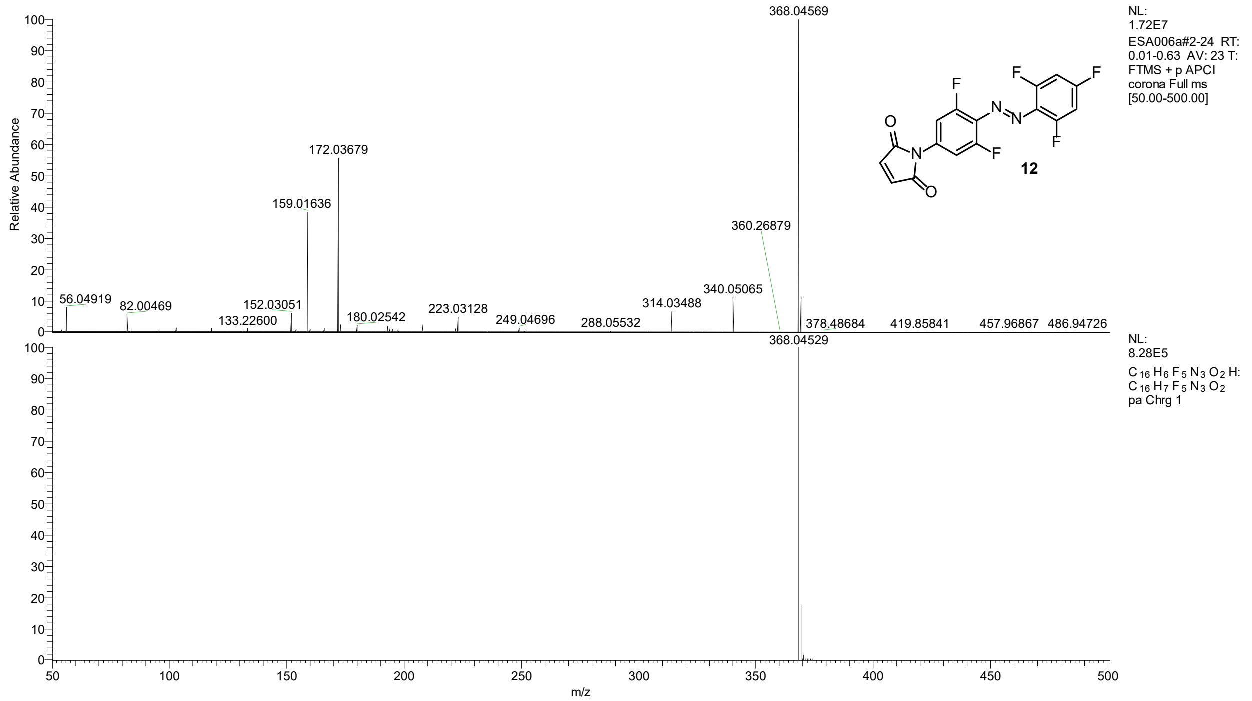

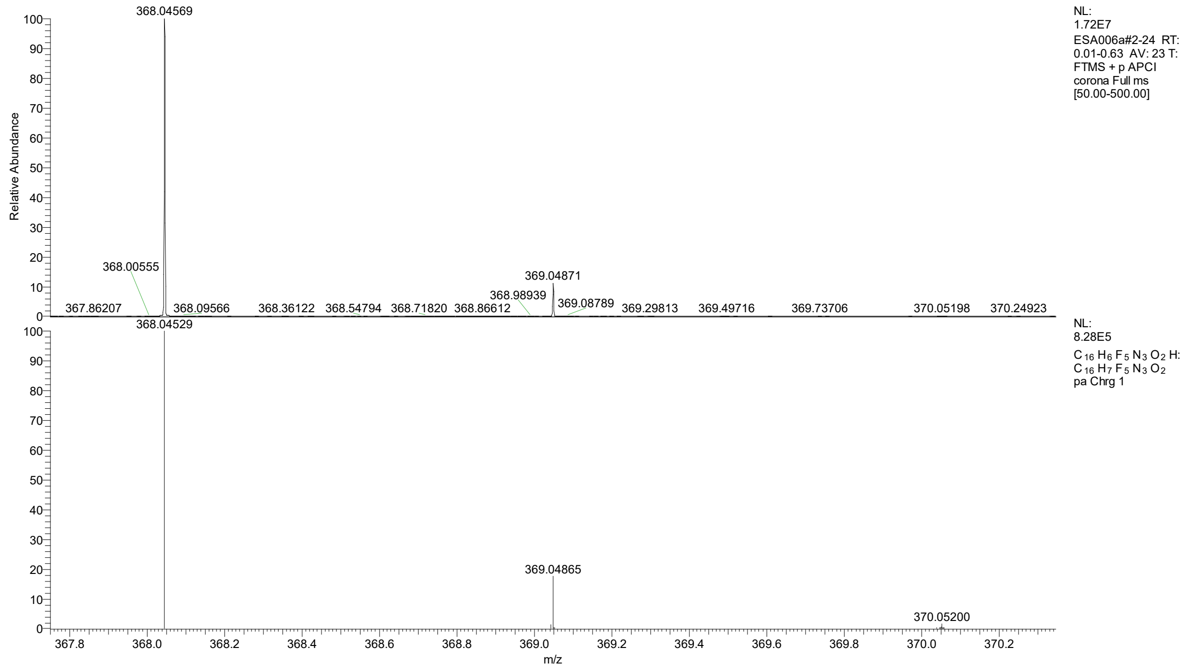

Figure S83. HRMS analysis for compound **12**.

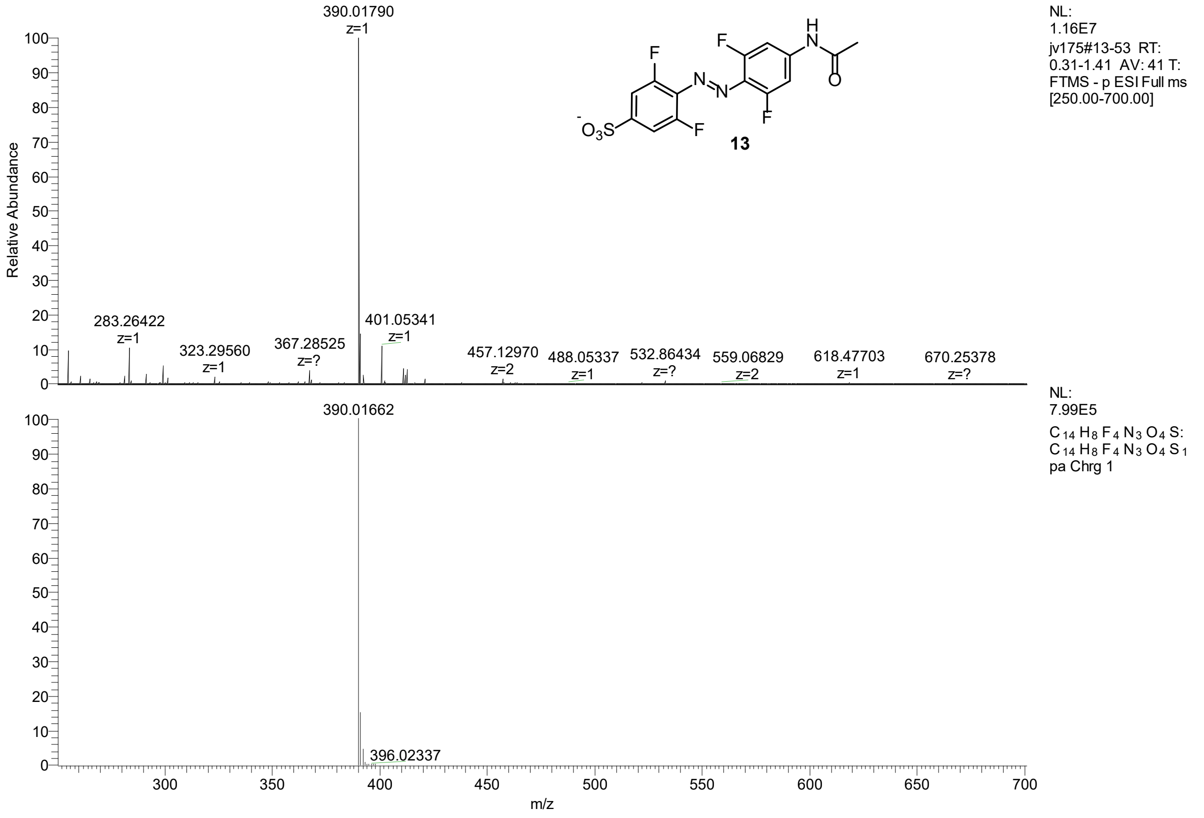

Figure S84. HRMS analysis of compound **13.**

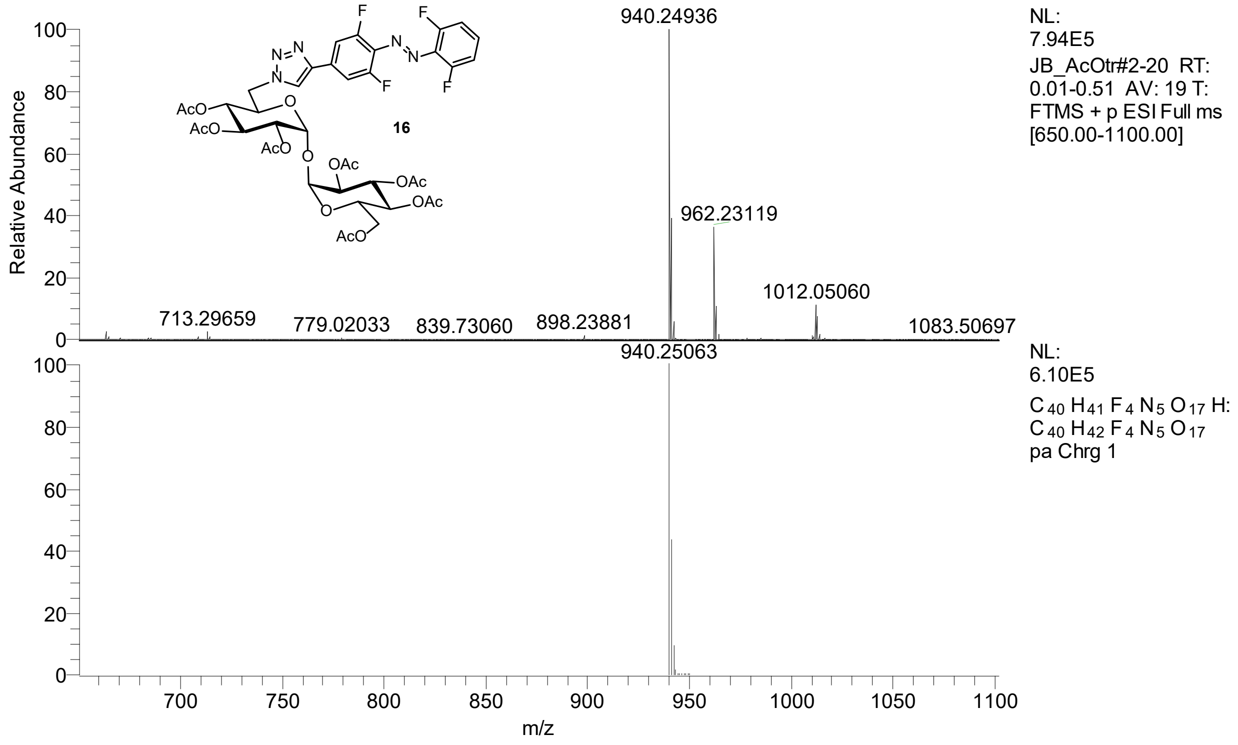

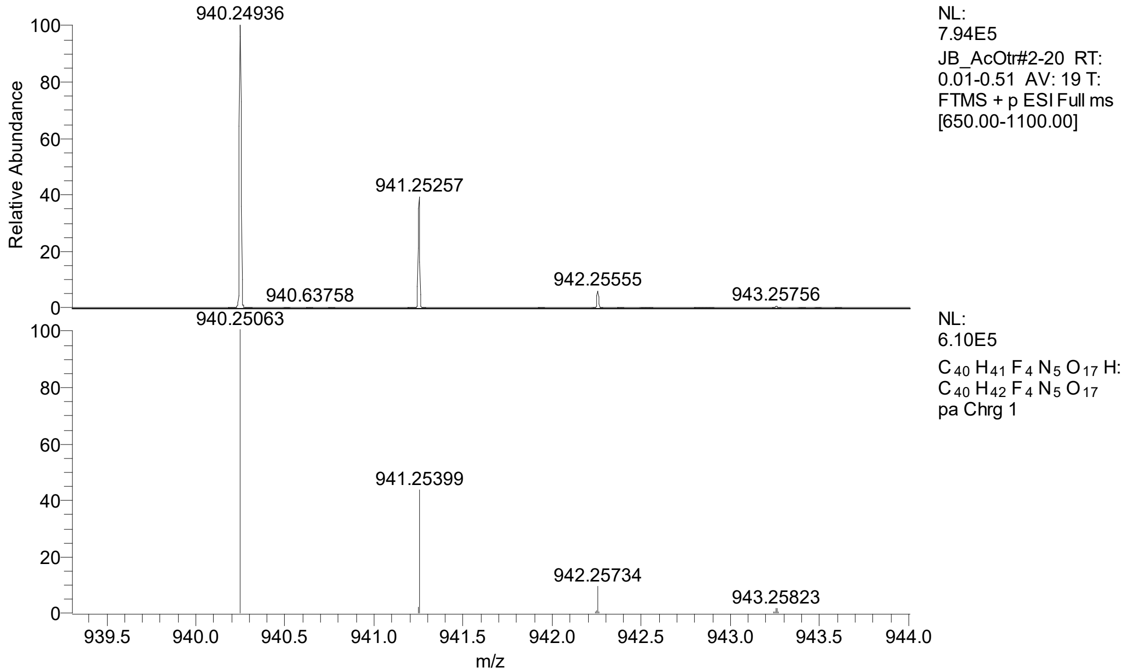

Figure S85. HRMS analysis for compound **16**.

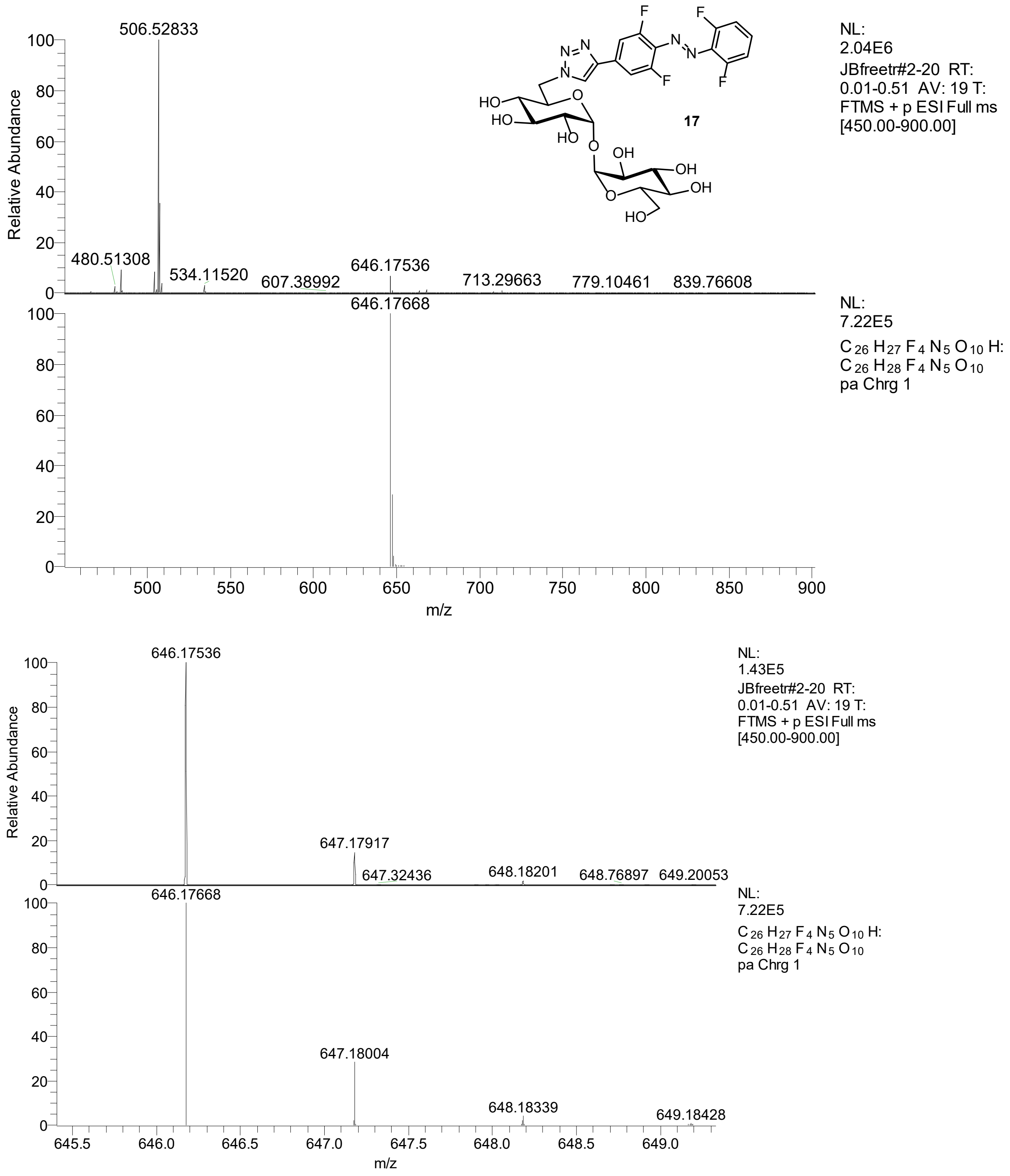

Figure S87. HRMS analysis for compound **17**.

6. Photochemical properties

The photochemical properties of each compound were determined both in organic solvent as standard (DMSO) and in an aqueous buffer (PBS). Available LED lamps with wavelengths closest to where the largest difference in absorbance of the two isomers was, namely 530 nm (green) and 430 nm (blue), were used for irradiation of the visible light-responsive azobenzenes.

The PSDs upon irradiation with 530 nm or 430 nm were observed by ^{19}F NMR spectroscopy as follows: Solutions of known concentrations were prepared with DMSO-d_6 or for aqueous conditions with 10% D_2O in PBS buffer and transferred into NMR tubes. The samples were irradiated with the respective LED lamp until no change in the spectra was observed thus confirming that the samples reached PSS. The PSD was determined based on relative integration of peaks from both isomers.

UV-Vis spectroscopy was used to determine the remaining photochemical properties as follows: Stock solutions of the azobenzene compounds were prepared in DMSO or PBS buffer and the absorption coefficient was determined by measuring the UV-Vis spectrum at different concentrations. Next the absorbance at the absorption maximum was plotted against the concentration thus giving the molar absorption coefficient as the slope of the linearly (y=ax+b) fitted curve.
The stability and resistance of the compounds towards fatigue was determined by tracking the absorbance change at the absorbance maximum upon several irradiation cycles. The same irradiation cycles experiments were performed in the presence of 10 mM glutathione to confirm stability of the azobenzenes towards reduction.

Lastly, the half-lives were determined by tracking the absorbance change at λ_{max} at a defined temperature and solvents. The change was plotted against time and the resulting first-order kinetics data were fitted to an exponential curve ($y=a*e^{k*x}$), to give the half-life of the *cis* to *trans* thermal isomerization process via t1/2=ln2/k. Since the half-lives of most tested compounds were much longer than feasibly measurable with our setup, the half-lives were given as a range of >X, where X represents the lowest estimate of the result of the exponential fit at 37 °C.

6.1 Photochemical properties in DMSO

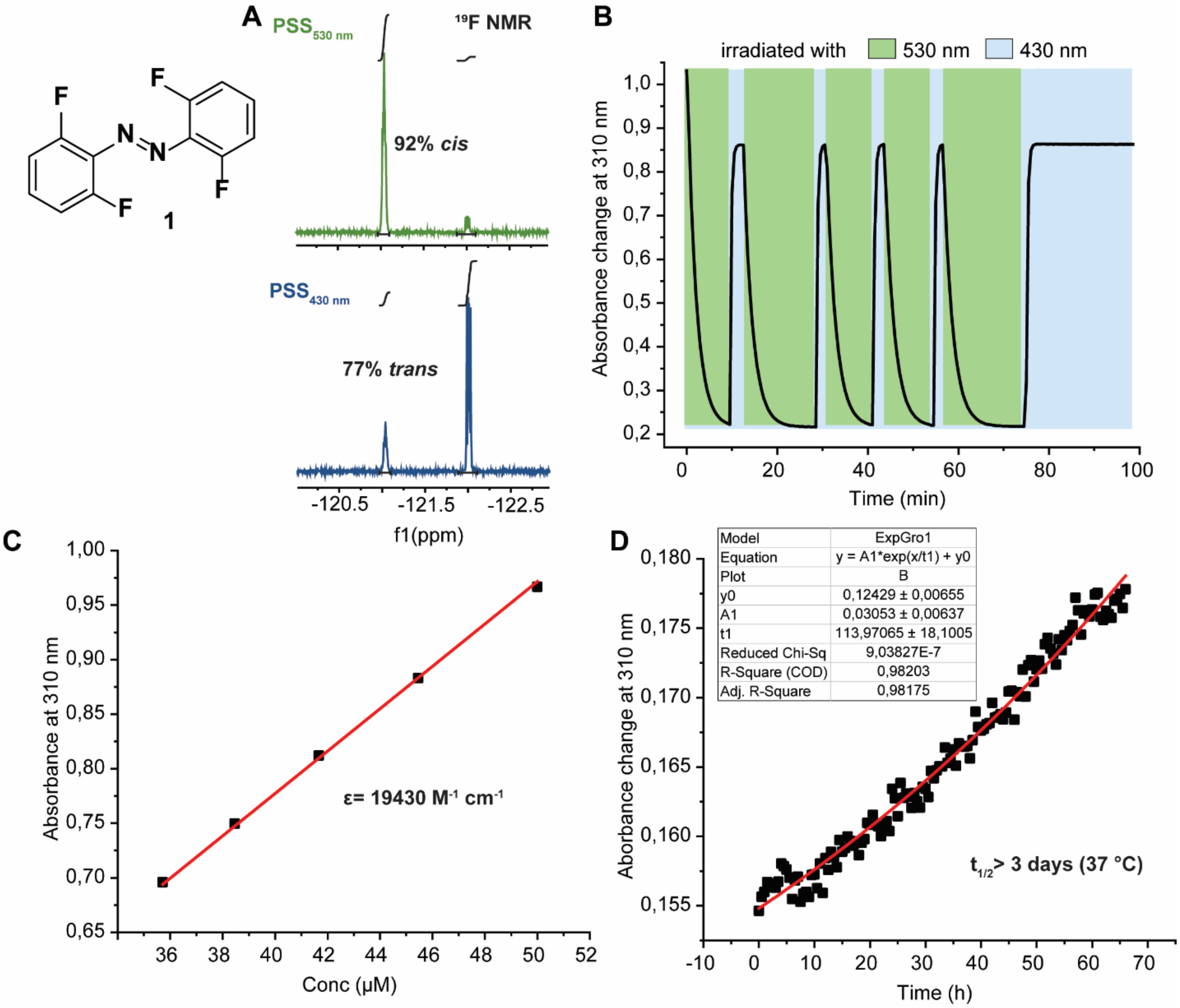

Figure S88. Determination of the photochemical properties of compound **1**. **A** PSS determination by ^{19}F NMR spectroscopy upon irradiation with 530 nm and 430 nm LED at rt, 4 mM in DMSO-d_6. **B** Fatigue resistance test upon irradiation with 530 and 430 nm LED in DMSO at 50 µM and 20 °C. **C** Determination of the molar absorptivity in DMSO at 20 °C. **D** Half-life determination in DMSO at 50 µM and 37 °C.

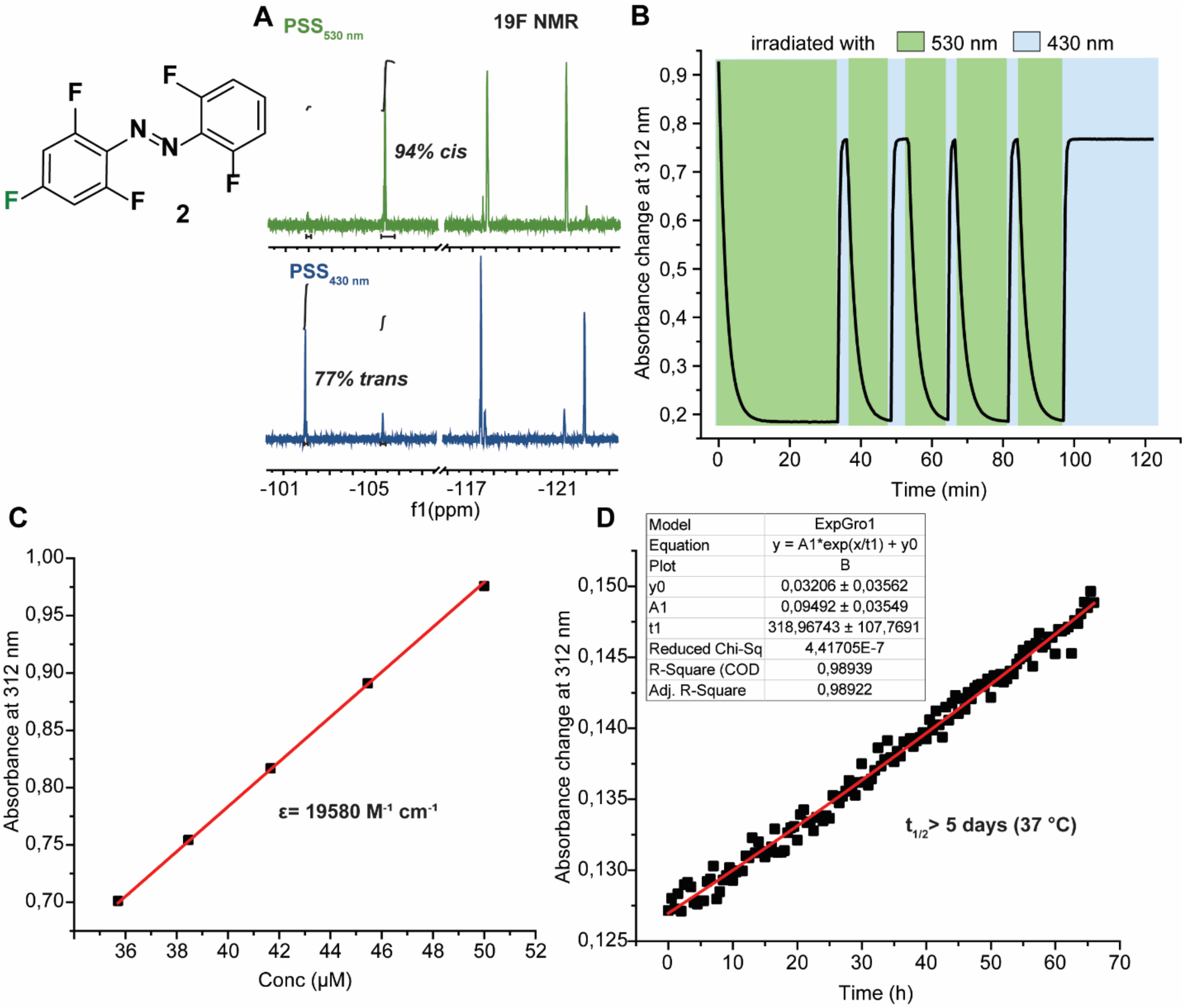

Figure S89. Determination of the photochemical properties of compound **2**. **A** PSS determination by ^{19}F NMR spectroscopy upon irradiation with 530 nm and 430 nm LED at rt, 4 mM in DMSO-*d*$_6$. **B** Fatigue resistance test upon irradiation with 530 and 430 nm LED in DMSO at 50 µM and 20 °C. **C** Determination of the molar absorptivity in DMSO at 20 °C. **D** Half-life determination in DMSO at 50 µM and 37 °C.

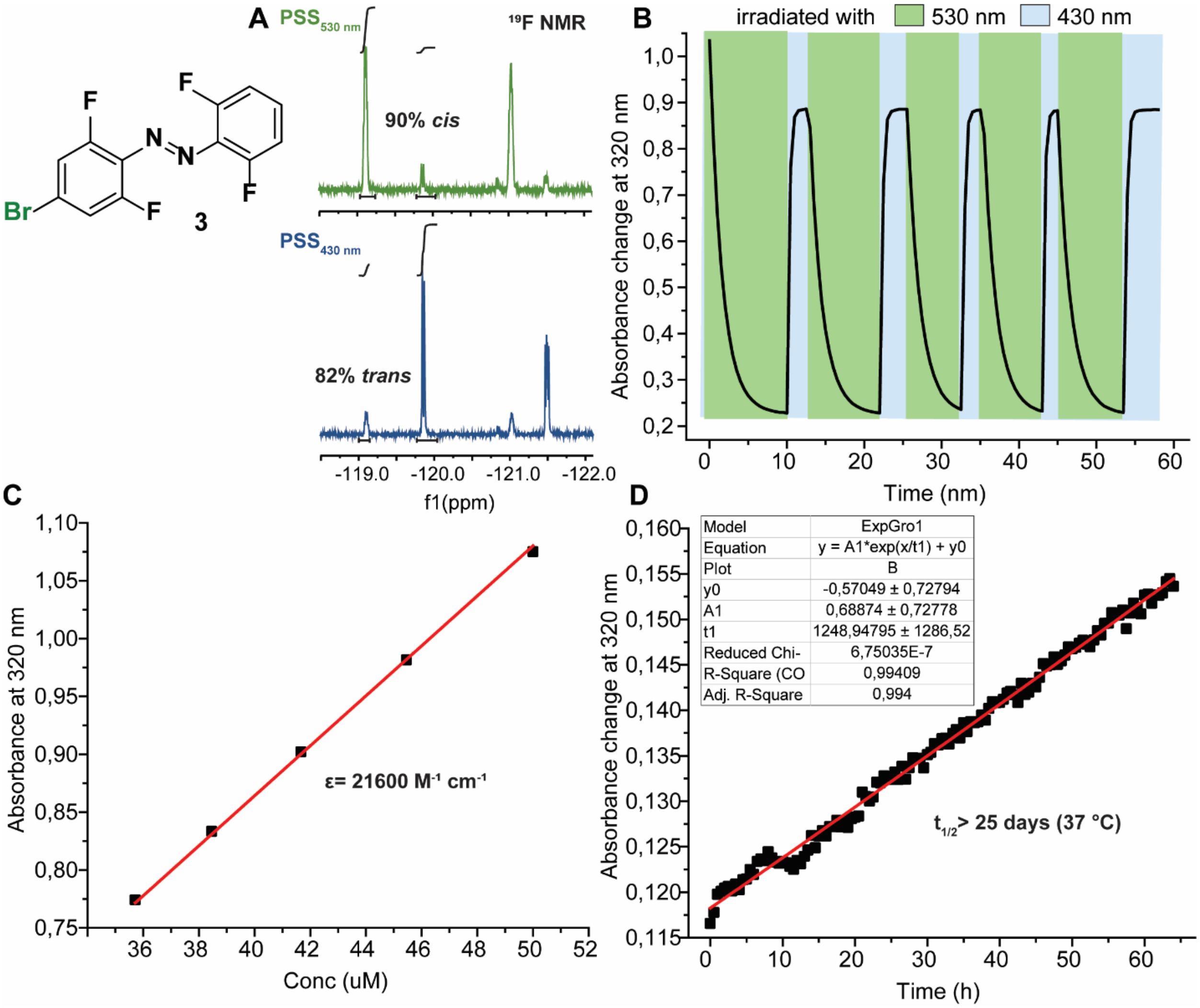

Figure S90. Determination of the photochemical properties of compound **3**. **A** PSS determination by ^{19}F NMR spectroscopy upon irradiation with 530 nm and 430 nm LED at rt, 4 mM in DMSO-d_6. **B** Fatigue resistance test upon irradiation with 530 and 430 nm LED in DMSO at 50 µM and 20 °C. **C** Determination of the molar absorptivity in DMSO at 20 °C. **D** Half-life determination in DMSO at 50 µM and 37 °C.

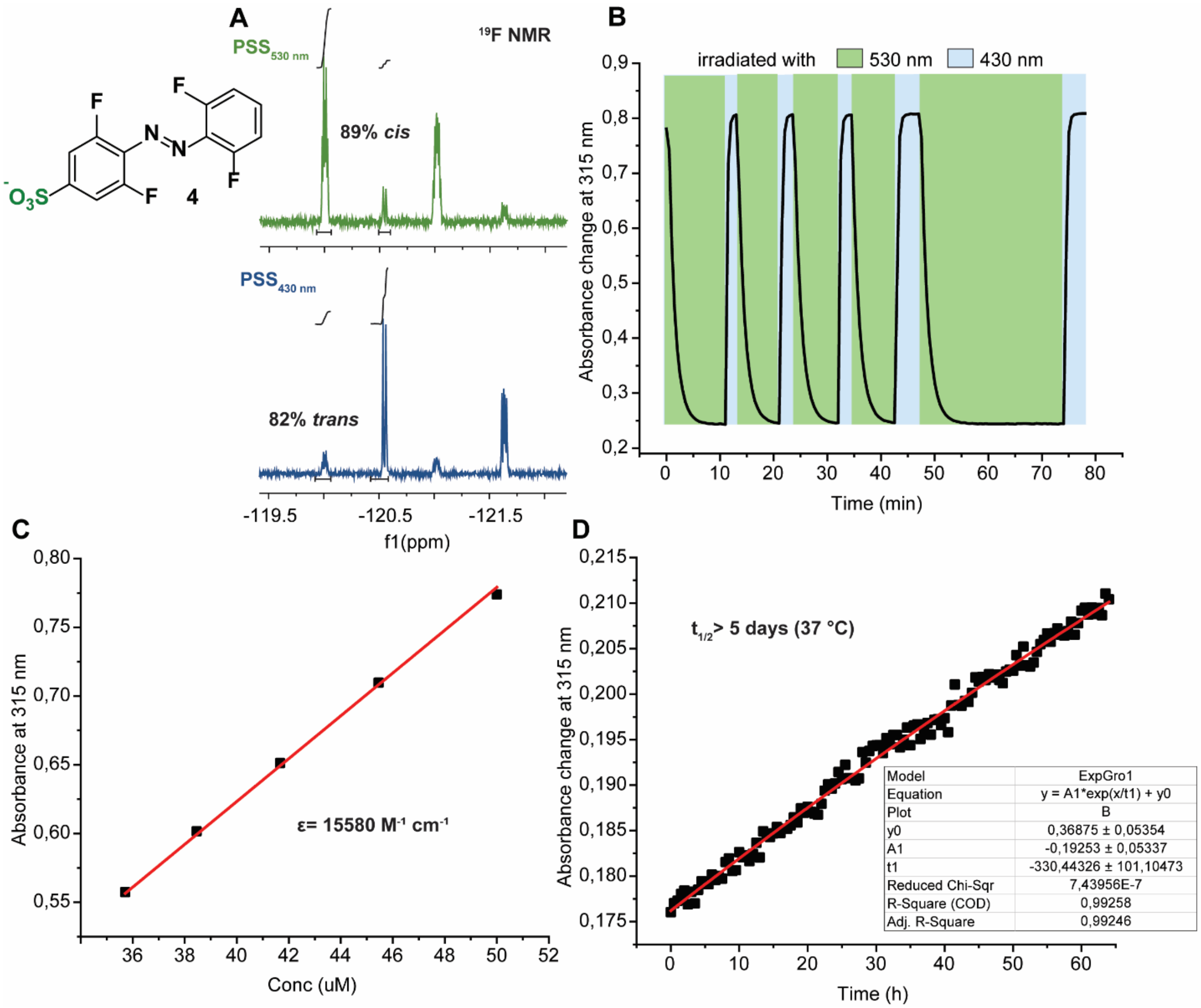

Figure S91. Determination of the photochemical properties of compound **4**. **A** PSS determination by ^{19}F NMR spectroscopy upon irradiation with 530 nm and 430 nm LED at rt, 2 mM in DMSO-d_6. **B** Fatigue resistance test upon irradiation with 530 and 430 nm LED in DMSO at 50 µM and 20 °C. **C** Determination of the molar absorptivity in DMSO at 20 °C. **D** Half-life determination in DMSO at 50 µM and 37 °C.

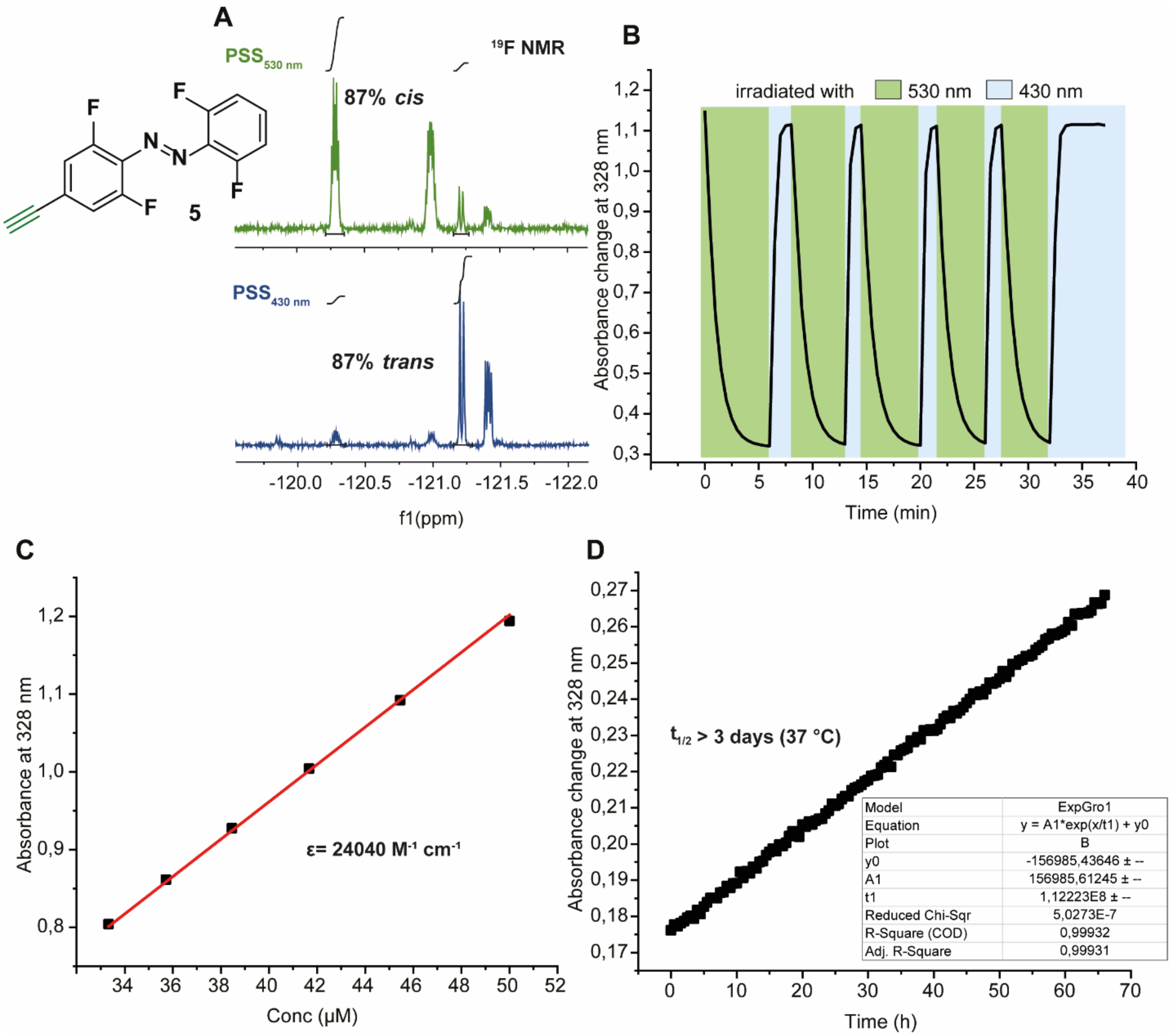

Figure S92. Determination of the photochemical properties of compound **5**. **A** PSS determination by ^{19}F NMR spectroscopy upon irradiation with 530 nm and 430 nm LED at rt, 4 mM in DMSO-d_6. **B** Fatigue resistance test upon irradiation with 530 and 430 nm LED in DMSO at 50 µM and 20 °C. **C** Determination of the molar absorptivity in DMSO at 20 °C. **D** Half-life determination in DMSO at 50 µM and 37 °C.

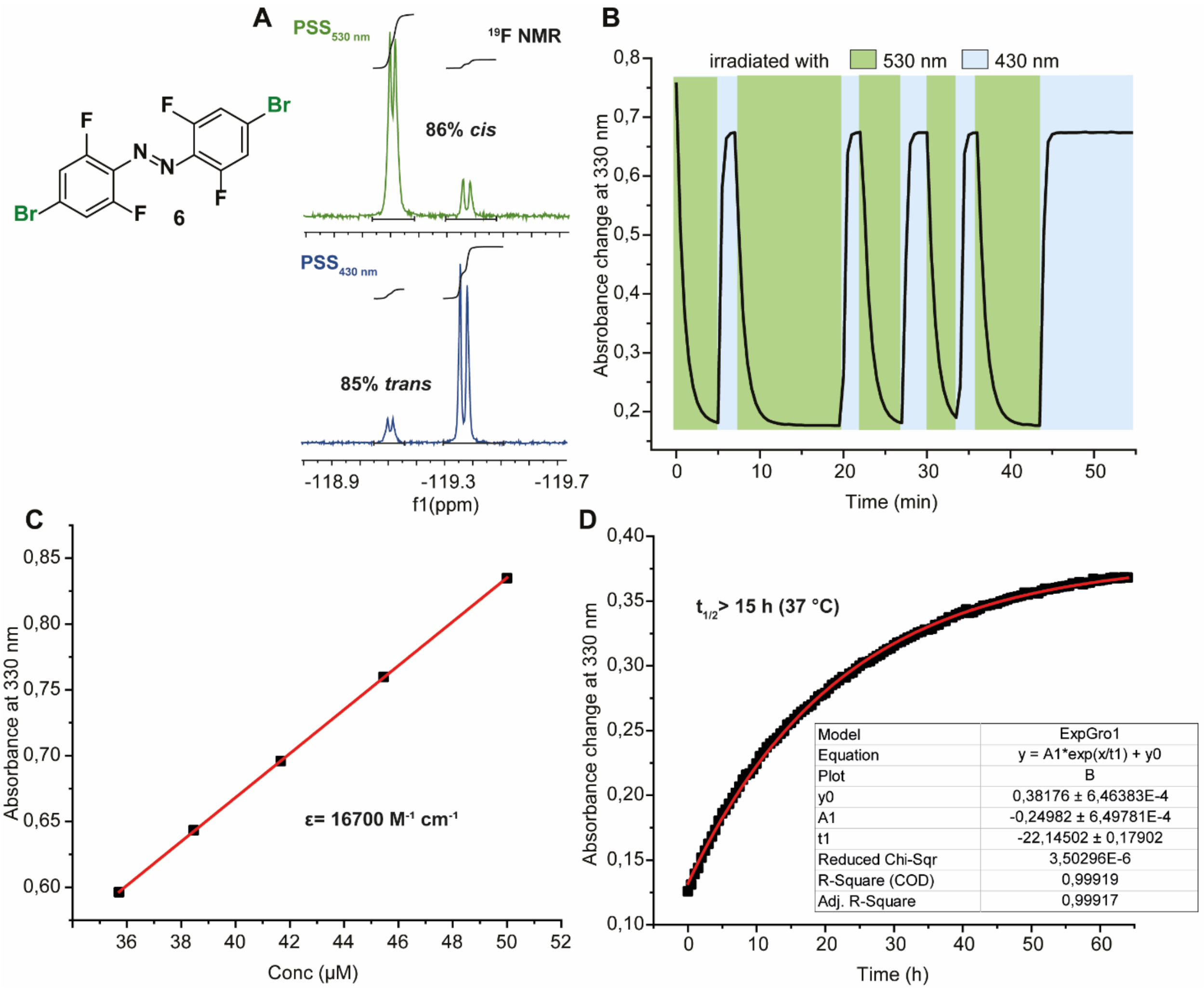

Figure S93. Determination of the photochemical properties of compound **6**. **A** PSS determination by ^{19}F NMR spectroscopy upon irradiation with 530 nm and 430 nm LED at rt, 4 mM in DMSO-d_6. **B** Fatigue resistance test upon irradiation with 530 and 430 nm LED in DMSO at 50 µM and 20 °C. **C** Determination of the molar absorptivity in DMSO at 20 °C. **D** Half-life determination in DMSO at 50 µM and 37 °C.

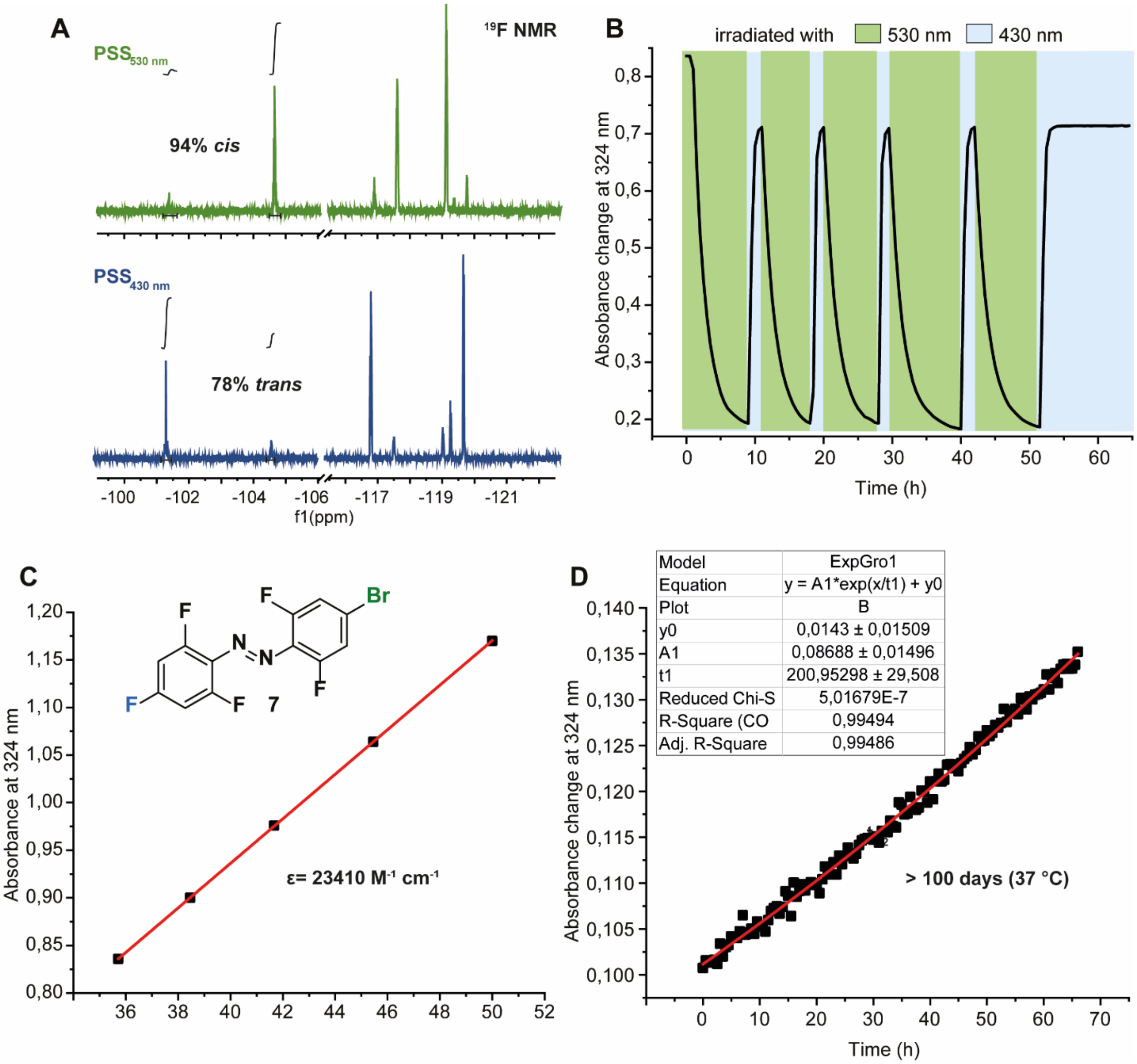

Figure S94. Determination of the photochemical properties of compound **7**. **A** PSS determination by ^{19}F NMR spectroscopy upon irradiation with 530 nm and 430 nm LED at rt, 4 mM in DMSO-d_6. **B** Fatigue resistance test upon irradiation with 530 and 430 nm LED in DMSO at 50 µM and 20 °C. **C** Determination of the molar absorptivity in DMSO at 20 °C. **D** Half-life determination in DMSO at 50 µM and 37 °C.

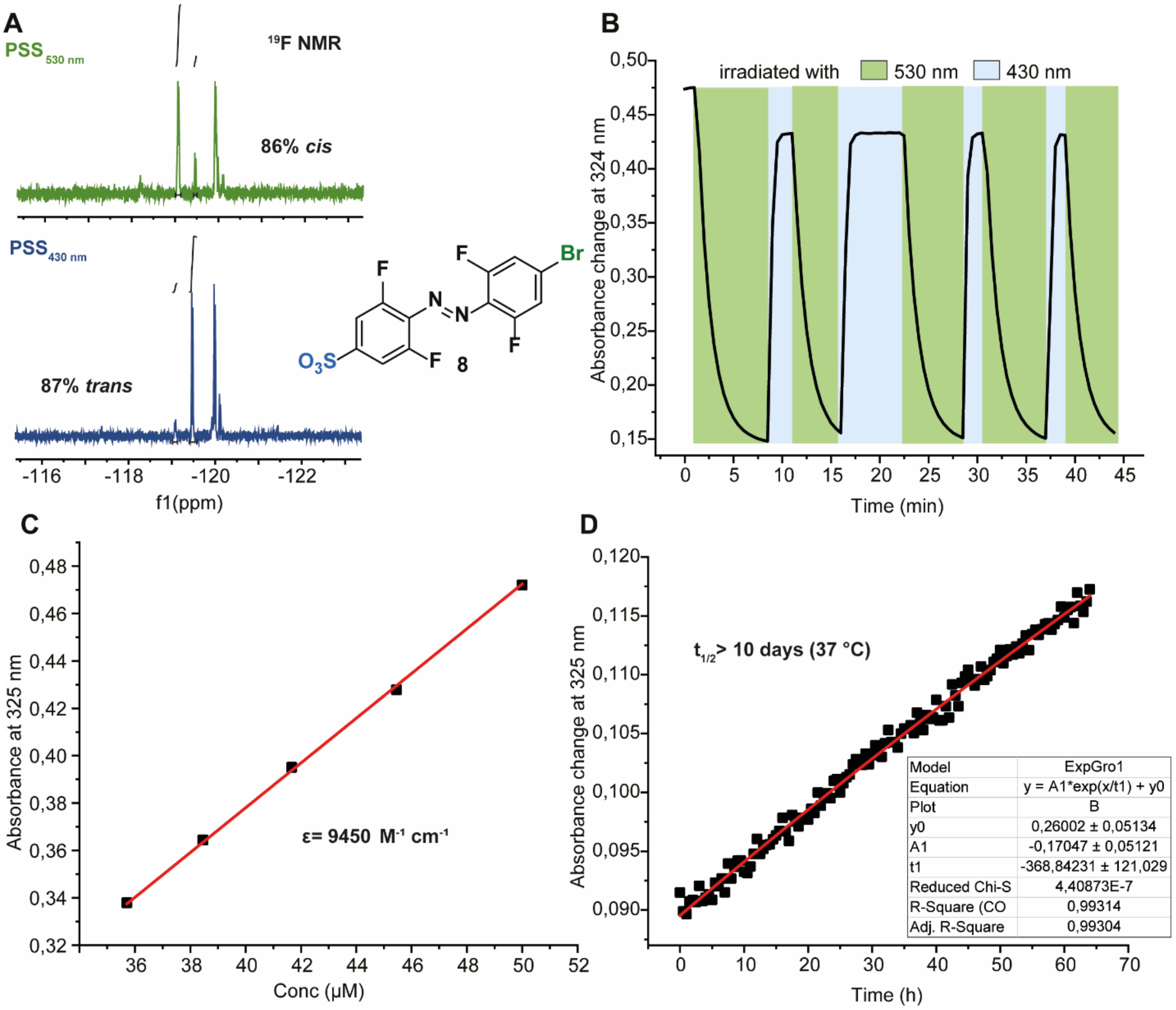

Figure S95. Determination of the photochemical properties of compound **8**. **A** PSS determination by ^{19}F NMR spectroscopy upon irradiation with 530 nm and 430 nm LED at rt, 2 mM in DMSO-d_6. **B** Fatigue resistance test upon irradiation with 530 and 430 nm LED in DMSO at 50 μM and 20 °C. **C** Determination of the molar absorptivity in DMSO at 20 °C. **D** Half-life determination in DMSO at 50 μM and 37 °C.

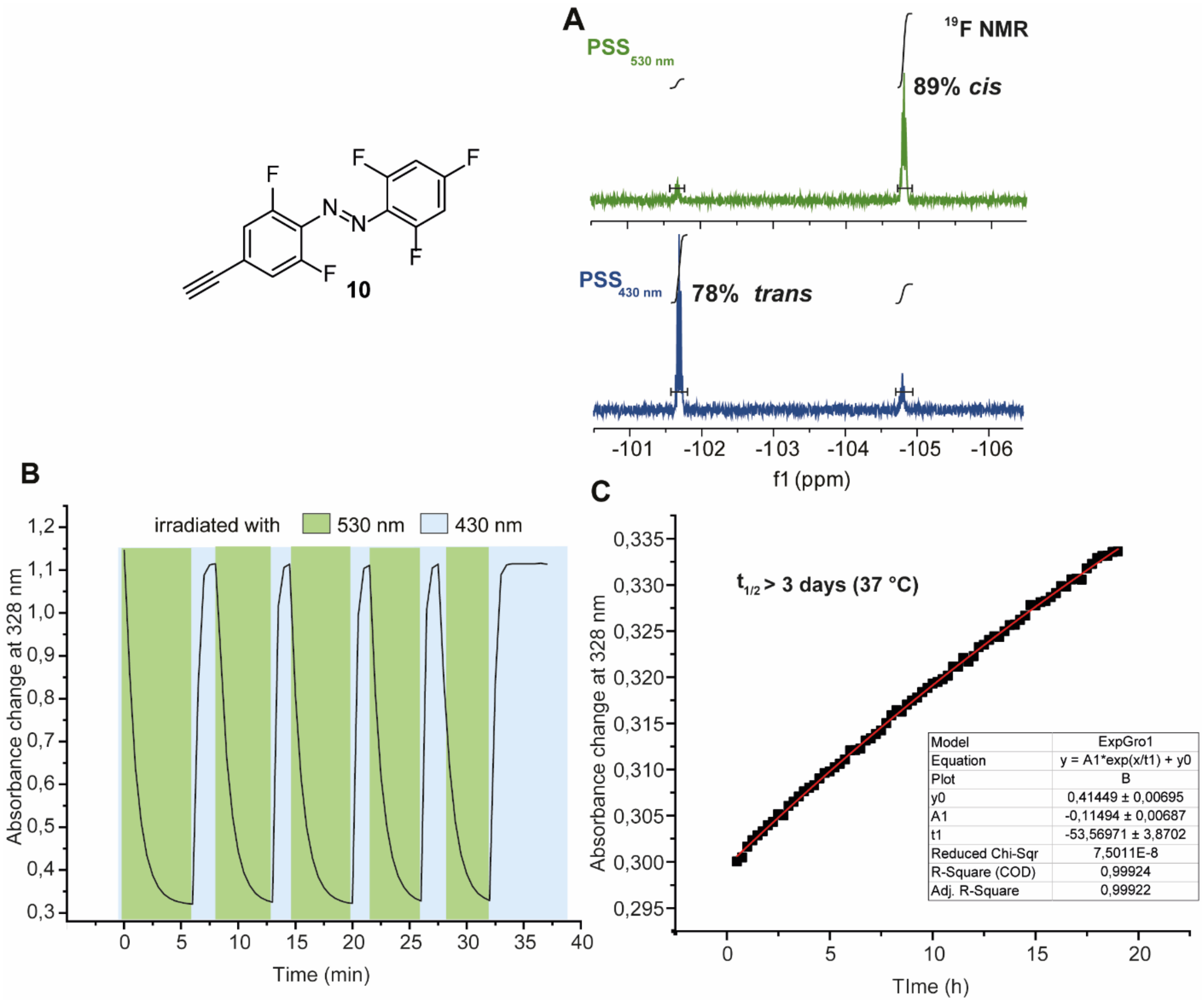

Figure S96. Determination of the photochemical properties of compound **10**. **A** PSS determination by ^{19}F NMR spectroscopy upon irradiation with 530 nm and 430 nm LED at rt, 5 mM in DMSO-d_6. **B** Fatigue resistance test upon irradiation with 530 and 430 nm LED in DMSO at 50 µM and 20 °C. **C** Half-life determination in DMSO at 50 µM and 37 °C.

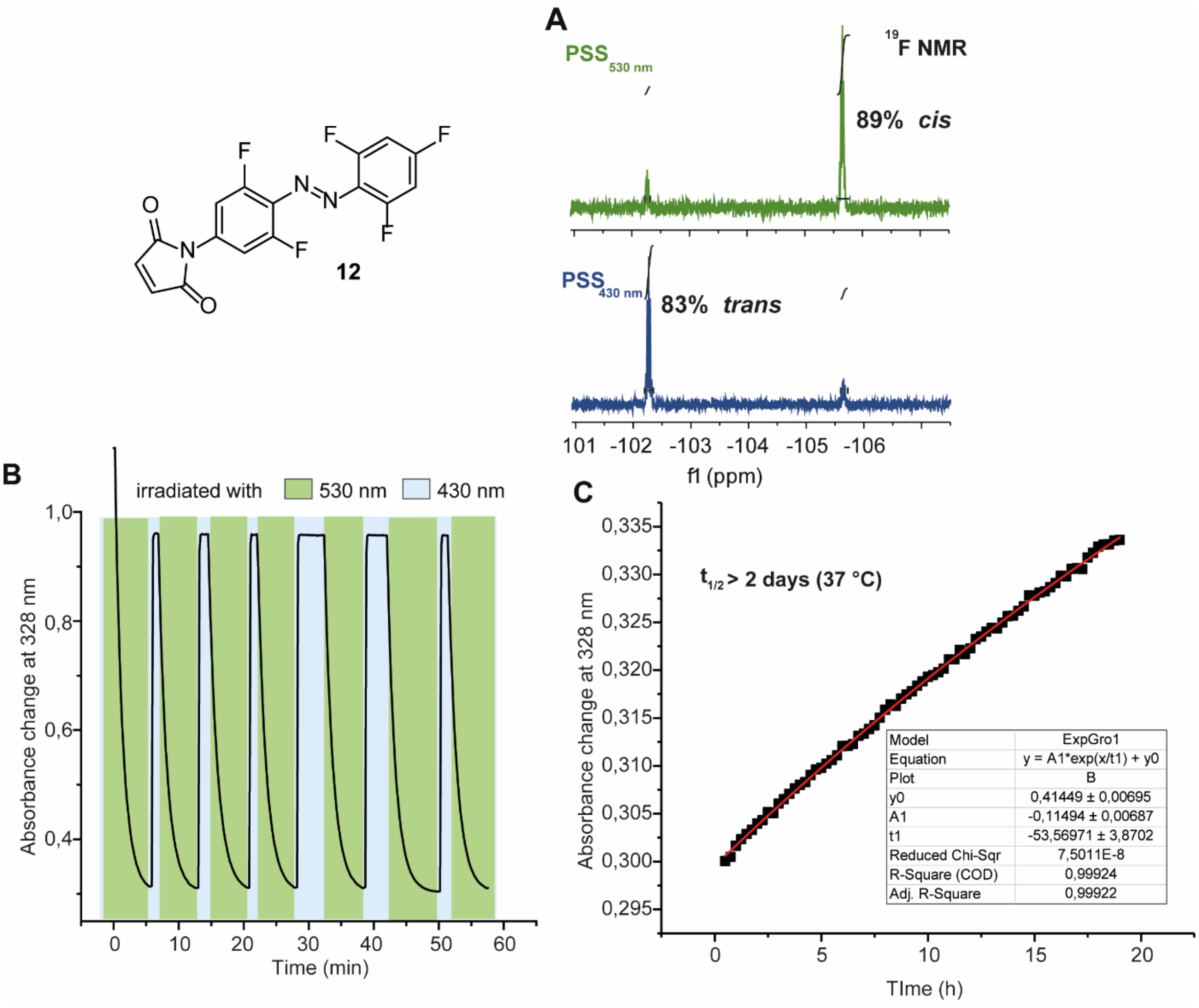

Figure S97. Determination of the photochemical properties of compound **12**. **A** PSS determination by ^{19}F NMR spectroscopy upon irradiation with 530 nm and 430 nm LED at rt, 5 mM in DMSO-d_6. **B** Fatigue resistance test upon irradiation with 530 and 430 nm LED in DMSO at 50 µM and 20 °C. **C** Half-life determination in DMSO at 50 µM and 37 °C.

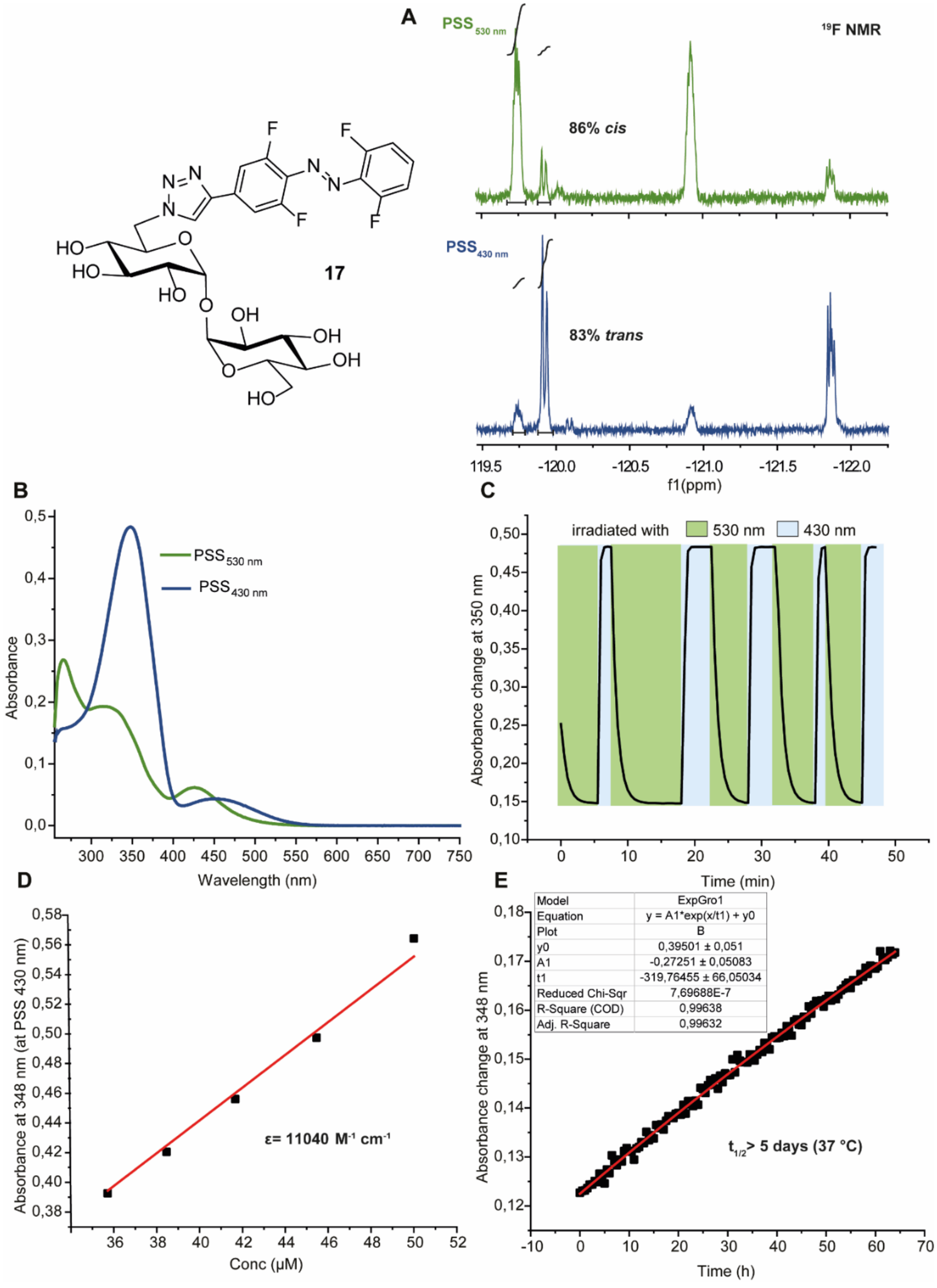

Figure S98. Determination of the photochemical properties of compound **17**. **A** PSS determination by ^{19}F NMR spectroscopy upon irradiation with 530 nm and 430 nm LED at rt, 2 mM in DMSO-d_6. **B** UV-Vis spectrum of **8** upon reaching PSS upon irradiation with 530 nm and 430 nm LED at 50 µM concentration in DMSO at 25 °C. **C** Fatigue resistance test upon irradiation with 530 and 430 nm LED in DMSO at 50 µM and 20 °C. **D** Determination of the molar absorptivity in DMSO at 20 °C. **E** Half-life determination in DMSO at 50 µM and 37 °C.

		S_0--S_1 λ_{max}		isosbestic point
		trans	*cis*	
**1**	**H-H**	446	416	444
**2**	**F-H**	455	417	443
**3**	**Br-H**	452	420	447
**4**	**SO_3^--H**	450	420	447
**5**	**Alkyne-H**	460	423	450

Figure S99. Comparison of S_0-S_1 absorption bands maxima for compounds **1**-**5** in DMSO.

6.2 Photochemical properties in aqueous medium

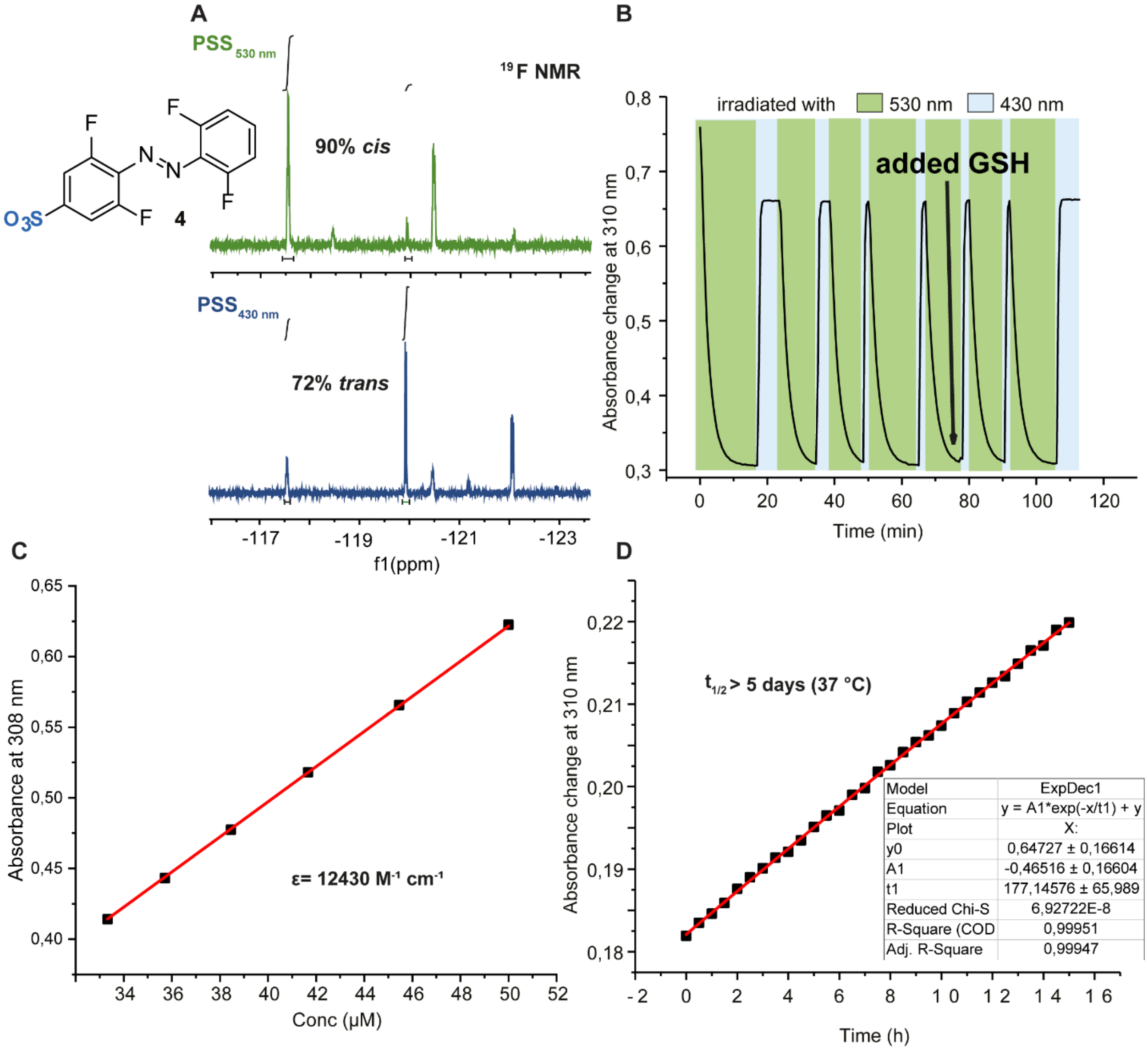

Figure S100. Determination of the photochemical properties of compound **4**. **A** PSS determination by ^{19}F NMR spectroscopy upon irradiation with 530 nm and 430 nm LED at rt, 2 mM in PBS buffer (25% D_2O). **B** Fatigue resistance and GSH stability (10mM) test upon irradiation with 530 and 430 nm LED in PBS buffer at 50 µM and 20 °C. **C** Determination of the molar absorptivity in PBS buffer at 20 °C. **D** Half-life determination in PBS buffer at 50 µM and 37 °C.

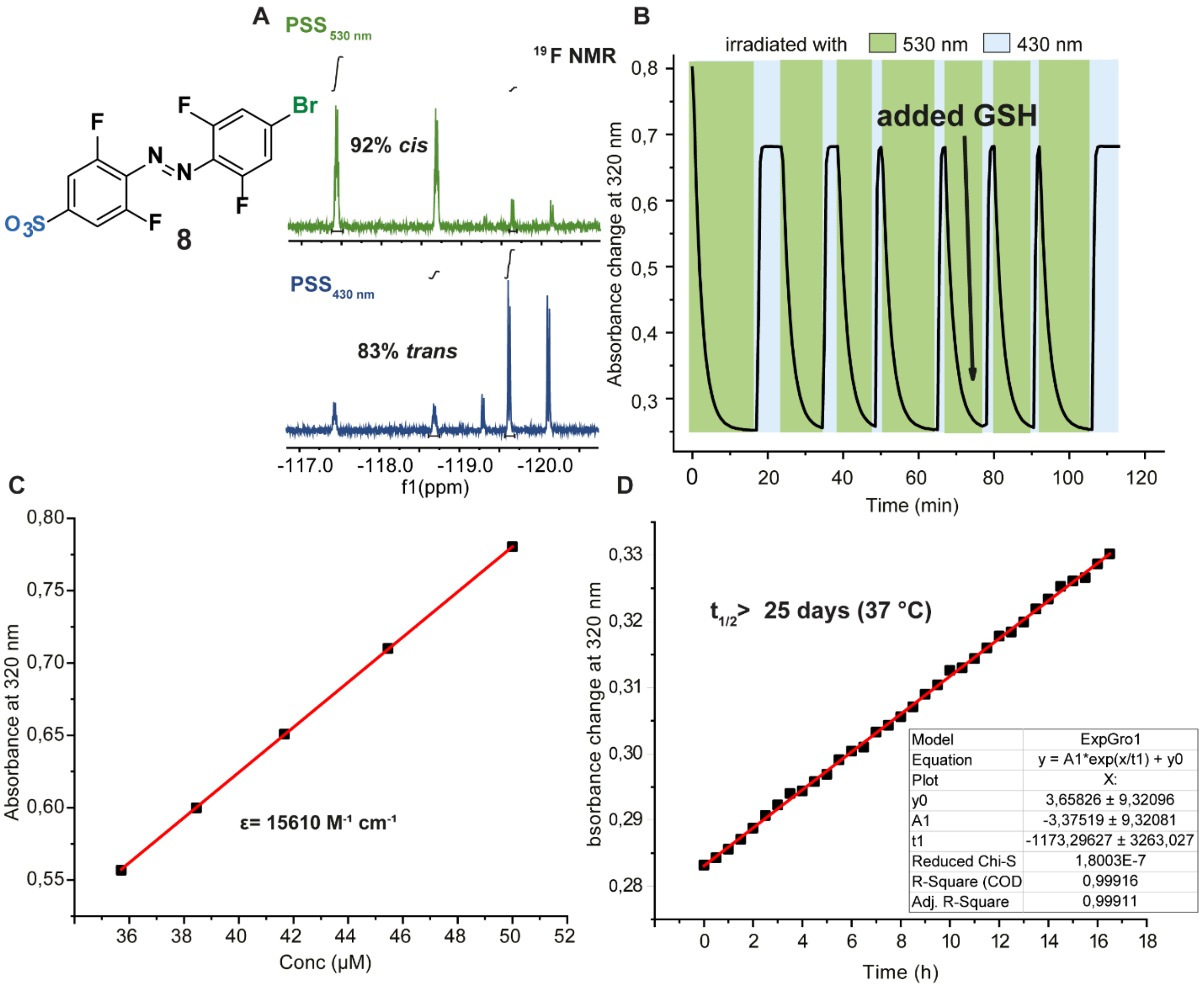

Figure S101. Determination of the photochemical properties of compound **8**. **A** PSS determination by ^{19}F NMR spectroscopy upon irradiation with 530 nm and 430 nm LED at rt, 2 mM in PBS buffer (25% D_2O). **B** Fatigue resistance and GSH stability (10mM) test upon irradiation with 530 and 430 nm LED in PBS buffer at 50 µM and 20 °C. **C** Determination of the molar absorptivity in PBS buffer at 20 °C. **D** Half-life determination in PBS buffer at 50 µM and 37 °C.

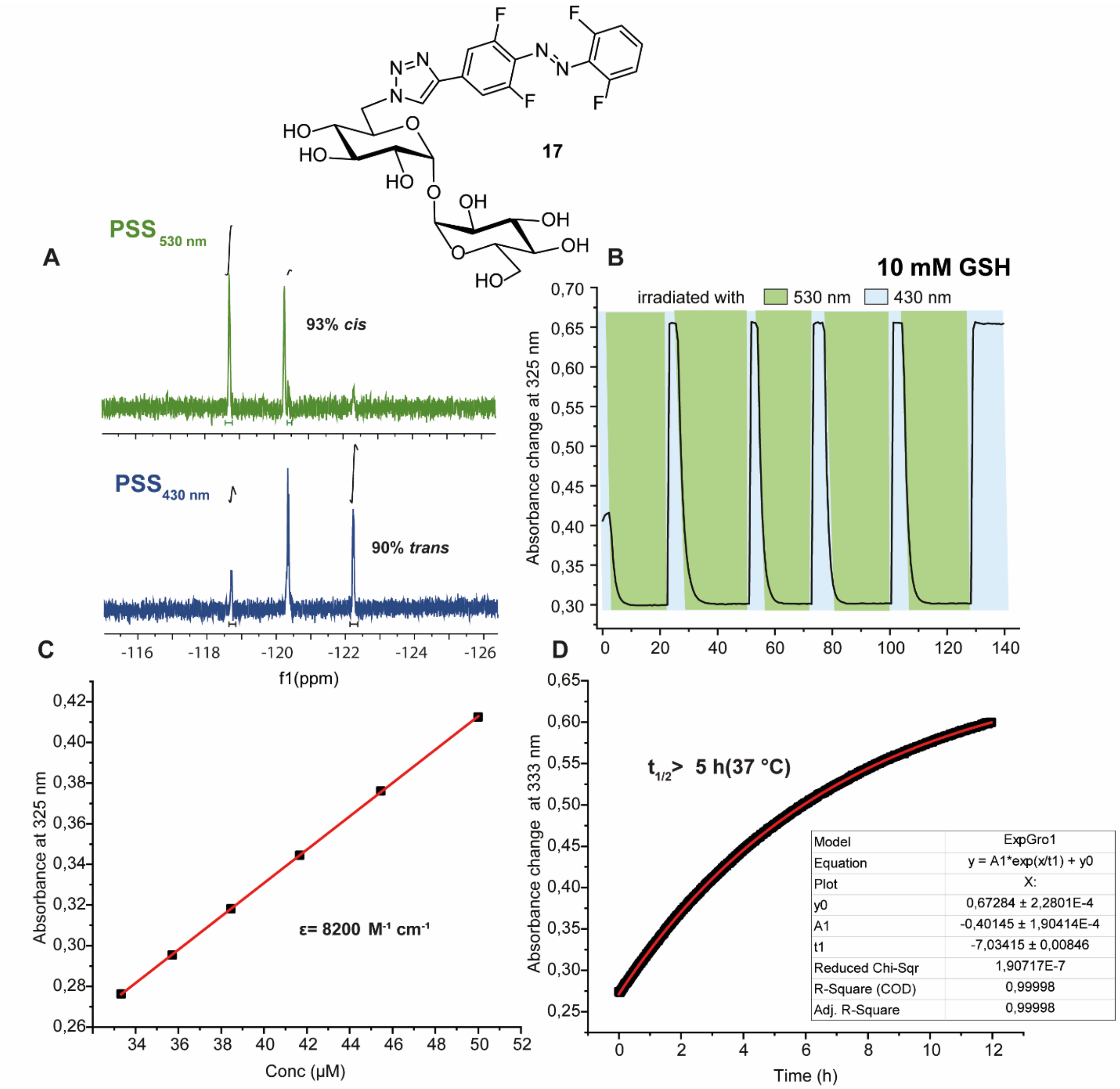

Model	ExpGro1
Equation	y = A1*exp(x/t1) + y0
Plot	X:
y0	0,67284 ± 2,2801E-4
A1	-0,40145 ± 1,90414E-4
t1	-7,03415 ± 0,00846
Reduced Chi-Sqr	1,90717E-7
R-Square (COD)	0,99998
Adj. R-Square	0,99998

Figure S102. Determination of the photochemical properties of compound **17**. **A** PSS determination by ^{19}F NMR spectroscopy upon irradiation with 530 nm and 430 nm LED at rt, 2 mM in PBS buffer (25% D_2O, 2.5 % DMSO-d_6). **B** Fatigue resistance and GSH stability (10mM) test upon irradiation with 530 and 430 nm LED in PBS buffer at 50 µM and 20 °C. **C** Determination of the molar absorptivity in PBS buffer at 20 °C. **D** Half-life determination in PBS buffer at 50 µM and 37 °C.

6.3 ^{19}F NMR spectroscopy of the unlabeled and labeled lipid extract

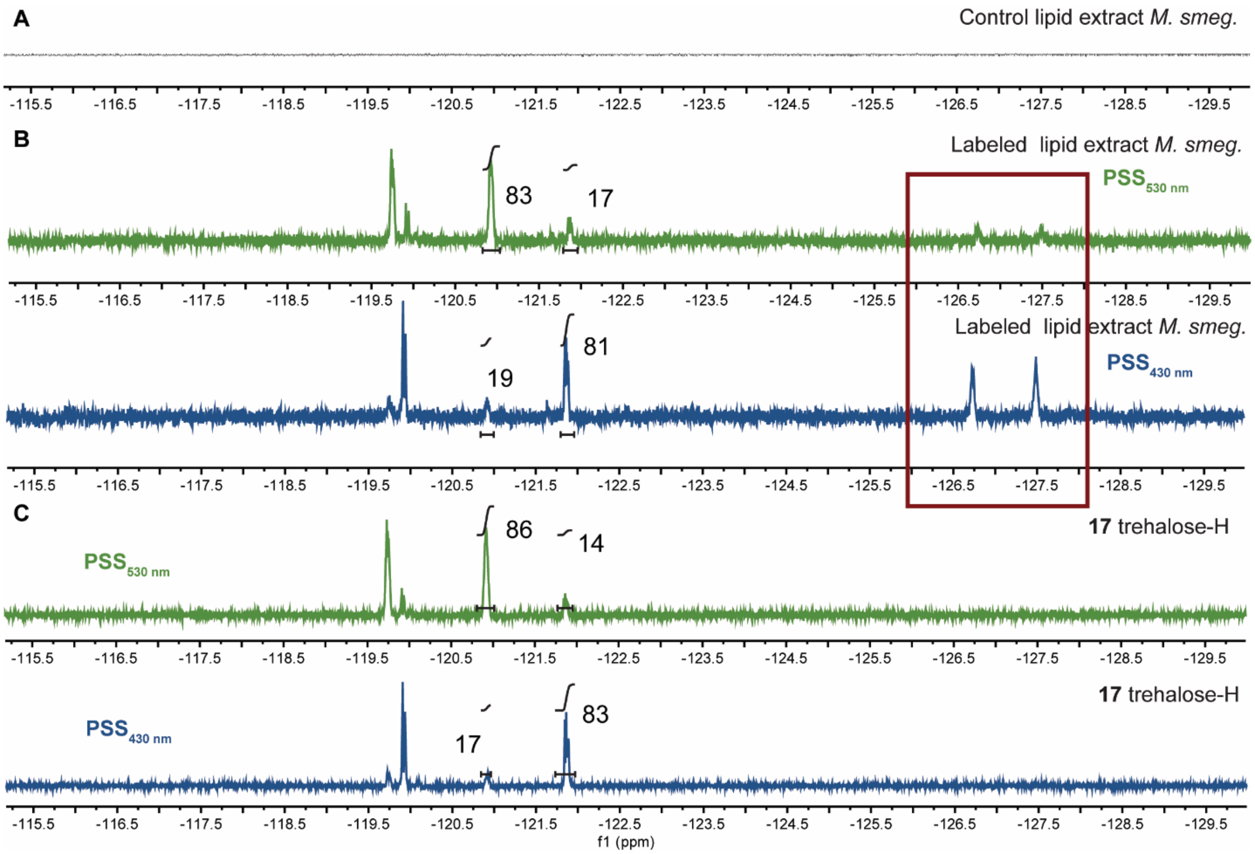

Figure S103. Irradiation study of the labeled lipid extract *of M. smeg*. in DMSO-d_6 and a comparison with the free probe. **A** ^{19}F NMR spectrum of the unlabeled lipid extract of *M. Smeg.* **B** ^{19}F NMR spectrum of the lipid extract of *M. Smeg.* labeled with probe **8** upon irradiation with 530 nm (up) and 430 nm (down) light where the newly appeared peaks are highlighted in the red box. **C** ^{19}F NMR spectrum of the free trehalose probe **17** upon irradiation with 530 nm (up) and 430 nm (down) light in DMSO-d_6.

7. References

[1] H. Helbert, P. Visser, J. G. H. Hermens, J. Buter, B. L. Feringa, *Nat. Catal.* **2020**, *3*, 664–671.

[2] A. Purkait, S. K. Roy, H. K. Srivastava, C. K. Jana, *Org. Lett.* **2017**, *19*, 2540–2543.

[3] I. G. Powers, J. M. Andjaba, X. Luo, J. Mei, C. Uyeda, *J. Am. Chem. Soc.* **2018**, *140*, 4110–4118.

[4] M. J. Hansen, M. M. Lerch, W. Szymanski, B. L. Feringa, *Angew. Chemie Int. Ed.* **2016**, *55*, 13514–13518.

[5] F. Zhao, L. Grubert, S. Hecht, D. Bléger, *Chem. Commun.* **2017**, *53*, 3323–3326.

[6] D. Mutruc, A. Goulet-Hanssens, S. Fairman, S. Wahl, A. Zimathies, C. Knie, S. Hecht, *Angew. Chemie Int. Ed.* **2019**, *58*, 12862–12867.

[7] C. R. Opie, N. Kumagai, M. Shibasaki, *Angew. Chemie* **2017**, *56*, 3397–3401.

[8] Q. Liu, H. Dong, Y. Li, H. Li, D. Chen, L. Wang, Q. Xu, J. Lu, *Chem. - An Asian J.* **2016**, *11*, 512–519.

[9] A. Antoine John, Q. Lin, *J. Org. Chem.* **2017**, *82*, 9873–9876.
